# Atroposelective Ir-Catalyzed C–H Borylation of Phthalazine Heterobiaryls

**DOI:** 10.1021/acs.joc.3c01534

**Published:** 2023-09-26

**Authors:** Paul Stehrer, Anke Spannenberg, Marko Hapke

**Affiliations:** † Institute for Catalysis (INCA), Johannes Kepler University Linz (JKU), Altenberger Strasse 69, 4040 Linz, Austria; ‡ Leibniz Institute for Catalysis e.V. (LIKAT), Albert-Einstein-Strasse 29a, 18069 Rostock, Germany

## Abstract

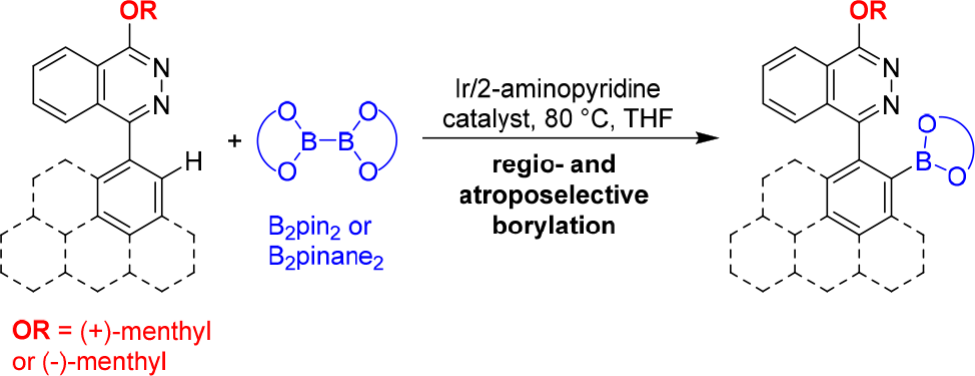

The atroposelective iridium-catalyzed borylation of menthyloxy-substituted phthalazine heterobiaryls with diborons is reported. Utilizing [Ir(OMe)(COD)]_2_/2-aminopyridine as a rarely used efficient catalyst system, the heterobiaryls were selectively borylated in the 2-position of the carbocycle, exclusively yielding only one of the atropisomers, depending on the substitution of the phthalazine with (+)-menthyl or (−)-menthyl moieties. Exemplary further functionalization of a borylated atropisomer demonstrated that nickel-catalyzed Suzuki-Miyaura cross-coupling with an aryl halide was able to provide stereoretention to a certain degree (up to 75% *de*).

The position-selective and stereoselective transition metal-catalyzed C–H borylation reaction has evolved into a highly useful methodology for the introduction of functional groups, especially for arene substrates but also for alkyl C–H bonds.^1^ The approach circumvents the use of functionalized substrates like aryl halides, where the halide can be transformed into the boronate by using organometallic bases (R-Li, Grignard reagents) and quenching with borates, transition metal-catalyzed processes with diborons,^2^ or other functional group transformations.^3^ Also, a number of transition metal-free processes have been reported lately, relying on electrophilic borylation.^4^ Borylation reactions have been used for the synthesis of active pharmaceutical ingredients.^5^ Within the evolution of the methodology, recently a lot of attention is focusing on developing position-selective borylation reactions for the *meta* or *para* position of arenes^6,7^ or for asymmetric borylation reactions.^8^

Asymmetric assembling of atropisomers by catalytic methodologies saw a huge advance over the last two decades.^9^ The synthesis of selectively borylated (hetero)biaryls using guidance by nitrogen atoms has been less developed^10^ but could deliver highly interesting building blocks for the assembly of functionalized molecules for photochemical applications.^11^ Substitution in the 2-position of a sufficiently large arene connected to a nitrogen-containing ring as directing group can increase the configurational stability of the biaryl axis. Under ideal conditions, it can be utilized for the preparation of chiral compounds, e.g., as demonstrated for dynamic kinetic resolution via asymmetric Heck reactions^12^ or the synthesis of enantiopure QUINAP derivatives by asymmetric catalysis.^13^

The synthesis of heterobiaryls containing other stereocenters beside the chiral axis potentially leads to resolvable diastereomers, which could be separated by, e.g., chromatographic methods.^14^ Since we are experienced in the *de novo* synthesis of heterobiaryls by [2+2+2] cycloaddition, our aim was to venture into the selective postfunctionalization of the 2-position of the carbocycles by C–H borylation using chiral diborons like **DB1** (see Table 1 for structure). Preliminary experiments suggested phthalazine heterocycles, utilized before as a heterobiaryl backbone in the assembly of PINAP ligands,^15^ as suitable test substrates for the borylation of the heterobiaryl carbocycle in the 2-position. The test substrates containing a chiral (+)- or (−)-menthol (**1**) group can be easily assembled from commercially available 1,4-dichloro-phthalazine in three steps via **2a** with a final Suzuki-Miyaura coupling (Scheme 1).^16^ The heterobiaryls (+)-**3a**–**k** and (−)-**3a**, **b**, and **e** were conveniently obtained by the same protocol with generally very good yields.

**Scheme 1:**
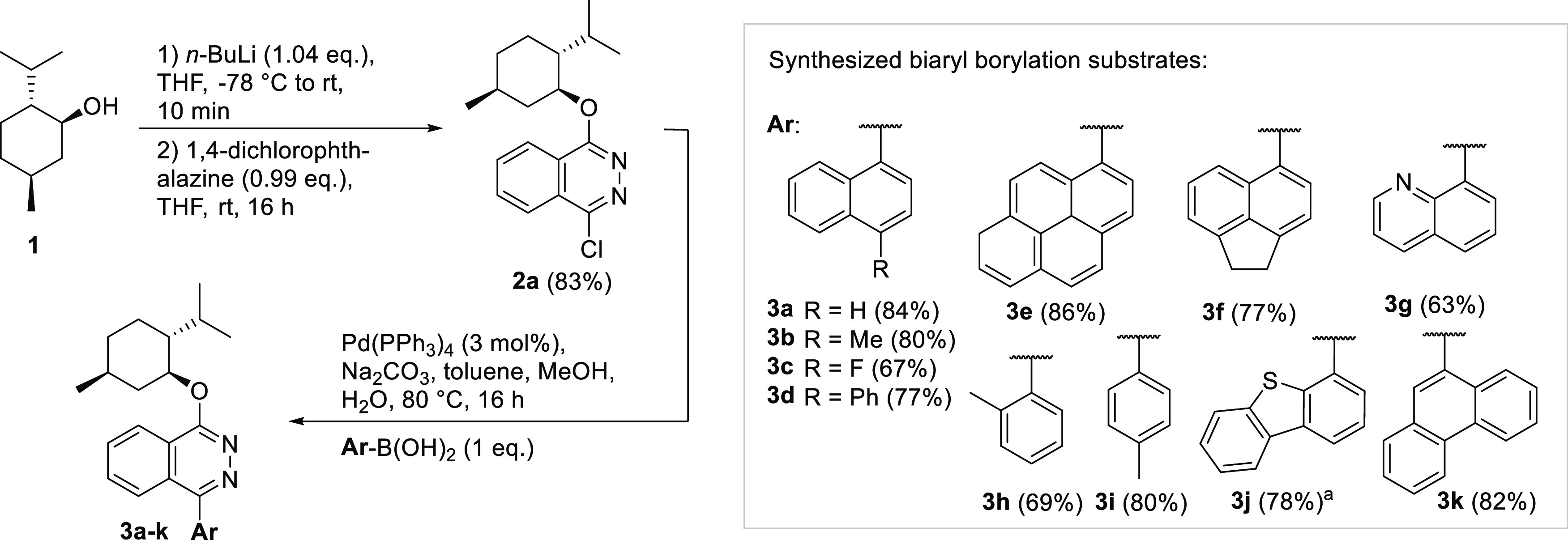
Exemplary Synthesis of (+)-Menthyloxy 1-naphthyl-phthalazines THF was used as solvent.

**Table 1:**
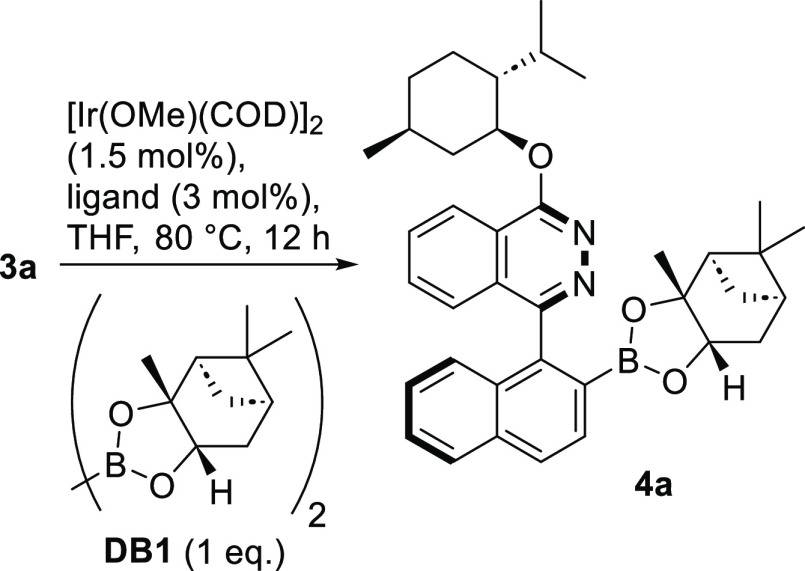
Screening of Ligands for Directed Borylation Reaction with Substrate **3a**

No.	Ligand	Yield [%]^a^
1	1,10-phenanthroline (**L1**)	(94)^b^
2	3,4,7,8-tetramethyl-1,10-phenanthroline (**L2**)	(67)^b^
3	2,2′-bipyridine (**L3**)	(68)^b^
4	4,4′-di-*tert*-butyl-2,2′-bipyridine (**L4**)	(79)^b^
5	2-aminopyridine (**L5**)	88
6	2-aminomethylpyridine (**L6**)	n.r.^c^
7	2-(dimethyamino)-pyridine (**L7**)	74
8	2-(benzylamino)-pyridine (**L8**)	87
9	2-(benzylimino)-pyridine (**L9**)	68

a Isolated yields.

b Reaction yielded mixture of indeterminable regioisomerically borylated heterobiaryls.

c No reaction and quantitative recovery of starting material.

In the next step, we investigated the optimal conditions for the Ir-catalyzed borylation of the prepared heterobiaryls using commercially available chiral diboron **DB1**. Table 1 shows the results of the ligand screening for the Ir-catalyzed process at the optimized temperature of 80 °C. Utilizing 2-aminopyridine (**L5**) as very rare ligand for C–H borylation gave excellent 88% yield exclusively of the desired regioisomer (contrary to entries 1–4).^17^ It came as a genuine surprise that only one stereoisomer was found as product, suggesting a sufficient energetical difference between diastereomers on an intermediary catalytic stage. Except for ligand **L6**, leading to no reaction, all other ligands **L7** to **L9** gave good to very good yields (entries 7–9, Table 1).

After having identified that the pyridine **L5** afforded the most suitable catalyst, we investigated the borylation of different substrates **3a**–**k** to evaluate the role of the chiral menthyloxy substitutent as well as the chiral diboron **DB1**, its enantiomer **DB2**, and bis(pinacolato)diboron (**DB3**) in more detail. The results are displayed in Scheme 2. The most intriguing observation is the dependence of the configuration of the biaryl axis from the configuration of the menthyloxy moiety attached to the phthalazine ring while overriding any effect of the chiral diborons **DB1**/**DB2**, thus also allowing selective borylations using achiral B_2_pin_2_ (**DB3**).

**Scheme 2:**
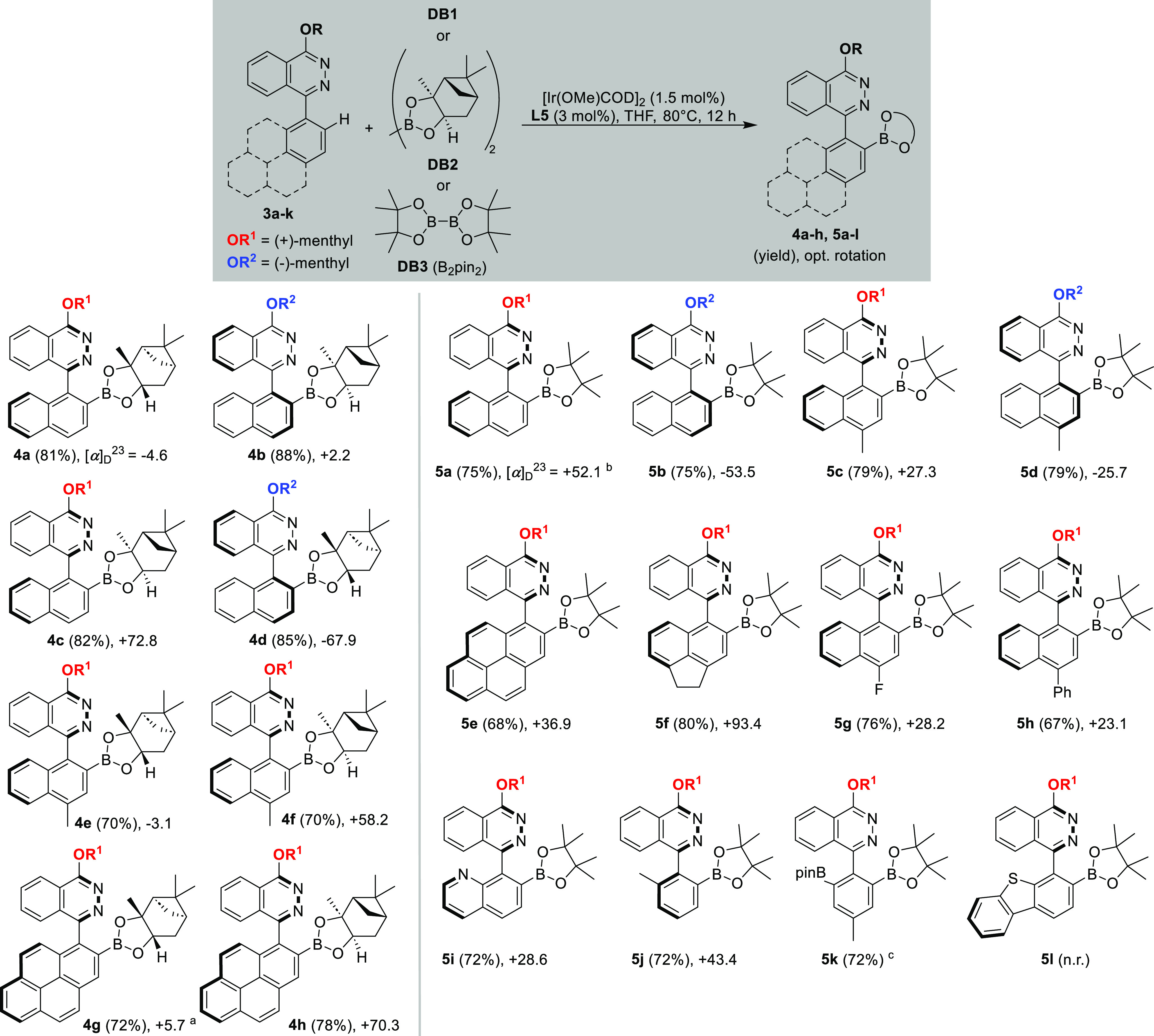
Synthesis of Borylated Phthalazine Derivatives with Chiral Diborons **DB1** and **DB2** and Achiral B_2_pin_2_ (**DB3**) Repeated on a 1 mmol scale, providing **4g** in 77% yield. Repeated on a 1 mmol scale, providing **5a** in 85% yield. 2.0 equiv of B_2_pin_2_ used.

While (+)-menthyloxy substitution led to the (*R*_*a*_)-configuration (compounds **4a**, **c**, **e**, **f**, **g**, **h**), the introduction of a (−)-menthyloxy substituent led to the (*S*_*a*_)-configuration (compounds **4b**, **d**), which was derived from structure determination in the series of heterobiaryls **5** (*vide infra*). The isolated yields for the heterobiaryls **4** are in general very good and range from 70 to 88% (Scheme 2, left column).^18^ Replacing the chiral diborons **DB1** and **DB2** by B_2_pin_2_ (**DB3**) to evaluate the role of the chiral boryl group demonstrated that atroposelective borylations were also occurring in this case (**5a**–**k**, Scheme 2, right column). The dibenzothiophene **3j** and phenanthrene **3k** were either not reactive in the borylation (e.g., to **5l**) or underwent unselective borylations.^19,20^ These experiments therefore established that the chiral boron moiety does not exert a direct influence on the selectivity of the C–H borylation process; however, the sterically crowded iridium-boryl complex species might pronounce the effect of the chiral menthyl auxiliar for the borylation process.^21^ Assignation of the absolute configuration of the biaryl axis was possible by SC-XRD of compound **5c**, unambiguously confirming the (*R*_*a*_)-configuration of the biaryl axis (see Figure S1, Supporting Information).^22,23^

Finally, we exemplarily investigated the further functionalization of the 2-boronate function from either the **4g** or **4k-SI** atropisomer by a Suzuki-Miyaura cross-coupling reaction to evaluate the configurational stability of the biaryl axis in the products **6a** (Table 2). Such C–C coupling reactions with chiral boronic esters are very rare.^24^ We turned our attention to nickel-catalyzed cross-coupling reactions at lower reaction temperatures with 4-haloacetophenones to **6a** (entries 1–4, Table 2).^25^ Application of Ni[P(*n*-Bu)_3_]_2_(COD) as nickel precursor together with 1,4-bis(diphenylphosphino)butane (dppb) as ligand increased the yield as well as even more significantly the *de* value for **6a** to 75% as the best case; beneficially, the aryl bromide as well as iodide reacted identically (entries 2–4, Table 2). We investigated lower reaction temperatures to improve the selectivity and found the reaction with these catalysts to be possible even at room temperature, unfortunately without improvement of the *de* value (entries 5 and 6, Table 2). Shorter reaction times do not improve the stereoretention at lower yields (entries 7 and 8, Table 2). However, the developed catalytic system represents one of the very few nickel-catalyzed Suzuki-Miyaura couplings successfully working at mild reaction temperatures with Bpinane esters.

**Table 2:**
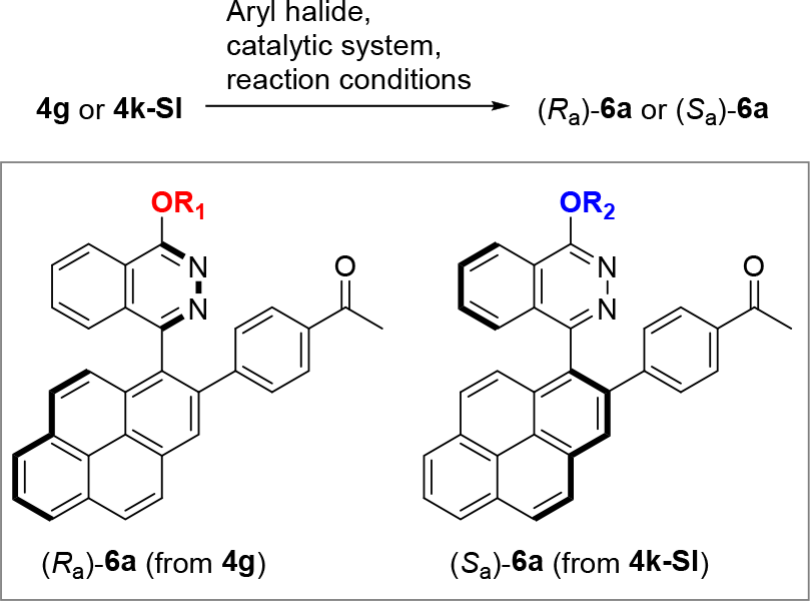
Cross-coupling Reactions with Borylated Atropisomers

No.	Atropisomer/Aryl halide/Catalyst system/Conditions	Yield [%]	Stereoret. [% *de*]
1	**4g**, 4-Iodoacetophenone, Ni(COD)_2_, dppb, 2-MeTHF/H_2_O, *i*-Pr_2_EtN, 50 °C, 12 h	75	25
2	**4g**, 4-Bromoacetophenone, Ni[P(*n*-Bu)_3_]_2_(COD), dppb, 2-MeTHF/H_2_O, *i*-Pr_2_EtN, 50 °C, 12 h	81	74
3	**4g**, 4-Iodoacetophenone, Ni[P(*n*-Bu)_3_]_2_(COD), dppb, 2-MeTHF/H_2_O, *i*-Pr_2_EtN, 50 °C, 12 h	82	75
4	**4k-SI**, 4-Iodoacetophenone, Ni[P(*n*-Bu)_3_]_2_(COD), dppb, 2-MeTHF/H_2_O, *i*-Pr_2_EtN, 50 °C, 12 h	82	67
5	**4g**, 4-Iodoacetophenone, Ni[P(*n*-Bu)_3_]_2_(COD), 2-MeTHF/H_2_O, *i*-Pr_2_EtN, rt, 12 h	67	72
6	**4g**, 4-Iodoacetophenone, Ni(dppb)(COD), 2-MeTHF/H_2_O, *i*-Pr_2_EtN, rt, 12 h	85	71
7	**4g**, 4-Iodoacetophenone, Ni(dppb)(COD), 2-MeTHF/H_2_O, *i*-Pr_2_EtN, 50 °C, 0.5 h	10	70
8	**4g**, 4-Iodoacetophenone, Ni(dppb)(COD), 2-MeTHF/H_2_O, *i*-Pr_2_EtN, 50 °C, 2 h	33	69

In summary, we could demonstrate the ability of menthyl groups as convenient chiral auxiliaries in menthyloxy-substituted phthalazine heterobiaryls to direct the stereo- and position-selective C–H borylation with achiral and chiral diborons. The chirality of the used diborons did not play a role for the stereoselectivity of the borylation process. A rarely used *in situ*-generated Ir(I)-2-aminopyridine catalytic system proved to be most efficient for the process. Further functionalization of the atropisomeric 2-aryl-borylated heterobiaryls by nickel-catalyzed Suzuki-Miyaura coupling reaction with aryl halides at room temperature led to up to 75% *de* and 82% yield for the triaryl coupling product.

## Data Availability

The data underlying this study are available in the published article and its Supporting Information.

## Supporting Information Available

Experimental procedures, synthesis of starting materials, data of screening experiments, compound characterization data, SC-XRD data for **5c**, NMR spectra (PDF)

## Dedication

Dedicated to the 60th birthday of Prof. Dr. Matthias Beller.
