# Supplementary material for: Atroposelective Ir-Catalyzed C–H Borylation of Phthalazine Heterobiaryls
Source: J Org Chem. 2023 Sep 26;88(19):14222–6. doi: 10.1021/acs.joc.3c01534 (PMC10563123; doi:10.1021/acs.joc.3c01534)
Supplement: Supplementary file 1 — jo3c01534_si_001.pdf [file jo3c01534_si_001.pdf]

Supporting Information

Atroposelective Ir-catalyzed C-H borylation of phthalazine heterobiaryls

Paul Stehrer,[§] Anke Spannenberg[#] and Marko Hapke[*§,#]

[§] Institut für Katalyse (INCA), Johannes Kepler University Linz, Altenberger Str. 69, A-4040 Linz (Austria)

[#] Leibniz-Institut für Katalyse e.V. (LIKAT), Albert-Einstein-Str. 29a, D-18059 Rostock (Germany)

E-mail corresponding author: marko.hapke@jku.at

Table of Contents

1. General Methods SI-2
2. Synthesis of Substrates SI-3
2.1. Syntheses of Chiral Diboranes SI-3
2.2. Syntheses of Aryl and Biaryl Substrates and Derivatives SI-4
3. Screening of Conditions for Iridium-catalyzed C-H Borylation of Biaryl Substrates SI-17
4. Syntheses of Chiral Borylated Heterobiaryls SI-19
4.1. C-H Borylation with Chiral Diborons SI-19
4.2. C-H Borylation with B_2pin_2 (**DB3**) and Related Diborons SI-29
4.3. Synthesis and C-H borylation of problematic substrate motif SI-42
5. Cross-coupling Reactions of Borylated Atropisomers SI-48
6. SC-XRD-Data SI-52
7. NMR Spectra SI-54
References SI-108

1. General Methods

All experiments were carried out under an inert gas atmosphere in flame-dried Schlenk tubes or glass reaction vials using either standard Schlenk-line technique or an argon-filled glovebox unless stated otherwise. The anhydrous solvents (dichloromethane, *n*-hexane, tetrahydrofuran and toluene) were dried in a solvent purification system MB SPS7 from MBraun and degassed via three cycles of freeze-pump-thaw. 1,4-Dioxane, 1,2-dichloroethane, and 2-methyl tetrahydrofurane (2-Me-THF) were purchased from Acros Chemicals. Bispinacolato diboron (B_2pin_2) was purchased from ChemPur Chemicals and further purified via sublimation. $[IrCl(COD)]_2$ and $[Ir(OMe)(COD)]_2$ were synthesized according to the literature.[1] All other chemicals (Aldrich, Aurora Fine Chemicals LLC, BLDPharm, Chempur Chemicals, Fisher Scientific, Fluorochem, Merck and TCI) were purchased and used as received. All reactions were heated using a temperature-controlled oil bath and a magnetic stirrer.

Thin-layer chromatography was performed on Merck 60 F254 silica gel plates or ALUGRAM ALOX N / UV 254 plates with a layer size of 0.2 mm from MACHERY-NAGEL. Column chromatography utilized Silica 60 with 0.04-0.063 mm particle size.

NMR spectra were recorded on a Bruker Avance III 300 MHz spectrometer or Bruker Avance III 500 MHz spectrometer with a broad band observe probe and were referenced to the residual solvent peak $CHCl_3$ (δ = 7.26 ppm for ^1H and 77.0 ppm for ^{13}C spectra). For the ^{13}C spectra of **5a** to **5l** a doubling of peaks was observed in the region between 42-16 ppm, which were elucidated to be rotamers of the menthyloxy moiety via several NMR studies of compound **5e**. Structural assignments were made with additional information from ^1H NMRs ranging from -10°C to +60°C as well as quantitative ^{13}C NMR and HSQC of compound **5e**. The rotamer peaks are marked by an asterisk ($^{\#}$) accordingly in the subsequent text.

HRMS (ESI) was performed on a Thermo Fisher Scientific LTQ Orbitrap XL and HRMS (EI) on an Agilent 1200/6210 ToF LC-MS. Optical rotation was measured on a Schmidt + Haensch UniPol L1000.

2. Synthesis of Substrates

(E)-1-Phenyl-N-(pyridine-2-yl)methanimine (L9)

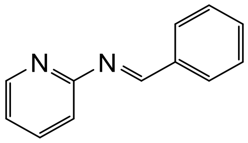

In the glovebox, 2-aminopyridine (1 mmol, 94.1 mg), benzaldehyde (1 mmol, 101.8 µL, 1 equiv.), mol sieves (4 Å) (50 mg) and 3 mL of toluene were weighed in a 10 mL Schlenk flask. The reaction mixture was refluxed for 2 h, cooled to room temperature and filtered through a Whatman filter. The filtrate was concentrated via reduced pressure. After recrystallization from Et_2O, 82 mg (0.45 mmol) of a white powder was obtained with a yield of 45%. The NMR data are according to the literature.[2]

^{1}H NMR (300 MHz, $CDCl_3$): δ = 9.16 (s, 1H), 8.50 (dd, *J* = 4.7, 1.7 Hz, 1H), 8.00 (dd, *J* = 7.4, 1.7 Hz, 2H), 7.80-7.73 (m, 1H), 7.53-7.44 (m, 3H), 7.34 (d, *J* = 7.9 Hz, 1H), 7.19 (ddd, *J* = 7.4, 4.9, 0.9 Hz, 1H).

2.1. Syntheses of Chiral Diboranes

For the initial investigations bis[(+)-pinanediolato]diboron (**DB1**) and bis[(-)-pinanediolato]diboron (**DB2**) were purchased from BLDPharm and used without further purification. No difference in yield and selectivity could be observed while using self-synthesized versus purchased diboronic ester **DB1** and **DB2** in the subsequent experiments.

Bis[(+)-pinanediolato]diboron (DB1)

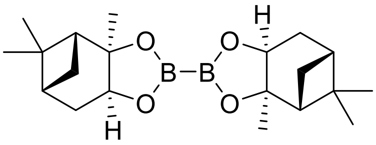

In the glovebox hypodiboric acid (1.0 mmol, 0.089 g), (1S,2S,3R,5S)-2,6,6-trimethylbicyclo[3.1.1]heptane-2,3-diol (2.0 mmol, 0.370 g, 2 equiv.), mol sieves (4 Å) (0.5 g) as well as 6 mL THF were put in a 10 mL Schlenk flask. After stirring overnight at 70 °C, the sample was filtered via a paper filter. The filtrate was concentrated and recrystallized in *n*-hexane. After isolation and drying 0.320 g (0.89 mmol) of a white powder was isolated with a yield of 89%. The NMR data are according to the literature.[3]

^{1}H NMR (300 MHz, $CDCl_3$): δ = 4.26 (dd, *J* = 8.7, 1.6 Hz, 2H), 2.37-2.25 (m, 2H), 2.24-2.14 (m, 2H), 2.10-2.05 (m, 2H), 1.97-1.85 (m, 4H), 1.40 (s, 6H), 1.28 (s, 6H), 1.11 (d, *J* = 11.0 Hz), 0.84 (s, 6H).

Bis[(-)-pinanediolato]diboron (DB2)

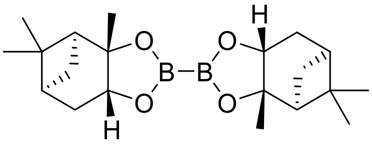

In the glovebox hypodiboric acid (1.0 mmol, 0.089 g), (1R,2R,3S,5R)-2,6,6-trimethylbicyclo[3.1.1]heptane-2,3-diol (2.0 mmol, 0.370 g, 2 equiv.), mol sieves (4 Å) (0.5 g) as well as 6 mL THF were put in a 10 mL Schlenk flask. After stirring overnight at 70 °C, the sample was filtered via a paper filter. The filtrate was concentrated and recrystallized in *n*-hexane. After isolation and drying 0.319 g (0.89 mmol) of a white powder was obtained with a yield of 89%. The NMR data are in accordance with the literature.[4]

^{1}H NMR (300 MHz, $CDCl_3$): δ = 4.26 (dd, *J* = 8.7, 1.2 Hz, 2H), 2.37-2.25 (m, 2H), 2.25-2.14 (m, 2H), 2.11-2.04 (m, 2H), 1.97-1.85 (m, 4H), 1.40 (s, 6H), 1.28 (s, 6H), 1.11 (d, *J* = 10.9 Hz), 0.84 (s, 6H).

2.2. Syntheses of Aryl and Biaryl Substrates and Derivatives

General Procedure 1 (GP1) – S_NAr of aromatic halides with alcohols.

According to a modified literature procedure,[5] in a dry 250 mL Schlenk flask, the respective chiral alcohol (10.1 mmol, 1.01 equiv.)[6] was dissolved in 110 mL of dry THF and cooled to -78 °C. After the mixture was stirred for 20 minutes, *n*-BuLi (1.6 M in hexane) (10.5 mmol, 6.7 mL, 1.05 equiv.) was added dropwise over 15 minutes. The mixture was allowed to warm to room temperature, before adding 1,4-dichlorophthalazine (10.0 mmol, 1.99 g, 1.equiv.) dissolved in 50 mL dry THF, dropwise. After stirring overnight at 35 °C, the reaction was quenched with sat. $NaHCO_3$, the aqueous phase was extracted thrice with EtOAc. The combined organic phases were dried over Na_2SO_4. Purification was achieved by column chromatography over silica.

Substrate (+)-2a

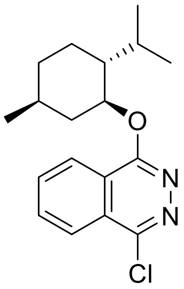

Following **GP1**, (+)-menthol (10.1 mmol, 1.578 g, 1.01 equiv.) was dissolved in 110 mL of dry THF, *n*-BuLi (1.6 M in hexane) (10.5 mmol, 6.7 mL, 1.05 equiv.) 1,4-dichlorophthalazine (10.0 mmol, 1.99 g, 1.equiv.) dissolved in 50 mL dry THF, were employed. After aqueous work-up, column chromatography over silica using *n*-heptane/EtOAc (3:1 v/v) as eluent was performed and 2.66 g (8.32 mmol) of a tan solid in a yield of 83% was obtained.

^{1}H NMR (300 MHz, $CDCl_3$): δ = 8.26-8.12 (m, 2H), 7.97-7.84 (m, 2H), 5.49-5.36 (m, 1H), 2.52-2.38 (m, 1H), 2.18-2.02 (m, 1H), 1.84-1.53 (m, 5H), 1.28-0.88 (m, 10H), 0.82-0.77 (m, 3H).

^{13}C{^{1}H} NMR (75 MHz, $CDCl_3$) (mixture of diastereomers): δ = 160.2, 149.3, 133.1, 132.8, 127.9, 125.2, 123.8, 122.1, 71.6, 47.8, 40.2, 34.5, 31.4, 26.9, 24.0, 23.2, 22.2, 20.8, 17.0.

$[\alpha]_D^{23}$ = 100.2 (*c* 1.0, $CHCl_3$)

HRMS (ESI): Calcd. for $C_{18}H_{23}N_2O_1Cl_1$ [M+H]+: 319.1571. Found: 319.1574.

Substrate (-)-2a

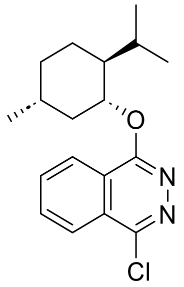

Following **GP1**, (-)-menthol (10.1 mmol, 1.578 g, 1.01 equiv.) was dissolved in 110 mL of dry THF, *n*-BuLi (1.6 M in hexane) (10.5 mmol, 6.7 mL, 1.05 equiv.) 1,4-dichlorophthalazine (10.0 mmol, 1.99 g, 1.equiv.) dissolved in 50 mL dry THF, were employed. After aqueous work-up, column chromatography over silica using *n*-heptane/EtOAc (3:1 v/v) as eluent was performed and 2.66 g (8.32 mmol) of a tan solid in a yield of 83% was obtained.

^{1}H NMR (300 MHz, $CDCl_3$): δ = 8.26-8.12 (m, 2H), 7.97-7.84 (m, 2H), 5.49-5.36 (m, 1H), 2.52-2.38 (m, 1H), 2.18-2.02 (m, 1H), 1.84-1.53 (m, 5H), 1.28-0.88 (m, 10H), 0.82-0.77 (m, 3H).

^{13}C{^{1}H} NMR (75 MHz, $CDCl_3$) (mixture of diastereomers): δ = 160.2, 149.3, 133.1, 132.8, 127.9, 125.2, 123.8, 122.1, 71.6, 47.8, 40.2, 34.5, 31.4, 26.9, 24.0, 23.2, 22.2, 20.8, 17.0.

$[\alpha]_D^{23}$ = -100.1 (*c* 1.0, $CHCl_3$)

HRMS (ESI): Calcd. for $C_{18}H_{23}N_2OCl$ [M+H]+: 319.1571. Found: 319.1579.

General Procedure (GP2) for the synthesis of substrates 3 – Cross-coupling protocol.

In accordance with the literature[7], in a Schlenk flask, aryl halide (1 mmol), aryl boronic acid (1.1 equiv.), Na_2CO_3 (2 equiv.) and $Pd(PPh_3)_4$ (3 mol%) were weighed in the glovebox. The resulting mixture was suspended in a mixture of degassed toluene, degassed MeOH as well and degassed H_2O. After stirring overnight at 80 °C, the reaction was quenched with H_2O, the aqueous phase was extracted thrice with ethyl acetate and the combined organic phases were dried over Na_2SO_4. Purification was achieved by column chromatography over silica using *n*-heptane/EtOAc as eluent.

Substrate (+)-3a

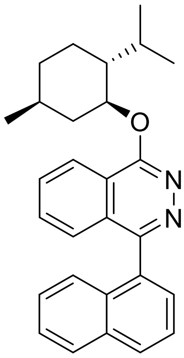

Following **GP2**, (+)-**2a** (5.0 mmol, 1.60 g), naphthalene-1-ylboronic acid (6 mmol, 0.98 g, 1.1 equiv.), Na_2CO_3 (10.0 mmol, 1.06 g, 2 equiv.) and $Pd(PPh_3)_4$ (3 mol%, 173 mg) dissolved in 10 mL degassed toluene, 2.5 mL degassed MeOH as well as 10 mL degassed H_2O were utilized. After aqueous work-up, column chromatography over silica using *n*-heptane/EtOAc (3:1 v/v) as eluent was performed and 1.72 g (4.20 mmol) of a tan solid in a yield of 84% was obtained.

^{1}H NMR (300 MHz, $CDCl_3$): δ = 8.34 (d, *J* = 8.0 Hz, 1H), 8.02 (dd, *J* = 6.0, 3.5 Hz, 1H), 7.95 (d, *J* = 8.1 Hz, 1H), 7.86 (t, *J* = 7.3 Hz, 1H), 7.69-7.59 (m, 3H), 7.55-7.41 (m, 3H), 7.35 (ddd, *J* = 8.3, 7.0, 1.3 Hz, H), 5.66-5.50 (m, 1H), 2.71-2.60 (m, 1H), 2.34-2.12 (m, 1H), 1.88-1.52 (m, 6H), 1.34-0.77 (m, 17H).

^{13}C{^{1}H} NMR (75 MHz, $CDCl_3$) (mixture of diastereomers): δ = 159.9, 155.7, 133.7, 132.6, 132.0, 131.7, 129.4, 128.5, 128.4, 126.6, 126.5, 126.2, 126.1, 125.4, 123.3, 120.4, 48.1, 48.0, 40.6, 40.4, 34.7, 31.5, 27.0, 24.1, 22.3, 21.06, 21.01, 17.23, 17.18.

$[\alpha]_D^{23}$ = +77.0 (*c* 1.0, $CHCl_3$)

HRMS (ESI): Calcd. for $C_{28}H_{30}$ N_2O_1 [M+H]+: 411.2428. Found: 411.2432.

Substrate (+)-3b

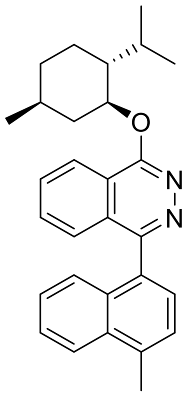

Following **GP2**, (+)-**2a** (1.0 mmol, 319 mg), (4-methyl naphthalene-1-yl)boronic acid (1.1 mmol, 205 mg, 1.1 equiv.), Na_2CO_3 (2.0 mmol, 212 mg, 2 equiv.) and $Pd(PPh_3)_4$ (3 mol%, 34.5 mg) dissolved in 2 mL degassed toluene, 0.5 mL degassed MeOH as well as 2 mL degassed H_2O were employed. After aqueous work-up, column chromatography over silica using *n*-heptane/EtOAc (3:1 v/v) as eluent was performed and 341 mg (0.80 mmol) of an off-white solid in a yield of 80% was obtained.

^{1}H NMR (300 MHz, $CDCl_3$): δ = 8.33 (d, *J* = 8.1 Hz, 1H), 8.11 (dd, *J* = 8.2 Hz, 1H), 7.84 (t, *J* = 7.5 Hz, 1H), 7.63 (t, *J* = 7.9 Hz, 1H), 7.57-7.42 (m, 5H), 7.35 (t, *J* = 7.5 Hz, 1H), 5.66-5.50 (m, 1H), 2.81 (s, 3H), 2.69-2.54 (m, 1H), 2.31-2.10 (m, 1H), 1.86-1.50 (m, 6H), 1.34-0.77 (m, 18H).

^{13}C{^{1}H} NMR (75 MHz, $CDCl_3$) (mixture of diastereomers): δ = 159.9, 155.7, 133.4, 132.6, 132.0, 131.7, 129.4, 128.5, 128.4, 126.6, 126.5, 126.2, 126.1, 125.4, 123.3, 120.4, 48.1, 48.0, 40.6, 40.4, 34.7, 31.5, 27.0, 34.1, 22.3, 21.1, 21.0, 17.22, 17.19.

$[\alpha]_D^{23}$ = +72.7 (*c* 1.0, $CHCl_3$)

HRMS (ESI): Calcd. for $C_{29}H_{32}N_2O_1$ [M+H]+: 425.2584. Found: 425.2587.

Substrate (+)-3c

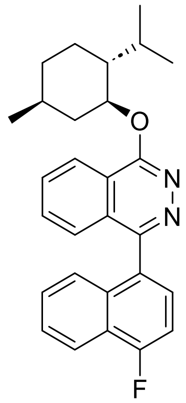

Following **GP2**, (+)-**2a** (1.0 mmol, 319 mg), (4-fluoronaphthalene-1-yl)boronic acid (1.1 mmol, 209 mg, 1.1 equiv.), Na_2CO_3 (2.0 mmol, 212 mg, 2 equiv.) and $Pd(PPh_3)_4$ (3 mol%, 34.5 mg)

dissolved in 2 mL degassed toluene, 0.5 mL degassed MeOH as well as 2 mL degassed H_2O were employed. After aqueous work-up, column chromatography over silica using *n*-heptane/EtOAc (3:1 v/v) as eluent was performed and 285 mg (0.67 mmol) of an off-white solid in a yield of 67% was obtained.

^{1}H NMR (300 MHz, $CDCl_3$): δ = 8.35 (d, *J* = 8.4 Hz, 1H), 8.22 (d, *J* = 8.2 Hz, 1H), 7.82 (ddd, *J* = 8.1, 7.1, 1.0 Hz, 1H), 7.70-7.49 (m, 4H), 7.47-7.36 (m, 2H), 7.35-7.24 (m, 1H), 5.66-5.50 (m, 1H), 2.76-2.55 (m, 1H), 2.40-2.10 (m, 1H), 1.99-1.53 (m, 5H), 1.37-1.13 (m, 2H), 1.13-0.77 (m, 12H).

^{13}C{^{1}H} NMR (75 MHz, $CDCl_3$) (mixture of diastereomers): δ = 160.4, 159.9, 158.4, 155.1, 134.0, 132.0, 131.7, 130.3, 129.3, 128.4, 127.4, 126.4, 126.3, 126.2, 124.0, 123.8, 123.4, 120.9, 120.8, 120.4, 109.2, 109.0, 71.6, 48.02, 47.98, 40.5, 40.4, 34.7, 31.5, 27.0, 24.1, 22.2, 21.00, 20.96, 17.21, 17.16.

^{19}F NMR (282 MHz, $CDCl_3$): δ = -122.5.

$[\boldsymbol{\alpha}]_D^{23}$ = +61.4 (*c* 1.0, $CHCl_3$)

HRMS (ESI): Calcd. for $C_{28}H_{29}N_2O_1F_1$ [M+H]+:429.2337. Found: 429.2338.

Substrate (+)-3d

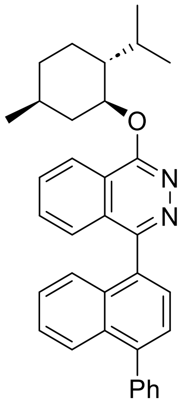

Following **GP2**, (+)-**2a** (1.0 mmol, 319 mg), (4-phenylnaphthalen-1-yl)boronic acid (1.1 mmol, 273 mg, 1.1 equiv.), Na_2CO_3 (2.0 mmol, 212 mg, 2 equiv.) and $Pd(PPh_3)_4$ (3 mol%, 34.5 mg) dissolved in 2 mL degassed toluene, 0.5 mL degassed MeOH as well as 2 mL degassed H_2O were employed. After aqueous work-up, column chromatography over silica using *n*-heptane/EtOAc (3:1 v/v) as eluent was performed and 376 mg (0.77 mmol) of an off-white solid in a yield of 77% was obtained.

^{1}H NMR (300 MHz, $CDCl_3$): δ = 8.91 (dd, *J* = 4.7,1.6 Hz, 1H), 8.33 (d, *J* = 8.1 Hz, 1H), 8.26 (d, *J* = 8.2 Hz, 1H), 8.00 (d, *J* = 8.2 Hz, 1H),7.84-7.74 (m, 2H), 7.72-7.60 (m, 2H), 7.45 (d, *J* = 8.0 Hz, 1H), 7.25 (dd, *J* = 8.8, 4.2 Hz, 1H), 5.66-5.50 (m, 1H), 2.69-2.54 (m, 1H), 2.31-2.10 (m, 1H), 1.86-1.50 (m, 4H), 1.34-0.77 (m, 13H).

$^{13}C\{^1H\}$ NMR (75 MHz, $CDCl_3$) (mixture of diastereomers): δ = 159.8, 155.8, 141.4, 140.6, 133.7, 132.8, 131.9, 131.6, 130.2, 129.3, 128.4, 127.89, 127.86, 127.5, 126.44, 126.36, 126.2, 126.1, 123.2, 120.3, 76.7, 48.01, 47.97, 40.5, 40.4, 34.7, 31.4, 26.9, 24.1, 22.2, 21.1, 21.0, 20.9, 17.2, 17.1.

$[\alpha]_D^{23}$ = +49.1 (*c* 1.0, $CHCl_3$)

HRMS (ESI): Calcd. for $C_{34}H_{34}N_2O_1$ [M+H]+: 487.2744. Found: 487.2749.

Substrate (+)-3e

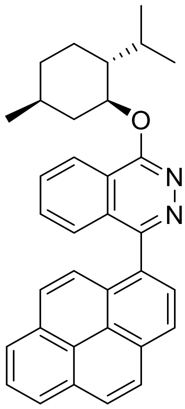

Following **GP2**, (+)-**2a** (1.0 mmol, 319 mg), pyren-1-yl boronic acid (1.1 mmol, 271 mg, 1.1 equiv.), Na_2CO_3 (2.0 mmol, 212 mg, 2 equiv.) and $Pd(PPh_3)_4$ (3 mol%, 34.5 mg) dissolved in 2 mL degassed toluene, 0.5 mL degassed MeOH as well as 2 mL degassed H_2O were brought to reaction. After aqueous work-up, column chromatography over silica using *n*-heptane/EtOAc = 3:1 as eluent was performed and 414 mg (0.86 mmol) of a pale-yellow powder in a yield of 86% was obtained.

^1H NMR (300 MHz, $CDCl_3$): δ = 8.30 (dd, *J* = 16.5, 8.1 Hz, 2H), 8.19 (d, *J* = 7.6 Hz, 1H), 8.16-8.05 (m, 4H), 8.03-7.86 (m, 2H), 7.84-7.70 (m, 2H), 7.60 (t, *J* = 7.9 Hz, 1H), 7.38 (d, *J* = 8.2 Hz, 1H), 5.66-5.50 (m, 1H), 2.70-2.56 (m, 1H), 2.31-2.07 (m, 1H), 1.86-1.50 (m, 6H), 1.34-0.77 (m, 18H).

$^{13}C\{^1H\}$ NMR (75 MHz, $CDCl_3$) (mixture of diastereomers): δ = 160.0, 156.2, 132.1, 131.9, 131.8, 131.5, 131.0, 130.2, 129.6, 128.45, 128.38, 128.2, 128.0, 127.6, 126.7, 126.3, 125.6, 125.5, 125.4, 124.9, 124.8, 124.7, 123.4, 120.5, 120.5, 71.7, 48.10, 47.98, 40.6, 40.5, 34.8, 31.5, 31.5, 27.0, 24.2, 22.3, 21.12, 21.06, 17.3, 17.2.

$[\alpha]_D^{23}$ = +61.2 (*c* 1.0, $CHCl_3$)

HRMS (ESI): Calcd. for $C_{34}H_{32}N_2O_1$ [M+H]+: 485.2587. Found: 485.2589.

Substrate (+)-3f

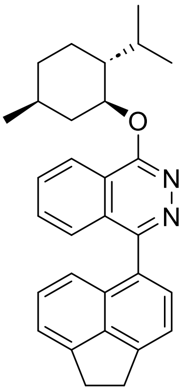

Following **GP2**, (+)-**2a** (1.0 mmol, 319 mg), (1,2-dihydroacenaphthylen-5-yl)boronic acid (1.1 mmol, 218 mg, 1.1 equiv.), Na_2CO_3 (2.0 mmol, 212 mg, 2 equiv.) and $Pd(PPh_3)_4$ (3 mol%, 34.5 mg) dissolved in 2 mL degassed toluene, 0.5 mL degassed MeOH as well as 2 mL degassed H_2O were subjected to reaction. After aqueous work-up, column chromatography over silica using *n*-heptane/EtOAc (3:1 v/v) as eluent was performed and 336.2 mg (0.77 mmol) of an off-white solid in a yield of 77% was obtained.

^{1}H NMR (300 MHz, $CDCl_3$): δ = 8.33 (d, *J* = 8.1 Hz, 1H), 7.80 (ddd, *J* = 8.3, 6.8, 1.3 Hz, 1H), 7.68-7.56 (m, 3H), 7.42 (d, *J* = 7.0 Hz, 1H), 7.38-7.27 (m, 3H), 5.69-5.52 (m, 1H), 3.48 (s, 4H), 2.71-2.59 (m, 1H), 2.31-2.14 (m, 1H), 1.88-1.55 (m, 5H), 1.35-0.778 (m, 16H).

^{13}C{^{1}H} NMR (75 MHz, $CDCl_3$) (mixture of diastereomers): δ = 159.7, 155.5, 147.5, 146.2, 139.5, 131.7, 131.5, 130.9, 130.4, 129.6, 129.2, 128.3, 126.7, 123.3, 121.3, 120.5, 119.6, 119.0, 76.8, 48.0, 34.7, 31.5, 30.6, 30.4, 27.0, 24.1, 22.3, 21.0, 17.2.

$[\alpha]_D^{23}$ = +96.7 (*c* 1.0, $CHCl_3$)

HRMS (ESI): Calcd. for $C_{30}H_{32}N_2O_1$ [M+H]+: 437.2587. Found: 437.2592.

Substrate (+)-3g

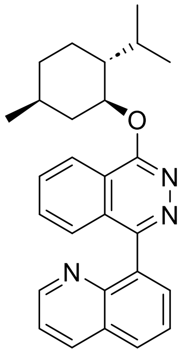

Following **GP2**, (+)-**2a** (1.0 mmol, 319 mg), (8-quinolin-4-yl)boronic acid (1.1 mmol, 190 mg, 1.1 equiv.), Na_2CO_3 (2.0 mmol, 212 mg, 2 equiv.) and $Pd(PPh_3)_4$ (3 mol%, 34.5 mg) dissolved in 2 mL degassed toluene, 0.5 mL degassed MeOH as well as 2 mL degassed H_2O were subjected to reaction. After aqueous work-up, column chromatography over silica using

n-heptane/EtOAc (3:1 v/v) as eluent was performed and 259 mg (0.63 mmol) of an off-white solid in a yield of 63% was obtained.

^{1}H NMR (300 MHz, $CDCl_3$): δ = 8.34 (d, *J* = 8.2 Hz, 1H), 8.25 (dd, *J* = 7.5, 1.2 Hz, 1H), 8.21-8.11 (m 1H), 7.88-7.75 (m, 3H), 7.72-7.64 (m, 1H), 7.58 (t, *J* = 8.3 Hz, 1H), 7.47-7.37 (m, 3H), 5.78-5.58 (m, 1H), 2.74-2.58 (m, 1H), 2.31-2.10 (m, 1H), 1.95-1.55 (m, 5H), 1.34-0.77 (m, 15H).

^{13}C{^{1}H} NMR (75 MHz, $CDCl_3$) (mixture of diastereomers): δ = 159.9, 154.4, 150.7, 150.5, 148.4, 136.1, 134.5, 134.3, 132.2, 131.9, 130.7, 129.5, 129.1, 128.9, 128.8, 127.9, 127.8, 126.6, 126.0, 123.5, 121.5, 121.1, 120.4, 48.0, 47.9, 40.5, 40.3, 34.6, 31.4, 26.9, 24.1, 22.2, 20.9, 17.2, 17.1.

$[\alpha]_D^{23}$ = +71.8 (*c* 1.0, $CHCl_3$)

HRMS (ESI): Calcd. for C27H29N3O [M+H]+: 412.2383. Found: 412.2383.

Substrate (+)-3h

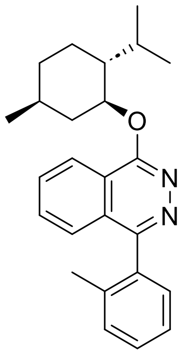

Following **GP2**, (+)-**2a** (1.0 mmol, 319 mg), (2-methylphenyl-1-yl)boronic acid (1.1 mmol, 150 mg, 1.1 equiv.), Na_2CO_3 (2.0 mmol, 212 mg, 2 equiv.) and $Pd(PPh_3)_4$ (3 mol%, 34.5 mg) dissolved in 2 mL degassed toluene, 0.5 mL degassed MeOH as well as 2 mL degassed H_2O were subjected to reaction. After aqueous work-up, column chromatography over silica using *n*-heptane/EtOAc (3:1 v/v) as eluent was performed and 260 mg (0.69 mmol) of an off-white solid in a yield of 69% was obtained.

^{1}H NMR (300 MHz, $CDCl_3$): δ = 8.30 (d, *J* = 7.9 Hz, 1H), 7.80 (ddd, *J* = 8.2, 7.1, 1.3 Hz, 1H), 7.84 (ddd, *J* = 8.2, 7.1, 1.3 Hz, 1H), 7.50 (t, *J* = 8.2 Hz, 1H), 7.43-7.27 (m, 4H), 5.66-5.46 (m, 1H), 2.72-2.47 (m, 1H), 2.31-2.18 (m, 1H), 2.16 (s, 3H), 2.06-1.52 (m, 5H), 1.45-0.77 (m, 15H).

^{13}C{^{1}H} NMR (75 MHz, $CDCl_3$) (mixture of diastereomers): δ = 159.6, 156.5, 137.1, 136.1, 131.9, 131.5, 130.4, 130.3, 128.8, 128.6, 126.1, 125.6, 123.3, 120.3, 77.4, 76.6, 71.5, 48.0, 40.5, 40.3, 34.7, 31.4, 26.9, 24.1, 22.2, 20.9, 20.1, 17.1.

$[\alpha]_D^{23}$ = +82.0 (*c* 1.0, $CHCl_3$)

HRMS (ESI): Calcd. for $C_{25}H_{30}N_2O_1$ [M+H]+: 375.2431. Found:375.2433.

Substrate (+)-3i

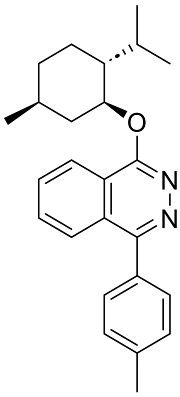

Following **GP2**, (+)-**2a** (1.0 mmol, 319 mg), (4-methylphenyl-1-yl)boronic acid (1.1 mmol, 150 mg, 1.1 equiv.), Na_2CO_3 (2.0 mmol, 212 mg, 2 equiv.) and $Pd(PPh_3)_4$ (3 mol%, 34.5 mg) dissolved in 2 mL degassed toluene, 0.5 mL degassed MeOH as well as 2 mL degassed H_2O were subjected to reaction. After aqueous work-up, column chromatography over silica using *n*-heptane/EtOAc (3:1 v/v) as eluent was performed and 341 mg (0.80 mmol) of an off-white solid in a yield of 80% was obtained.

^{1}H NMR (300 MHz, $CDCl_3$): δ = 8.28-8.21 (m, 1H), 7.98-7.91 (m, 1H) 7.78-7.65 (m, 2H), 7.60 (d, *J* = 8.8 Hz, 1H), 7.29 (d, *J* = 7.9 Hz, 1H), 5.61-5.46 (m, 1H), 2.63-2.48 (m, 1H), 2.40 (s, 3H), 2.25-2.10 (m, 1H), 1.82-1.51 (m, 5H), 1.28-0.77 (m, 14H).

^{13}C{^{1}H} NMR (75 MHz, $CDCl_3$) (mixture of diastereomers): δ = 159.4, 155.9, 138.7, 133.8, 131.8, 131.4, 129.9, 129.1, 127.9, 126.2, 123.4, 120.7, 76.7, 47.9, 40.4, 34.6, 31.4, 26.9, 24.1, 22.2, 21.4, 20.9, 17.1.

HRMS (ESI): Calcd. for $C_{25}H_{30}N_2O_1$ [M+H]+: 375.2431. Found: 375.2433.

Substrate (+)-3j

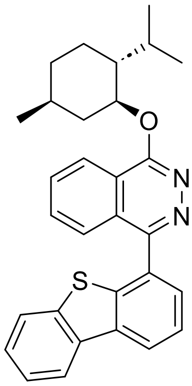

Following a modified **GP2**, (+)-**2a** (1.0 mmol, 319 mg), 4-dibenzothiophene boronic acid (1.1 mmol, 251 mg, 1.1 equiv.), Na_2CO_3 (2.0 mmol, 212 mg, 2 equiv.) and $Pd(PPh_3)_4$ (3 mol%, 34.5 mg) dissolved in 4 mL degassed THF as well as 2 mL degassed H_2O were subjected to reaction. After aqueous work-up, column chromatography over silica using

n-heptane/EtOAc (3:1 v/v) as eluent was performed and 364 mg (0.78 mmol) of an off-white solid in a yield of 78% was obtained.

^{1}H NMR (300 MHz, $CDCl_3$): δ = 8.33 (d, *J* = 8.2 Hz, 1H), 8.25 (dd, *J* = 7.7, 0.9 Hz, 1H), 8.22-8.14 (m, 1H), 7.89-7.78 8m, 3H), 7.73-7.65 (m, 2H), 7.59 (t, *J* = 7.7 Hz, 1H), 7.48-7.39 (m, 2H), 5.78-5.61 (m, 1H), 2.71-2.60 (m, 1H), 2.31-2.10 (m, 1H), 1.86-1.53 (m, 5H), 1.43-0.77 (m, 16H).

^{13}C{^{1}H} NMR (75 MHz, $CDCl_3$) (mixture of diastereomers): δ = 159.8, 154.7, 140.2, 140.1, 136.6, 135.2, 131.8, 131.7, 131.2, 128.4, 127.4, 126.9, 125.8, 124.3, 123.4, 122.6, 121.9, 121.6, 120.6, 76.8, 47.9, 40.3, 34.6, 31.3, 26.9, 24.0, 22.2, 20.9, 17.1.

$[\alpha]_D^{23}$ = +60.5 (*c* 1.0, $CHCl_3$)

HRMS (ESI): Calcd. for C30H30N2OS [M+H]+: 467.2152. Found: 4267.2154.

Substrate (+)-3k

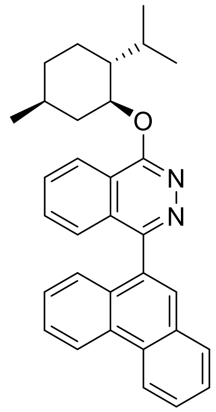

Following **GP2**, (+)-**2a** (1.0 mmol, 319 mg), phenanthrene-9-ylboronic acid (1.1 mmol, 244 mg, 1.1 equiv.), Na_2CO_3 (2.0 mmol, 212 mg, 2 equiv.) and $Pd(PPh_3)_4$ (3 mol%, 34.5 mg) dissolved in 2 mL degassed toluene, 0.5 mL degassed MeOH as well as 2 mL degassed H_2O were reacted. After aqueous work-up, column chromatography over silica using *n*-heptane/EtOAc (3:1 v/v) as eluent was performed and 377 mg (0.82 mmol) of a yellow solid in a yield of 82% was obtained.

^{1}H NMR (300 MHz, $CDCl_3$): δ = 8.83 (t, *J* = 7.7 Hz, 2H), 8.35 (d, *J* = 7.9 Hz, 1H), 7.97-7.90 (m, 2H), 7.82 (ddd, *J* = 8.2, 7.0, 1.2 Hz, 1H), 7.74 (ddd, *J* = 8.3, 7.0, 1.4 Hz, 1H), 7.70-7.53 (m, 4H), 7.51-7.4 (m, 2H),), 5.67-5.55 (m, 1H), 2.69-2.54 (m, 1H), 2.31-2.10 (m, 1H), 1.86-1.50 (m, 8H), 1.34-0.77 (m, 18H).

^{13}C{^{1}H} NMR (75 MHz, $CDCl_3$) (mixture of diastereomers): δ = 160.0, 155.84, 155.82, 133.0, 132.0, 131.8, 131.52, 131.47, 131.4, 130.8, 130.6, 129.61, 129.59, 129.5, 129.4, 129.2, 127.4, 127.1, 126.8, 126.5, 123.3, 123.0, 122.8, 120.40, 120.37, 71.6, 48.1, 48.0, 40.6, 40.4, 34.7, 31.7, 31.5, 27.0, 24.2, 22.3, 21.01, 20.98, 17.3, 17.2.

$[\alpha]_D^{23}$ = +61.2 (*c* 1.0, $CHCl_3$)

HRMS (ESI): Calcd. for $C_{32}H_{32}N_2O_1$ [M+H]+: 461.2587. Found: 461.2586.

Substrate (-)-3a

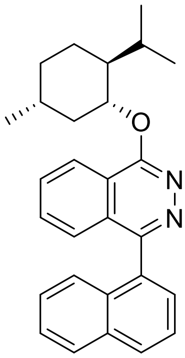

Following **GP2**, **(-)-2a** (5.0 mmol, 1.60 g), naphthalene-1-yl boronic acid (6 mmol, 0.98 g, 1.1 equiv.), Na_2CO_3 (10.0 mmol, 1.06 g, 2 equiv.) and $Pd(PPh_3)_4$ (3 mol%, 173 mg) dissolved in 10 mL degassed toluene, 2.5 mL degassed MeOH as well as 10 mL degassed H_2O were employed. After aqueous work-up, column chromatography over silica using *n*-heptane/EtOAc (3:1 v/v) as eluent was performed and 1.80 g (4.40 mmol) of a tan solid in a yield of 88% was obtained.

^{1}H NMR (300 MHz, $CDCl_3$): δ = 8.34 (d, *J* = 8.0 Hz, 1H), 8.02 (dd, *J* = 6.0, 3.5 Hz, 1H), 7.95 (d, *J* = 8.1 Hz, 1H), 7.86 (t, *J* = 7.3 Hz, 1H), 7.69-7.59 (m, 3H), 7.55-7.41 (m, 3H), 7.35 (ddd, *J* = 8.3, 7.0, 1.3 Hz, H), 5.66-5.50 (m, 1H), 2.71-2.60 (m, 1H), 2.34-2.12 (m, 1H), 1.88-1.52 (m, 5H), 1.34-0.77 (m, 18H).

^{13}C{^{1}H} NMR (75 MHz, $CDCl_3$) (mixture of diastereomers): δ = 160.0, 155.8, 133.8, 132.6, 132.0, 131.8, 129.4, 128.5, 126.6, 126.5, 126.2, 126.1, 125.4, 123.3, 120.4, 48.1, 40.6, 40.5, 34.7, 31.5, 27.0, 24.2, 22.3, 21.1, 17.3, 17.2.

$[\alpha]_D^{23}$ = -75.2 (*c* 1.0, $CHCl_3$)

HRMS (ESI): Calcd. for $C_{28}H_{30}$ N_2O_1 [M+H]+: 411.2428. Found: 411.2431.

Substrate (-)-3b

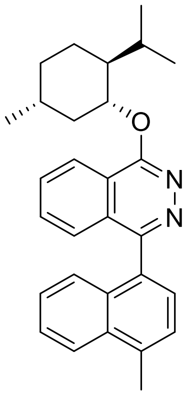

Following **GP2**, (-)-**2a** (1.0 mmol, 319 mg), (4-methylnaphthalen-1-yl) boronic acid (1.1 mmol, 205 mg, 1.1 equiv.), Na_2CO_3 (2.0 mmol, 212 mg, 2 equiv.) and $Pd(PPh_3)_4$ (3 mol%, 34.5 mg.) dissolved in 2 mL degassed toluene, 0.5 mL degassed MeOH as well as 2 mL degassed H_2O were reacted. After aqueous work-up, column chromatography over silica using *n*-heptane/EtOAc (3:1 v/v) as eluent was performed and 341 mg (0.80 mmol) of a yellow solid in a yield of 80% was obtained.

^{1}H NMR (300 MHz, $CDCl_3$): δ = 8.22 (d, *J* = 8.1 Hz, 1H), 8.10 (dd, *J* = 9.0, 1.2 Hz, 1H), 7.84 (t, *J* = 7.5 Hz, 1H), 7.63 (t, *J* = 7.9 Hz, 1H), 7.57-7.42 (m, 5H), 7.35 (t, *J* = 7.5 Hz, 1H), 5.66-5.50 (m, 1H), 2.81 (s, 3H), 2.69-2.54 (m, 1H), 2.31-2.10 (m, 1H), 1.86-1.50 (m, 5H), 1.34-0.77 (m, 18H).

^{13}C{^{1}H} NMR (75 MHz, $CDCl_3$) (mixture of diastereomers): δ = 159.9, 156.0, 135.2, 132.8, 132.6, 131.9, 129.5, 128.3, 126.8, 126.2, 126.1, 126.0, 124.5, 123.3, 120.5, 120.3, 48.1, 40.6, 40.4, 34.7, 31.5, 27.0, 24.2, 22.6, 21.1, 21.0, 19.9, 17.3, 17.2.

$[\alpha]_D^{23}$ = -68.6 (*c* 1.0, $CHCl_3$)

HRMS (ESI): Calcd. for $C_{29}H_{32}N_2O_1$ [M+H]+: 425.2584. Found: 425.2586.

Substrate (-)-3e

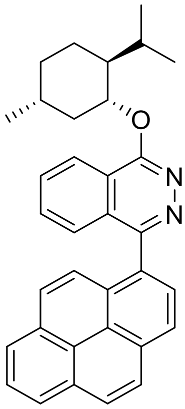

Following **GP2**, (-)-**2a** (1.0 mmol, 319 mg), pyren-1-ylboronic acid (1.1 mmol, 271 mg, 1.1 equiv.), Na_2CO_3 (2.0 mmol, 212 mg, 2 equiv.) and $Pd(PPh_3)_4$ (3 mol%, 34.5 mg.) dissolved in 2 mL degassed toluene, 0.5 mL degassed MeOH as well as 2 mL degassed H_2O were reacted. After aqueous work-up, column chromatography over silica using *n*-heptane/EtOAc (3:1 v/v) as eluent was performed and 328 mg (0.77 mmol) of a pale-yellow- powder in a yield of 77% was obtained.

^{1}H NMR (300 MHz, $CDCl_3$): δ = 8.30 (dd, *J* = 16.5, 8.1 Hz, 2H), 8.19 (d, *J* = 7.6 Hz, 1H), 8.16-8.05 (m, 4H), 8.03-7.86 (m, 2H), 7.84-7.70 (m, 2H), 7.60 (t, *J* = 7.9 Hz, 1H), 7.38 (d, *J* = 8.2 Hz, 1H), 5.66-5.50 (m, 1H), 2.70-2.56 (m, 1H), 2.31-2.07 (m, 1H), 1.86-1.50 (m, 6H), 1.34-0.77 (m, 17H).

$^{13}C\{^{1}H\}$ **NMR** (75 MHz, $CDCl_3$) (mixture of diastereomers): δ = 160.1, 156.2, 135.8, 132.1, 131.9, 131.5, 131.0, 130.2, 129.6, 128.4, 128.6, 128.0, 127.6, 126.8, 126.3, 125.7, 125.5, 125.4, 124.7, 126.5, 120.6, 48.1, 40.6, 40.5, 34.8, 31.5, 27.1, 24.2, 22.3, 21.1, 21.0, 17.3.

$[\alpha]_D^{23}$ = -64.8 (*c* 1.0, $CHCl_3$)

HRMS (ESI): Calcd. for $C_{34}H_{32}N_2O_1$ [M+H]+: 485.2587. Found: 485.2584.

3. Screening of Conditions for Iridium-catalyzed C-H Borylation of Biaryl Substrates

Table S1. Optimization of reaction conditions with substrate **3a** and **DB1**.

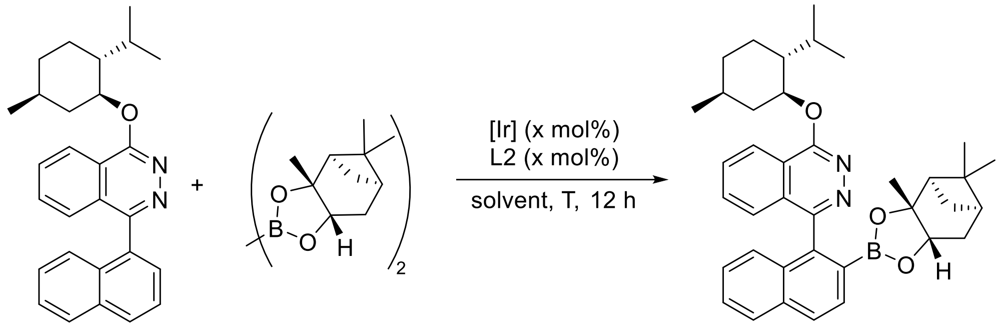

Entry	[Ir] (mol%)	Ligand (mol%)	Equiv. **DB1**	Solvent	T [°C]	t [h]	Yield **4a** [%][a]
1	$[Ir(OMe)(COD)]_2$ (1.5)	2-aminopyridine (3)	1	THF (non-dry.)	80	12	15
2	$[Ir(OMe)(COD)]_2$ (1.5)	2-aminopyridine (3)	1	abs. THF	80	12	88
3	$[Ir(OMe)(COD)]_2$ (1.5)	2-aminopyridine (3)	1	2-methyl-THF	80	12	72
4	$[Ir(OMe)(COD)]_2$ (1.5)	2-aminopyridine (3)	1	1,4-dioxane	80	12	74
5	$[Ir(OMe)(COD)]_2$ (1.5)	2-aminopyridine (3)	1	DCM	80	12	80
6	$[Ir(OMe)(COD)]_2$ (1.5)	2-aminopyridine (3)	1	DCE	80	12	-
7	$[Ir(OMe)(COD)]_2$ (1.5)	2-aminopyridine (3)	1	*n*-hexane	80	12	70
8	$[Ir(OMe)(COD)]_2$ (1.5)	2-aminopyridine (3)	1	Toluene	80	12	73
9	$[Ir(OH)(COD)]_2$ (1.5)	2-aminopyridine (3)	1	abs. THF	80	12	69
10	$[IrCl(COD)]_2$ (1.5)	2-aminopyridine (3)	1	abs. THF	80	12	62
11	$[Ir(OMe)(COD)]_2$ (1.5)	-	1	abs. THF	80	12	50
12	$[IrCl(COD)]_2$ (1.5)	-	1	abs. THF	80	12	0
13	$[Ir(OH)(COD)]_2$ (1.5)	-	1	abs. THF	80	12	0
14	$[Ir(OMe)(COD)]_2$ (1.5)	2-aminopyridine (3)	1.5	abs. THF	80	12	90
15	$[Ir(OMe)(COD)]_2$ (1.5)	2-aminopyridine (3)	2	abs. THF	80	12	94
16	$[Ir(OMe)(COD)]_2$ (1.5)	2-aminopyridine (3)	1	abs. THF	60	12	60
17	$[Ir(OMe)(COD)]_2$ (1.5)	2-aminopyridine (3)	1	abs. THF	100	12	89
18	$[Ir(OMe)(COD)]_2$ (0.15)	2-aminopyridine (0.3)	1	abs. THF	80	12	62
19	$[Ir(OMe)(COD)]_2$ (0.5)	2-aminopyridine (1)	1	abs. THF	80	12	73
20	$[Ir(OMe)(COD)]_2$ (1.0)	2-aminopyridine (2)	1	abs. THF	80	12	80

[a] Yield of isolated product.

Table S2. Ligand screening with substrate **3a** and **DB1**.

Entry	Ligand	Yield **4a** [%][a]
1	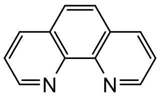	(94)[b]
2	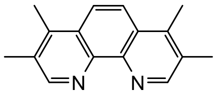	(67)[b]
3	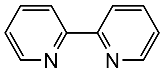	(68)[b]
4	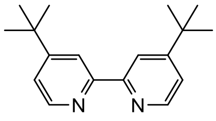	(79)[b]
5	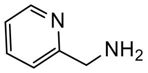	n.r. [c]
6	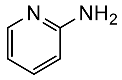	88
7	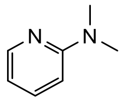	74
9	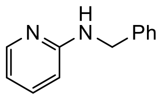	87
10	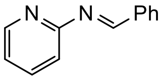	68
12	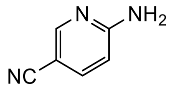	72
13	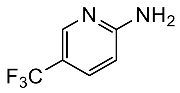	72
14	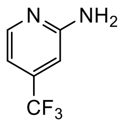	68

[a] Yield of isolated product.[b] Complex mixture of products, conversion is stated in parathesis. [c] No reaction.

4. Syntheses of Chiral Borylated Heterobiaryls

4.1. C-H Borylation with Chiral Diborons

General Procedure for Ir-catalyzed borylation (GP3)

In a glovebox with argon atmosphere, a 10 mL Schlenk flask was charged with heterobiaryl compound (0.1 mmol), diboronic ester (1 eq.), $[Ir(OMe)(COD)]_2$ (1.5 mol%, 1.0 mg) and 0.6 mL of anhydrous and degassed THF, unless stated otherwise. After stirring overnight at 80 °C, the mixture was quenched by exposure to air. Purification was achieved via column chromatography over silica using *n*-heptane/EtOAc.

Complete overview of the reactions:

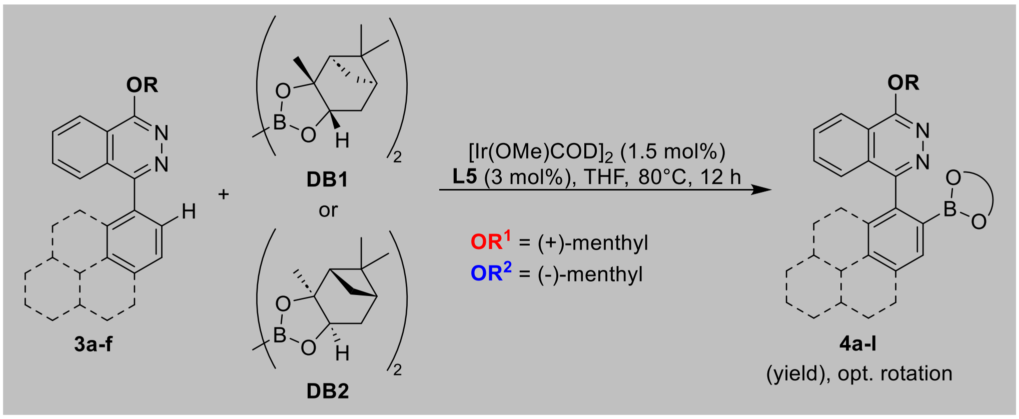

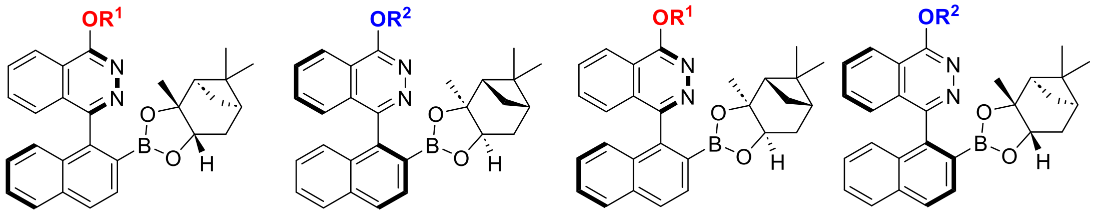

4a (88%), $[\alpha]_D^{23}$ = -4.6 **4b** (85%), +2.2 **4c** (82%), +72.8 **4d** (81%), -67.9

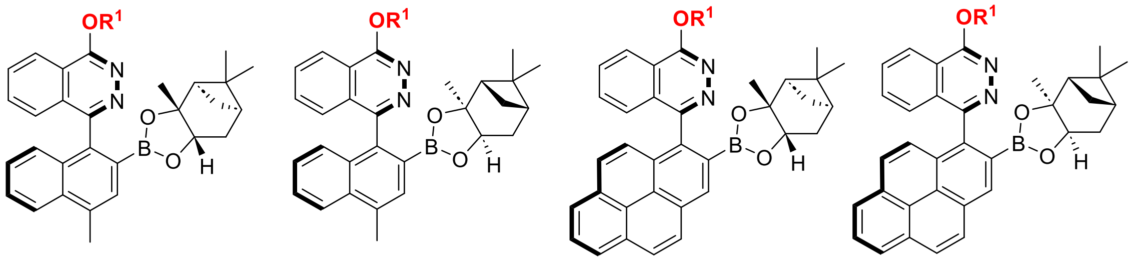

4e (70%), -3.1 **4f** (70%), +58.2 **4g** (81%), +5.7 **4h** (78%), +70.3

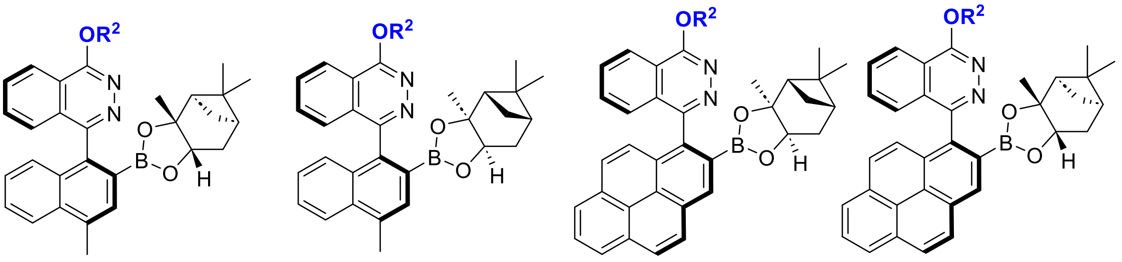

4i-SI (70%), -57.0 **4j-SI** (71%), +3.5 **4k-SI** (78%), -4.5 **4l-SI** (73%), -68.4

Compound 4a

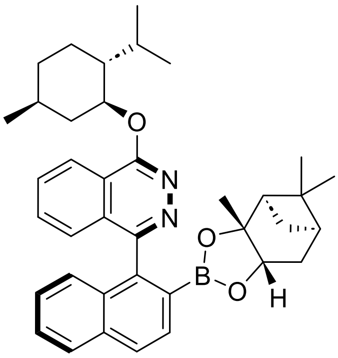

Following the general procedure **GP3**, heterobiaryl (+)-**3a** and diboronic ester **DB1** were employed. The isolation was performed via column chromatography over silica using *n*-heptane/EtOAc (1:1 v/v) as eluent and yielded 51.7 mg (0.088 mmol, 88%) as a yellow solid.

^{1}H NMR (300 MHz, $CDCl_3$): δ = 8.29 (d, *J* = 8.4 Hz, 1H), 7.99 (s, 2H), 7.93 (d, *J* = 8.0 Hz, 1H), 7.80-7.69 (m, 1H), 7.58-7.29 (m, 4H), 7.22 (t, *J* = 7.5 Hz, 1H), 5.67-5.52 (m, 1H), 4.05 (d, *J* = 8.5 Hz, 1H), 2.74-2.58 (m, 1H), 2.36-1.90 (m, 3H), 1.88-1.52 (m, 6H),), 1.22-1.08 (m, 6H), 1.08-0.84 (m, 13H), 0.69 (s, 3H).

^{13}C{^{1}H} NMR (75 MHz, $CDCl_3$): δ = 159.7, 156.8, 141.7, 135.0, 134.8, 132.5, 131.9, 131.0, 130.7, 128.3, 128.0, 127.0, 126.3, 126.0, 122.9, 120.1, 85.9, 78.0, 51.1, 48.0, 40.6, 40.3, 39.4, 37.9, 35.2, 34.7, 31.7, 28.4, 28.0, 27.1, 26.3, 26.1, 25.9, 24.0, 22.3, 20.9, 17.5, 16.2.

$[\alpha]_D^{23}$ = -4.6 (*c* 1.0, $CHCl_3$)

HRMS (EI): Calcd. for $C_{38}H_{45}N_2O_3B_1$ [M+H]+: 589.3603. Found: 589.3603.

Compound 4b

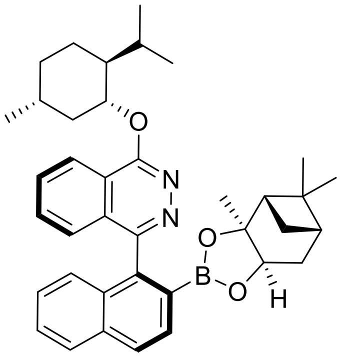

Following the general procedure **GP3**, heterobiaryl (-)-**3a** and diboronic ester **DB2** were employed. The isolation was performed via column chromatography over silica using *n*-heptane/EtOAc (1:1 v/v) as eluent and yielded 50.2 mg (0.085 mmol, 85%) as a yellow solid.

^{1}H NMR (300 MHz, $CDCl_3$): δ = 8.29 (d, *J* = 8.4 Hz, 1H), 7.99 (s, 2H), 7.93 (d, *J* = 8.0 Hz, 1H), 7.80-7.69 (m, 1H), 7.58-7.29 (m, 4H), 7.22 (t, *J* = 7.5 Hz, 1H), 5.67-5.52 (m, 1H), 4.05 (d, *J* = 8.5 Hz, 1H), 2.74-2.58 (m, 1H), 2.36-1.90 (m, 3H), 1.88-1.52 (m, 8H), 1.22-1.08 (m, 7H), 1.08-0.84 (m, 13H), 0.69 (s, 3H).

^{13}C{^{1}H} NMR (75 MHz, $CDCl_3$): δ = 159.7, 156.8, 135.0, 134.8, 132.5, 131.9, 131.0, 130.7, 128.3, 128.0, 127.0, 126.3, 126.0, 122.9, 120.1, 85.9, 78.0, 71.6, 51.1, 48.0, 40.6, 40.3, 39.4, 37.9, 35.2, 34.7, 31.7, 28.4, 28.0, 27.1, 26.3, 26.1, 25.9, 24.0, 22.3, 20.9, 17.5, 17.2.

$[\alpha]_D^{23}$ = +2.2 (*c* 1.0, $CHCl_3$)

HRMS (EI): Calcd. for $C_{38}H_{45}N_2O_3B_1$ [M+H]+: 589.3603. Found: 589.3606.

Compound 4c

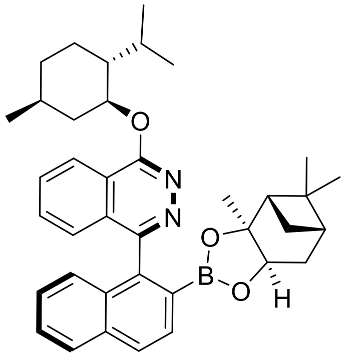

Following the general procedure **GP3**, heterobiaryl (+)-**3a** and diboronic ester **DB2** were employed. The isolation was performed via column chromatography over silica using *n*-heptane/EtOAc (1:1 v/v) as eluent and yielded 48.1 mg (0.082 mmol, 82%) as a yellow solid.

^{1}H NMR (300 MHz, $CDCl_3$): δ = 8.29 (d, *J* = 8.4 Hz, 1H), 7.99 (s, 2H), 7.93 (d, *J* = 8.0 Hz, 1H), 7.80-7.69 (m, 1H), 7.58-7.29 (m, 4H), 7.22 (t, *J* = 7.5 Hz, 1H), 5.67-5.52 (m, 1H), 4.05 (d, *J* = 8.5 Hz, 1H), 2.74-2.58 (m, 1H), 2.36-1.90 (m, 3H), 1.88-1.52 (m, 8H), 1.27 (s, 3H), 1.22-1.08 (m, 6H), 1.08-0.84 (m, 16H), 0.69 (s, 3H).

^{13}C{^{1}H} NMR (75 MHz, $CDCl_3$): δ = 159.7, 156.8, 135.0, 134.8, 132.5, 131.9, 131.0, 130.7, 128.3, 128.0, 127.0, 126.3, 126.0, 122.9, 120.1, 85.9, 78.0, 71.6, 51.1, 48.0, 40.6, 40.3, 39.4, 37.9, 35.2, 34.7, 31.7, 28.4, 28.0, 27.1, 26.3, 26.1, 25.9, 24.0, 22.3, 20.9, 17.5, 16.2.

^{11}B NMR (96 MHz, $CDCl_3$): δ = 30.20 (br, s).

$[\alpha]_D^{23}$ = +72.8 (*c* 1.0, $CHCl_3$)

HRMS (EI): Calcd. for $C_{38}H_{45}N_2O_3B_1$ [M+H]+: 589.3603. Found: 589.3606.

Compound 4d

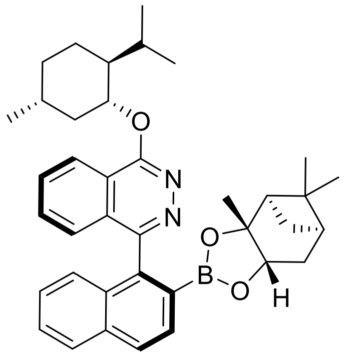

Following the general procedure **GP3**, heterobiaryl (-)-**3a** and diboronic ester **DB1** were employed. The isolation was performed via column chromatography over silica using *n*-heptane/EtOAc (1:1 v/v) as eluent and yielded 47.7 mg (0.081 mmol, 81%) as a yellow solid.

^{1}H NMR (300 MHz, $CDCl_3$): δ = 8.29 (d, *J* = 8.4 Hz, 1H), 7.99 (s, 2H), 7.93 (d, *J* = 8.0 Hz, 1H), 7.80-7.69 (m, 1H), 7.58-7.29 (m, 4H), 7.22 (t, *J* = 7.5 Hz, 1H), 5.67-5.52 (m, 1H), 4.05 (d, *J* = 8.5 Hz, 1H), 2.74-2.58 (m, 1H), 2.36-1.90 (m, 3H), 1.88-1.52 (m, 8H), 1.27 (s, 3H), 1.22-1.08 (m, 6H), 1.08-0.84 (m, 16H), 0.69 (s, 3H).

^{13}C{^{1}H} NMR (75 MHz, $CDCl_3$): δ = 159.7, 156.8, 135.0, 134.8, 132.5, 131.9, 131.0, 130.7, 128.3, 128.0, 127.0, 126.3, 126.0, 122.9, 120.1, 85.9, 78.0, 71.6, 51.1, 48.0, 40.6, 40.3, 39.4, 37.9, 35.2, 34.7, 31.7, 28.4, 28.0, 27.1, 26.3, 26.1, 25.9, 24.0, 22.3, 20.9, 17.5, 17.2.

$[\alpha]_D^{23}$ = -67.9 (*c* 1.0, $CHCl_3$)

HRMS (EI): Calcd. for $C_{38}H_{45}N_2O_3B_1$ [M+H]+: 589.3603. Found: 589.3607.

Compound 4e

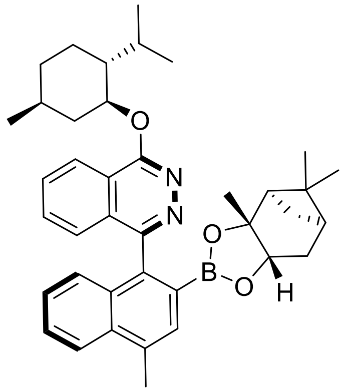

Following the general procedure **GP3**, heterobiaryl (+)-**3b** and diboronic ester **DB1** were employed. The isolation was performed via column chromatography over silica using *n*-heptane/EtOAc (1:1 v/v) as eluent and yielded 42.4 mg (0.070 mmol, 70%) as a yellow solid.

^{1}H NMR (300 MHz, $CDCl_3$): δ = 8.30 (d, *J* = 8.1 Hz, 1H), 8.08 (d, *J* = 8.2 Hz, 1H), 7.81 (s, 1H), 7.78 (t, *J* = 7.5 Hz, 1H), 7.59-7.49 (m, 3H), 7.33 (ddd, *J* = 8.3, 7.1, 1.0 Hz, 1H), 7.25-7.19 (m, 1H), 5.71-5.51 (m, 1H), 4.14 (d, *J* = 9.3 Hz, 1H), 2.80 (s, 3H), 2.70-2.56 (m, 1H), 2.36-1.95 (m, 3H), 1.86-1.50 (m, 12H), 1.34-1.10 (m, 9H) 1.08-0.88 (m, 13H), 0.69 (d, *J* = 4.0 Hz, 3H).

^{13}C{^{1}H} NMR (75 MHz, $CDCl_3$): δ = 159.9, 156.0, 140.2, 135.8, 132.8, 132.6, 131.9, 131.7, 129.5, 128.2, 126.8, 126.7, 126.2, 126.1, 125.9, 124.5, 123.3, 120.4, 87.3, 50.3, 48.1, 45.2, 40.6, 40.4, 37.8, 34.7, 31.8, 31.5, 27.0, 26.0, 24.2, 23.3, 22.4, 22.3, 21.2, 21.0, 19.9, 17.3, 16.2.

$[\alpha]_D^{23}$ = -3.1 (*c* 1.0, $CHCl_3$)

HRMS (EI): Calcd. for $C_{39}H_{47}N_2O_3B_1$ [M+H]+: 603.3759. Found: 603.3753.

Compound 4f

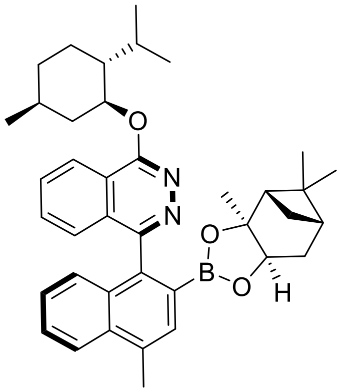

Following the general procedure **GP3**, heterobiaryl (+)-**3b** and diboronic ester **DB2** were employed. The isolation was performed via column chromatography over silica using *n*-heptane/EtOAc (1:1 v/v) as eluent and yielded 42.1 mg (0.070 mmol, 70%) as a yellow solid.

^{1}H NMR (300 MHz, $CDCl_3$): δ = 8.30 (d, *J* = 8.1 Hz, 1H), 8.08 (d, *J* = 8.2 Hz, 1H), 7.81 (s, 1H), 7.78 (t, *J* = 7.5 Hz, 1H), 7.59-7.49 (m, 3H), 7.33 (ddd, *J* = 8.3, 7.1, 1.0 Hz, 1H), 7.25-7.19 (m, 1H), 5.71-5.51 (m, 1H), 4.14 (d, *J* = 9.3 Hz, 1H), 2.80 (s, 3H), 2.70-2.56 (m, 1H), 2.36-1.95 (m, 3H), 1.86-1.50 (m, 8H), 1.34-1.10 (m, 8H) 1.08-0.88 (m, 13H), 0.69 (d, *J* = 4.0 Hz, 3H).

^{13}C{^{1}H} NMR (75 MHz, $CDCl_3$): δ = 159.9, 156.0, 135.8, 132.8, 132.6, 131.9, 131.7, 129.5, 128.2, 126.8, 126.7, 126.2, 126.1, 125.9, 124.5, 123.3, 120.4, 87.3, 71.7, 50.3, 48.1, 45.2, 40.6, 40.4, 37.8, 34.7, 31.8, 31.5, 27.0, 26.0, 24.2, 23.3, 22.4, 22.3, 21.2, 21.0, 19.9, 17.3, 16.2.

$[\alpha]_D^{23}$ = +58.2 (*c* 1.0, $CHCl_3$)

HRMS (EI): Calcd. for $C_{39}H_{47}N_2O_3B_1$ [M+H]+: 603.3759. Found: 603.3755.

Compound 4g

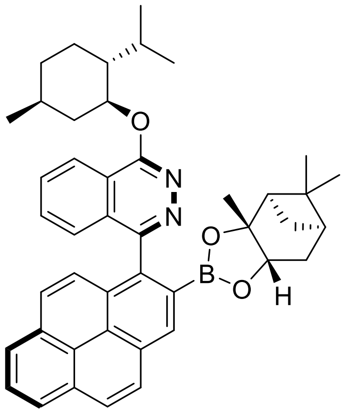

Following the general procedure **GP3**, heterobiaryl (+)-**3e** and diboronic ester **DB1** were employed. The isolation was performed via column chromatography over silica using *n*-heptane/EtOAc (1:1 v/v)- as eluent and yielded 47.8 mg (0.072 mmol, 72%) as a yellow solid.

1 mmol scale

Following **GP3**, heterobiaryl(+)-**3e** (1.00 mmol, 484.6 mg) and diboronic ester **DB1** (1 equiv., 358.0 mg), [Ir(OMe)(COD)]$_2$ (1.5 mol%, 10.0 mg) and 6.0 mL of anhydrous and degassed THF were employed. The isolation was performed via column chromatography over silica using *n*-heptane/EtOAc (1:1 v/v) as eluent and yielded 509.7 mg (0.77 mmol, 77%) as a pale-yellow solid.

^{1}H NMR (300 MHz, $CDCl_3$): δ = 8.79 (s, 1H), 8.24 (d, *J* = 8.3 Hz, 1H), 8.06 (t, *J* = 7.6 Hz, 1H), 7.92 (d, *J* = 9.6 Hz, 1H), 4.14 (d, *J* = 9.3 Hz, 1H), 2.70-2.56 (m, 1H), 2.36-1.95 (m, 3H), 1.86-1.50 (m, 12H), 1.34-1.10 (m, 9H) 1.08-0.88 (m, 13H), 0.69 (d, *J* = 4.0 Hz, 3H).

^{13}C{^{1}H} NMR (75 MHz, $CDCl_3$): δ = 159.8, 157.2, 131.9, 131.6, 131.3, 131.1, 130.9, 130.0, 127.9, 127.8, 126.6, 126.5, 126.2, 125.8, 125.4, 125.3, 124.6, 123.0, 86.1, 86.0, 71.6, 51.1, 48.1, 40.6, 40.3, 39.5, 39.3, 38.0, 35.3, 34.8, 31.5, 28.5, 28.1, 27.2, 26.4, 26.1, 25.9, 24.3, 24.2, 24.0, 22.3, 21.2, 21.0, 17.5, 16.2.

$[\alpha]_D^{23}$ = +5.7 (*c* 1.0, $CHCl_3$)

HRMS (EI): Calcd. for $C_{44}H_{47}N_2O_3B_1$ [M+H]+: 663.3760. Found: 663.3762.

Compound 4h

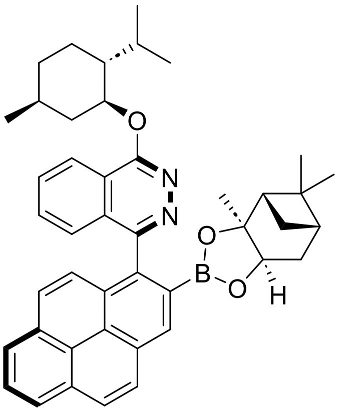

Following the general procedure **GP3**, heterobiaryl (+)-**3e** and diboronic ester **DB2** were employed. The isolation was performed via column chromatography over silica using *n*-heptane/EtOAc (1:1 v/v) as eluent and yielded 51.7 mg (0.078 mmol, 78%) as a yellow solid.

^{1}H NMR (300 MHz, $CDCl_3$): δ = 8.79 (s, 1H), 8.24 (d, *J* = 8.3 Hz, 1H), 8.06 (t, *J* = 7.6 Hz, 1H), 7.92 (d, *J* = 9.6 Hz, 1H), 4.14 (d, *J* = 9.3 Hz, 1H), 2.70-2.56 (m, 1H), 2.36-1.95 (m, 3H), 1.86-1.50 (m, 12H), 1.34-1.10 (m, 9H) 1.08-0.88 (m, 13H), 0.69 (d, *J* = 4.0 Hz, 3H).

^{13}C NMR (75 MHz, $CDCl_3$): δ = 159.8, 157.2, 131.9, 131.6, 131.3, 131.1, 130.9, 130.0, 127.9, 127.8, 126.6, 126.5, 126.2, 125.8, 125.4, 125.3, 124.6, 123.0, 86.1, 86.0, 71.6, 51.1, 48.1, 40.6, 40.3, 39.5, 39.3, 38.0, 35.3, 34.8, 31.5, 28.5, 28.1, 27.2, 26.4, 26.1, 25.9, 24.3, 24.2, 24.0, 22.3, 21.2, 21.0, 17.5, 16.2.

$[\alpha]_D^{23}$ = +70.3 (*c* 1.0, $CHCl_3$)

HRMS (EI): Calcd. for $C_{44}H_{48}N_2O_3B_1$ [M+H]+: 663.3760. Found: 663.3756.

Compound 4i-SI

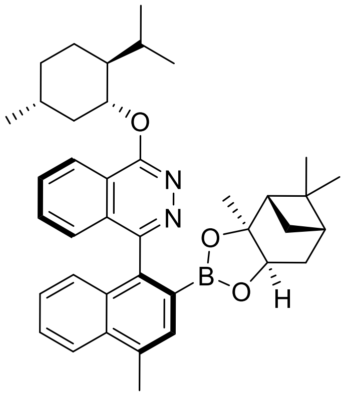

Following the general procedure **GP3**, heterobiaryl (-)-**3b** and diboronic ester **DB2** were employed. The isolation was performed via column chromatography over silica using *n*-heptane/EtOAc (1:1 v/v) as eluent and yielded 42.8 mg (0.071 mmol, 71%) as a yellow solid.

^{1}H NMR (300 MHz, $CDCl_3$): δ = 8.30 (d, *J* = 8.1 Hz, 1H), 8.08 (d, *J* = 8.2 Hz, 1H), 7.81 (s, 1H), 7.78 (t, *J* = 7.5 Hz, 1H), 7.59-7.49 (m, 3H), 7.33 (ddd, *J* = 8.3, 7.1, 1.0 Hz, 1H), 7.25-7.19 (m,

1H), 5.71-5.51 (m, 1H), 4.14 (d, J = 9.3 Hz, 1H), 2.80 (s, 3H), 2.70-2.56 (m, 1H), 2.36-1.95 (m, 3H), 1.86-1.50 (m, 12H), 1.34-1.10 (m, 9H) 1.08-0.88 (m, 13H), 0.69 (d, J = 4.0 Hz, 3H).

^{13}C{^{1}H} NMR (75 MHz, $CDCl_3$): δ = 159.9, 156.0, 135.8, 132.8, 132.6, 131.9, 131.7, 129.5, 128.2, 126.8, 126.7, 126.2, 126.1, 125.9, 124.5, 123.3, 120.4, 83.4, 76.3, 50.3, 48.1, 45.2, 40.6, 40.4, 37.8, 34.7, 31.8, 31.5, 27.0, 26.0, 24.2, 23.3, 22.4, 22.3, 21.2, 21.0, 19.9, 17.3, 16.2.

$[\alpha]_D^{23}$ = +3.5 (c 1.0, $CHCl_3$)

HRMS (EI): Calcd. for $C_{39}H_{47}N_2O_3B_1$ [M+H]+: 603.3759. Found: 603.3763.

Compound 4j-SI

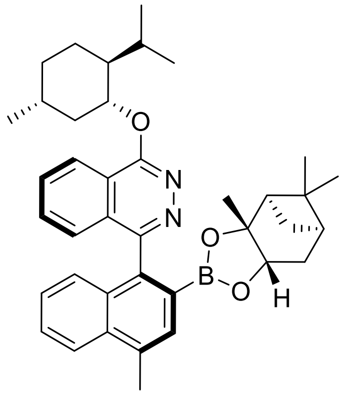

Following the general procedure **GP3**, heterobiaryl (-)-**3b** and diboronic ester **DB1** were employed. The isolation was performed via column chromatography over silica using *n*-heptane/EtOAc (1:1 v/v) as eluent and yielded 42.0 mg (0.070 mmol, 70%) as a yellow solid.

^{1}H NMR (300 MHz, $CDCl_3$): δ = 8.30 (d, J = 8.1 Hz, 1H), 8.08 (d, J = 8.2 Hz, 1H), 7.81 (s, 1H), 7.78 (t, J = 7.5 Hz, 1H), 7.59-7.49 (m, 3H), 7.33 (ddd, J = 8.3, 7.1, 1.0 Hz, 1H), 7.25-7.19 (m, 1H), 5.71-5.51 (m, 1H), 4.14 (d, J = 9.3 Hz, 1H), 2.80 (s, 3H), 2.70-2.56 (m, 1H), 2.36-1.95 (m, 3H), 1.86-1.50 (m, 12H), 1.34-1.10 (m, 9H) 1.08-0.88 (m, 13H), 0.69 (d, J = 4.0 Hz, 3H).

^{13}C{^{1}H} NMR (75 MHz, $CDCl_3$): δ = 159.9, 156.0, 135.8, 132.8, 132.6, 131.9, 131.7, 129.5, 128.2, 126.8, 126.7, 126.2, 126.1, 125.9, 124.5, 123.3, 120.4, 87.3, 71.7, 50.3, 48.1, 45.2, 40.6, 40.4, 37.8, 34.7, 31.8, 31.5, 27.0, 26.0, 24.2, 23.3, 22.4, 22.3, 21.2, 21.0, 19.9, 17.3, 16.2.

$[\alpha]_D^{23}$ = -57.0 (c 1.0, $CHCl_3$)

HRMS (EI): Calcd. for $C_{39}H_{47}N_2O_3B_1$ [M+H]+: 603.3759. Found: 603.3755.

Compound 4k-SI

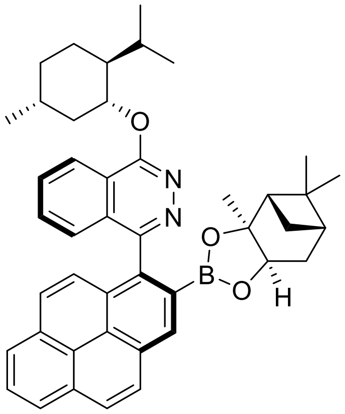

Following the general procedure **GP3**, heterobiaryl (-)-**3e** and diboronic ester **DB2** were employed. The isolation was performed via column chromatography over silica using *n*-heptane/EtOAc (1:1 v/v) as eluent and yielded 51.7 mg (0.078 mmol, 78%) as a yellow solid.

^{1}H NMR (300 MHz, $CDCl_3$): δ = 8.79 (s, 1H), 8.24 (d, *J* = 8.3 Hz, 1H), 8.06 (t, *J* = 7.6 Hz, 1H), 7.92 (d, *J* = 9.6 Hz, 1H), 4.14 (d, *J* = 9.3 Hz, 1H), 2.70-2.56 (m, 1H), 2.36-1.95 (m, 3H), 1.86-1.50 (m, 12H), 1.34-1.10 (m, 9H) 1.08-0.88 (m, 13H), 0.69 (d, *J* = 4.0 Hz, 3H).

^{13}C{^{1}H} NMR (75 MHz, $CDCl_3$): δ = 159.8, 157.2, 131.9, 131.6, 131.3, 131.1, 130.9, 130.0, 127.9, 127.8, 126.6, 126.5, 126.2, 125.8, 125.4, 125.3, 124.6, 123.0, 86.1, 86.0, 71.6, 51.1, 48.1, 40.6, 40.3, 39.5, 39.3, 38.0, 35.3, 34.8, 31.5, 28.5, 28.1, 27.2, 26.4, 26.1, 25.9, 24.3, 24.2, 24.0, 22.3, 21.2, 21.0, 17.5, 16.2.

$[\alpha]_D^{23}$ = -4.5 (*c* 1.0, $CHCl_3$)

HRMS (EI): Calcd. for $C_{44}H_{47}N_2O_3B_1$ [M+H]+: 663.3760. Found: 663.3762.

Compound 4l-SI

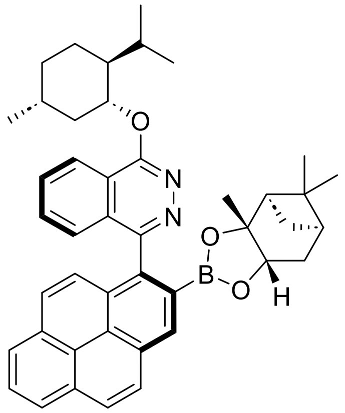

Following the general procedure **GP3**, heterobiaryl (-)-**3e** and diboronic ester **DB1** were employed. The isolation was performed via column chromatography over silica using *n*-heptane/EtOAc (1:1 v/v) as eluent and yielded 48.3 mg (0.073 mmol, 73%) as a yellow solid.

^{1}H NMR (300 MHz, $CDCl_3$): δ = 8.80 (s, 1H), 8.23 (d, *J* = 8.3 Hz, 1H), 8.06 (t, *J* = 7.5 Hz, 1H), 7.92 (d, *J* = 9.6 Hz, 1H), 4.14 (d, *J* = 9.3 Hz, 1H), 2.70-2.56 (m, 1H), 2.36-1.95 (m, 3H), 1.86-1.50 (m, 12H), 1.34-1.10 (m, 9H) 1.08-0.88 (m, 13H), 0.69 (d, *J* = 4.0 Hz, 3H).

$^{13}C\{^1H\}$ NMR (75 MHz, $CDCl_3$): δ = 159.8, 157.2, 131.9, 131.6, 131.3, 131.1, 130.9, 130.0, 127.9, 127.8, 126.6, 126.5, 126.2, 125.8, 125.4, 125.3, 124.6, 123.0, 86.1, 86.0, 71.6, 51.1, 48.1, 40.6, 40.3, 39.5, 39.3, 38.0, 35.3, 34.8, 31.5, 28.5, 28.1, 27.2, 26.4, 26.1, 25.9, 24.3, 24.2, 24.0, 22.3, 21.2, 21.0, 17.5, 16.2.

$[\boldsymbol{\alpha}]_{\boldsymbol{D}}^{\mathbf{23}}$ = -68.4 (*c* 1.0, $CHCl_3$)

HRMS (EI): Calcd. for $C_{44}H_{48}N_2O_3B_1$ [M+H]+: 663.3760. Found: 663.3757.

4.2. C-H Borylation with B₂pin₂ (DB3) and Related Diborons

Complete overview of the reactions:

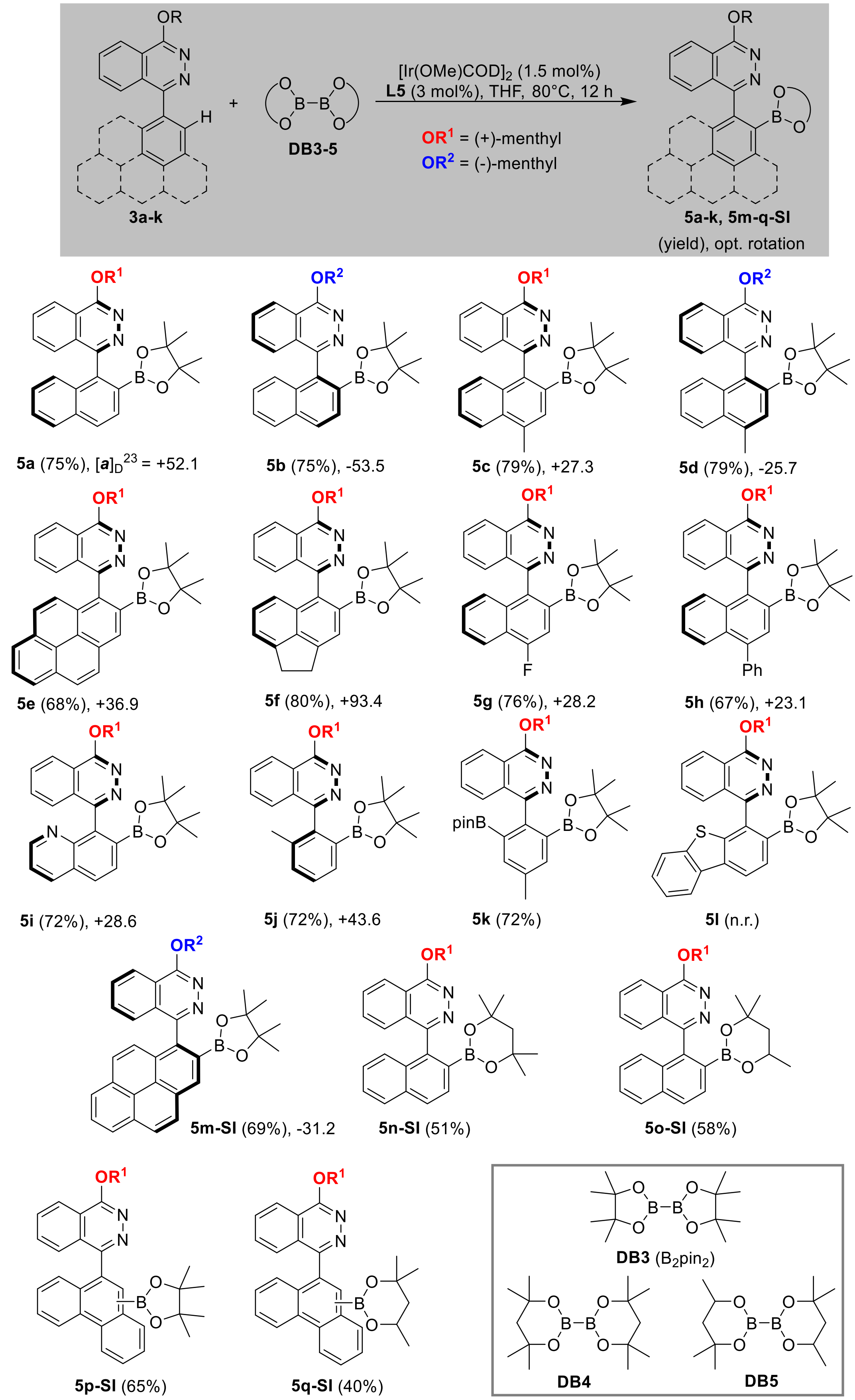

Additional experiments using diborons possessing a six-membered ring boronic ester (**DB4** and **DB5**) allowed to obtain the products still mediocre yields, but without the atroposelectivity observed before (examples **5n-SI** and **5o-SI**). On the other hand, connecting the phthalazine system to phenanthrene and subjecting the substrate to borylation with B_2pin_2 (**DB3**) under identical conditions led to position-unselective borylated products **5p-SI** and also **5q-SI**, if the diboron **DB5** was used in the latter case.

Compound 5a

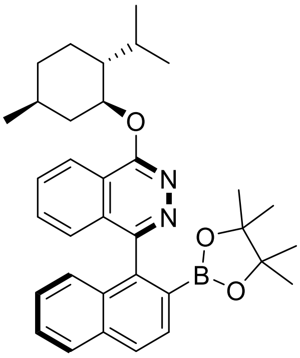

Following the general procedure **GP3**, heterobiaryl(+)-**3a** and diboronic ester **DB3** were employed. The isolation was performed via column chromatography over silica using *n*-heptane/EtOAc (1:1 v/v) as eluent and yielded 40.4 mg (0.075 mmol, 75%) as a pale-yellow solid.

1 mmol scale

Following **GP3**, heterobiaryl(+)-**3a** (1.00 mmol, 411.0 mg) and diboronic ester **DB3** (1 equiv., 254.0 mg), $[Ir(OMe)(COD)]_2$ (1.5 mol%, 10.0 mg) and 6.0 mL of anhydrous and degassed THF were employed. The isolation was performed via column chromatography over silica using *n*-heptane/EtOAc (1:1 v/v) as eluent and yielded 454.2 mg (0.85 mmol, 85%) as a pale-yellow solid.

^{1}H NMR (300 MHz, $CDCl_3$): δ = 8.30 (d, *J* = 8.1 Hz, 1H), 7.97 (s, 2H), 7.92 (d, *J* = 8.2 Hz, 1H), 7.76 (ddd, *J* = 8.1, 7.1, 1.1 Hz, 1H), 7.60-7.47 (m, 3H), 7.33 (ddd, *J* = 8.2, 7.1, 1.2 Hz, 1H), 7.25-7.19 (m, 1H), 5.73-5.51 (m, 1H), 2.74-2.57 (m, 1H), 2.36-2.11 (m, 1H), 1.88-1.60 (m, 5H), 1.42-1.15 (m, 6H), 1.09-0.83 (m, 12H), 1.34-0.84 (m, 8H), 0.79 (s, 3H).

^{13}C{^{1}H} NMR (75 MHz, $CDCl_3$): δ = 159.7, 156.8, 134.9, 132.6, 131.5, 131.1, 131.0, 130.6, 128.1, 128.0, 127.1, 127.0, 126.3, 126.2, 122.9, 120.0, 83.5, 48.1$^{\#}$, 48.0$^{\#}$, 40.7$^{\#}$, 40.3$^{\#}$, 34.7, 31.5$^{\#}$, 31.4$^{\#}$, 27.2$^{\#}$, 27.0$^{\#}$, 24.6, 24.5, 24.4, 24.3, 22.3, 21.1$^{\#}$, 21.0$^{\#}$, 17.5$^{\#}$, 17.3$^{\#}$. ($^{\#}$ = rotamers)

$[\alpha]_D^{23}$ = +52.1 (*c* 1.0, $CHCl_3$)

HRMS (EI): Calcd. for $C_{34}H_{41}N_2O_3B_1$ [M+H]+: 537.3289. Found: 537.3287.

Compound 5b

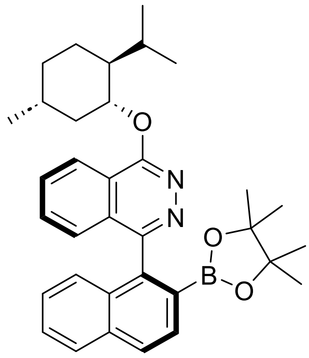

Following the general procedure **GP3**, heterobiaryl (**-**)-**3a** and diboronic ester **DB3** were employed. The isolation was performed via column chromatography over silica using *n*-heptane/EtOAc (1:1 v/v) as eluent and yielded 40.4 mg (0.075 mmol, 75%) as a yellow solid.

^{1}H NMR (300 MHz, $CDCl_3$): δ = 8.30 (d, *J* = 8.1 Hz, 1H), 7.97 (s, 2H), 7.92 (d, *J* = 8.2 Hz, 1H), 7.76 (ddd, *J* = 8.1, 7.1, 1.1 Hz, 1H), 7.60-7.47 (m, 3H), 7.33 (ddd, *J* = 8.2, 7.1, 1.2 Hz, 1H), 7.25-7.19 (m, 1H), 5.73-5.51 (m, 1H), 2.74-2.57 (m, 1H), 2.36-2.11 (m, 1H), 1.88-1.60 (m, 4H), 1.36-1.15 (m, 4H), 1.09-0.83 (m, 18H), 0.79 (s, 3H).

^{13}C{^{1}H} NMR (75 MHz, $CDCl_3$): δ = 159.6, 156.8, 134.8, 131.6, 131.5, 131.0, 130.8, 128.4, 128.0, 127.1, 126.9, 126.3, 126.2, 122.8, 120.0, 85.9, 48.1, 40.7$^{\#}$, 40.4$^{\#}$, 38.0, 34.8, 31.5, 27.1, 24.6, 24.42, 24.40, 24.0, 22.3, 21.1$^{\#}$, 21.0$^{\#}$, 17.5$^{\#}$, 17.3$^{\#}$. ($^{\#}$ = rotamers)

$[\alpha]_D^{23}$ = -53.5 (*c* 1.0, $CHCl_3$)

HRMS (EI): Calcd. for $C_{34}H_{41}N_2O_3B_1$ [M+H]+: 537.3289. Found: 537.3289.

Compound 5c

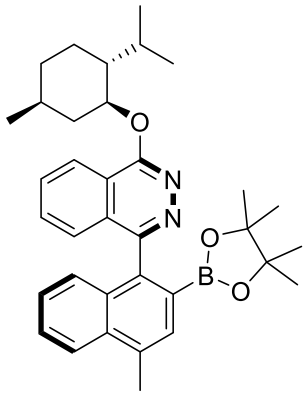

Following the general procedure **GP3**, heterobiaryl (+)-**3b** and diboronic ester **DB3** were employed. The isolation was performed via column chromatography over silica using *n*-heptane/EtOAc (1:1 v/v) as eluent and yielded 43.2 mg (0.079 mmol, 79%) as a yellow solid.

^{1}H NMR (300 MHz, $CDCl_3$): δ = 8.29 (d, *J* = 8.1 Hz, 1H), 8.09 (d, *J* = 8.2 Hz, 1H), 7.81 (s, 1H), 7.78 (t, *J* = 7.5 Hz, 1H), 7.59-7.49 (m, 3H), 7.33 (ddd, *J* = 8.3, 7.1, 1.0 Hz, 1H), 7.25-7.19 (m, 1H), 5.71-5.51 (m, 1H), 2.80 (s, 3H), 2.70-2.56 (m, 1H), 2.31-2.07 (m, 1H), 1.86-1.50 (m, 6H), 1.35-1.20 (m, 9H), 1.10-0.84 (m, 20H), 0.79 (d, *J* = 2.7 Hz, 3H).

^{13}C{^{1}H} NMR (75 MHz, $CDCl_3$): δ = 159.6, 157.0, 134.2, 134.1, 132.6, 131.4, 131.2, 131.1, 131.0, 127.7, 126.8, 126.3, 125.9, 124.2, 122.8, 120.0, 83.4, 71.6, 48.1$^{\#}$, 48.0$^{\#}$, 40.7$^{\#}$, 40.3$^{\#}$, 34.7, 31.5$^{\#}$, 31.3$^{\#}$, 27.2$^{\#}$, 27.0$^{\#}$, 24.6, 24.5, 24.4, 24.3, 22.3, 21.1$^{\#}$, 20.9$^{\#}$, 19.6, 17.5$^{\#}$, 17.3$^{\#}$. ($^{\#}$ = rotamers)

$[\alpha]_D^{23}$ = +27.3 (*c* 1.0, $CHCl_3$)

HRMS (EI): Calcd. for $C_{35}H_{43}N_2O_3B_1$ [M+H]+: 551.3446. Found: 551.3450.

Compound 5d

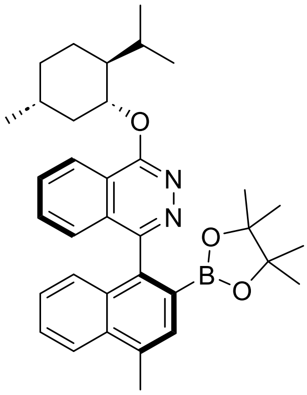

Following the general procedure **GP3**, heterobiaryl (-)-**3b** and diboronic ester **DB3** were employed. The isolation was performed via column chromatography over silica using *n*-heptane/EtOAc (1:1 v/v) as eluent and yielded 43.3 mg (0.079 mmol, 79%) as a yellow solid.

^{1}H NMR (300 MHz, $CDCl_3$): δ = 8.29 (d, *J* = 8.1 Hz, 1H), 8.09 (d, *J* = 8.2 Hz, 1H), 7.81 (s, 1H), 7.78 (t, *J* = 7.5 Hz, 1H), 7.59-7.49 (m, 3H), 7.33 (ddd, *J* = 8.3, 7.1, 1.0 Hz, 1H), 7.25-7.19 (m, 1H), 5.71-5.51 (m, 1H), 2.80 (s, 3H), 2.70-2.56 (m, 1H), 2.31-2.07 (m, 1H), 1.86-1.50 (m, 6H), 1.35-1.20 (m, 9H), 1.10-0.84 (m, 15H), 0.79 (d, *J* = 2.7 Hz, 3H).

^{13}C{^{1}H} NMR (75 MHz, $CDCl_3$): δ = 159.6, 157.0, 134.1, 132.6, 131.4, 131.2, 130.9, 127.8, 126.8, 126.3, 125.9, 124.2, 122.8, 83.4, 48.2, 40.8$^{\#}$, 40.3$^{\#}$, 34.8, 31.5$^{\#}$, 31.4$^{\#}$, 27.2$^{\#}$, 27.1$^{\#}$, 25.0, 24.7, 24.6, 24.4, 22.6, 21.1$^{\#}$, 20.9$^{\#}$, 19.6, 17.5$^{\#}$, 17.3$^{\#}$. ($^{\#}$ = rotamers)

$[\alpha]_D^{23}$ = -25.7 (*c* 1.0, $CHCl_3$)

HRMS (EI): Calcd. for $C_{35}H_{43}N_2O_3B_1$ [M+H]+: 551.3446. Found: 551.3447.

Compound 5e

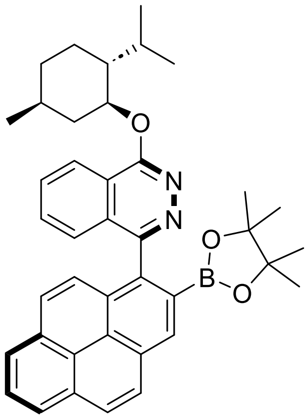

Following the general procedure **GP3**, heterobiaryl (+)-**3e** and diboronic ester **DB3** were employed. The isolation was performed via column chromatography over silica using *n*-heptane/EtOAc (1:1 v/v) as eluent and yielded 41.5 mg (0.068 mmol, 68%) as a yellow solid.

^{1}H NMR (300 MHz, $CDCl_3$): δ = 8.76 (s, 1H), 8.36 (d, *J* = 7.8 Hz, 1H), 8.23 (d, *J* = 7.6 Hz, 1H), 8.20-8.11 (m, 3H) , 8.06 (t, *J* = 7.6 Hz, 1H), 7.94 (d, *J* = 9.2 Hz, 1H), 7.83-7.72 (m, 1H), 7.61-7.50 (m, 1H), 7.24-7.12 (m, 1H), 5.75-5.54 (m, 1H), 2.70-2.56 (m, 1H), 2.31-2.07 (m, 1H), 1.86-1.50 (m, 6H), 1.35-1.20 (m, 9H), 1.10-0.84 (m, 20H), 0.79 (d, *J* = 2.7 Hz, 3H).

^{13}C{^{1}H} NMR (126 MHz, $CDCl_3$): δ = 159.8, 157.2, 131.9, 131.7, 131.4, 131.1, 130.0, 129.9, 128.0, 127.9, 126.7, 126.4, 126.2, 125.9, 125.4, 125.3, 124.6, 123.0, 83.7, 48.2$^\#$, 48.0$^\#$, 40.8$^\#$, 40.3$^\#$, 34.8, 31.5$^\#$, 31.4$^\#$, 27.2$^\#$, 27.1$^\#$, 24.7, 24.6, 24.5, 24.3, 22.3, 21.1$^\#$, 21.0$^\#$, 17.5$^\#$, 17.4$^\#$. ($^\#$ = rotamers)

^{13}C NMR, quantitative (126 MHz, $CDCl_3$ at -10°C): 159.7 (s, 1C), 157.2 (s, 1C), 136.9 (s, 1C), 131.7-131.4 (m, 2C), 131.3-131.1 (m, 3C), 130.7 (s, 1C), 129.8 (s, 1C), 128.0-127.6 (m, 3C), 126.7 (s, 1C), 126.3-125.6 (m, 3C), 125.5-125.1 (m, 2C), 124.4 (s, 1C), 122.9 (s, 1C), 199.9 (s, 1C), 83.6 (s, 2C), 47.8(d, *J* = 18 Hz, 1C), 40.2 (d, *J* = 74 Hz, 1C), 34.6, 31.4 (d, *J* = 20 Hz, 1C), 26.9 (d, *J* = 25 Hz, 1C), 24.7 (d, *J* = 7.8 Hz, 1C), 24.8-23.6 (m, 4C), 22.4 (s, 1C), 21.1 (d, *J* = 18 Hz, 1C), 17.2 (d, *J* = 19 Hz, 1C).

^{13}C{^{1}H} NMR (126 MHz, DMSO at 60°C): 159.0, 156.2, 136.4, 133.4, 133.1, 132.1, 131.7., 131.0, 130.7, 130.4, 130.0, 128.9, 127.7, 127.3, 126.7, 125.6, 125.1, 124.7, 123.4, 122.1, 121.4, 83.1, 47.1, 46.9, 33.8, 30.7, 26.7, 26.6, 23.8, 21.6, 20.2, 20.1, 17.2, 17.1.

$[\alpha]_D^{23}$ = +36.9 (*c* 1.0, $CHCl_3$)

HRMS (EI): Calcd. for $C_{40}H_{43}N_2O_3B_1$ [M+H]+: 611.3446. Found: 611.3443.

Compound 5f

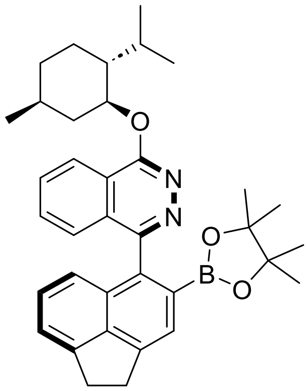

Following the general procedure **GP3**, heterobiaryl(+)-**3f** and diboronic ester **DB3** were employed. The isolation was performed via column chromatography over silica using *n*-heptane/EtOAc (1:1 v/v) as eluent and yielded 45.1 mg (0.080 mmol, 80%) as a pale-yellow solid.

^{1}H NMR (300 MHz, $CDCl_3$): δ = 8.31 (d, *J* = 8.1 Hz, 1H), 7.81 (s, 1H), 7.77 (ddd, *J* = 8.2, 7.3, 1.0 Hz, 1H), 7.58 (ddd, *J* = 8.2, 7.1, 1.0 Hz, 1H), 7.40-7.27 (m, 4H), 5.73-5.55 (m, 1H), 3.50 (s, 4H), 2.74-2.57 (m, 1H), 2.36-2.11 (m, 1H), 1.88-1.60 (m, 5H), 1.37-0.77 (m, 28H).

^{13}C{^{1}H} NMR (75 MHz, $CDCl_3$): δ = 159.5, 156.5, 146.0, 145.9, 140.7, 137.0, 131.3, 131.0, 130.9, 130.8, 128.1, 126.4, 124.0, 122.8, 121.9, 120.4, 120.1, 83.4, 76.3, 48.1, 40.5, 34.8, 32.0, 27.1, 24.6, 24.5, 24.3, 22.3, 21.0, 17.4. ($^{\#}$ = rotamers)

$[\alpha]_D^{23}$ = +93.4 (*c* 1.0, $CHCl_3$)

HRMS (EI): Calcd. for $C_{36}H_{43}N_2O_3B_1$ [M+H]+: 563.3446. Found: 563.3446.

Compound 5g

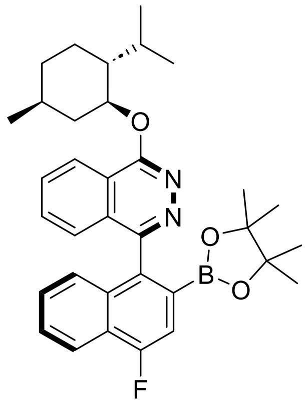

Following the general procedure **GP3**, heterobiaryl(+)-**3c** and diboronic ester **DB3** were employed. The isolation was performed via column chromatography over silica using *n*-heptane/EtOAc (1:1 v/v) as eluent and yielded 42.1 mg (0.076 mmol, 76%) as a pale-yellow solid.

^{1}H NMR (300 MHz, $CDCl_3$): δ = 8.30 (d, *J* = 8.1 Hz, 1H), 8.20 (d, *J* = 8.5 Hz, 1H), 7.76 (ddd, *J* = 8.2, 7.1, 1.1 Hz, 1H), 7.63 (d, *J* = 10.9 Hz, 1H), 7.60-7.47 (m, 3H), 7.38 (ddd, *J* = 8.2, 6.6,

1.1 Hz, 1H), 7.21 (d, *J* = 8.2 Hz, 1H), 5.73-5.51 (m, 1H), 2.74-2.52 (m, 1H), 2.36-2.06 (m, 1H), 1.88-1.57 (m, 5H),1.42-0.70 (m, 28H).

^{13}C{^{1}H} NMR (75 MHz, $CDCl_3$): δ = 159.7, 156.2, 137.7, 134.3, 131.6, 131.1, 131.0, 127.3, 127.2, 127.1, 126.0, 125.2, 125.1, 122.9, 120.6, 120.0, 113.8, 113.7, 83.7, 76.3, 48.1, 40.7$^{\#}$, 40.3$^{\#}$, 34.8, 31.5, 27.2$^{\#}$, 27.1$^{\#}$, 25.0, 24.5, 24.4, 22.3, 21.1$^{\#}$, 20.9$^{\#}$, 17.5. ($^{\#}$ = rotamers)

^{19}F NMR (282 MHz, $CDCl_3$): δ = -123.8.

$[\alpha]_D^{23}$ = +28.2 (*c* 1.0, $CHCl_3$)

HRMS (EI): Calcd. for $C_{34}H_{40}N_2O_3B_1F$ [M+H]+: 555.3195. Found: 555.3193.

Compound 5h

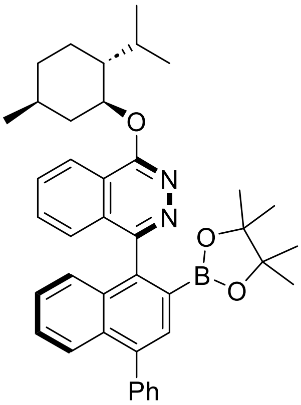

Following the general procedure **GP3**, heterobiaryl(+)-**3d** and diboronic ester **DB3** were employed. The isolation was performed via column chromatography over silica using *n*-heptane/EtOAc (1:1 v/v) as eluent and yielded 41.0 mg (0.067 mmol, 67%) as an off-white solid.

^{1}H NMR (300 MHz, $CDCl_3$): δ = 8.31 (d, *J* = 8.1 Hz, 1H), 7.98 (d, *J* = 8.6 Hz, 1H), 7.90 (s, 1H), 7.78 (t, *J* = 7.3 Hz, 1H), 7.66-7.56 (m, 4H), 7.55-7.41 (m, 4H), 7.34 (t, *J* = 6.9 Hz, 1H), 5.77-5.45 (m, 1H), 2.74-2.57 (m, 1H), 2.36-2.11 (m, 1H), 1.88-1.55 (m, 5H),1.38-0.70 (m, 28H).

^{13}C{^{1}H} NMR (75 MHz, $CDCl_3$): δ = 159.7, 157.0, 141.2, 140.9, 140.1, 133.2, 132.9, 131.6, 131.5, 131.0, 130.4, 128.3, 127.5, 127.4, 127.0, 126.3, 126.1, 122.9, 120.1, 83.5, 76.2, 48.2$^{\#}$, 48.1$^{\#}$, 40.8$^{\#}$, 40.3$^{\#}$, 34.8, 34.7, 31.5$^{\#}$, 31.4$^{\#}$, 27.2$^{\#}$, 27.1$^{\#}$, 24.7$^{\#}$, 24.6$^{\#}$, 24.4, 22.3, 21.1$^{\#}$, 21.0$^{\#}$, 17.5$^{\#}$, 17.4$^{\#}$. ($^{\#}$ = rotamers)

$[\alpha]_D^{23}$ = +23.1 (*c* 1.0, $CHCl_3$)

HRMS (EI): Calcd. for $C_{40}H_{45}N_2O_3B_1$ [M+H]+: 613.3603. Found: 613.3607.

Compound 5i

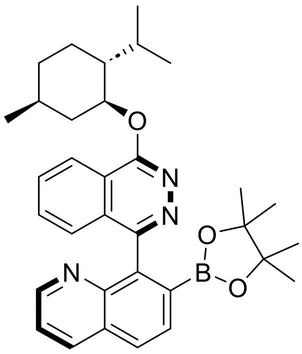

Following the general procedure **GP3**, heterobiaryl(+)-**3g** and diboronic ester **DB3** were employed. The isolation was performed via column chromatography over silica using *n*-heptane/EtOAc (1:1 v/v) as eluent and yielded 38.7 mg (0.072 mmol, 72%) as a pale-yellow solid.

^{1}H NMR (300 MHz, $CDCl_3$): δ = 8.95 (dd, *J* = 4.2, 1.6 Hz, 1H), 8.39-8.17 (m, 2H), 8.01 (d, *J* = 8.3 Hz, 1H), 7.95-7.74 (m, 2H), 7.74-7.65 (m, 1H), 7.64-7.43 (m, 1H), 7.30 (q, *J* = 4.2 Hz, 1H), 2.79-2.56 (m, 1H), 2.36-2.13 (m, 1H), 1.91-1.57 (m, 6H), 1.47-0.79 (m, 27H)

^{13}C{^{1}H} NMR (75 MHz, $CDCl_3$): δ = 160.0, 154.4, 151.4, 150.7, 148.4, 135.3, 134.6, 134.4, 134.3, 132.2, 131.9, 131.7, 131.3, 130.7, 129.2, 129.0, 128.8, 127.8, 126.0, 125.8, 123.5, 121.5, 83.7, 75.1, 48.0, 40.5$^{\#}$, 40.3$^{\#}$, 34.7, 31.5, 27.0, 25.0, 24.4, 24.1, 22.2, 21.0, 17.2.

($^{\#}$ = rotamers)

$[\alpha]_D^{23}$ = +28.6 (*c* 1.0, $CHCl_3$)

HRMS (EI): Calcd. for $C_{33}H_{40}N_3O_3B_1$ [M+H]+: 538.3241. Found: 538.3248.

Compound 5j

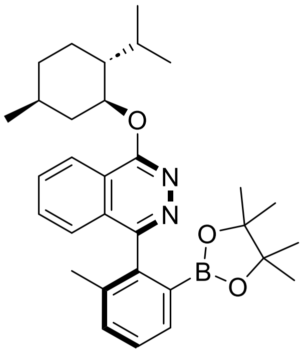

Following the general procedure **GP3**, heterobiaryl(+)-**3h** and diboronic ester **DB3** were employed. The isolation was performed via column chromatography over silica using *n*-heptane/EtOAc (1:1 v/v) as eluent and yielded 36.0 mg (0.072 mmol, 72%) as a yellow solid.

^{1}H NMR (300 MHz, $CDCl_3$): δ = 8.25 (d, *J* = 7.8 Hz, 1H), 7.82-7.68 (m, 2H), 7.62 (ddd, *J* = 8.3, 7.1, 1.3 Hz, 1H), 7.46-7.35 (m, 2H), 7.31 (d, *J* = 8.0 Hz, 1H), 5.65-5.49 (m, 1H), 2.66-2.51 (m, 1H), 2.24-2.15 (m, 1H), 2.13 (s, 3H), 1.86-1.57 (m, 5H), 1.45-0.74 (m, 2H).

$^{13}C\{^1H\}$ NMR (75 MHz, $CDCl_3$): δ = 159.3, 157.5, 141.9, 136.7, 132.8, 132.7, 131.4, 130.9, 130.2, 128.0, 125.8, 122.9, 120.2, 83.2, 76.2, 48.1, 40.6, 34.8, 31.4, 27.1, 24.39, 24.37, 24.3, 22.3, 21.0, 20.1, 17.4. ($^{\#}$ = rotamers)

$[\alpha]_D^{23}$ = +43.4 (*c* 1.0, $CHCl_3$)

HRMS (EI): Calcd. for $C_{31}H_{41}N_2O_3B_1$ [M+H]+: 501.3288. Found: 501.3289.

Compound 5k

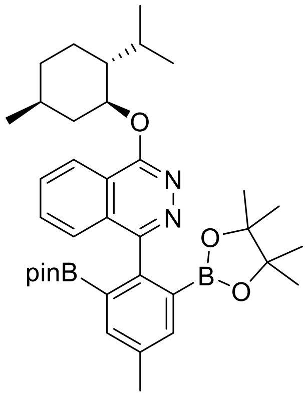

Following the general procedure **GP3**, heterobiaryl(+)-**3i** and diboronic ester **DB3** were employed. The isolation was performed via column chromatography over silica using *n*-heptane/EtOAc (1:1 v/v) as eluent and yielded 45.1 mg (0.072 mmol, 72%) as a pale-yellow solid.

^1H NMR (300 MHz, $CDCl_3$): δ = 8.30 (d, *J* = 8.1 Hz, 1H), 7.97 (s, 2H), 7.92 (d, *J* = 8.2 Hz, 1H), 7.76 (ddd, *J* = 8.1, 7.1, 1.1 Hz, 1H), 7.60-7.47 (m, 3H), 7.33 (ddd, *J* = 8.2, 7.1, 1.2 Hz, 1H), 7.25-7.19 (m, 1H), 5.73-5.51 (m, 1H), 2.74-2.57 (m, 1H), 2.36-2.11 (m, 1H), 1.88-1.60 (m, 5H), 1.42-1.15 (m, 6H), 1.09-0.83 (m, 12H), 1.34-0.84 (m, 8H), 0.79 (s, 3H).

$^{13}C\{^1H\}$ NMR (75 MHz, $CDCl_3$): δ = 159.0, 158.8, 145.7, 137.6, 136.4, 131.2, 130.8, 130.2, 126.6, 122.4, 119.7, 83.3, 75.8, 48.1, 40.5, 34.8, 31.4, 27.3, $24.5^{\#}$, $24.4^{\#}$, 22.3, 21.2, 20.8, 17.4. ($^{\#}$ = rotamers)

HRMS (EI): Calcd. for $C_{37}H_{52}N_2O_5B_2$ [M+H]+: 627.4148. Found: 627.4150.

Compound 5m-SI

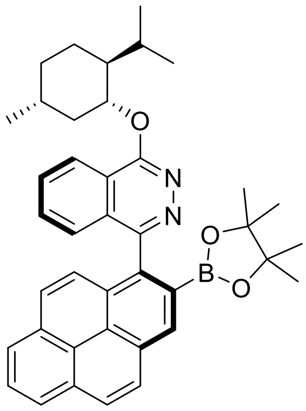

Following the general procedure **GP3**, heterobiaryl (-)-**3e** and diboronic ester **DB3** were employed. The isolation was performed via column chromatography over silica using *n*-heptane/EtOAc (1:1 v/v) as eluent and yielded 42.0 mg (0.069 mmol, 69%) as a yellow solid.

^{1}H NMR (300 MHz, $CDCl_3$): δ = 8.76 (s, 1H), 8.36 (d, *J* = 7.8 Hz, 1H), 8.23 (d, *J* = 7.6 Hz, 1H), 8.20-8.11 (m, 3H) , 8.06 (t, *J* = 7.6 Hz, 1H), 7.94 (d, *J* = 9.2 Hz, 1H), 7.83-7.72 (m, 1H), 7.61-7.50 (m, 1H), 7.24-7.12 (m, 1H), 5.75-5.54 (m, 1H), 2.70-2.56 (m, 1H), 2.31-2.07 (m, 1H), 1.86-1.50 (m, 6H), 1.35-1.20 (m, 9H), 1.10-0.84 (m, 20H), 0.79 (d, *J* = 2.7 Hz, 3H).

^{13}C{^{1}H} NMR (75 MHz, $CDCl_3$): δ = 159.8, 157.2, 132.0, 131.7, 131.6, 131.4, 131.1, 130.8, 130.0, 127.9, 126.7, 126.4, 125.9, 125.4, 125.3, 124.7, 122.9, 83.7, 48.2$^\#$, 48.1$^\#$, 40.8$^\#$, 40.3$^\#$, 34.8, 31.6$^\#$, 31.4$^\#$, 27.3$^\#$, 27.1$^\#$, 24.7, 24.6, 24.5, 24.3, 22.3, 21.0, 17.5$^\#$, 17.4$^\#$. ($^\#$ = rotamers)

$[\alpha]_D^{23}$ = -+31.2 (*c* 1.0, $CHCl_3$)

HRMS (EI): Calcd. for $C_{40}H_{43}N_2O_3B_1$ [M+H]+: 611.3446. Found: 611.3446.

Compound 5n-SI

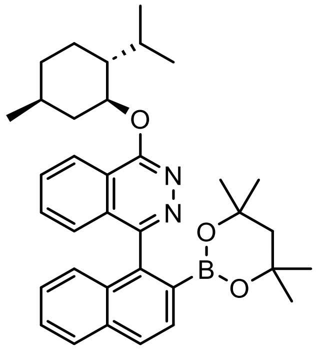

Following the general procedure **GP3**, heterobiaryl (+)-**3a** and diboronic ester **DB4** were employed. The isolation was performed via column chromatography over silica using *n*-heptane/EtOAc (1:1 v/v) as eluent and yielded 28.1 mg (0.051 mmol, 51%) as a yellow solid.

^{1}H NMR (300 MHz, CDCl3): δ = 8.30 (d, J = 8.1 Hz, 1H), 8.05 (dd, J = 8.3, 2.3 Hz, 1H), 7.93 (ddd, J = 15.7, 8.3 Hz, 2H), 7.78 (t, J = 7.4 Hz, 1H), 7.55 (t, J = 7.5 Hz, 1H), 7.60-7.37 (m, 2H), 7.38-7.26 (m, 2H), 5.68-5.49 (m, 1H), 2.76-2.59 (m, 1H), 2.38-2.12 (m, 1H), 1.88-1.62 (m, 6H), 1.61-1.41 (m, 3H), 1.35-1.17 (m, 5H), 1.06-0.80 (m, 10H).

$^{13}C\{^1H\}$ NMR (75 MHz, CDCl3) (mixture of regioisomers) : δ = 159.9, 159.6, 159.5, 157.9, 157.8, 134.6, 132.9, 132.8, 131.9, 131.7, 131.4, 131.21, 131.17, 130.9, 130.58, 130.56, 129.3, 128.3, 127.9, 127.7, 126.9, 126.6, 126.5, 126.40, 126.38, 126.2, 126.1, 126.0, 125.3, 123.3, 122.7, 120.0, 70.7, 70.6, 48.3, 48.1, 40.6, 40.3, 34.7, 31.5, 31.3, 31.1, 27.0, 26.9, 26.8, 24.1, 24.0, 22.30, 22.27, 21.1, 17.3, 17.1, 16.2.

HRMS (EI): Calcd. for $C_{35}H_{43}N_2O_3B$ [M+H]+: 551.3446. Found: 551.3450.

Compound 5o-SI

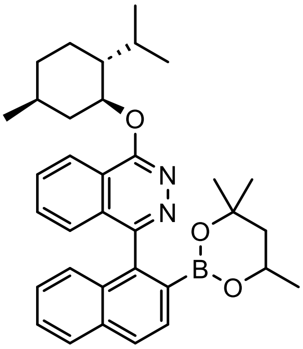

Following the general procedure **GP3**, heterobiaryl (+)-**3a** and diboronic ester **DB5** were employed. The isolation was performed via column chromatography over silica using *n*-heptane/EtOAc (1:1 v/v) as eluent and yielded 31.1 mg (0.058 mmol, 58%) as a yellow solid.

^{1}H NMR (300 MHz, CDCl3): δ = 8.30 (d, J = 8.2 Hz, 1H), 8.05-7.99 (m, 1H), 7.93 (ddd, J = 15.0, 8.3 Hz, 2H), 7.78 (t, J = 7.4 Hz, 1H), 7.60-7.27 (m, 4H), 5.68-5.49 (m, 1H), 4.48-3.83 (m, 1H), 2.76-2.58 (m, 1H), 2.36-2.11 (m, 1H), 2.00-1.60 (m, 6H), 1.46-1.36 (m, 2H), 1.36-1.12 (m, 6H), 1.07-0.78 (m, 16H), 0.74 (d, J = 6.2 Hz, 1H), 0.63 (d, J = 4.7 Hz, 1H).

^{13}C{^{1}H} NMR (75 MHz, CDCl3) (mixture of diastereomers): δ = 159.6, 157.8, 134.6, 134.5, 132.8, 131.9, 131.4, 130.5, 129.3, 128.5, 128.4, 128.0, 127.9, 127.7, 126.9, 126.52, 126.49, 126.44, 126.41, 126.2, 126.1, 125.4, 122.7, 70.9, 70.8, 70.7, 64.9, 64.8, 50.2, 48.1, 45.4, 40.6, 40.5, 40.4, 34.7, 31.5, 30.8, 30.4, 27.5, 27.0, 26.9, 26.8, 25.9, 24.2, 24.1, 24.0, 23.2, 22.8, 22.6, 22.3, 21.1, 17.3, 17.2, 16.2.

HRMS (EI): Calcd. for $C_{34}H_{41}N_2O_3B$ [M+H]+: 537.3289. Found: 537.3288.

Compound 5p-SI

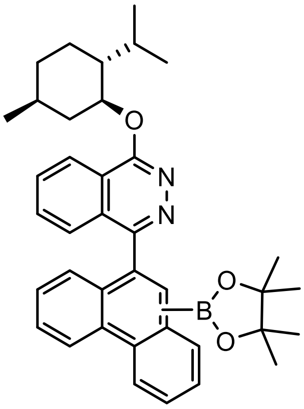

Following the general procedure **GP3**, heterobiaryl (+)-**3k** and diboronic ester **DB3** were employed. The isolation was performed via column chromatography over silica using *n*-heptane/EtOAc (1:1 v/v) as eluent and yielded 38.2 mg (0.065 mmol, 65%) as a yellow solid.

^{1}H NMR (300 MHz, CDCl3): δ = 8.79 (dd, J = 8.1, 1.5 Hz, 1H), 8.39 (d, J = 7.8 Hz, 1H), 8.31 (d, J = 8.2 Hz, 1H), 7.81 (t, J = 7.2 Hz, 1H), 7.73-7.52 (m, 4H), 7.36 (dd, J = 5.0, 2.9 Hz, 1H),

5.70-5.51 (m, 1H), 2.75-2.60 (m, 1H), 2.35-2.12 (m, 1H), 1.89-1.53 (m, 5H), 1.32-1.22 (m, 10H), 1.06-0.93 (m, 20H), 0.92-0.86 (m, 3H).

^{13}C{^{1}H} NMR (75 MHz, CDCl3) (mixture of regioisomers): δ = 159.9, 157.5, 133.9, 131.8, 131.4, 131.1, 130.5, 130.2, 129.4, 127.6, 127.3, 127.1, 126.9, 126.6, 122.84, 122.81, 83.8, 71.6, 48.0, 40.6, 40.3, 34.7, 31.4, 31.3, 27.0, 25.9, 25.1, 24.9, 24.7, 24.1, 23.2, 22.30, 22.28, 21.1, 21.0, 17.3, 16.2.

HRMS (EI): Calcd. for $C_{40}H_{43}N_2O_3B$ [M+H]+: 611.3433. Found: 611.3444

Compound 5q-SI

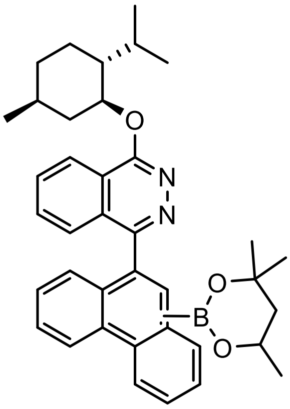

Following the general procedure **GP3**, heterobiaryl (+)-**3k** and diboronic ester **DB5** were employed. The isolation was performed via column chromatography over silica using *n*-heptane/EtOAc (1:1 v/v) as eluent and yielded 23.6 mg (0.040 mmol, 40%) as a pale-yellow solid.

^{1}H NMR (300 MHz, CDCl3): δ = 8.85-8.71 (m, 2H), 8.67-8.28 (m, 1H), 8.00-7.88 (m, 1H), 7.84-7.31 (m, 8H), 5.70-5.49 (m, 1H), 4.50-3.92 (m, 1H), 2.72-2.52 (m, 1H), 2.34-1.95 (m, 6H), 1.88-1.55 (m, 6H), 1.47-1.35 (m, 2H), 1.32-1.10 (m, 9H), 1.06-0.84 (m,13 H), 0.83-0.70 (m, 3H).

^{13}C{^{1}H} NMR (75 MHz, CDCl3) (mixture of regioisomers) : δ = 155.8, 153.5, 153.3, 137.0, 136.9, 136.4, 136.1, 135.2, 132.0, 131.8, 131.4, 130.8, 130.7, 130.4, 129.7, 129.3, 127.3, 127.1, 127.0, 126.8, 126.6, 123.4, 123.1, 120.8, 120.1, 71.9, 71.7, 71.6, 65.6, 65.4, 65.3, 50.3, 48.0, 46.2, 46.1, 46.0, 45.8, 45.2, 40.6, 40.4, 34.7, 31.8, 31.5, 31.4, 31.1, 30.9, 28.3, 28.2, 27.8, 27.0, 26.0, 24.2, 23.2, 23.0, 22.8, 22.6, 22.3, 21.2, 21.0, 17.3, 16.2.

4.3. Synthesis and C-H borylation of problematic substrate motif

The synthesis of a menthylated 3-chloro-4,5-dimethyl-pyridazine from 3,6-dichloro-4,5-dimethyl-pyridazine (**SI-1**) and subsequent reactions suffer from the inseparable residues of (+)-menthol remaining in the purified products, even after successful borylation. Even after further purification attempts the chiral alcohol was still coeluting during chromatography and the products could not be purified satisfyingly. The results and data obtained are listed below.

Substrate (+)-SI-2

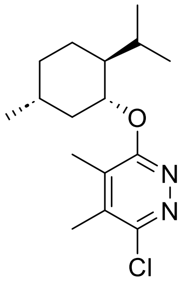

Following **GP1**, (+)-menthol (1.01 mmol, 0.158 g, 1.01 equiv.) was dissolved in 11 mL of dry THF, *n*-BuLi (1.6 M in hexane) (1.05 mmol, 0.67 mL, 1.05 equiv.) 3,6-dichloro-4,5-dimethyl-pyridazine (**SI-1**, 1.0 mmol, 0.177 g, 1.equiv.) dissolved in 5 mL dry THF, were employed. After aqueous work-up, column chromatography over silica using *n*-heptane/EtOAc (3:1 v/v) as eluent was performed and 0.531 g (1.79 mmol) of a tan solid in a yield of 60% was obtained.

^{1}H NMR (300 MHz, $CDCl_3$): δ = 5.29-5.14 (m, 1H), 2.32 (s, 3H), 2.18 (s, 3H), 2.00-1.92 (m, 1H), 1.76-1.44 (m, 3H), 1.32-1.20 (m, 7H), 1.00-0.77 (m, 14H).

^{13}C{^{1}H} NMR (75 MHz, $CDCl_3$) (mixture of diastereomers): δ = 163.2, 151.8, 137.3, 129.2, 77.6, 77.2, 76.9, 76.7, 71.7, 47.8, 40.3, 34.7, 34.6, 31.4, 26.7, 23.8, 22.4, 21.1, 16.9.

$[\alpha]_D^{23}$ = +83.2 (*c* 1.0, $CHCl_3$)

HRMS (ESI): Calcd. for $C_{16}H_{25}N_2OCl$ [M+H]+: 297.1734. Found: 297.1737.

^{1}H NMR ($CDCl_3$, 300 Hz) of **SI-2**

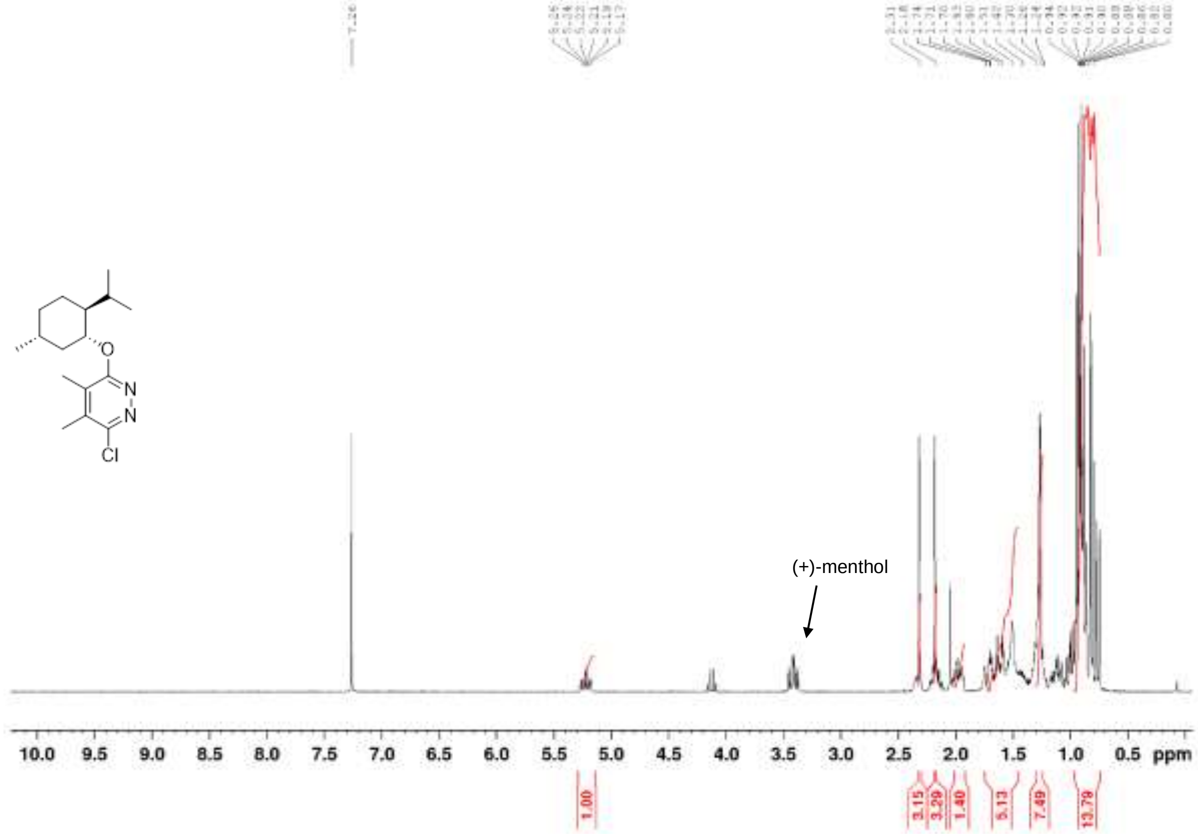

$^{13}C\{^{1}H\}$ NMR ($CDCl_3$, 125 Hz) of **SI-2**

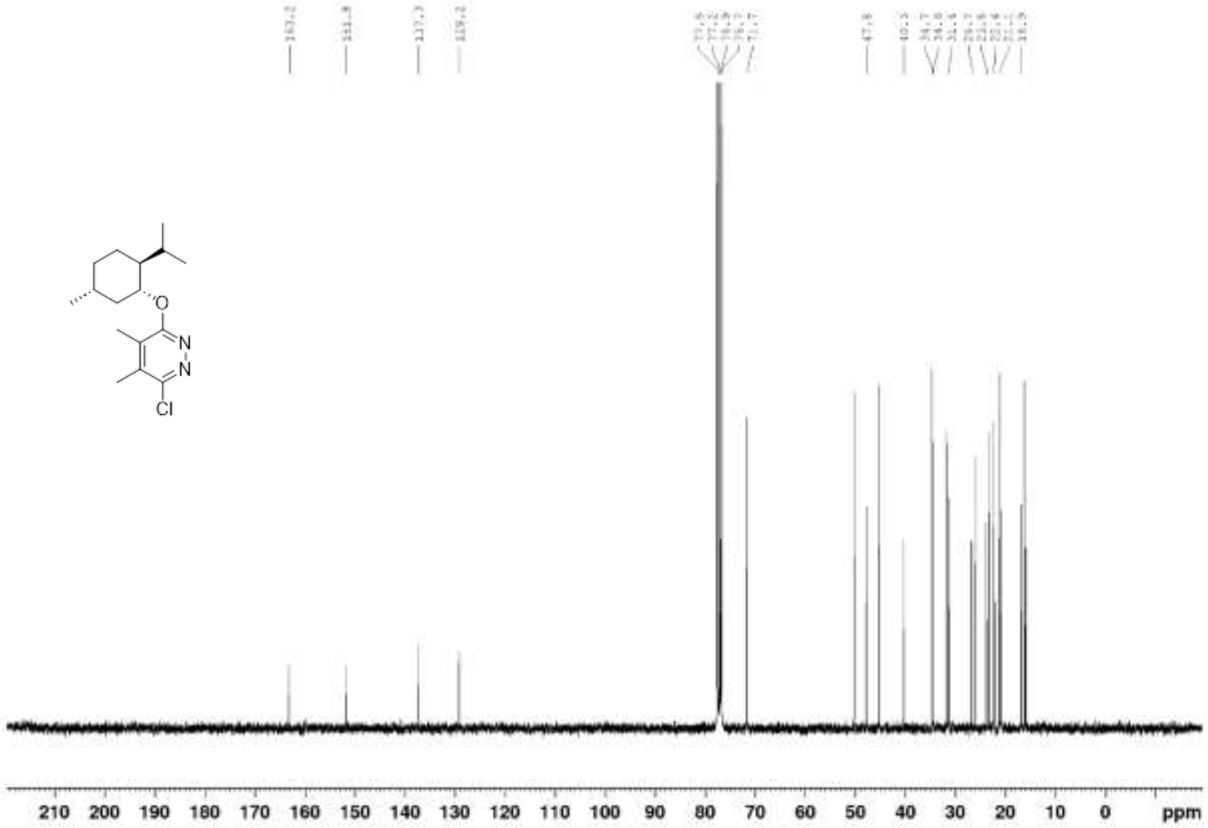

Substrate (+)-SI-3

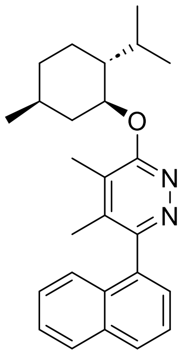

Following **GP2**, **2b** (1.0 mmol, 297 mg), naphthalen-1-yl boronic acid (1.1 mmol, 189 mg, 1.1 equiv.), Na_2CO_3 (2.0 mmol, 212 mg, 2 equiv.) and $Pd(PPh_3)_4$ (3 mol%, 34.5 mg) dissolved in 2 mL degassed toluene, 0.5 mL degassed MeOH as well as 2 mL degassed H_2O were subjected to reaction. After aqueous work-up, column chromatography over silica using *n*-heptane/EtOAc (3:1 v/v) as eluent was performed and 272 mg (0.70 mmol) of an off-white solid in a yield of 70% was obtained.

^{1}H NMR (300 MHz, $CDCl_3$): δ = 7.95-7.84 (m, 2H), 7.53 (dd, *J* = 7.1 Hz, 1H), 7.49-7.34 (m, 4H), 5.46-5.32 (m, 1H), 2.81 (s, 3H), 2.59-2.46 (m, 1H), 2.23 (s, 3H), 2.21-2.11 (m, 1H), 1.92 (s, 3H), 1.82-1.51 (m, 5H), 1.37-0.77 (m, 18H).

^{13}C{^{1}H} NMR (75 MHz, $CDCl_3$) (mixture of diastereomers): δ = 163.1, 156.9, 137.7, 136.0, 133.6, 132.0, 128.7, 128.4, 127.4, 126.4, 126.0, 125.9, 125.6, 125.4, 76.4, 71.5, 47.9, 40.7, 40.6, 34.7, 34.6, 31.4, 26.8, 23.9, 22.3, 22.2, 21.1, 21.0, 17.0.

$[\alpha]_D^{23}$ = +59.0 (*c* 1.0, $CHCl_3$)

HRMS (ESI): Calcd. for $C_{26}H_{32}N_2O_1$ [M+H]+: 389.2587. Found: 389.2589.

^{1}H NMR ($CDCl_3$, 300 Hz) of **SI-3**

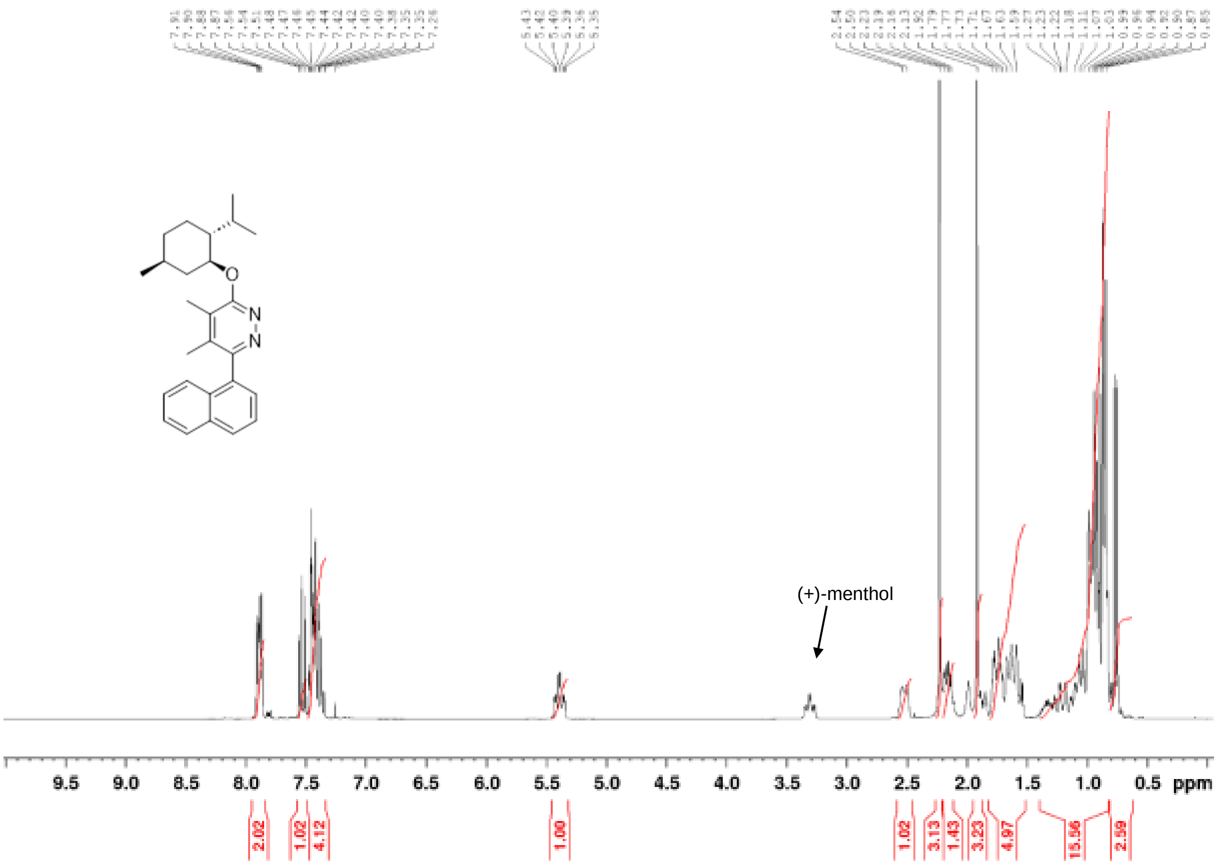

^{13}C{^{1}H} NMR ($CDCl_3$, 125 Hz) of **SI-3**

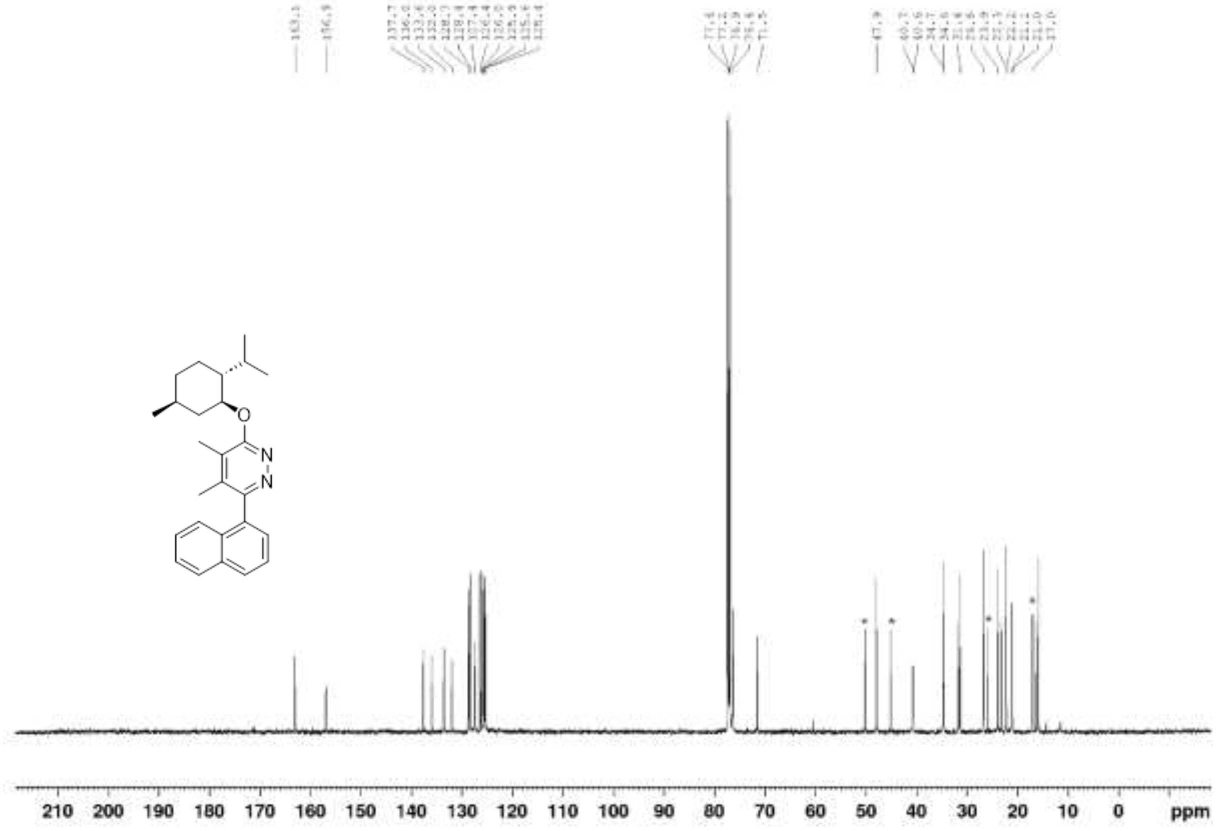

Peaks marked with * indicate impurities stemming from inseparable menthol.

Compound SI-4

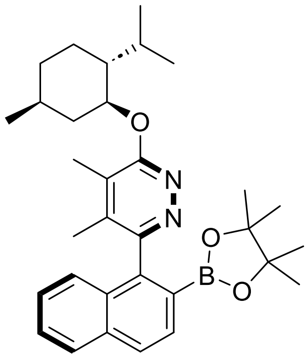

Following the general procedure **GP3**, heterobiaryl(+)-**3l** and diboronic ester **DB3** were employed. The isolation was performed via column chromatography over silica using *n*-heptane/EtOAc (1:1 v/v) as eluent and yielded 36.5 mg (0.071 mmol, 71%) as a pale-yellow solid.

^{1}H NMR (300 MHz, $CDCl_3$): δ = 7.88 (d, *J* = 8.1 Hz, 3H), 7.49 (ddd, *J* = 8.2, 5.8, 2.2 Hz, 1H), 7.44-7.33 (m, 3H), 5.48-5.31 (m, 1H), 2.74-2.57 (m, 1H), 2.58-2.47 (m, 1H), 2.23 (s, 3H), 2.22-2.15 (m, 1H), 1.85 (s, 3H), 1.80-1.58 (m, 5H), 1.31-0.73 (m, 28H).

^{13}C{^{1}H} NMR (75 MHz, $CDCl_3$): δ = 162.9, 157.9, 143.6, 138.4, 134.9, 132.0, 130.6, 128.1, 127.4, 126.8, 126.5, 126.2, 124.7, 83.5, 75.9, 48.0, 34.8, 31.4, 26.9, 24.83, 24.77, 24.1, 22.3, 17.2, 15.6. (* = rotamers)

$[\alpha]_D^{23}$ = +35.2 (*c* 1.0, $CHCl_3$)

HRMS (EI): Calcd. for $C_{32}H_{43}N_2O_3B_1$ [M+H]+: 515.3445. Found: 515.3446.

^{1}H NMR ($CDCl_3$, 300 Hz) of **SI-4**

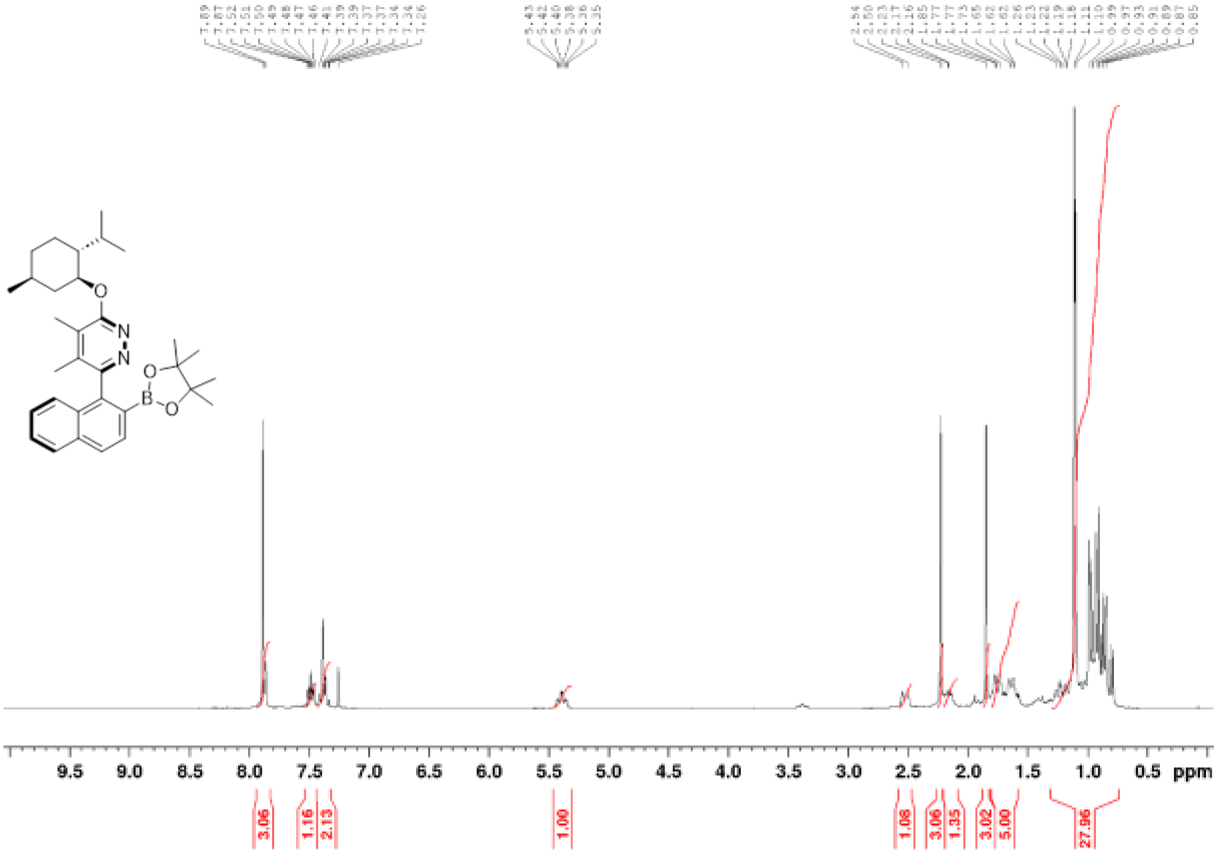

$^{13}C\{^{1}H\}$ NMR ($CDCl_3$, 125 Hz) of **SI-4**

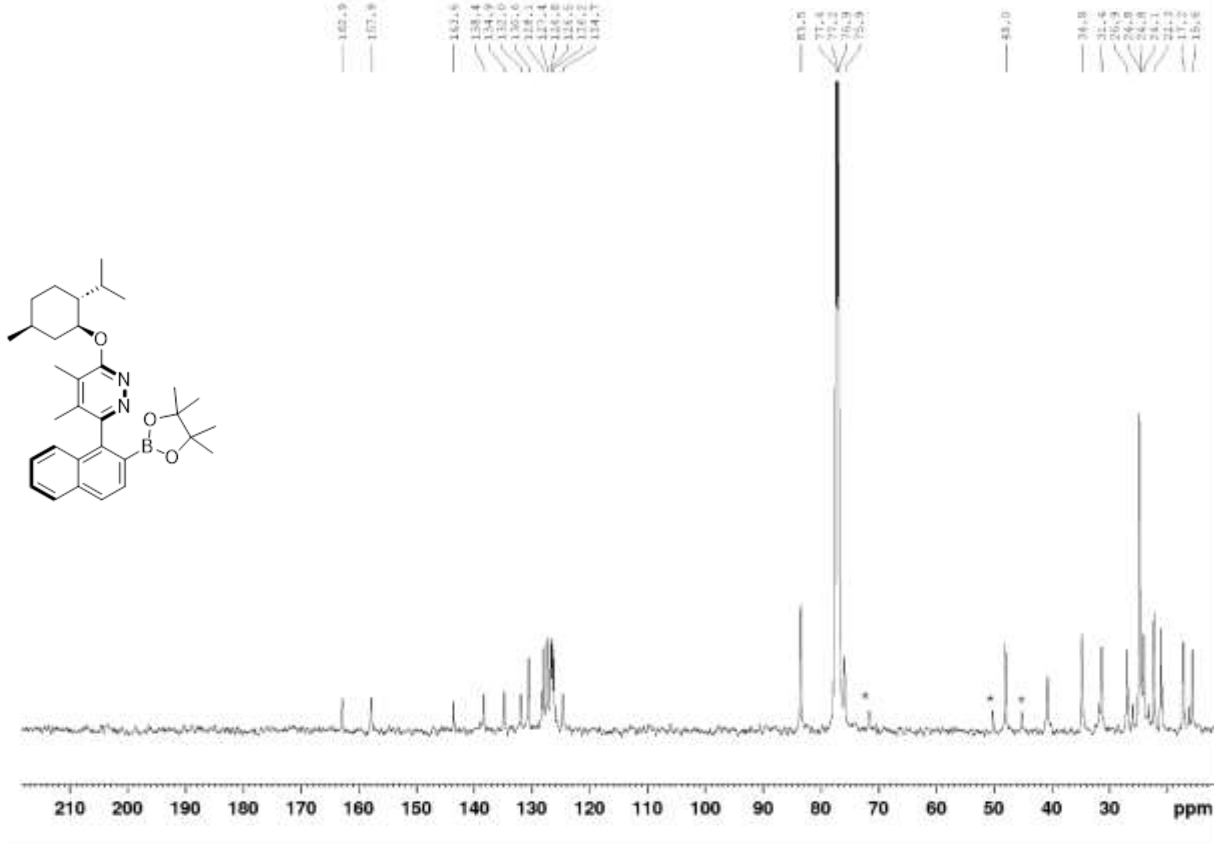

Peaks marked with * indicate impurities stemming from inseparable menthol.

5. Cross-coupling Reactions of Borylated Atropisomers

General procedure for coupling reaction of borylated atropisomers

In an argon-filled glovebox, a 10 mL Schlenk flask was charged with **4g** or **4k-SI** (0.1 mmol, 66.3 mg), the corresponding reagent (1.5 equiv.), the stated catalyst (5 mol%), the corresponding ligand (if stated in Table S3, 5 mol%), the stated base (2.0 equiv.) as well as the stated solvent (0.5 mL). The reaction was then stirred for the annotated time and temperature given in Table S3. The reaction was quenched by exposure to air. The aqueous phase was extracted thrice with EtOAc (5 mL each), and the combined organic phases were dried over Na_2SO_4. Column chromatography over silica using *n*-heptane/EtOAc (5:1, v/v) as eluent was performed. The yields are given in Table S3.

Preparation of stock solution of nickel catalyst

In an argon-filled glovebox, a 4 mL scintillation vial was charged with Ni[P(*n*-Bu)$_3$]$_2$(COD) (0.25 mmol, 12.0 mg), dppb (0.25 mmol, 10.5 mg) and dry 2-Me-THF (0.75 mL). After capping the vial it was placed on a hot plate and stirred for 30 min at 50 °C. The orange solution was then allowed to cool to rt. The amount of 0.15 mL of the stock solution was used to provide the nickel catalyst solution.

Compound (*R*$_a$)-6a (75% *de*)

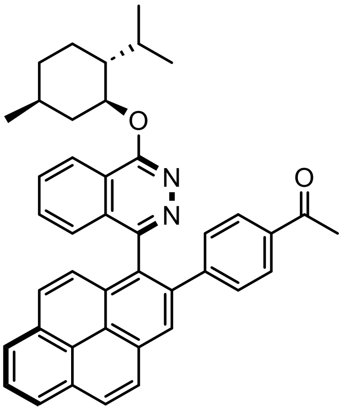

In an argon-filled glovebox, a 10 mL Schlenk flask was charged with **4g** (0.1 mmol, 66.3 mg), 4-iodo-acetophenone (0.15 mmol, 36.9 mg, 1.5 equiv.), *i*Pr$_2$EtN (0.2 mmol, 34.8 µL, 2.0 equiv.) and the nickel catalyst solution (5 mol%, 0.15 mL). After transferring the flask outside of the glovebox H_2O (0.25 mL) was added and the reaction was stirred overnight at 50 °C. The reaction was quenched by exposure to air. The aqueous phase was extracted thrice with EtOAc (5 mL), and the combined organic phases were dried over Na_2SO_4. Column chromatography over silica using *n*-heptane/EtOAc (5:1 v/v) as eluent was performed and 49.4 mg (0.082 mmol) of a yellow amorphous solid in a yield of 82% and 75% *de* was obtained. **^{1}H NMR** (300 MHz, CDCl3): δ = 8.30 (s, 1H), 8.27 (dd, *J* = 7.6, 0.9 Hz, 1H), 8.25-8.16 (m, 4H), 8.06 (t, *J* = 7.6 Hz, 1H), 7.98 (dd, *J* = 9.3, 2.5 Hz, 1H), 7.75-7.58 (m, 4H), 7.52-7.38 (m, 3H),

7.10 (dd, *J* = 8.2, 2.6 Hz, 1H) , 5.68-5.49 (m, 1H), 2.76-2.59 (m, 1H), 2.49, (d, *J* = 7.7, 3H) 2.17-1.97 (m, 1H), 1.88-1.62 (m, 4H), 1.35-1.17 (m, 4H), 1.06-0.76 (m, 10H).

^{13}C{^{1}H} NMR (75 MHz, CDCl3) (mixture of stereoisomers): δ = 197.9, 159.9, 155.3, 146.6, 139.4, 135.3, 132.03, 132.01, 131.8, 131.7, 131.5, 130.98, 130.96, 130.5, 130.4, 130.0, 129.9, 128.8, 128.6, 127.8, 127.4, 126.5, 126.0, 125.9, 125.79, 125.76, 125.6, 124.5, 124.4, 123.3, 119.8, 60.5, 48.2, 40.7, 34.7, 31.6, 27.1, 24.2, 22.3, 21.2, 20.9, 17.1.

$[\alpha]_D^{23}$ = +26.1 (*c* 1.0, $CHCl_3$)

HRMS (EI): Calcd. for $C_{42}H_{38}N_2O_2$ [M+H]+: 605.3163. Found: 605.3160.

Compound (*S*$_a$)-6a (67% *de*)

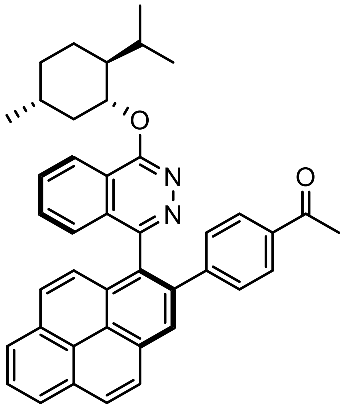

In an argon-filled glovebox, a 10 mL Schlenk flask was charged with **4k-SI** (0.1 mmol, 66.3 mg), 4-iodo-acetophenone (0.15 mmol, 36.9 mg, 1.5 equiv.), *i*Pr_2EtN (0.2 mmol, 34.8 µL, 2.0 equiv.) and the nickel catalyst solution (5 mol%, 0.15 mL). After transferring the flask outside of the glovebox H_2O (0.25 mL) was added and the reaction was stirred overnight at 50 °C. The reaction was quenched by exposure to air. The aqueous phase was extracted thrice with EtOAc (5 mL), and the combined organic phases were dried over Na_2SO_4. Column chromatography over silica using *n*-heptane/EtOAc (5:1 v/v) as eluent was performed and 49.4 mg (0.082 mmol) of a yellow amorphous solid in a yield of 82% and 67% *de* was obtained.

^{1}H NMR (300 MHz, CDCl3): δ = 8.30 (s, 1H), 8.27 (dd, *J* = 7.6, 0.9 Hz, 1H), 8.24-8.16 (m, 4H), 8.06 (t, *J* = 7.6 Hz, 1H), 7.98 (dd, *J* = 9.3, 2.5 Hz, 1H), 7.75-7.58 (m, 4H), 7.52-7.38 (m, 3H), 7.10 (dd, *J* = 8.2, 2.6 Hz, 1H) , 5.68-5.49 (m, 1H), 2.76-2.59 (m, 1H), 2.49 (d, *J* = 7.7, 3H), 2.17-1.97 (m, 1H), 1.88-1.62 (m, 4H), 1.35-1.17 (m, 4H), 1.06-0.76 (m, 10H).

^{13}C{^{1}H} NMR (75 MHz, CDCl3) (mixture of stereoisomers): δ = 197.9, 159.9, 155.3, 146.6, 139.4, 135.3, 132.0, 131.8, 131.7, 131.5, 131.0, 130.4, 130.0, 128.8, 128.6, 127.8, 127.4, 126.5, 126.0, 125.9, 125.8, 125.6, 124.5, 124.4, 123.3, 119.8, 60.5, 48.2, 40.7, 34.7, 31.6, 27.1, 24.2, 22.3, 21.2, 20.9, 17.1.

$[\alpha]_D^{23}$ = -25.6 (*c* 1.0, $CHCl_3$)

HRMS (EI): Calcd. for $C_{42}H_{38}N_2O_2$ [M+H]+: 605.3163. Found: 605.3165.

Table S3: Screening table of catalytic cross-coupling conditions for borylated heterobiaryls.

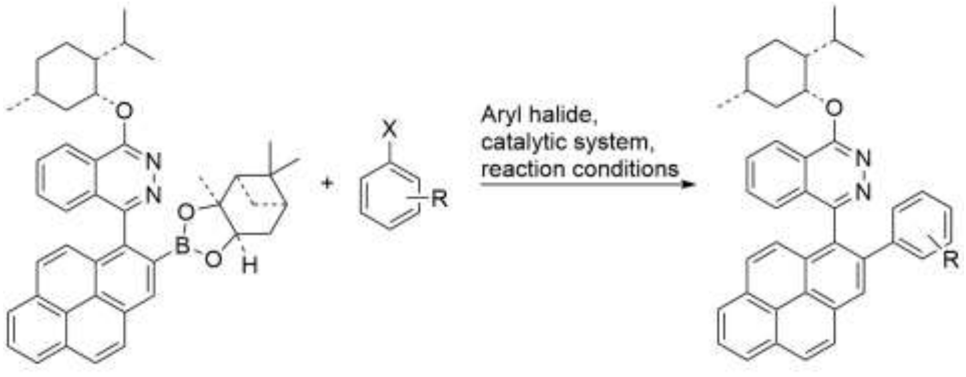

4i or **4j**

Entry	Molecule + [M] + Ligand	Coupling partner	Additive/Base	Solvent	T [°C]	T [h]	de	Yield [%]
1	**4i**, $Pd(PPh_3)_4$	4-Bromoanisole	CsF	THF	60	12	46	86
2	**4j**, $Pd(PPh_3)_4$	4-Bromoanisole	CuCl, CsF	THF	80	12	0	16
3	**4j**, $Pd(OAc)_2$, dppf	4-Bromoanisole	CuCl, Cs_2CO_3	DMF	60	12	0	48
4	**4i**, $Pd(dba)_2$, Xantphos	4-Bromoanisole	Cs_2CO_3	THF	60	12	0	41
5	**4i**, $Pd(dba)_2$, BINAP	4-Bromoanisole	Cs_2CO_3	THF	60	12	0	57
6	**4j**, $Pd(dppf)Cl_2$	Iodobenzene	Na_2CO_3	THF/H_2O	80	12	10	75
7	**4i**, $Pd(PPh_3)_4$	Iodobenzene	Na_2CO_3	Toluene/MeOH/H_2O	80	12	36	82
8	**4j**, $Pd(PPh_3)_4$	4-Bromoanisole	Cs_2CO_3	THF	80	12	44	79
9	**4j**, $Pd(PPh_3)_4$	4-Bromoanisole	ZnI, Cs_2CO_3	THF	60	12	-	-
13[8]	**4j**, $Pd(OAc)_2$, XPhos	4-Bromoanisole	NaOH	*n*-BuOH/H_2O	rt	12	-	-
14[9]	**4j**, $Pd(OAc)_2$, IPr	4-Bromoanisole	CsF	THF	50	12	20	50
15	**4i**, Ni[P(*n*-Bu)$_3$]$_2$(COD), dppb	4-Bromoaceto-phenone	*i*-Pr_2EtN	2-Me THF/H_2O	50	12	74	81
16	**4i**, $Ni(COD)_2$, dppb	4-Iodoaceto-phenone	*i*-Pr_2EtN	2-Me THF/H_2O	50	12	25	75
17[10]	**4i**, Ni[P(*n*-Bu)$_3$]$_2$(COD), dppb	4-Iodoaceto-phenone	*i*-Pr_2EtN	2-Me THF/H_2O	50	12	75	82
18	**4j**, Ni[P(*n*-Bu)$_3$]$_2$(COD), dppb	4-Iodoaceto-phenone	*i*-Pr_2EtN	2-Me THF/H_2O	50	12	67	82

Entry	Molecule + [M] + Ligand	Coupling partner	Additive/Base	Solvent	T [°C]	T [h]	de	Yield [%]
19	**4i**, Ni[P(*n*-Bu)$_3$]$_2$(COD) (1 equiv.), dppb (1 equiv.)	4-Iodoaceto-phenone	*i*-Pr$_2$EtN	2-Me THF/H_2O	50	12	72	85
20	**4i**, Ni[P(*n*-Bu)$_3$]$_2$(COD), dppe	4-Iodoaceto-phenone	*i*-Pr$_2$EtN	2-Me THF/H_2O	50	12	63	9
21	**4i**, Ni[P(*n*-Bu)$_3$]$_2$(COD), dcpe	4-Iodoaceto-phenone	*i*-Pr$_2$EtN	2-Me THF/H_2O	50	12	62	6
22	**4i**, Ni[P(*n*-Bu)$_3$]$_2$(COD), dppp	4-Iodoaceto-phenone	*i*-Pr$_2$EtN	2-Me THF/H_2O	50	12	64	11
23	**4i**, Ni[P(*n*-Bu)$_3$]$_2$(COD), dcpp	4-Iodoaceto-phenone	*i*-Pr$_2$EtN	2-Me THF/H_2O	50	12	63	65
24	**4i**, Ni[P(*n*-Bu)$_3$]$_2$(COD), dpppent	4-Iodoaceto-phenone	*i*-Pr$_2$EtN	2-Me THF/H_2O	50	12	70	83
25	**4i**, Ni[P(*n*-Bu)$_3$]$_2$(COD), PCy_3	4-Iodoaceto-phenone	*i*-Pr$_2$EtN	2-Me THF/H_2O	50	12	10	85
26	Ni[P(*n*-Bu)$_3$]$_2$(COD)	4-Iodoaceto-phenone	*i*-Pr$_2$EtN	2-Me THF/H_2O	rt	12	72	67
26	Ni(dppb)(COD), dppb	4-Iodoaceto-phenone	*i*-Pr$_2$EtN	2-Me THF/H_2O	50	12	70	65
27	Ni(dppb)(COD)	4-Iodoaceto-phenone	*i*-Pr$_2$EtN	2-Me THF/H_2O	50	12	68	88
28	Ni(dppb)(COD)	4-Iodoaceto-phenone	*i*-Pr$_2$EtN	2-Me THF/H_2O	rt	12	71	85
29[a]	**4i**, Ni[P(*n*-Bu)$_3$]$_2$(COD), dppb	4-Iodoaceto-phenone	*i*-Pr$_2$EtN	2-Me THF/H_2O	50	0.5	70	10
30[b]	**4i**, Ni[P(*n*-Bu)$_3$]$_2$(COD), dppb	4-Iodoaceto-phenone	*i*-Pr$_2$EtN	2-Me THF/H_2O	50	2	69	33
31[c]	**4i**, Ni[P(*n*-Bu)$_3$]$_2$(COD), dppb	4-Iodoaceto-phenone	*i*-Pr$_2$EtN	2-Me THF/H_2O	50	4	71	58

dba = dibenzylideneacetone, XPhos = dicyclohexyl[2′,4′,6′-tris(propan-2-yl)[1,1′-biphenyl]-2-yl]phosphifne, BINAP = ([1,1′-binaphthalene]-2,2′-diyl) bis(diphenylphosphine), IPr = 1,3-bis-(2,6-diisopropylphenyl)-imidazolium chloride, dppf = 1,1'-bis(diphenylphosphino)ferrocene, dppb = 1,4-bis(diphenylphosphino)butane, COD = 1,5-cyclooctadiene, P(*n*-Bu)$_3$ = tri-*n*-butylphosphine, dppe = 1,2-bis(diphenylphosphino)ethane, dcpe =1,2-bis(dicyclohexylphosphino)ethane, dppp = 1,3-bis(diphenylphosphino)propane, dcpp = 1,3-bis(dicyclohexylphosphino)propane, dpppent = 1,5-bis(diphenylphosphino)pentane, PCy_3 = tricyclohexylphosphine.

6. SC-XRD-Data

X-ray quality crystals were grown via slow evaporation from a concentrated solution of **5c** in DCM layered with *n*-pentane at ambient temperature. Data were collected on a Bruker Kappa APEX II Duo diffractometer. The structure was solved by direct methods[11] and refined by full-matrix least-squares procedures on F^2.[12] H atoms were placed in idealized positions with d(C—H) = 0.95-1.00 Å (CH), 0.99 Å (CH_2), 0.98 Å (CH_3) and refined using a riding model with U_{iso}(H) fixed at 1.2 U_{eq}(C) for CH and CH_2 and 1.5 U_{eq}(C) for CH_3. A rotating model was used for the methyl groups.

CCDC 2224469 contains the supplementary crystallographic data for this paper. These data are provided free of charge by the joint Cambridge Crystallographic Data Centre and Fachinformationszentrum Karlsruhe Access Structures service www.ccdc.cam.ac.uk/structures.

Figure S1: Molecular structure of **5c** (thermal ellipsoids drawn at the 50% probability level at 150 K, hydrogen atoms omitted for clarity).

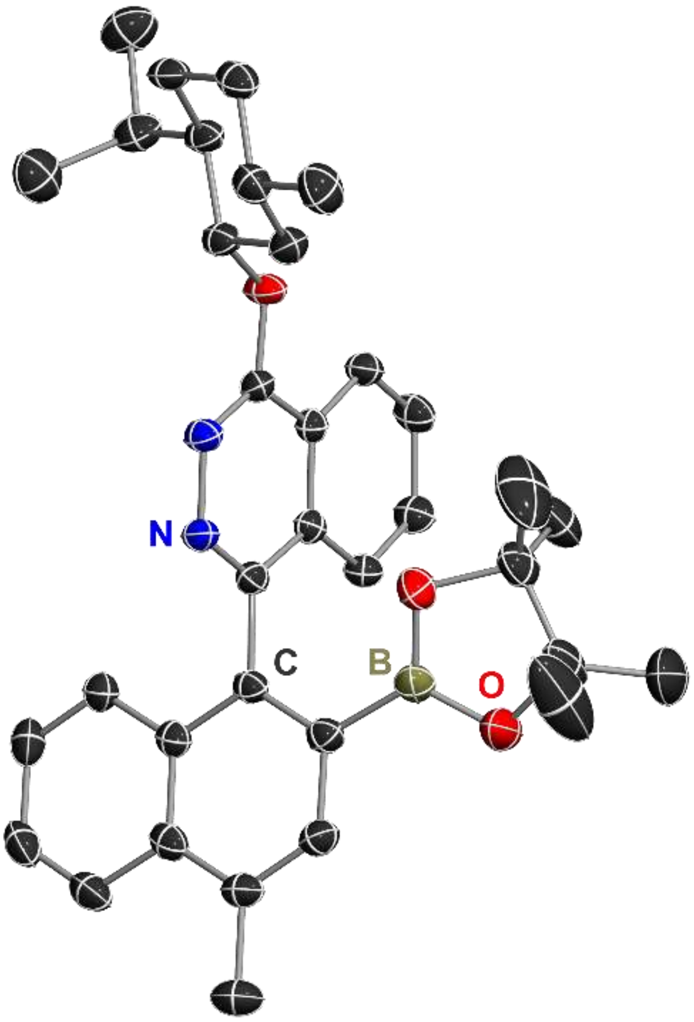

Table S4: Crystal data, data collection and structure refinement details for compound **5c**.

Compound	**5c**
Empirical formula	$C_{35}H_{43}BN_2O_3$
Formula weight [g/mol]	550.52
Colour	Yellow
Crystal size [mm]	0.29 × 0.23 × 0.17
Crystal system	Orthorhombic
Space group	$P2_12_12_1$
a [Å]	9.8775(3)
b [Å]	17.1809(5)
c [Å]	18.3778(6)
α [°]	90
β [°]	90
γ [°]	90
V [Å^3]	3118.79(17)
Z	4
D_{calc} [g/cm^3]	1.172
μ [mm^{-1}]	0.57
T [K]	150
θ range [°]	3.5-66.6
No. of reflections measured	43270
No. of independent reflections	5510
Obs. Reflections with $I > 2\sigma(I)$	5477
No. of Parameters refined/restraints	378/0
Absorption correction	multi-scan
T_{min}, T_{max}	0.85, 0.91
$\Delta\rho_{min}/\Delta\rho_{max}$ [e Å^{-3}]	−0.28/0.21
F(000)	1184
R_{int}	0.029
R_1 ($R[F^2 \geq 2\sigma(F^2)]$)	0.034
wR_2 ($wR(F^2)$)	0.088
GooF	1.04
Flack parameter	−0.02(4)

7. NMR Spectra

^{1}H NMR ($CDCl_3$, 300 Hz) of (**+**)-**2a**

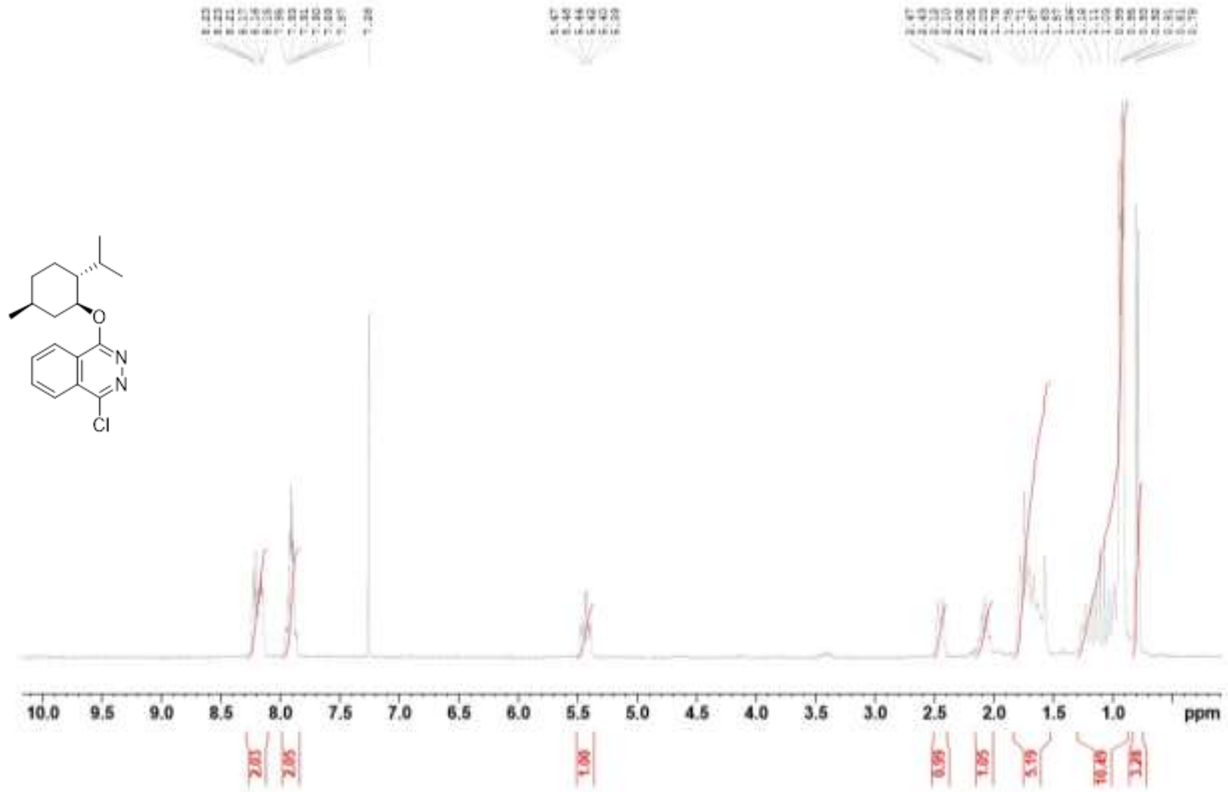

^{13}C{^{1}H} NMR ($CDCl_3$, 125 Hz) of (**+**)-**2a**

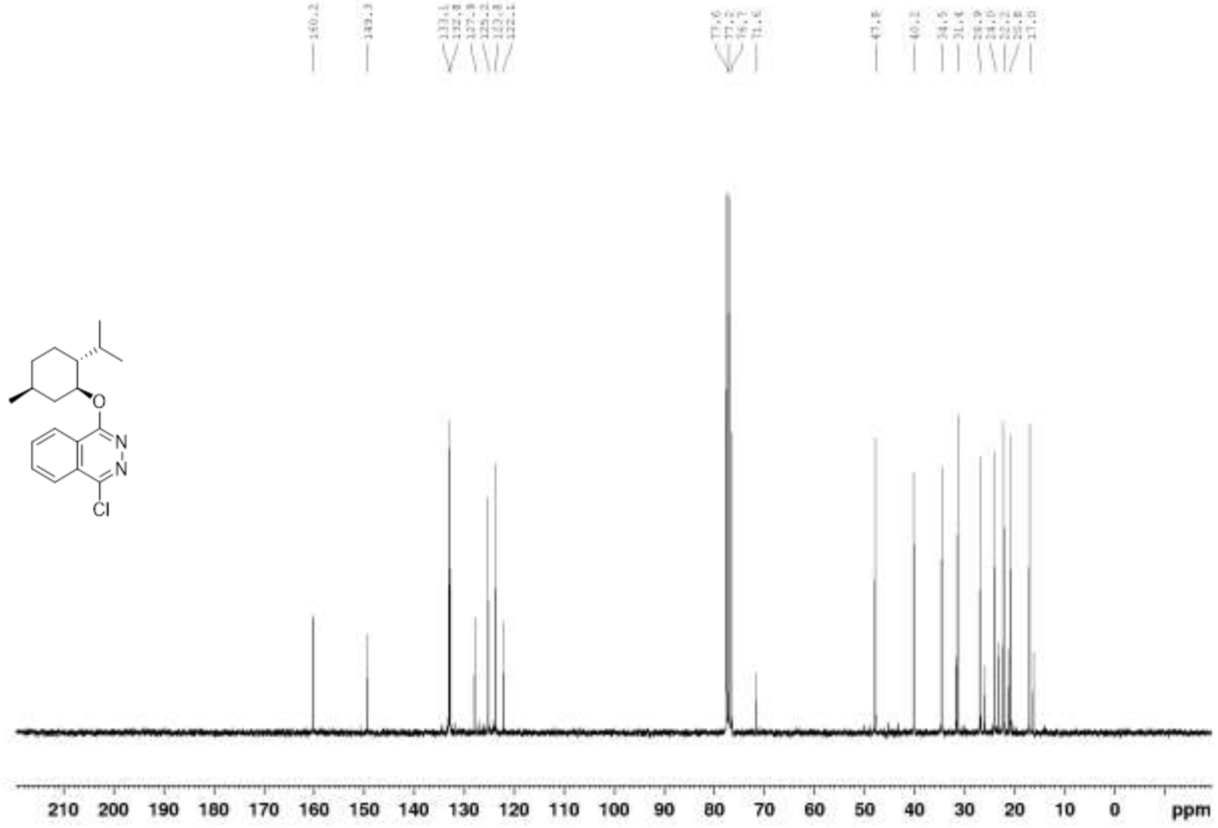

^{1}H NMR ($CDCl_3$, 300 Hz) of (**-**)-**2a**

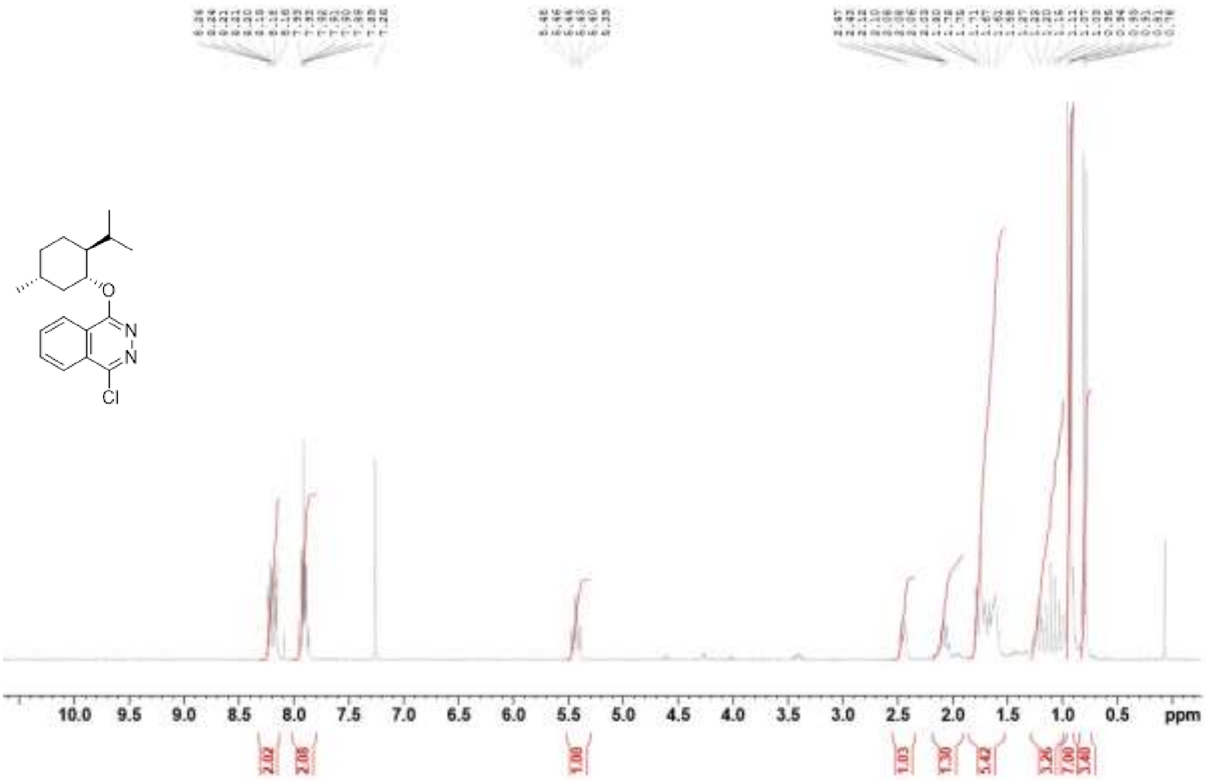

$^{13}C\{^{1}H\}$ NMR ($CDCl_3$, 125 Hz) of (**-**)-**2a**

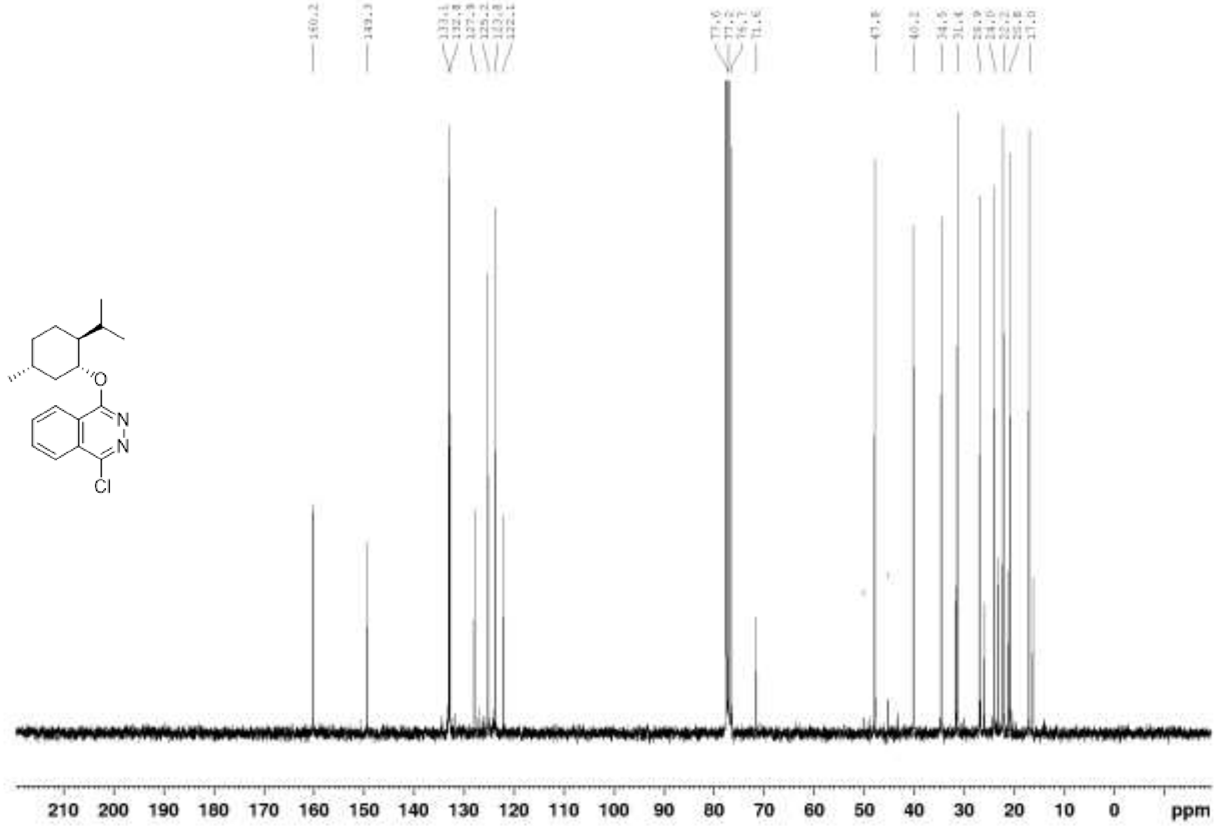

^{1}H NMR ($CDCl_3$, 300 Hz) of (**+**)-**3a**

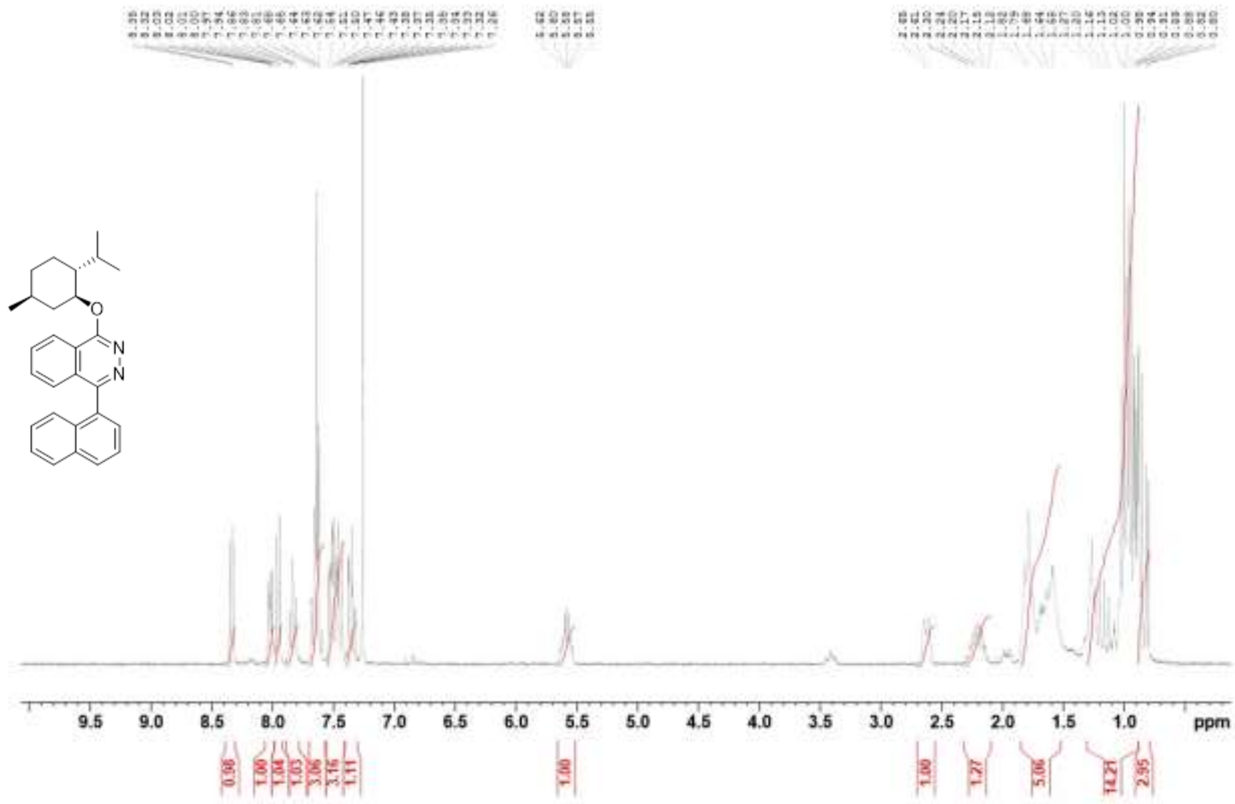

^{13}C{^{1}H} NMR ($CDCl_3$, 125 Hz) of (**+**)-**3a**

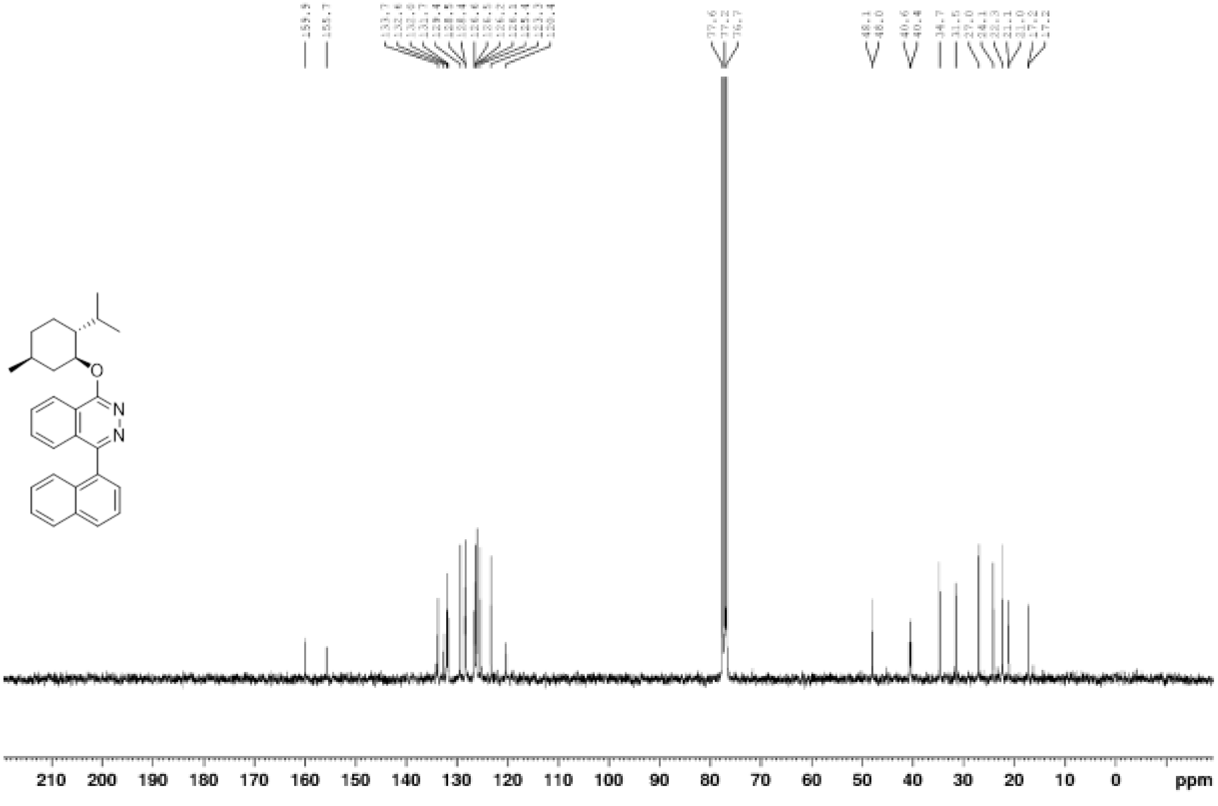

^{1}H NMR ($CDCl_3$, 300 Hz) of **(+)-3b**

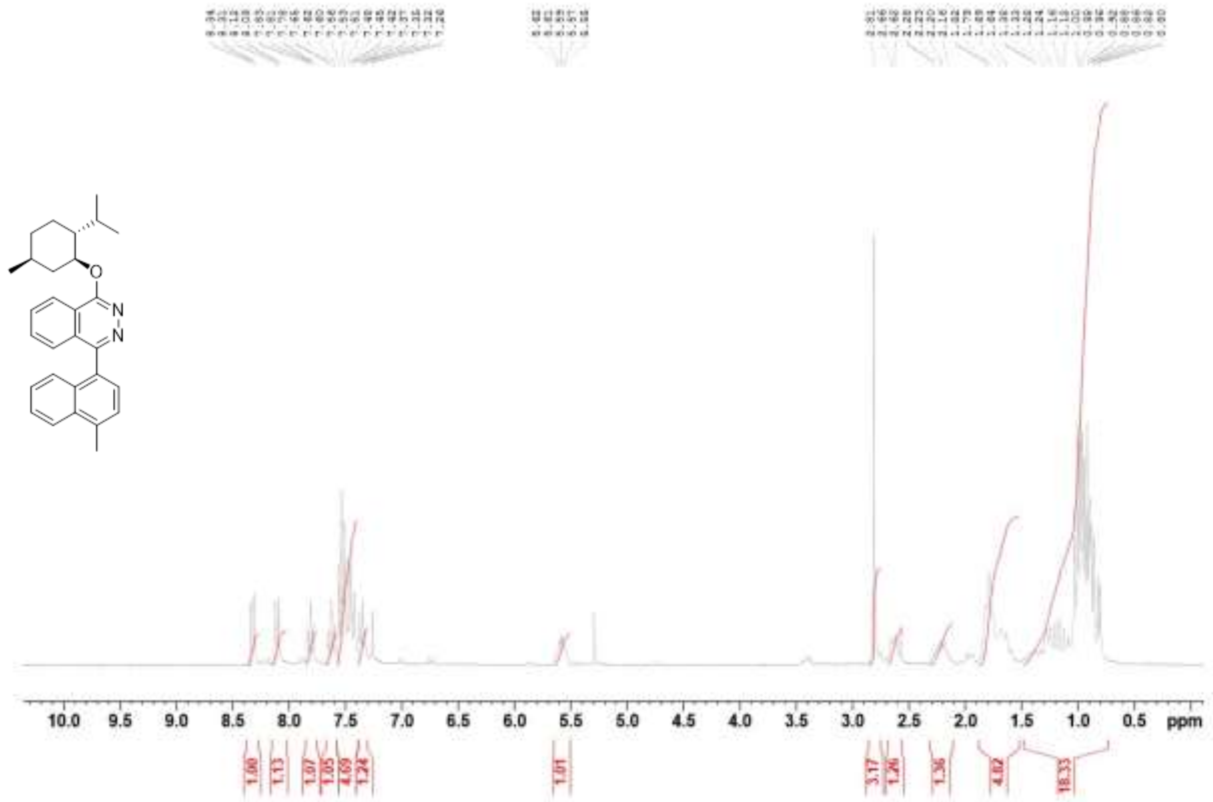

^{13}C{^{1}H} NMR ($CDCl_3$, 125 Hz) of (**+**)-**3b**

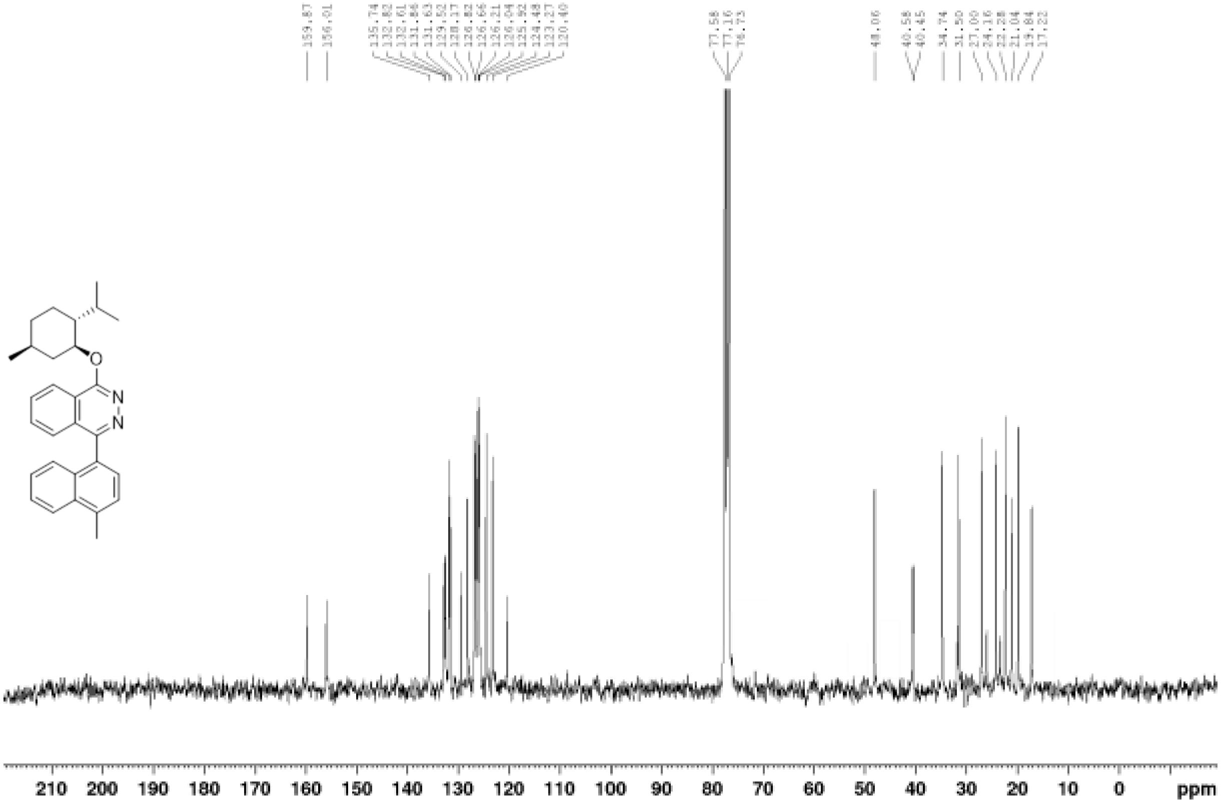

^{1}H NMR ($CDCl_3$, 300 Hz) of **3c**

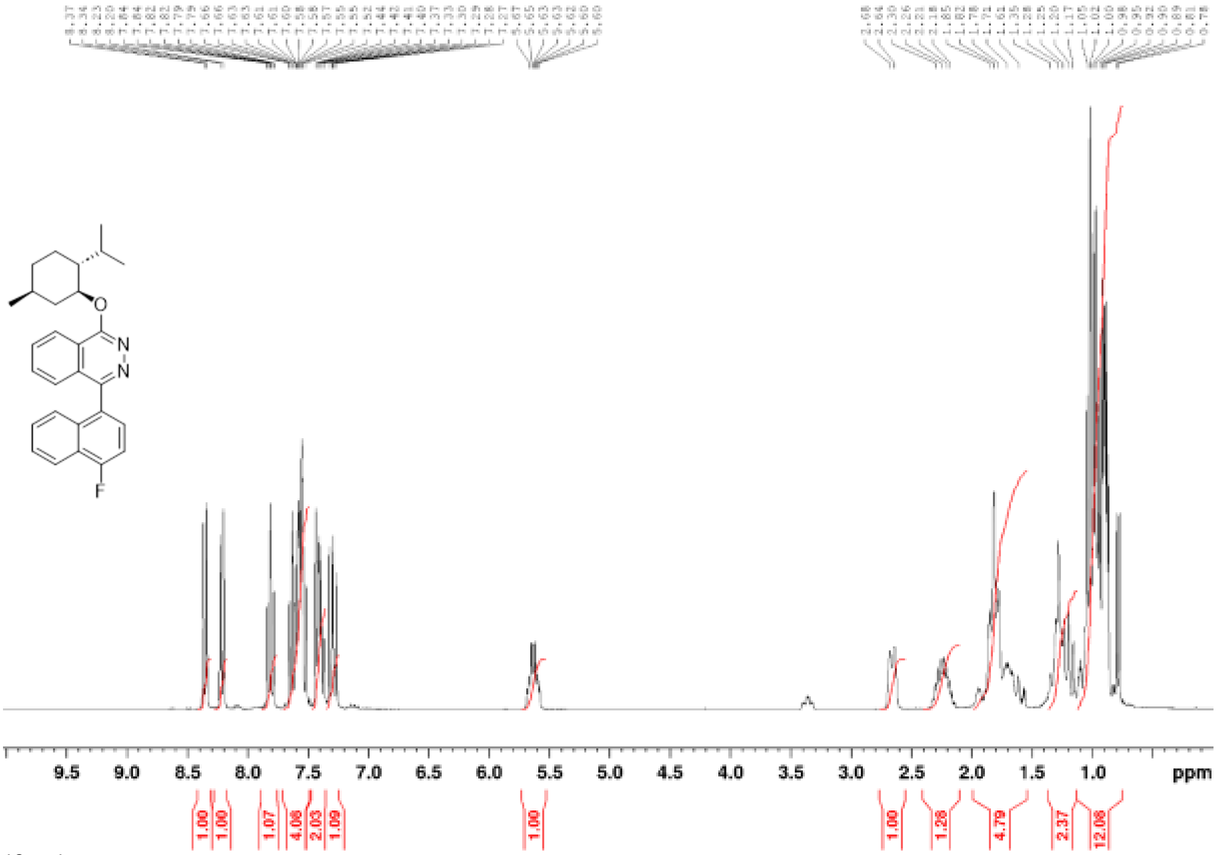

^{13}C{^{1}H} NMR ($CDCl_3$, 125 Hz) of **3c**

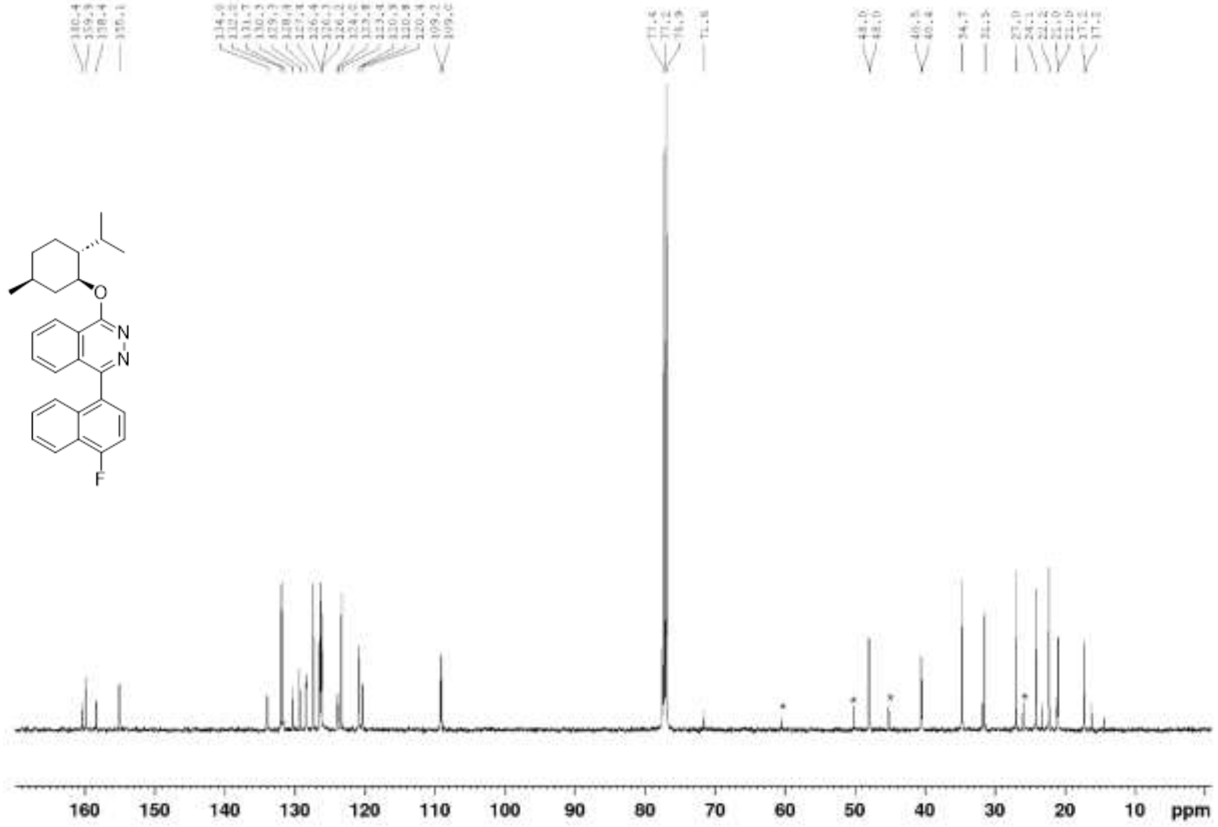

Peaks marked with * indicate impurities stemming from inseparable menthol.

^{19}F NMR ($CDCl_3$, 282 Hz) of **3c**

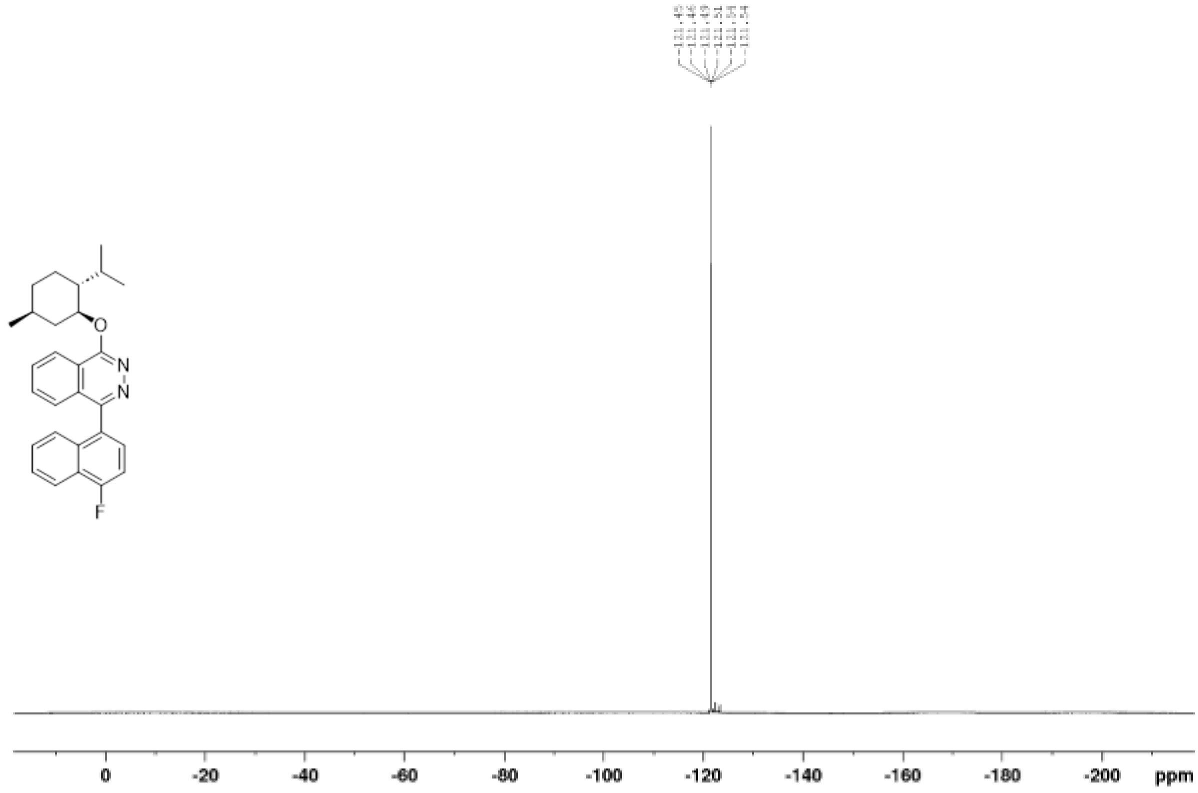

^{1}H NMR ($CDCl_3$, 300 Hz) of **3d**

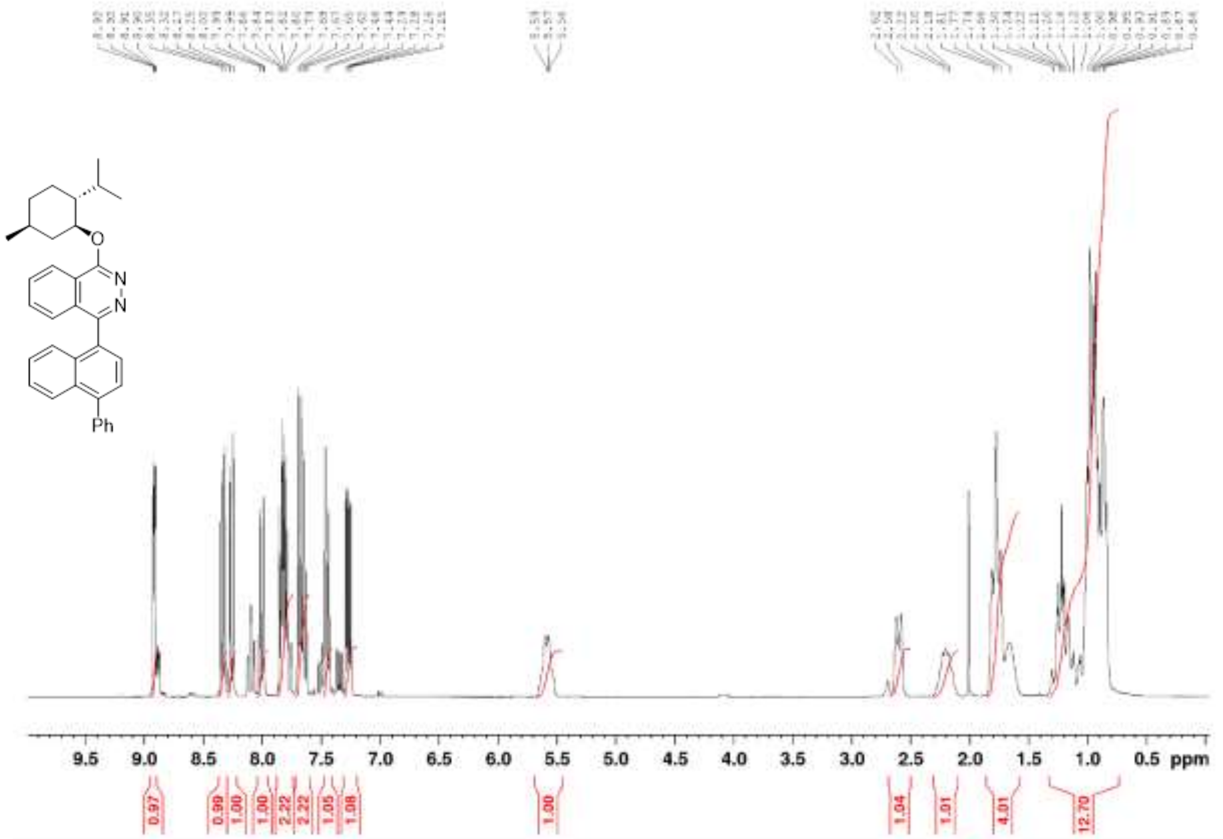

^{13}C{^{1}H} NMR ($CDCl_3$, 125 Hz) of **3d**

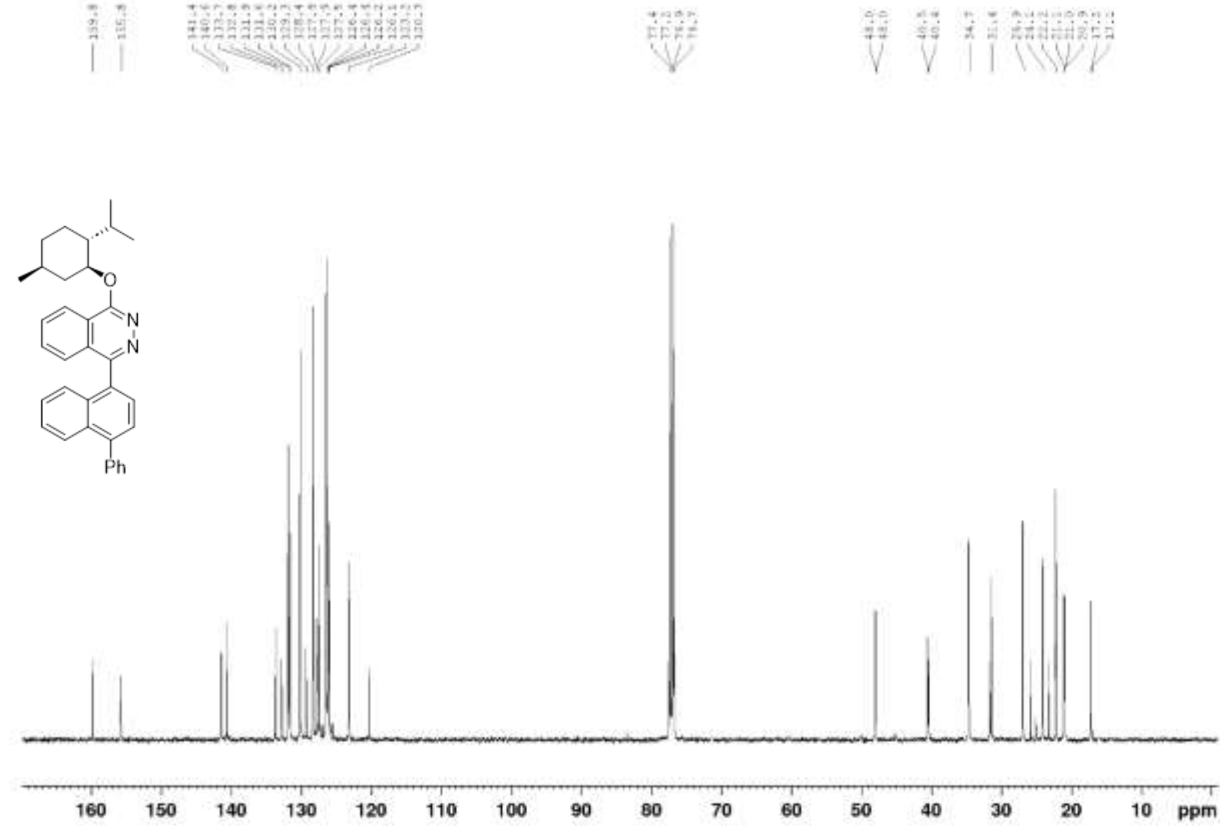

^{1}H NMR ($CDCl_3$, 300 Hz) of (**+**)-**3e**

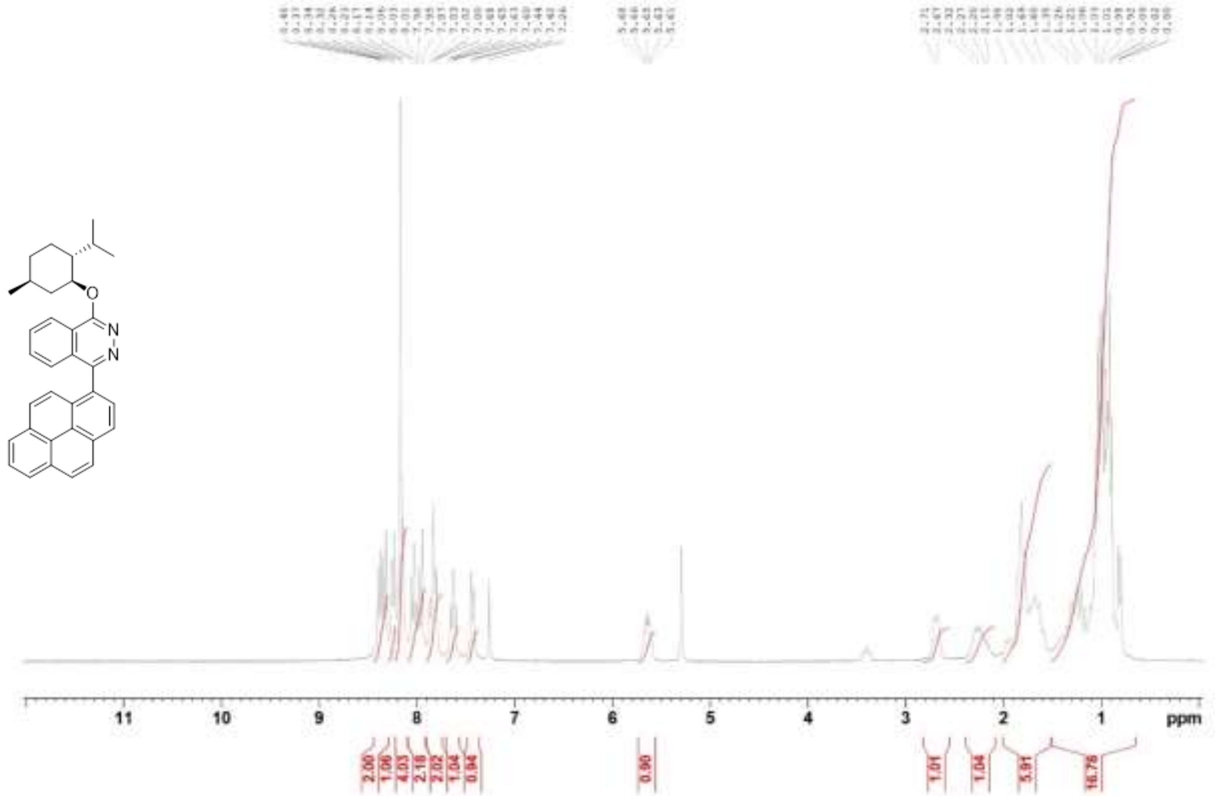

$^{13}C\{^{1}H\}$ NMR ($CDCl_3$, 125 Hz) of (**+**)-**3e**

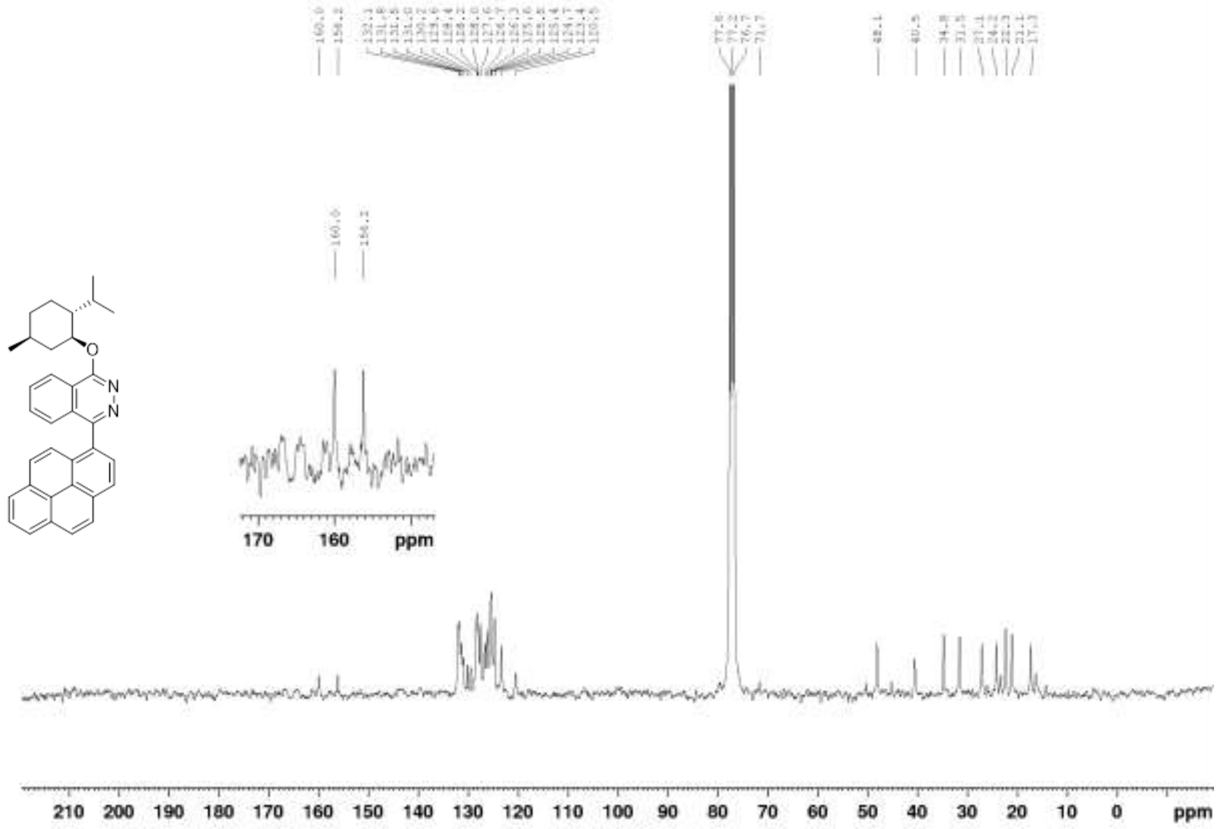

^{1}H NMR ($CDCl_3$, 300 Hz) of **3f**

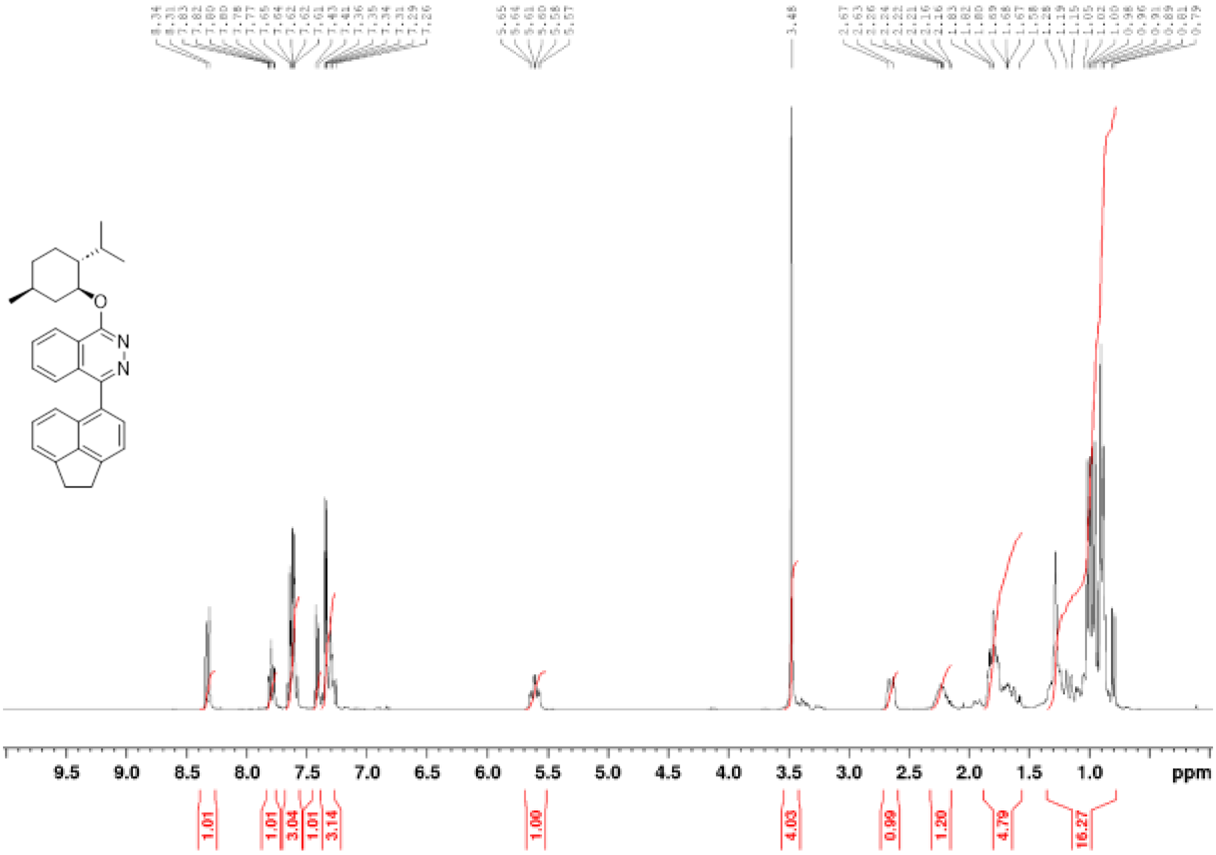

$^{13}C\{^{1}H\}$ NMR ($CDCl_3$, 125 Hz) of **3f**

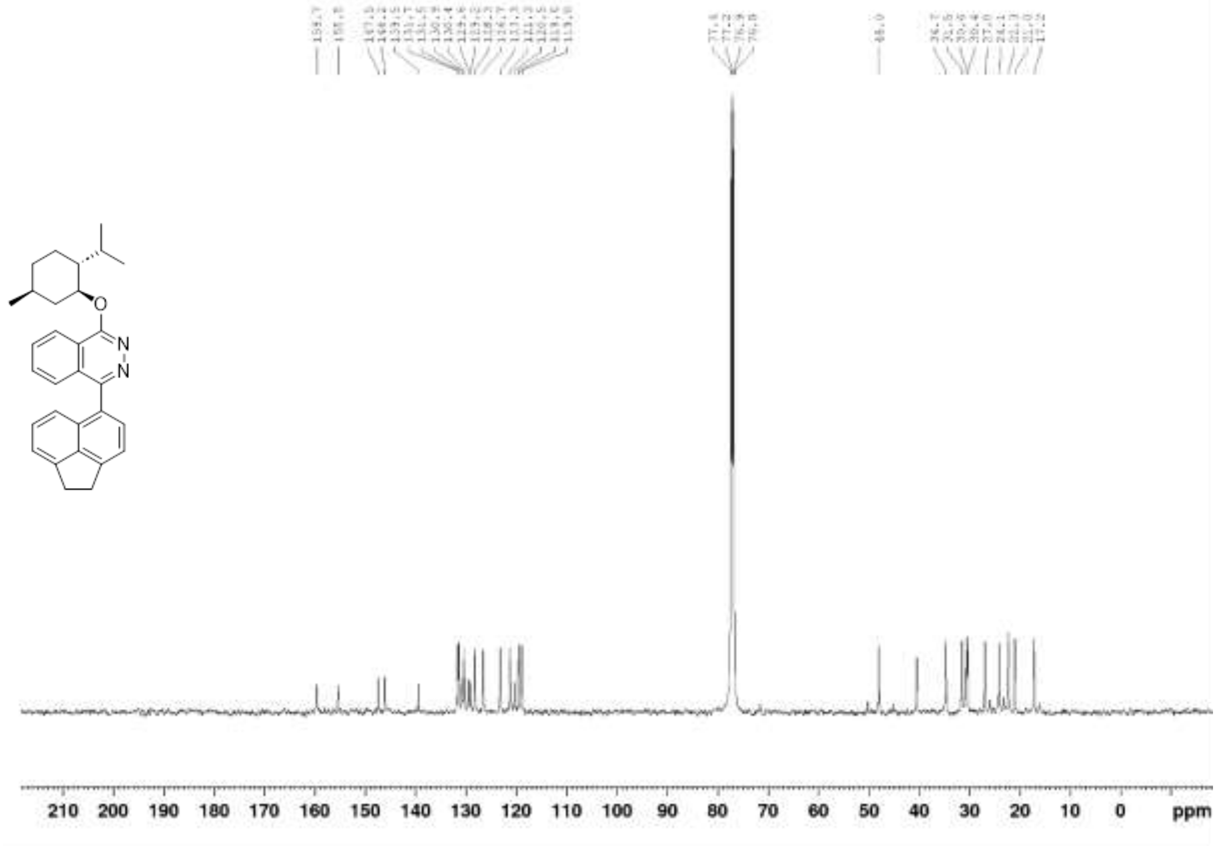

^{1}H NMR ($CDCl_3$, 300 Hz) of **3g**

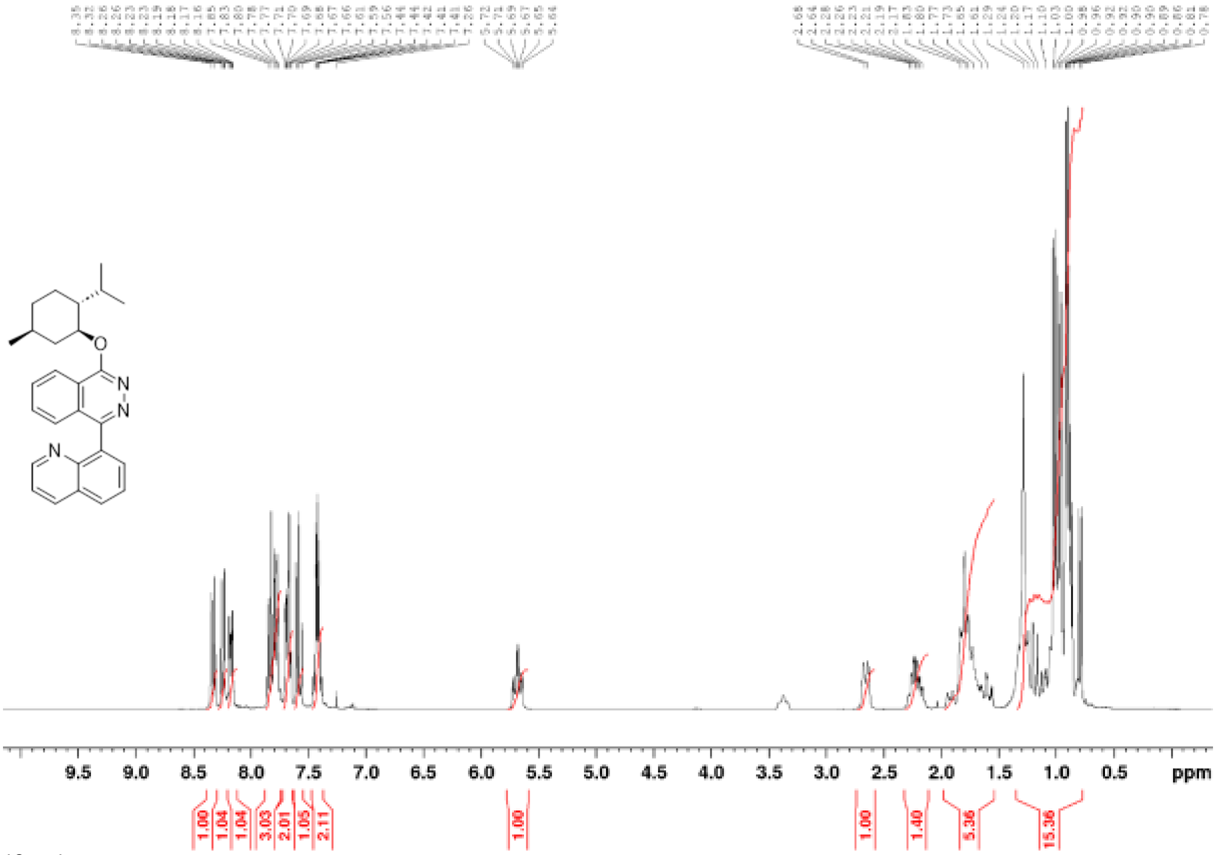

^{13}C{^{1}H} NMR ($CDCl_3$, 125 Hz) of **3g**

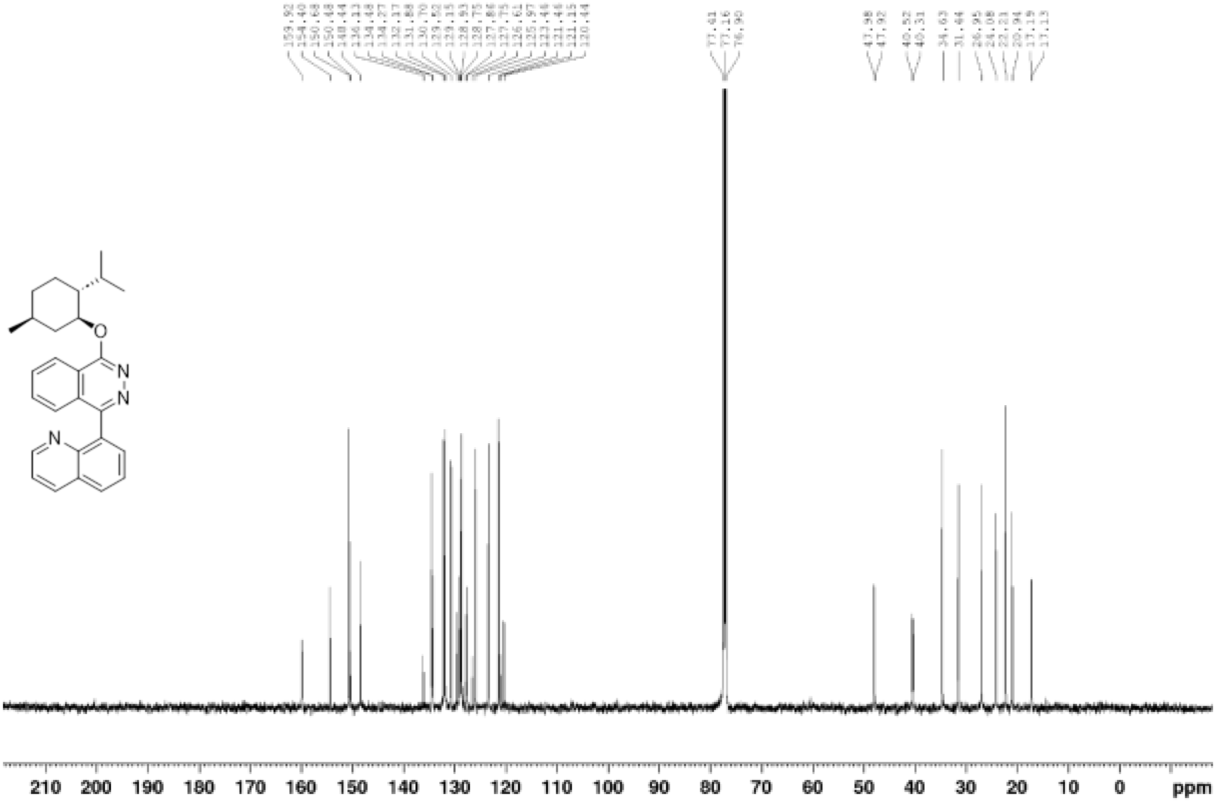

^{1}H NMR ($CDCl_3$, 300 Hz) of **3h**

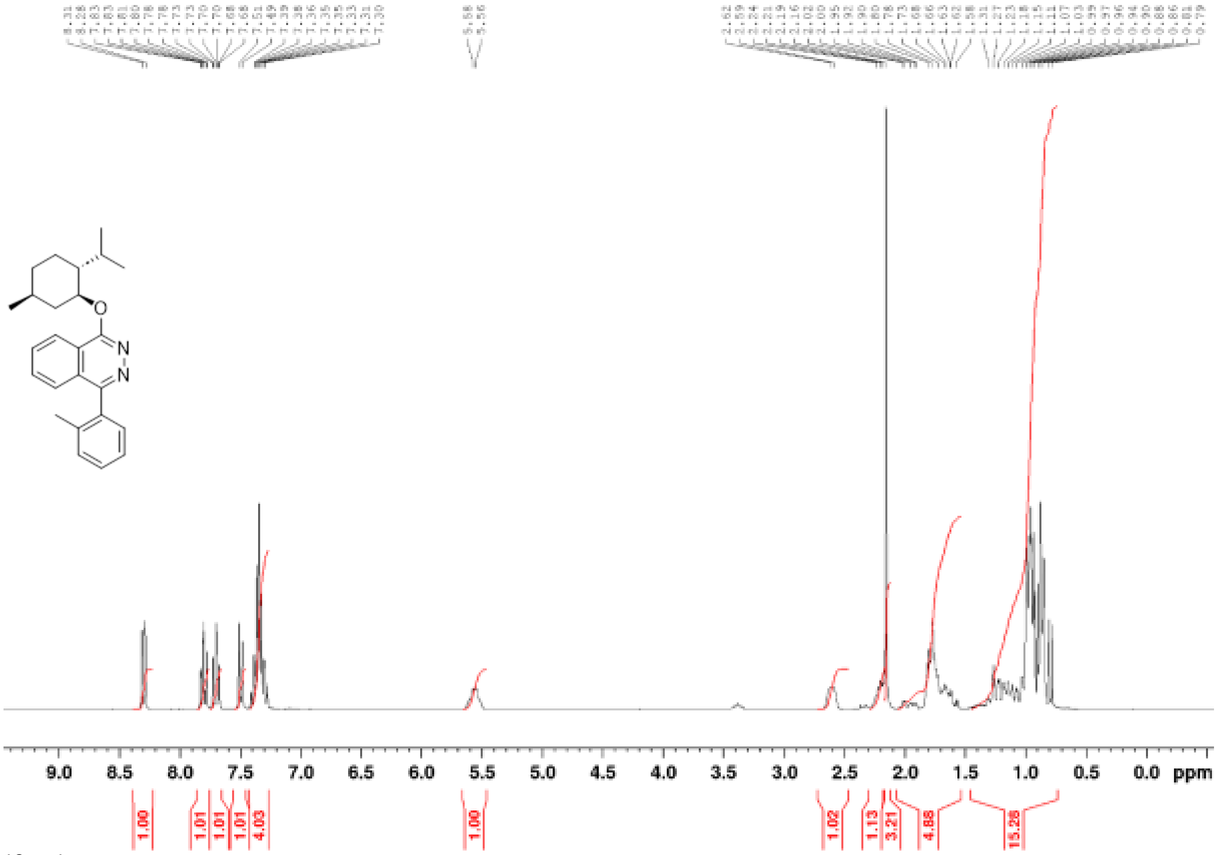

^{13}C{^{1}H} NMR ($CDCl_3$, 125 Hz) of **3h**

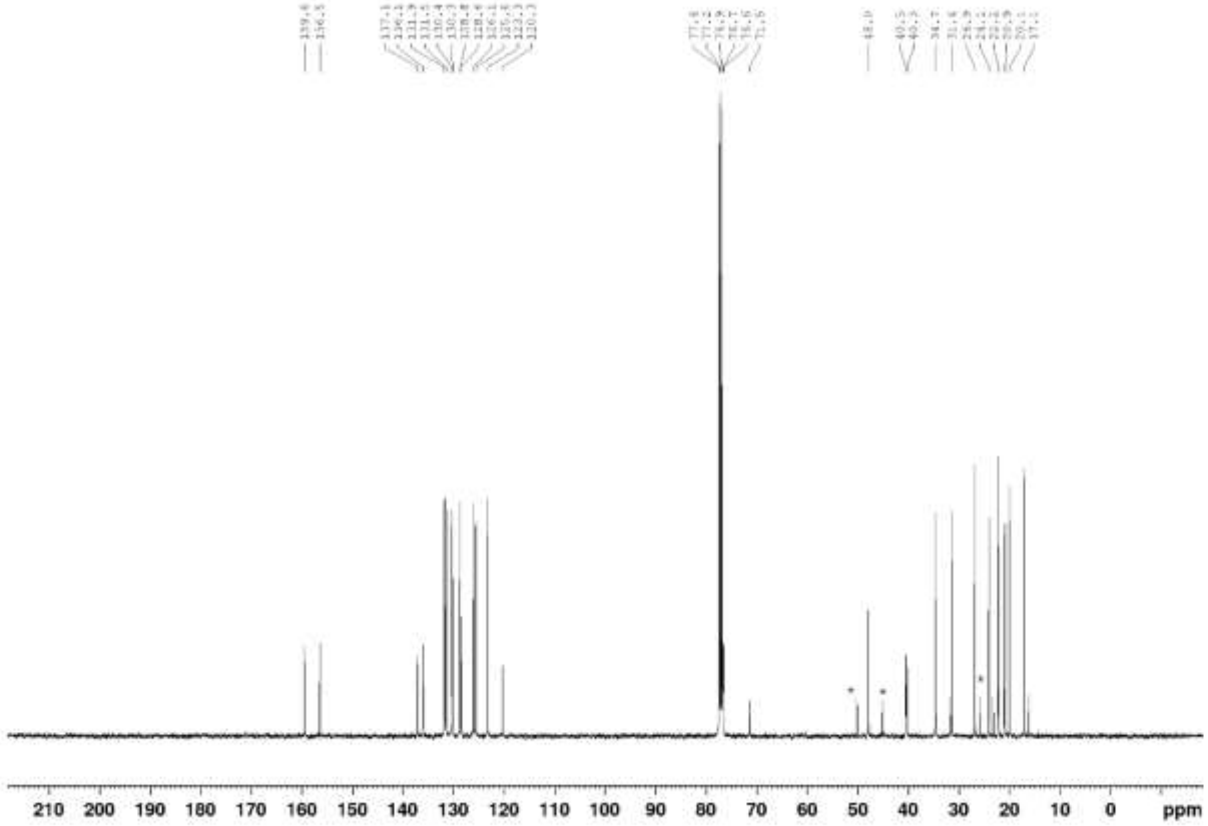

Peaks marked with * indicate impurities stemming from inseparable menthol.

^{1}H NMR ($CDCl_3$, 300 Hz) of **3i**

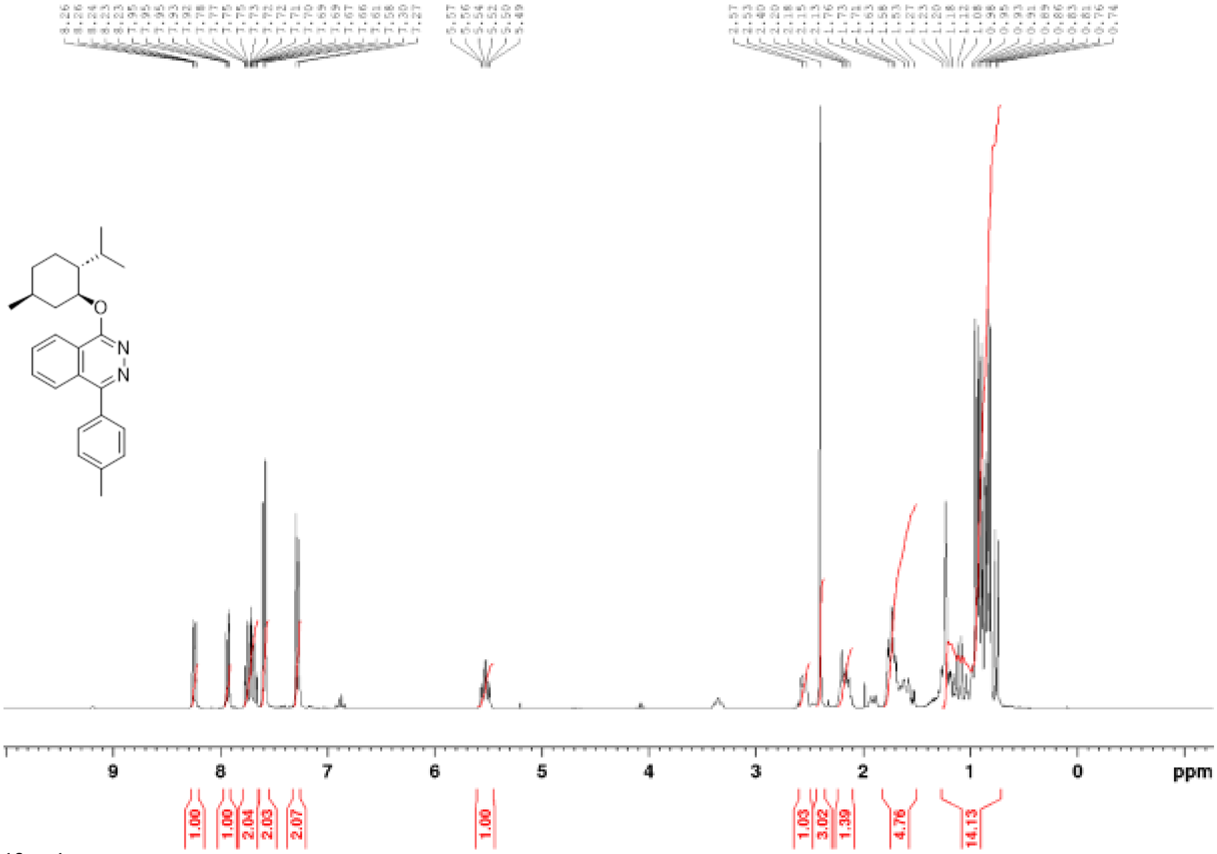

$^{13}C\{^{1}H\}$ NMR ($CDCl_3$, 125 Hz) of **3i**

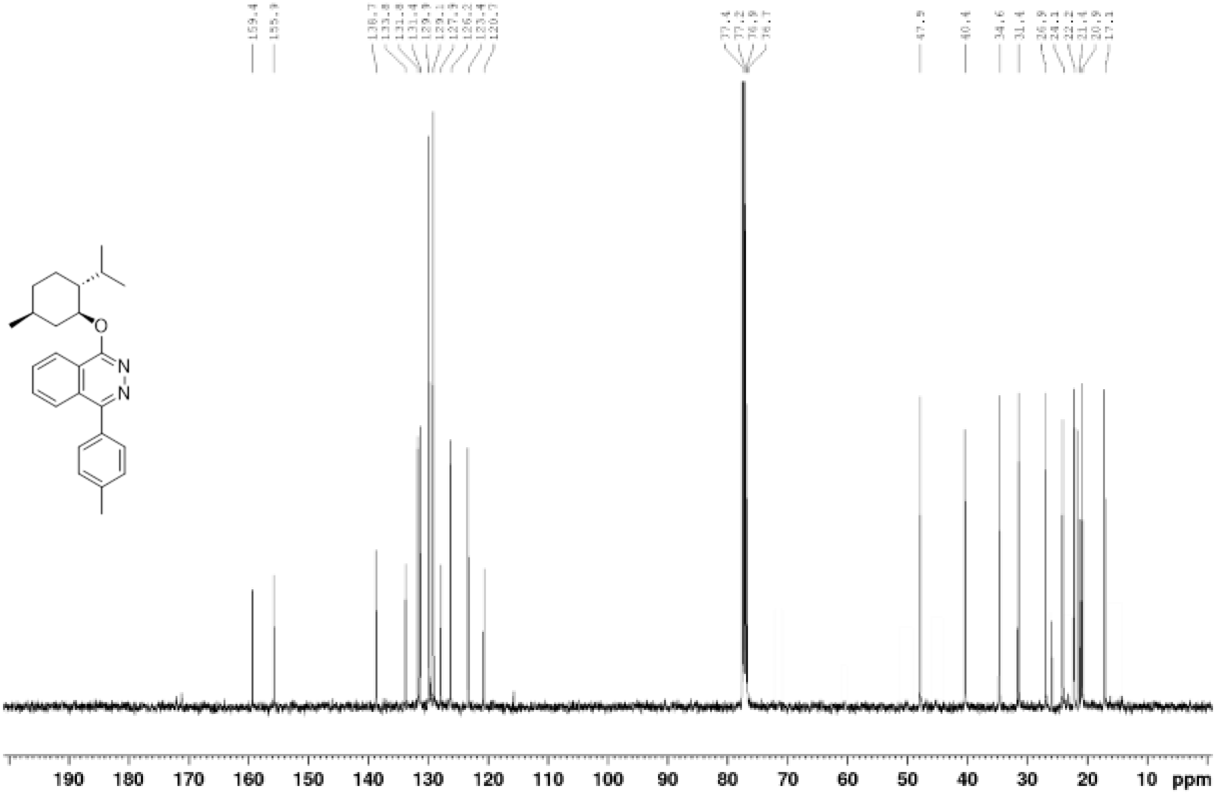

^{1}H NMR ($CDCl_3$, 300 Hz) of **3j**

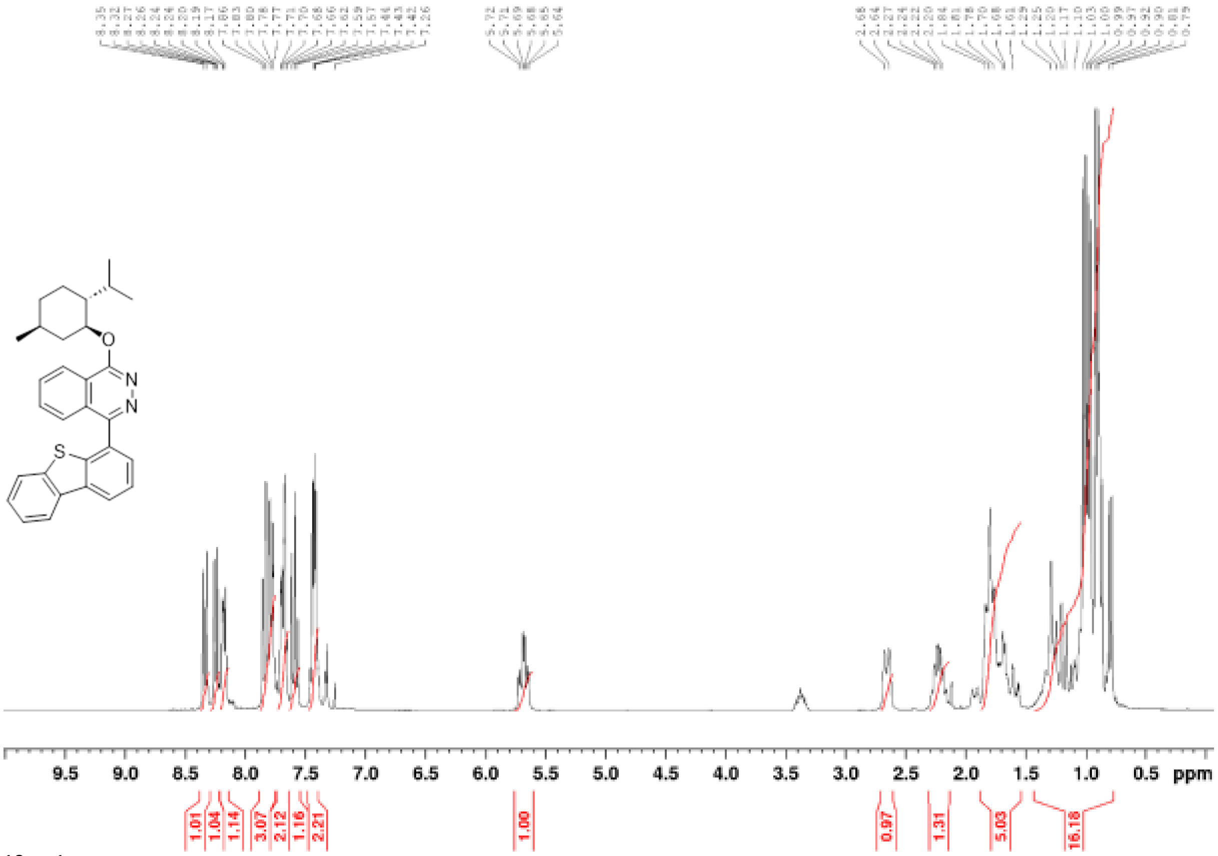

^{13}C{^{1}H} NMR ($CDCl_3$, 125 Hz) of **3j**

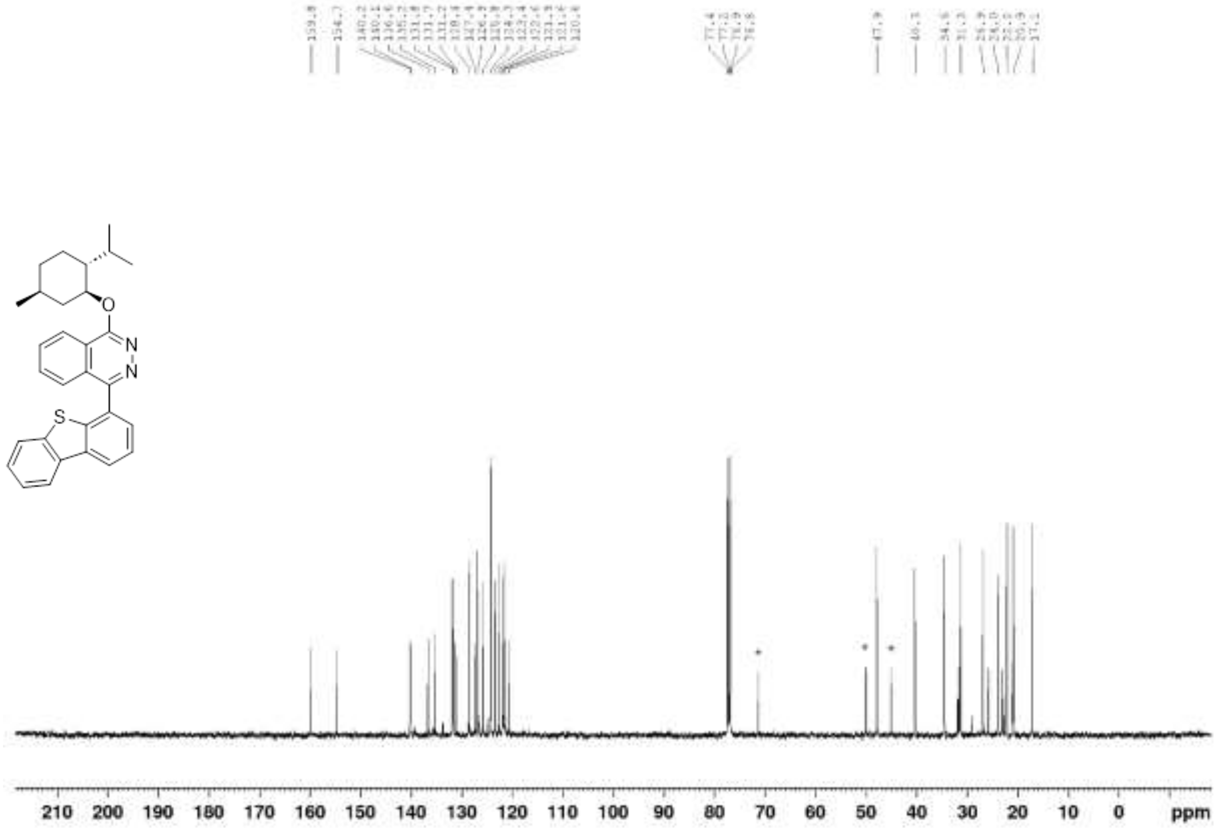

Peaks marked with * indicate impurities stemming from inseparable menthol.

^{1}H NMR ($CDCl_3$, 300 Hz) of (**+**)-**3k**

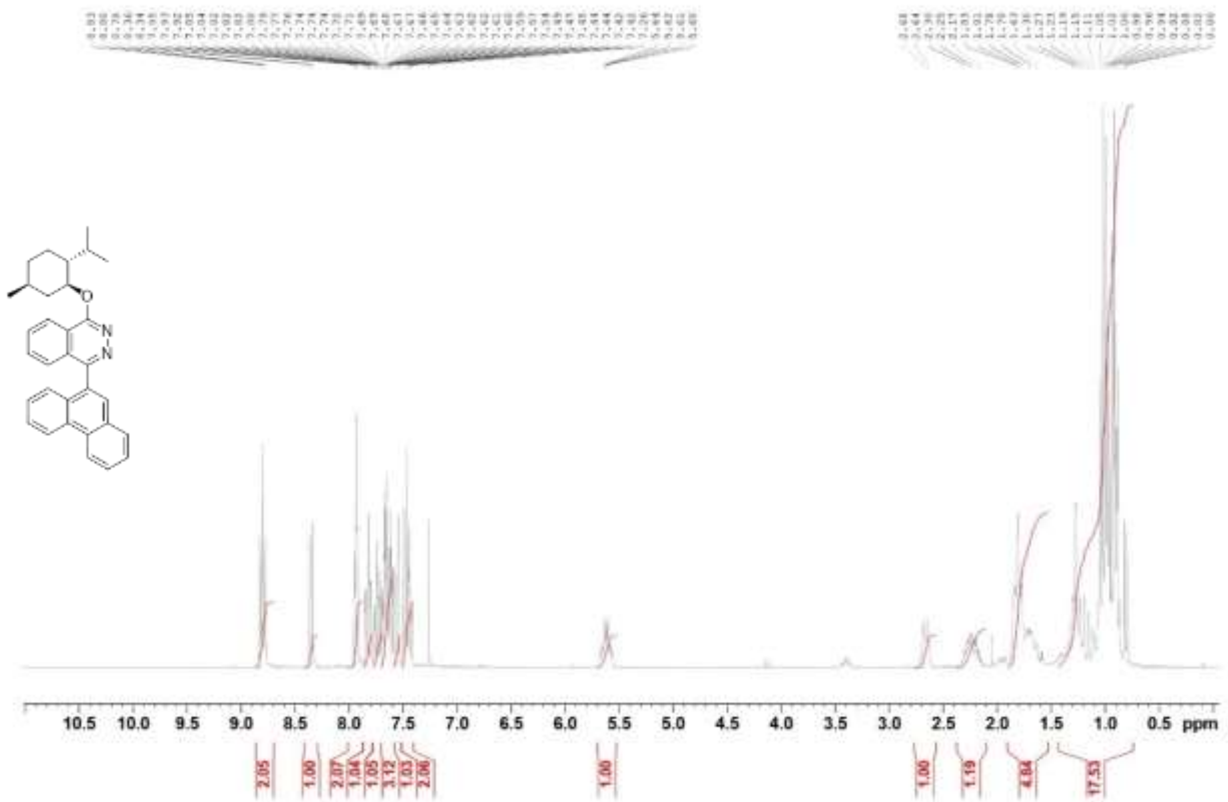

^{13}C{^{1}H} NMR ($CDCl_3$, 125 Hz) of (**+**)-**3k**

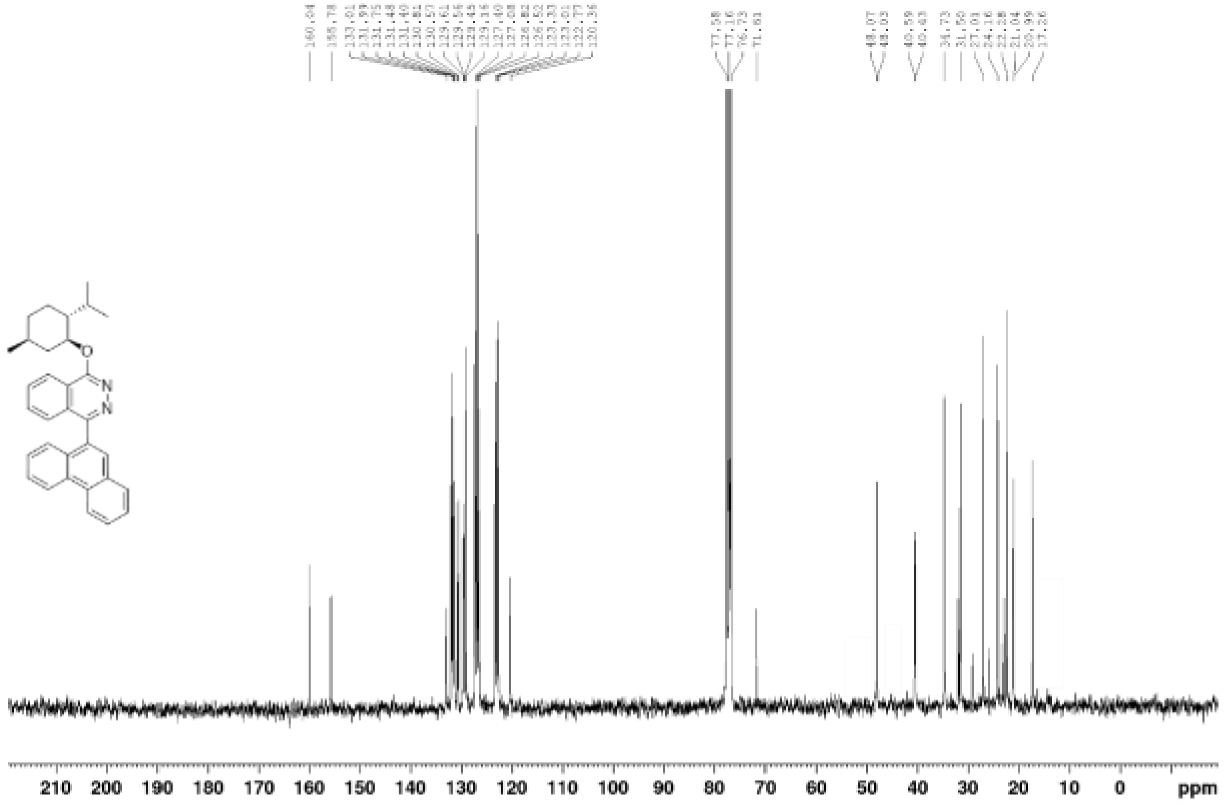

^{1}H NMR ($CDCl_3$, 300 Hz) of (**-**)-**3a**

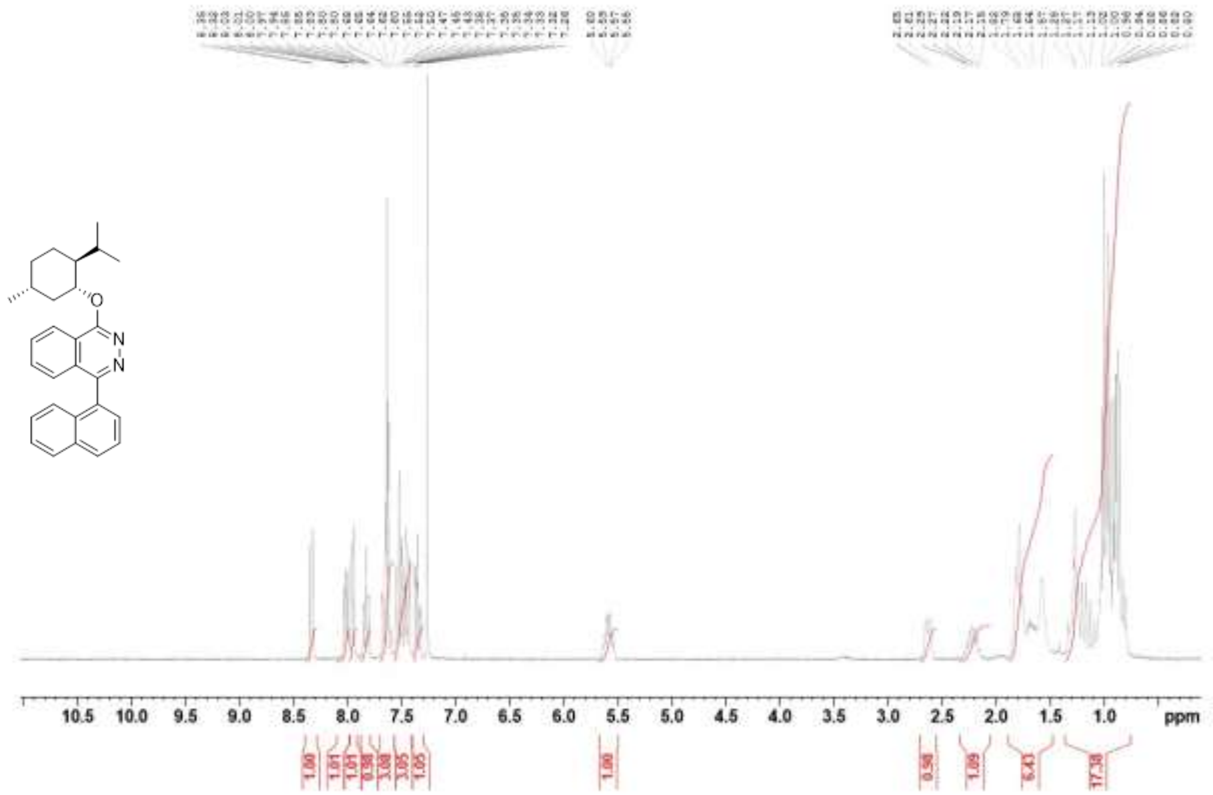

$^{13}C\{^{1}H\}$ NMR ($CDCl_3$, 125 Hz) of (**-**)-**3a**

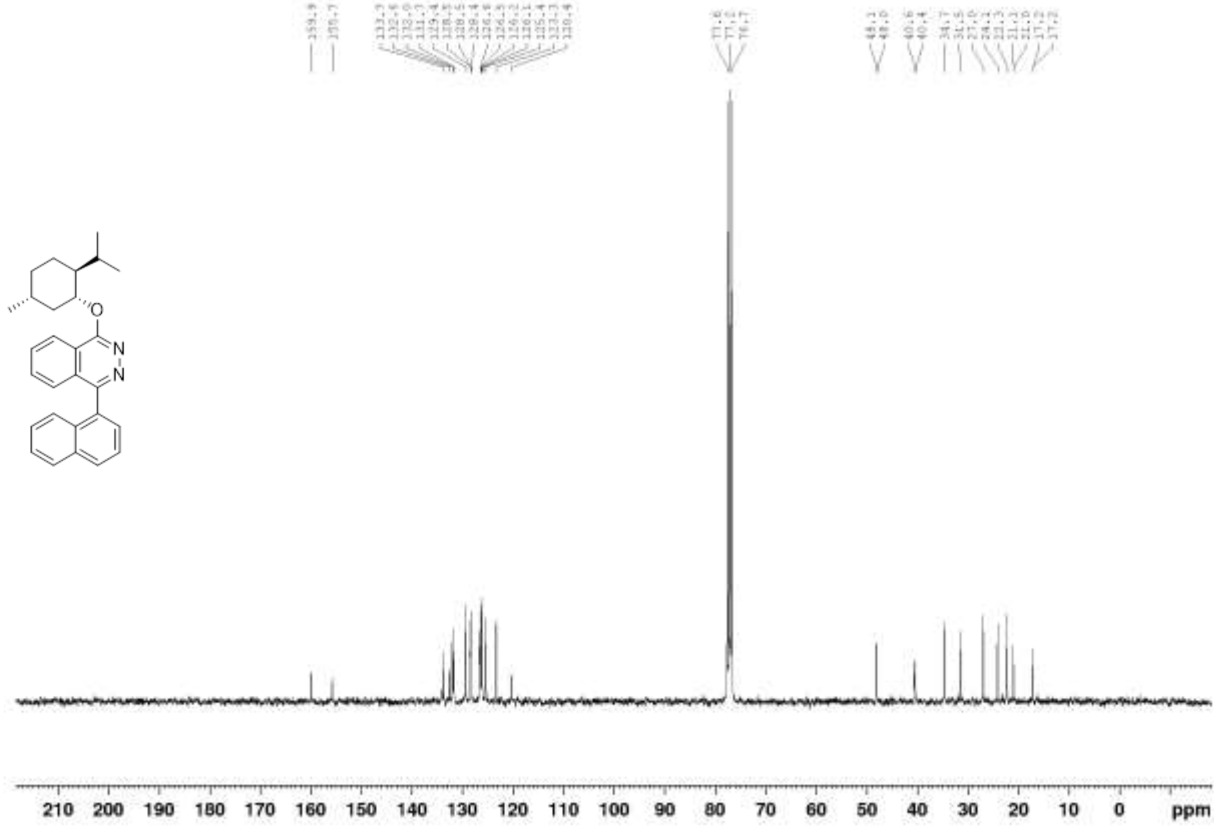

^{1}H NMR ($CDCl_3$, 300 Hz) of (**-**)-**3b**

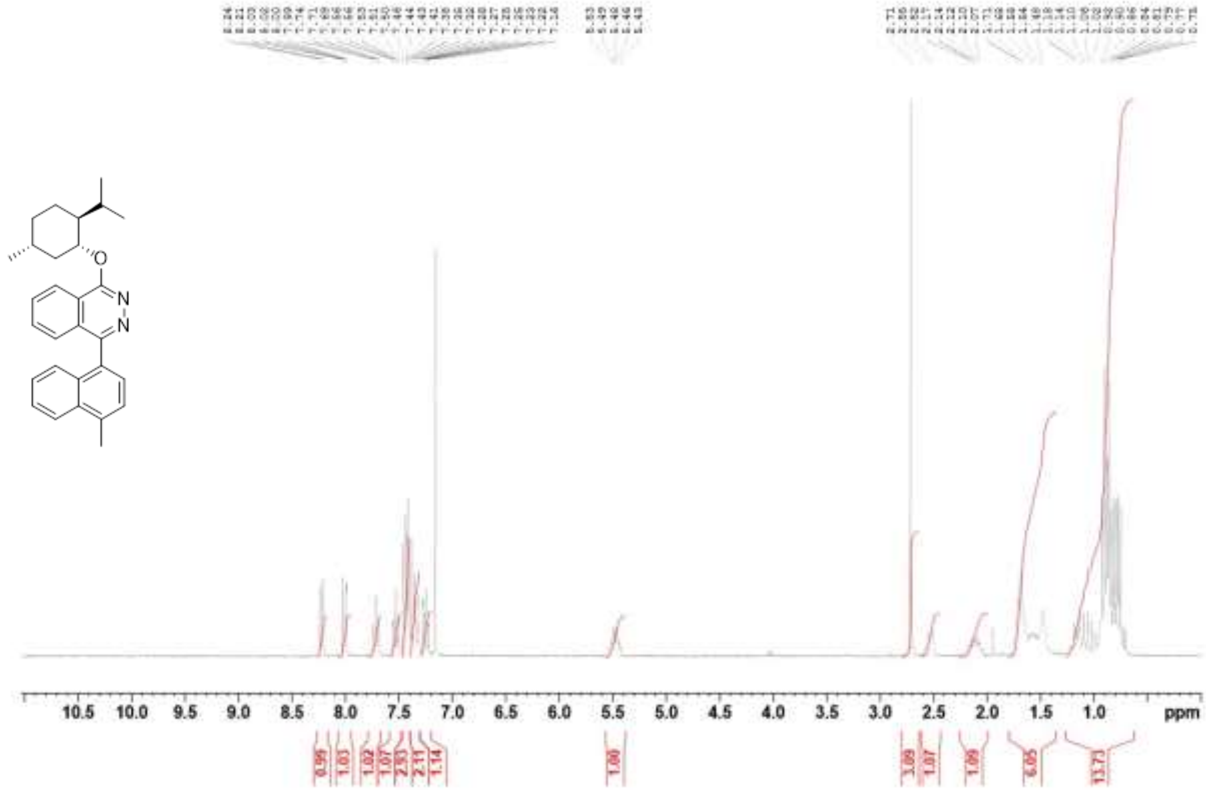

^{13}C{^{1}H} NMR ($CDCl_3$, 125 Hz) of (**-**)-**3b**

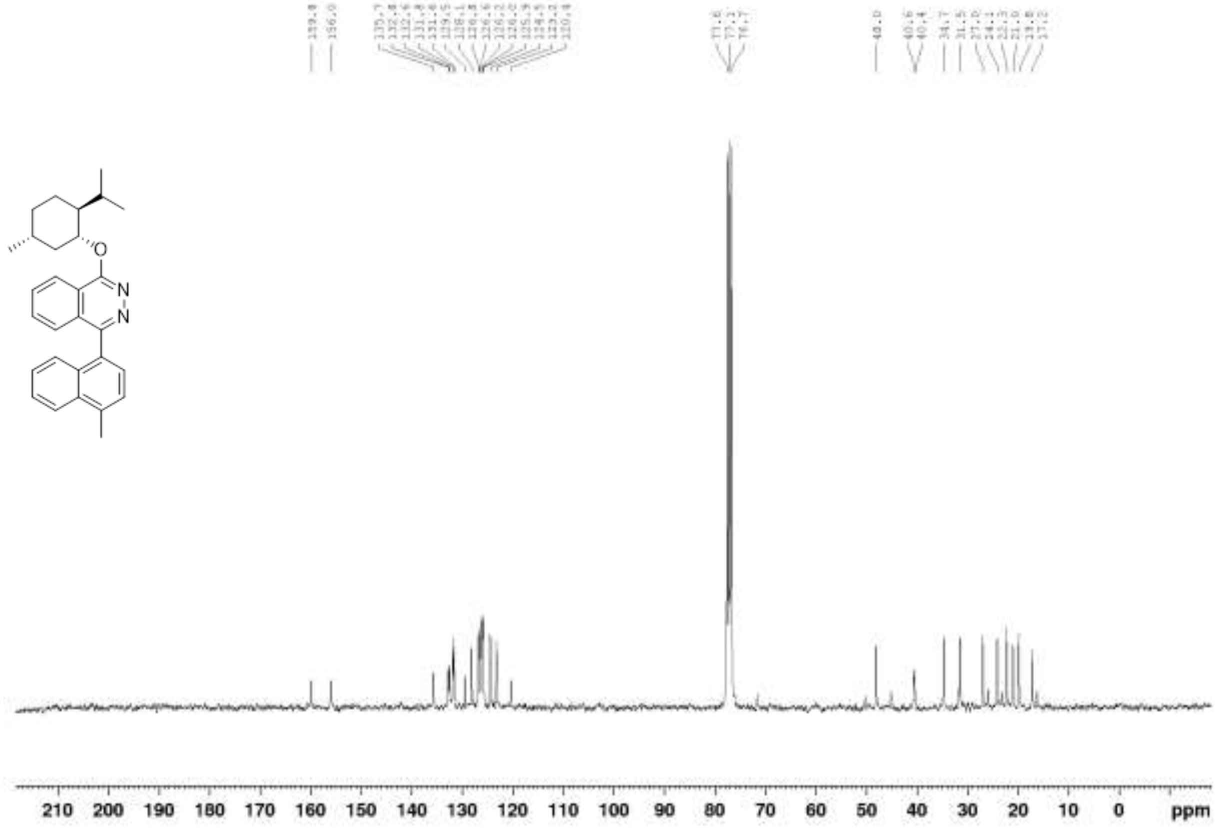

^{1}H NMR ($CDCl_3$, 300 Hz) of (**-**)-**3e**

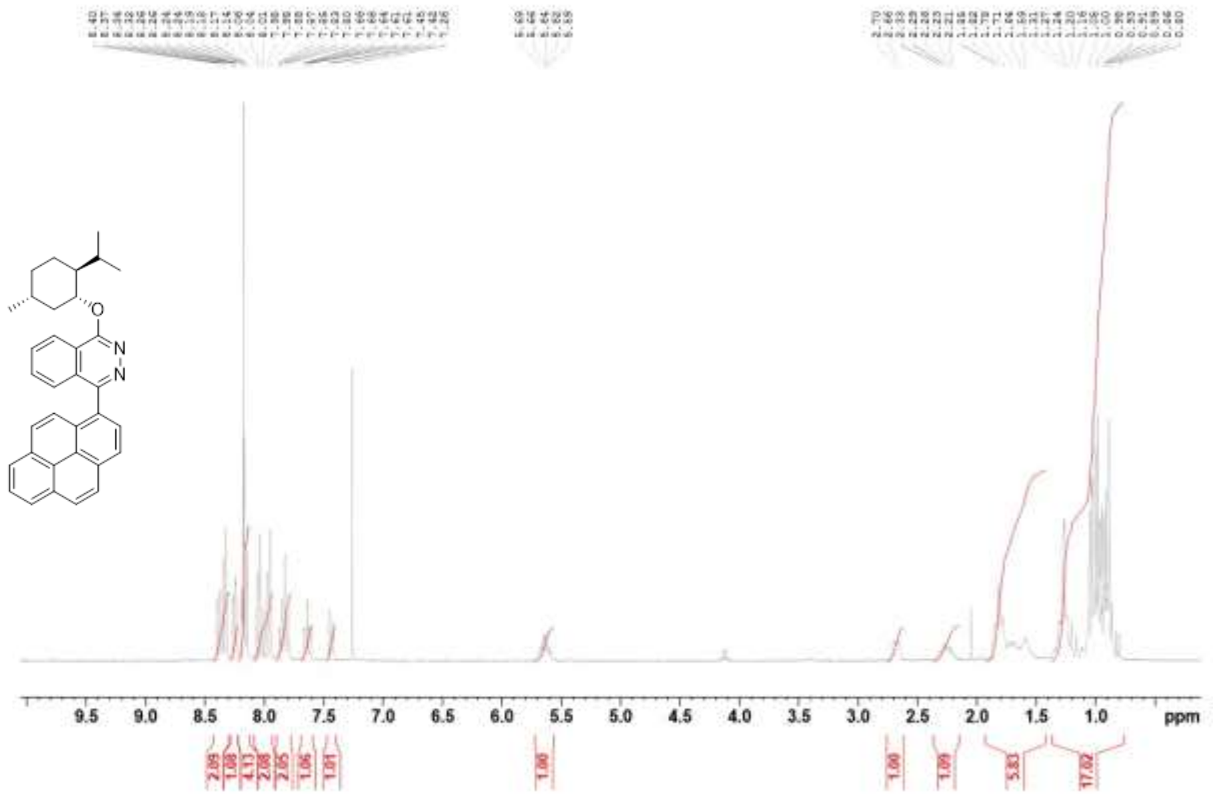

$^{13}C\{^{1}H\}$ -NMR ($CDCl_3$, 125 Hz) of (**-**)-**3e**

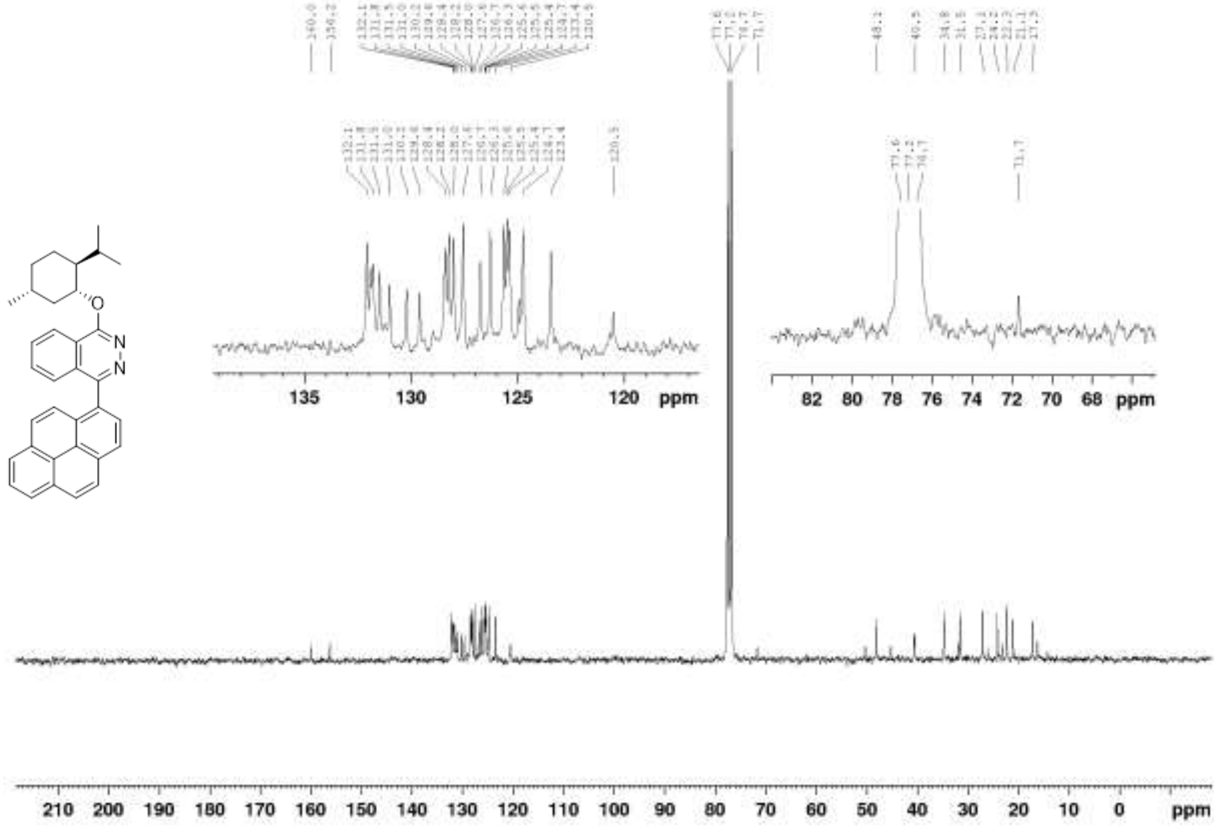

^{1}H NMR ($CDCl_3$, 300 Hz) of **4a**

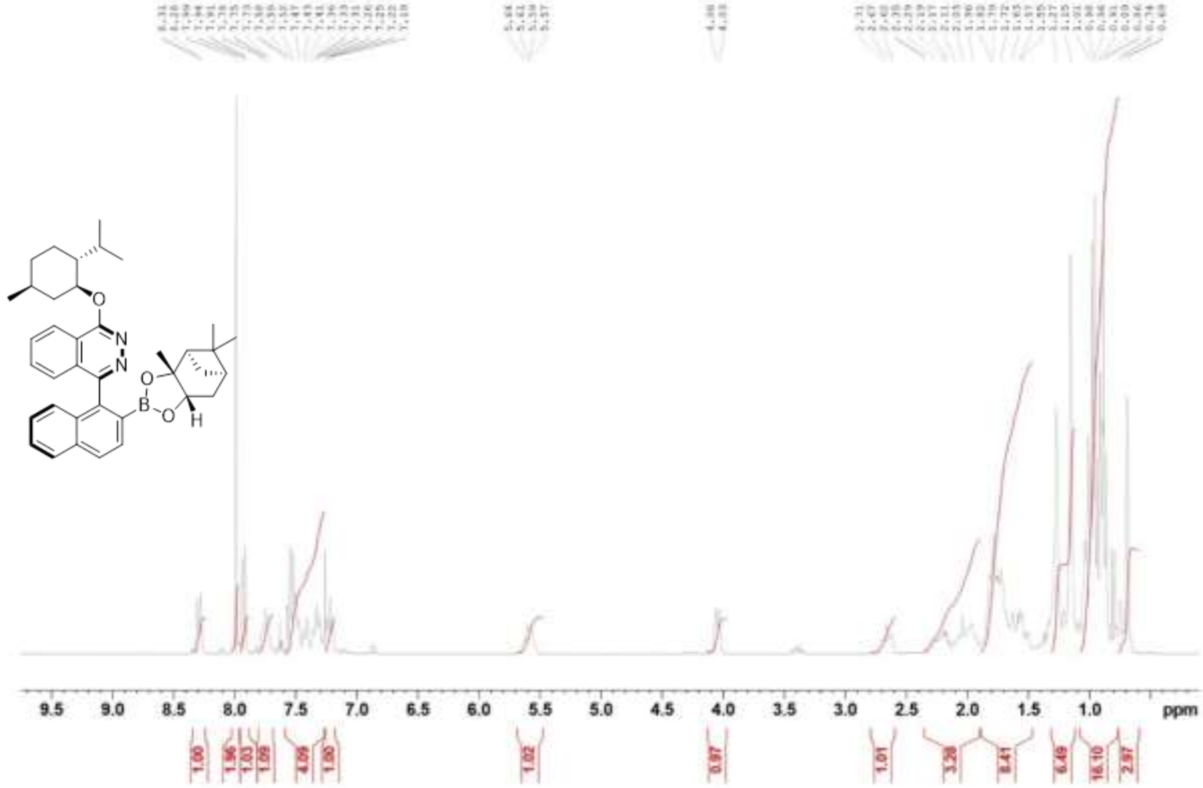

$^{13}C\{^{1}H\}$ NMR ($CDCl_3$, 125 Hz) of **4a**

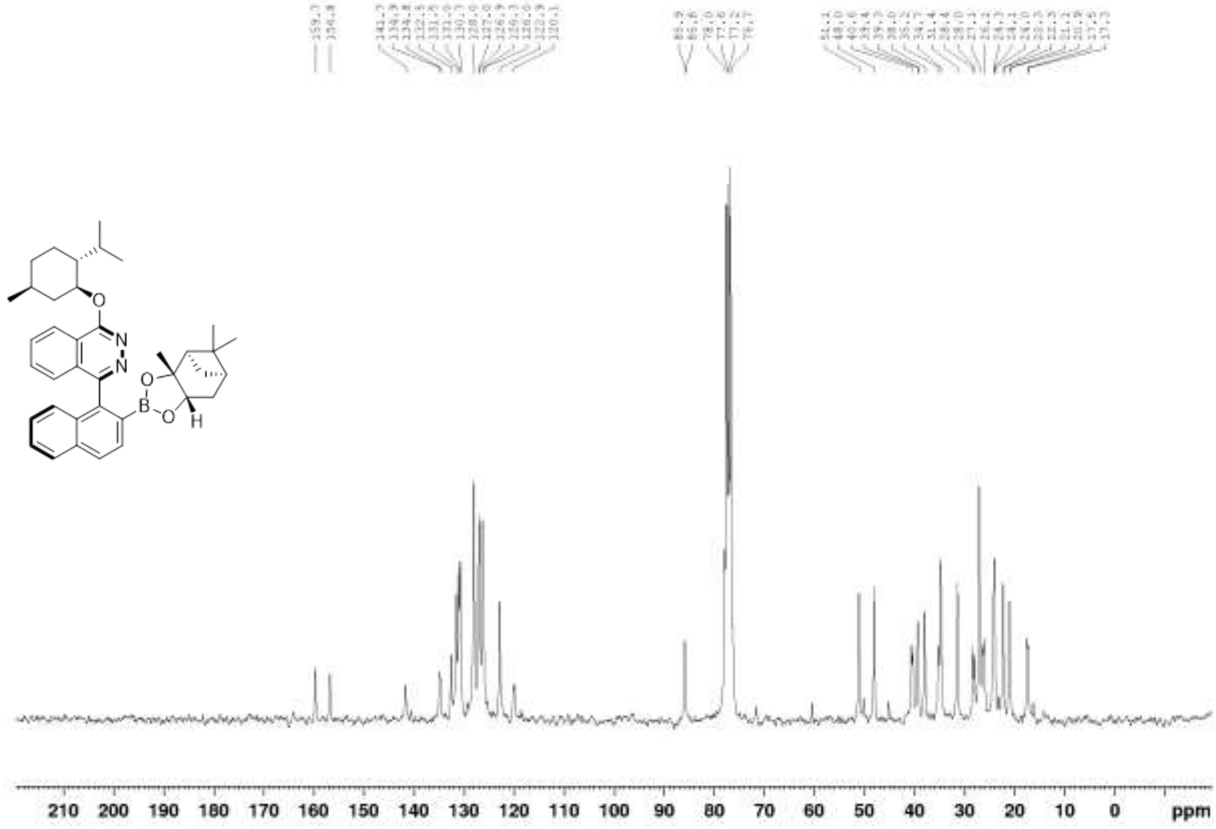

^{1}H NMR ($CDCl_3$, 300 Hz) of **4b**

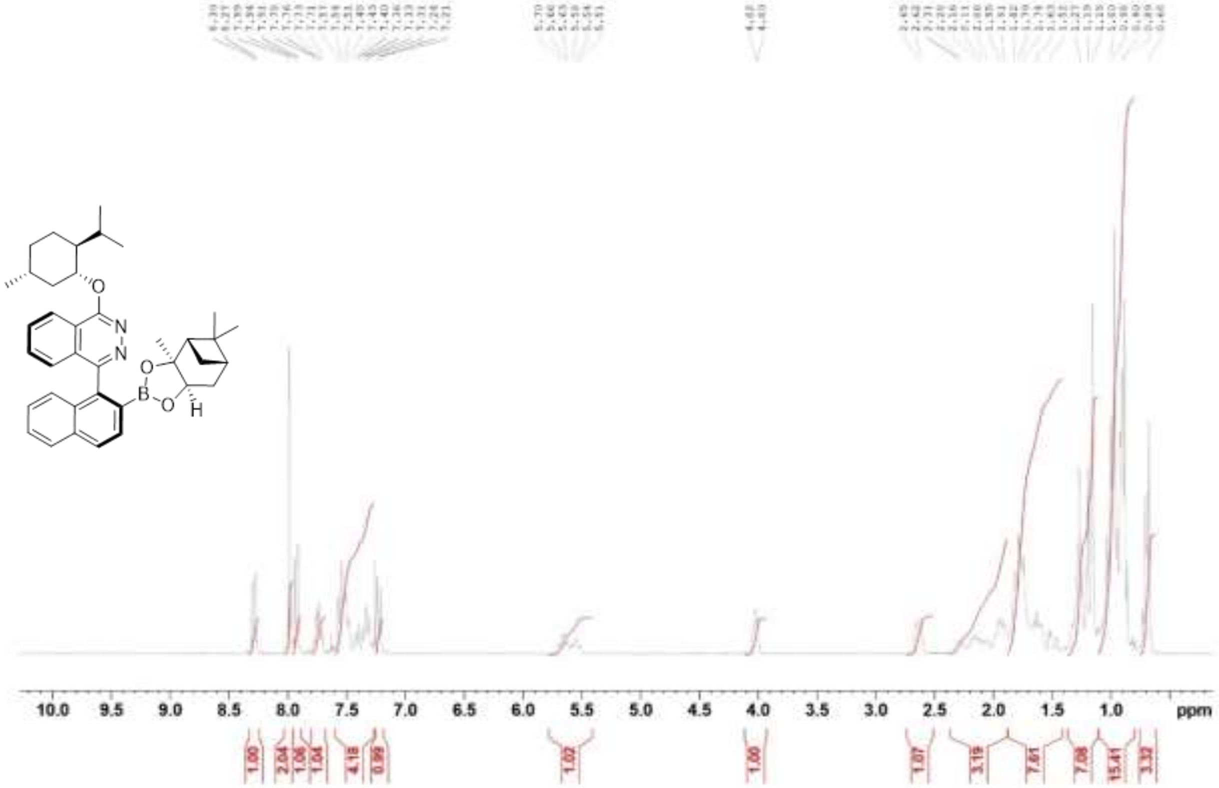

^{13}C{^{1}H} NMR ($CDCl_3$, 125 Hz) of **4b**

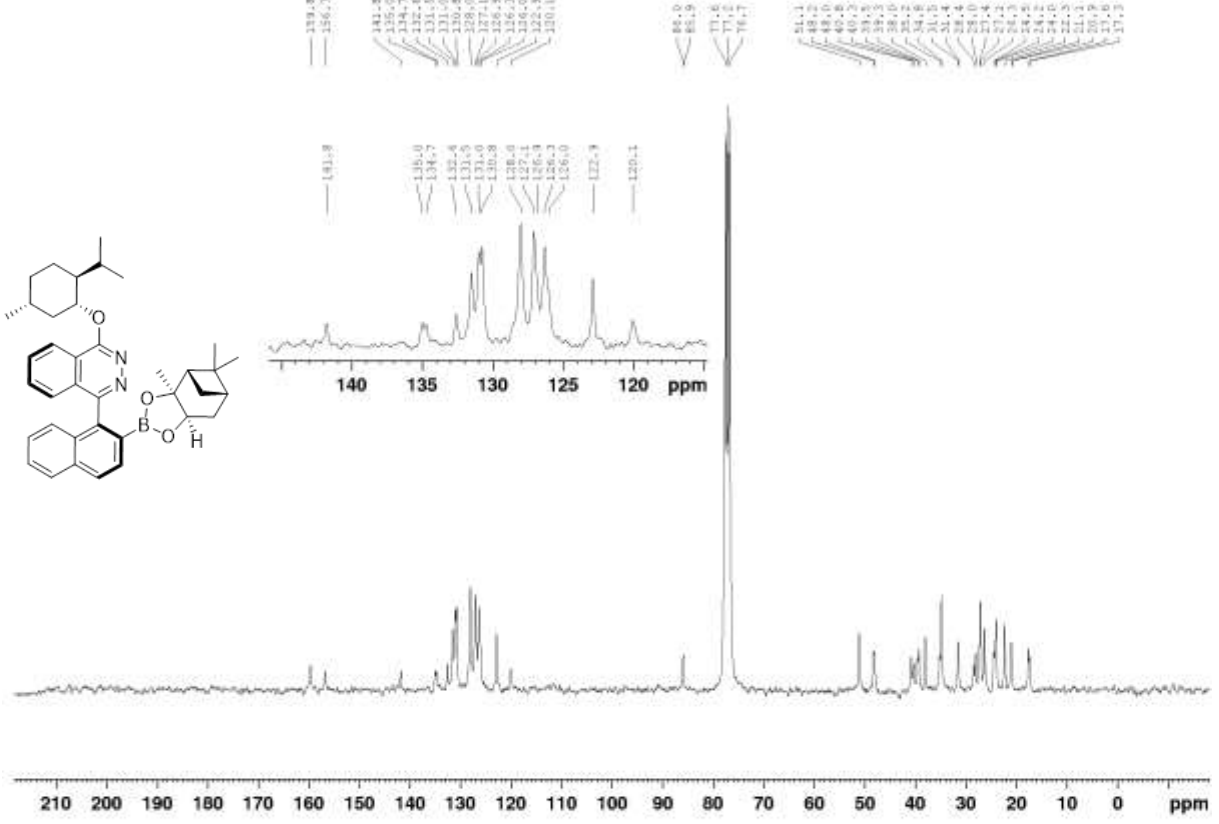

^{1}H NMR ($CDCl_3$, 300 Hz) of **4c**

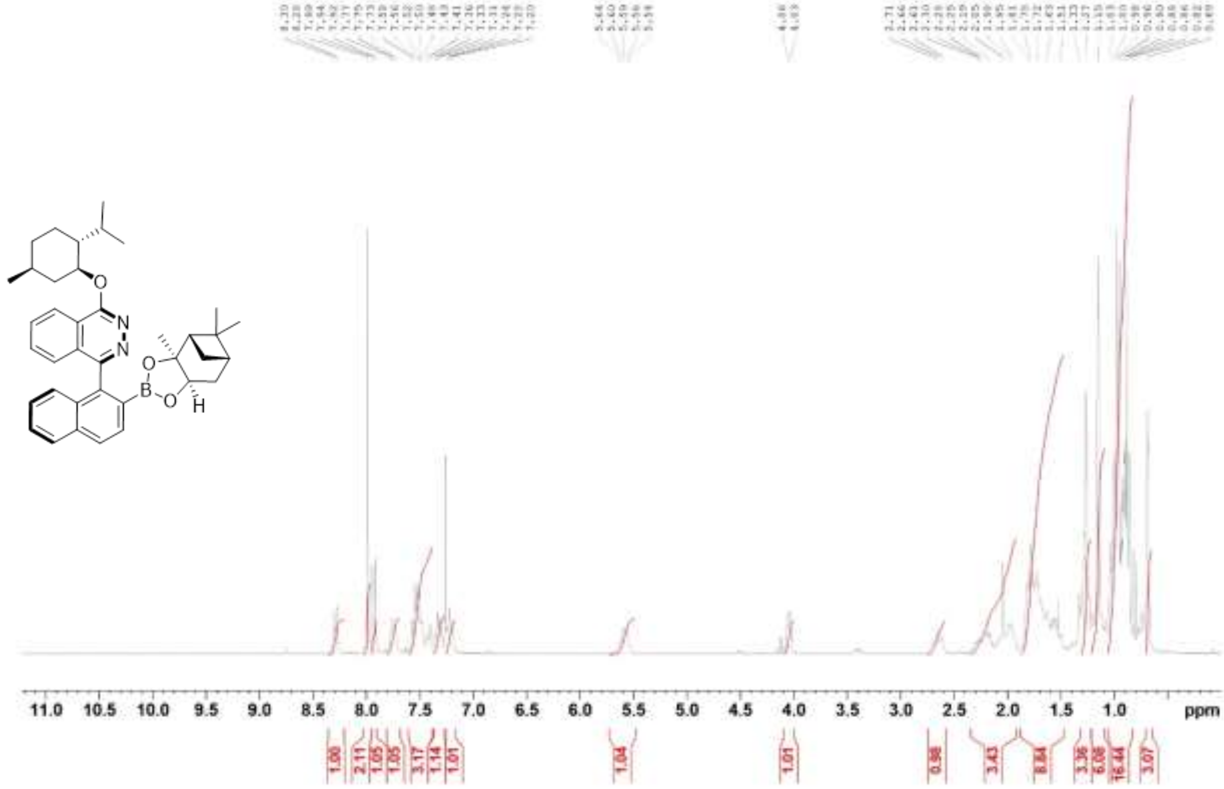

$^{13}C\{^{1}H\}$ NMR ($CDCl_3$, 125 Hz) of **4c**

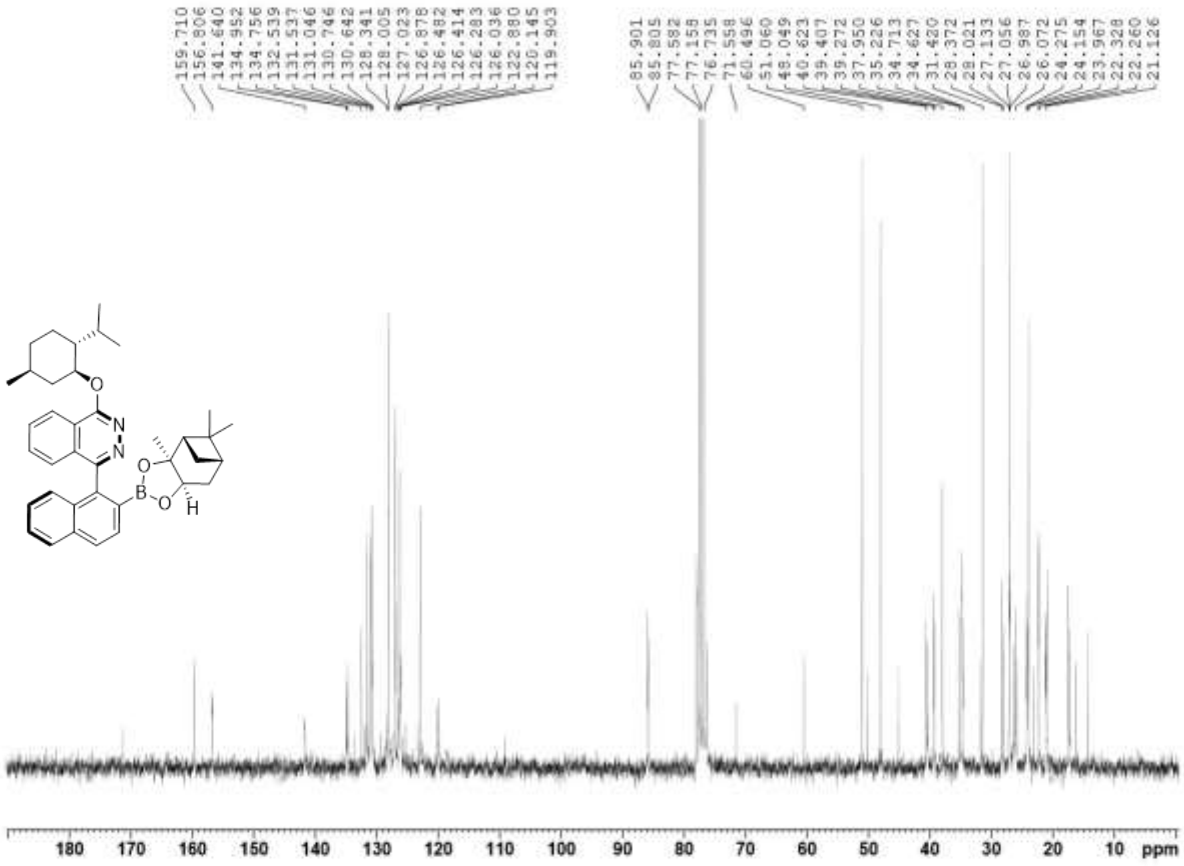

^{11}B NMR ($CDCl_3$, 96 Hz) of **4c**

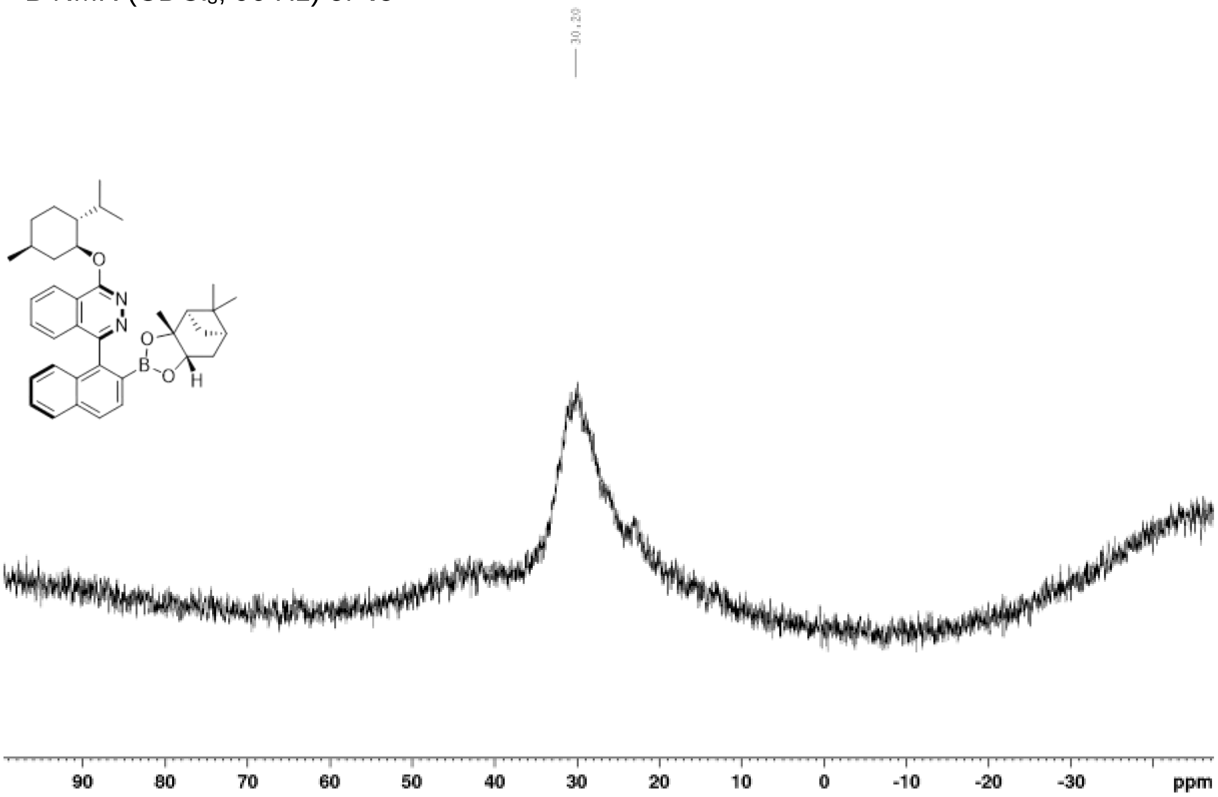

^{1}H NMR ($CDCl_3$, 300 Hz) of **4d**

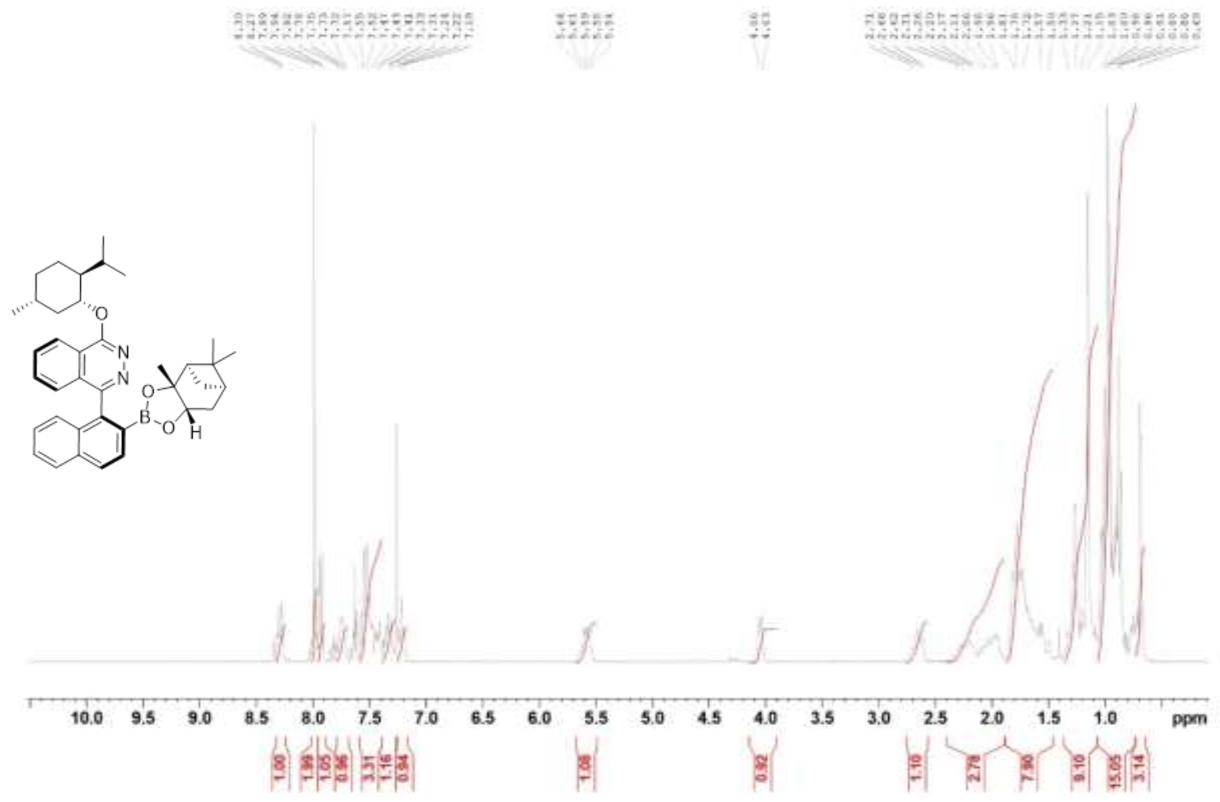

^{13}C{^{1}H} NMR ($CDCl_3$, 125 Hz) of **4d**

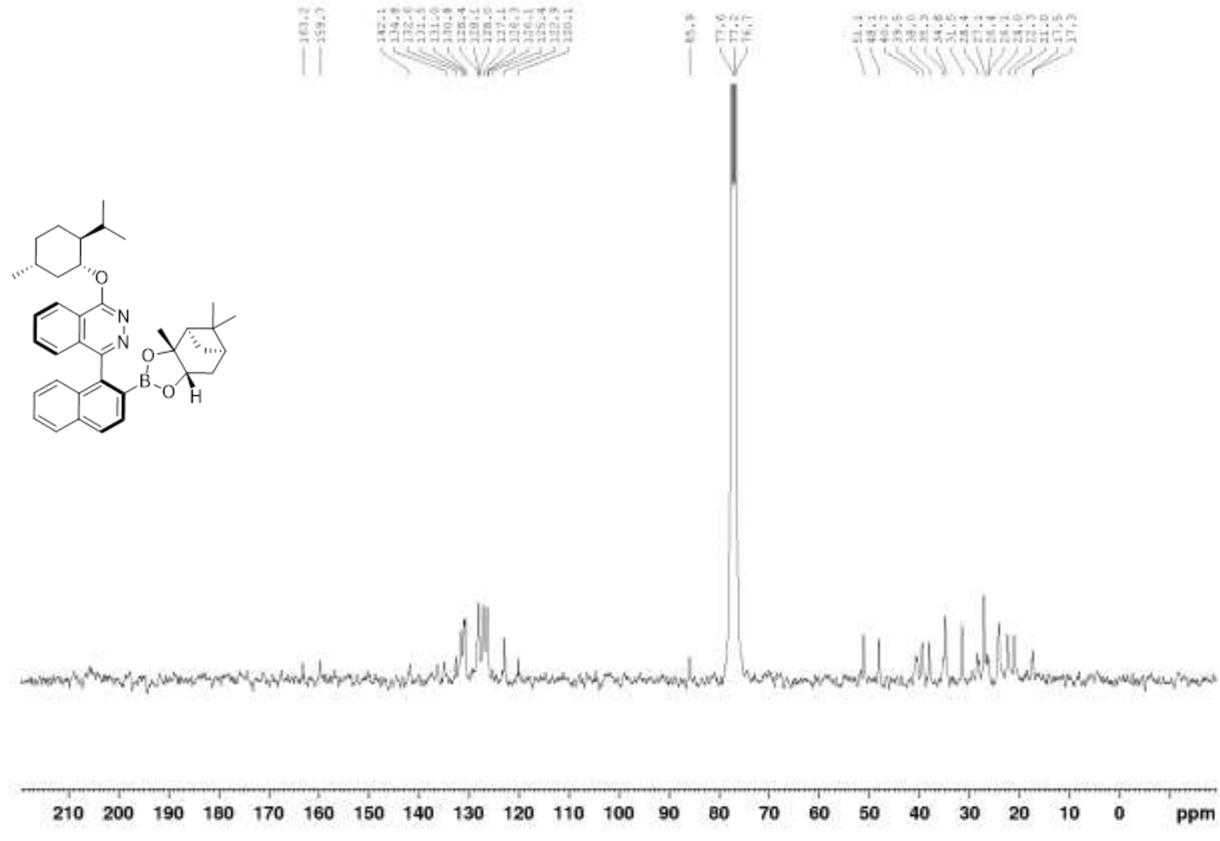

^{1}H NMR ($CDCl_3$, 300 Hz) of **4e**

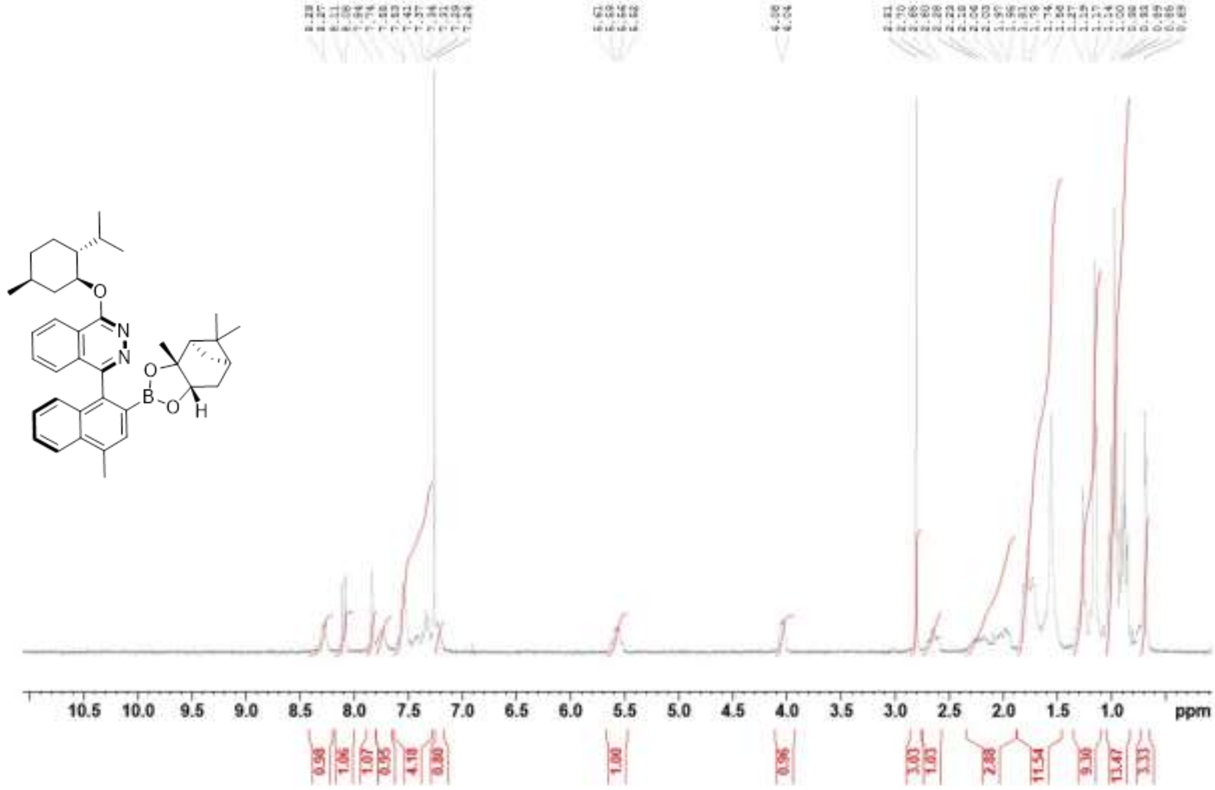

$^{13}C\{^{1}H\}$ NMR ($CDCl_3$, 125 Hz) of **4e**

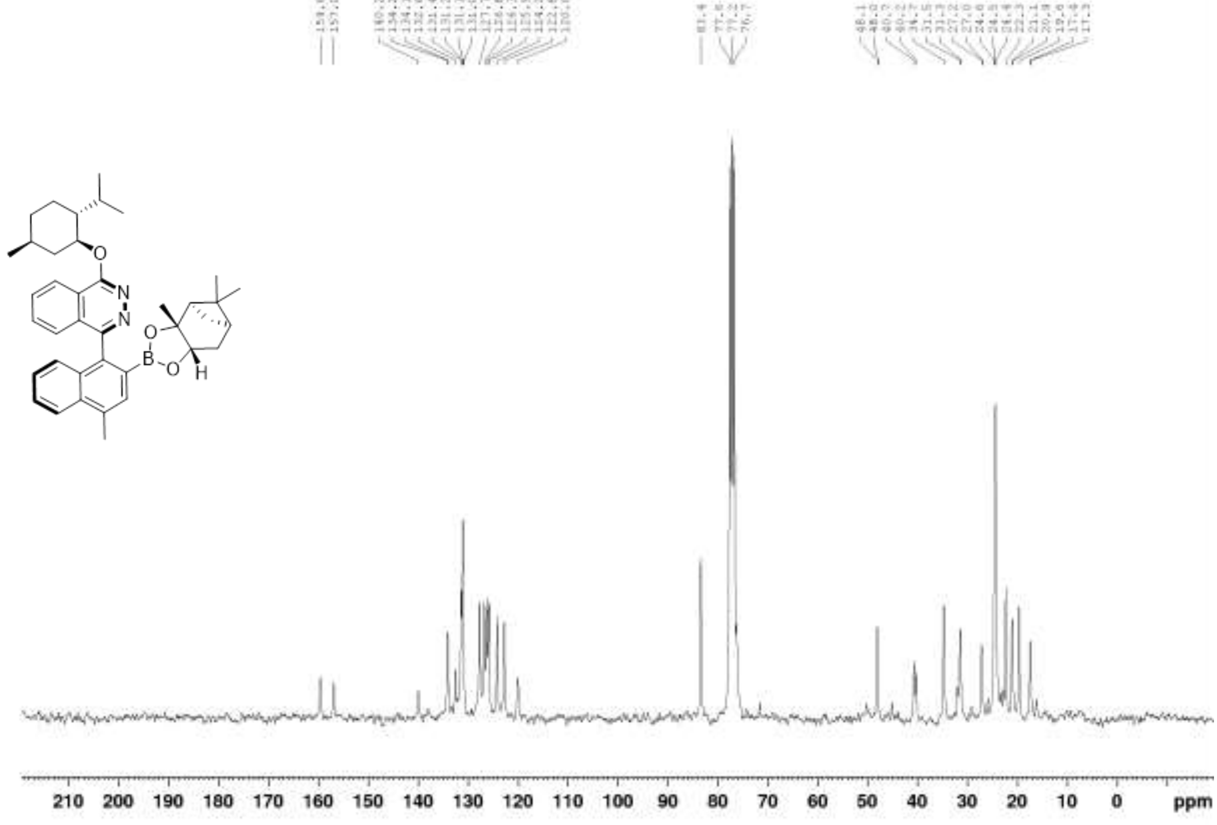

^{1}H NMR ($CDCl_3$, 300 Hz) of **4f**

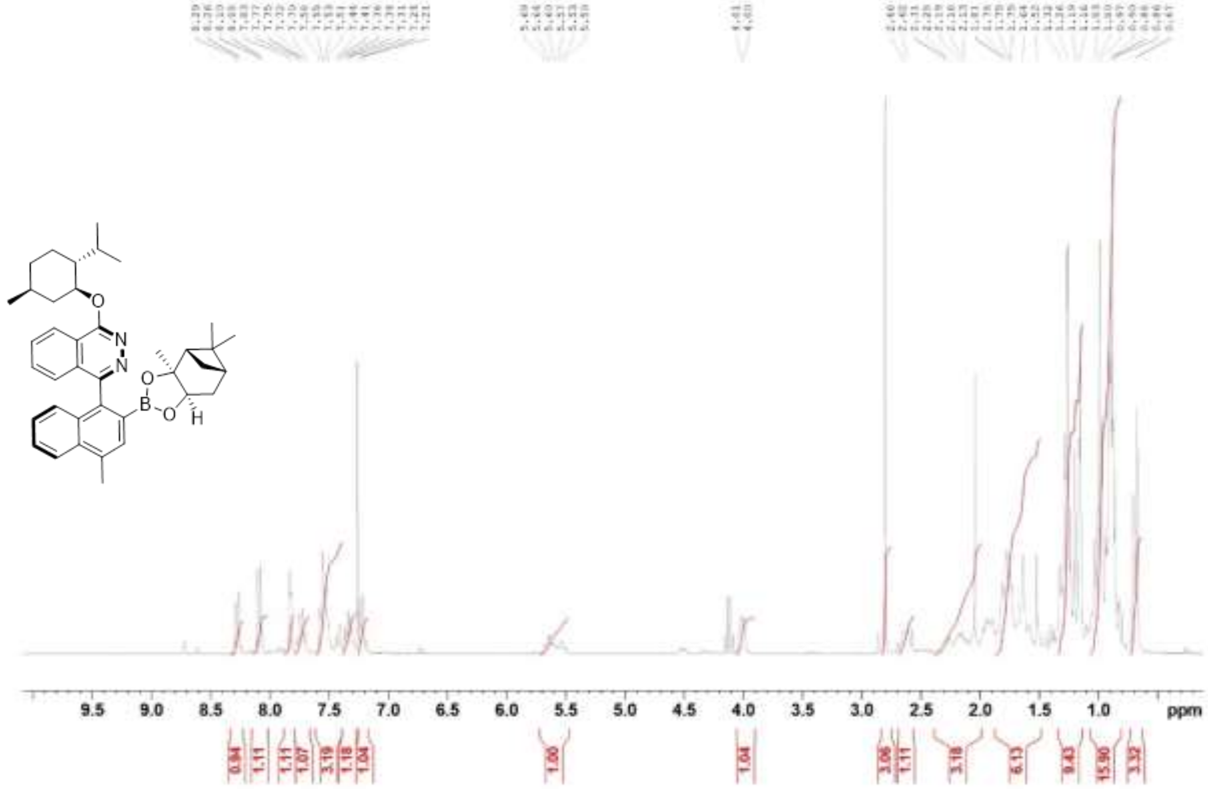

$^{13}C\{^{1}H\}$ NMR ($CDCl_3$, 125 Hz) of **4f**

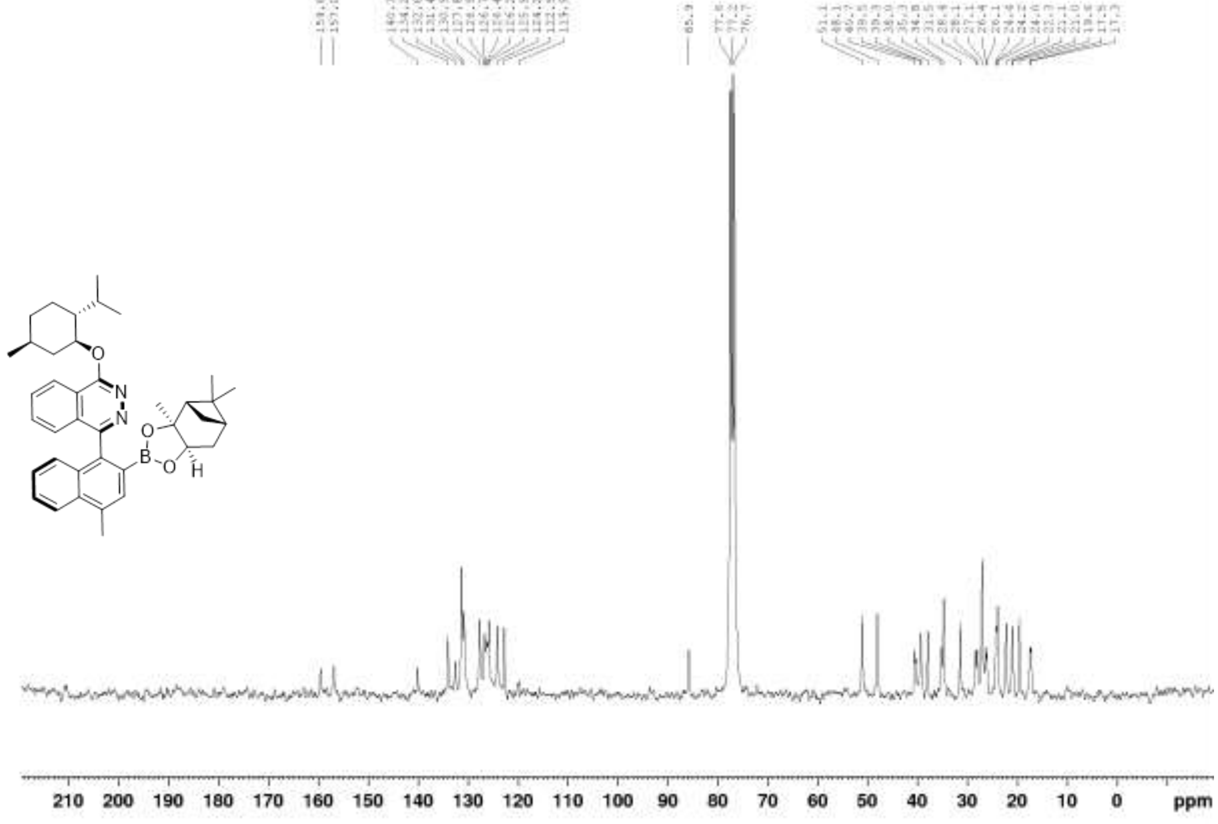

^{1}H NMR ($CDCl_3$, 300 Hz) of **4g**

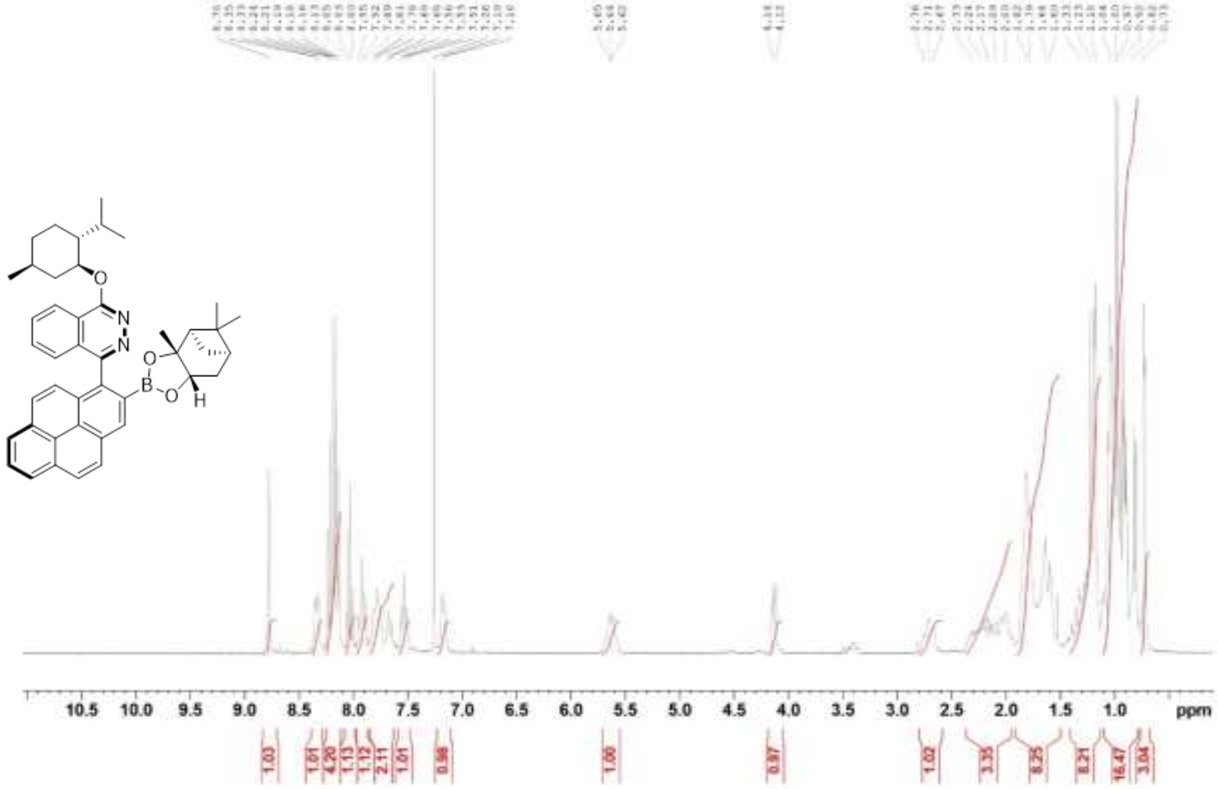

$^{13}C\{^{1}H\}$ NMR ($CDCl_3$, 125 Hz) of **4g**

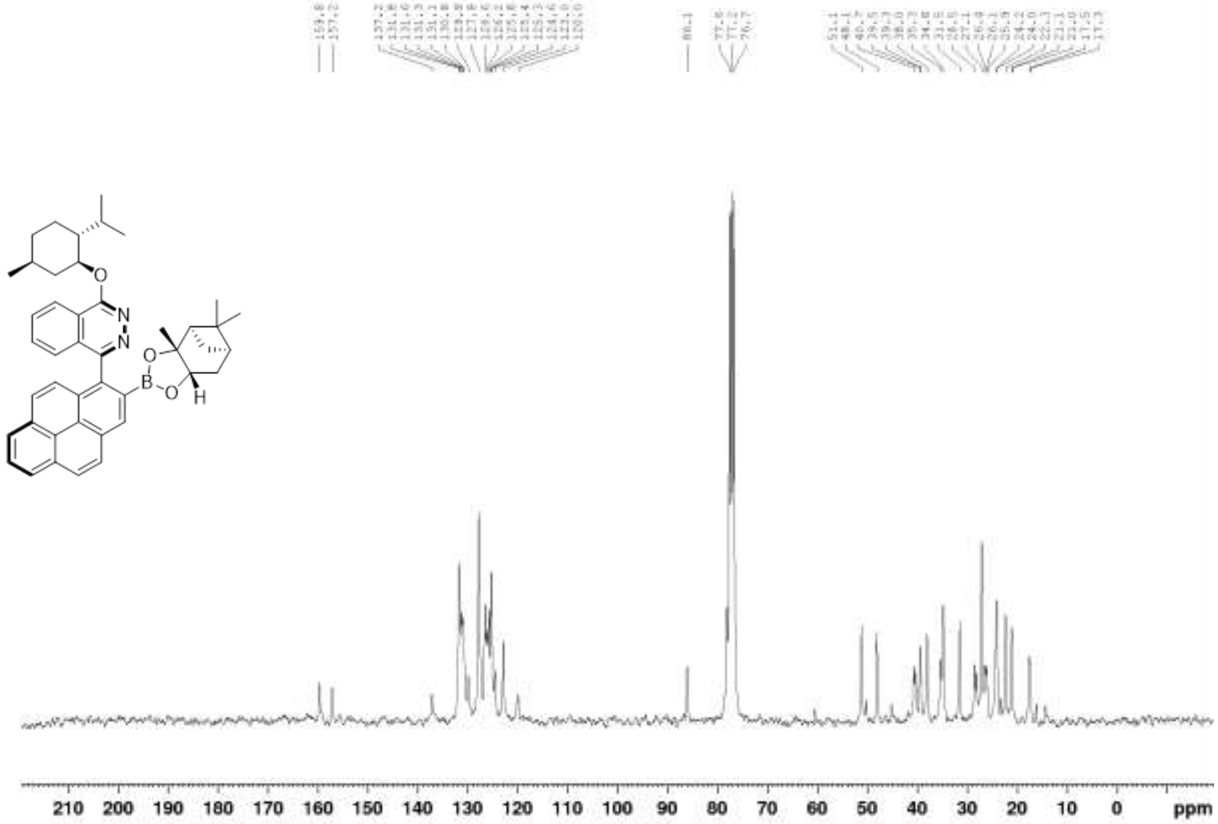

^{1}H NMR ($CDCl_3$, 300 Hz) of **4h**

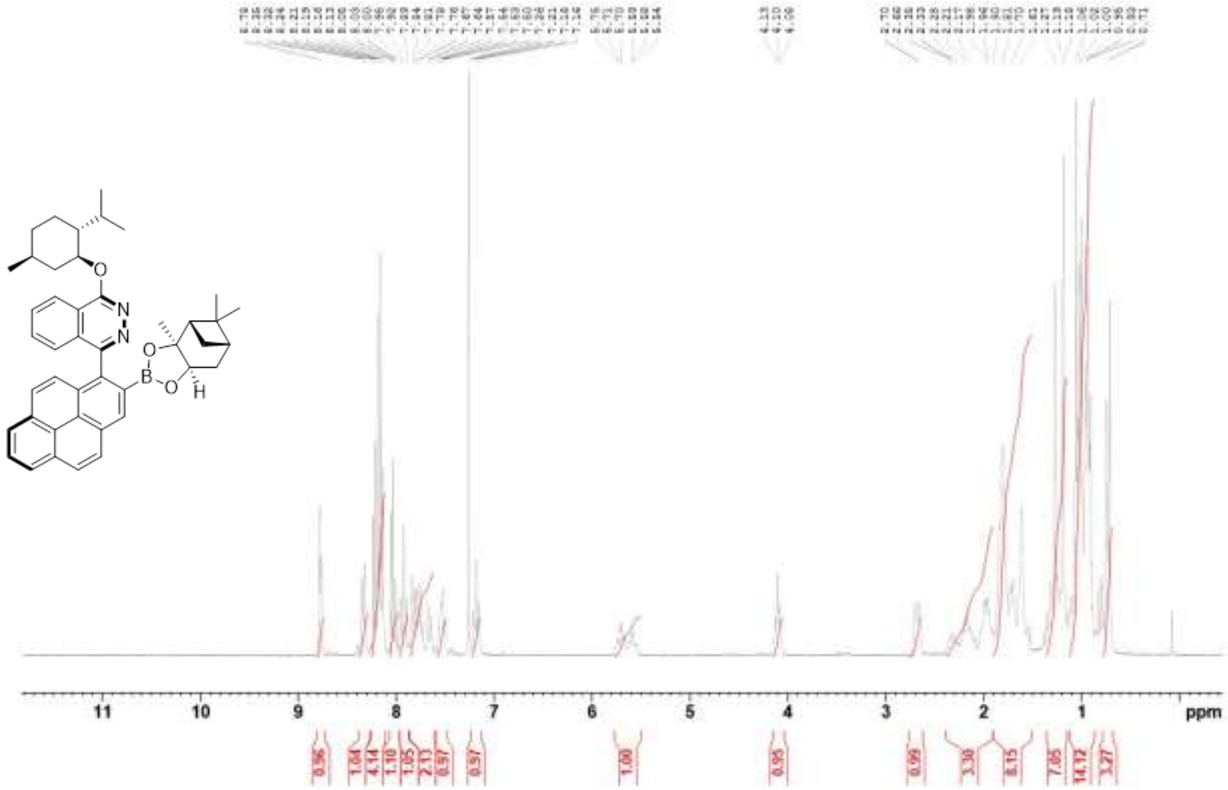

$^{13}C\{^{1}H\}$ NMR ($CDCl_3$, 125 Hz) of **4h**

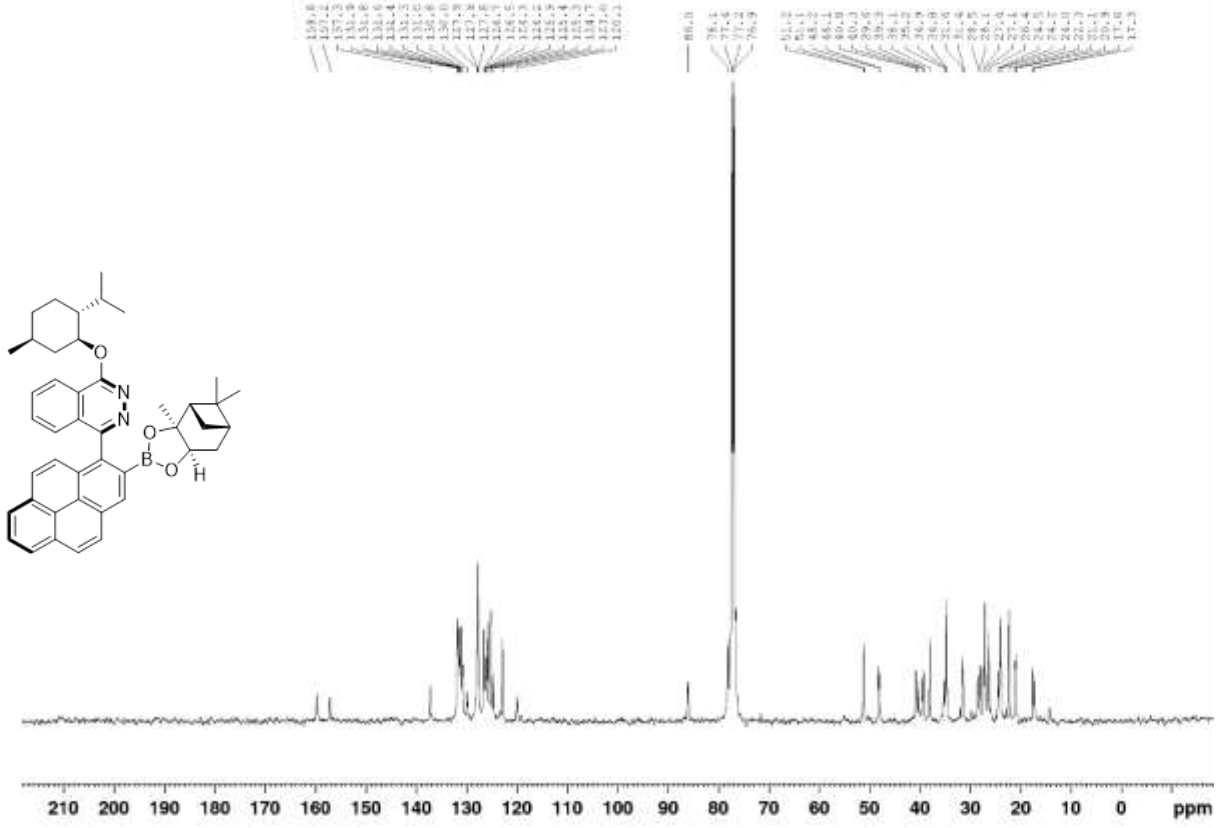

^{1}H NMR ($CDCl_3$, 300 Hz) of **4i-SI**

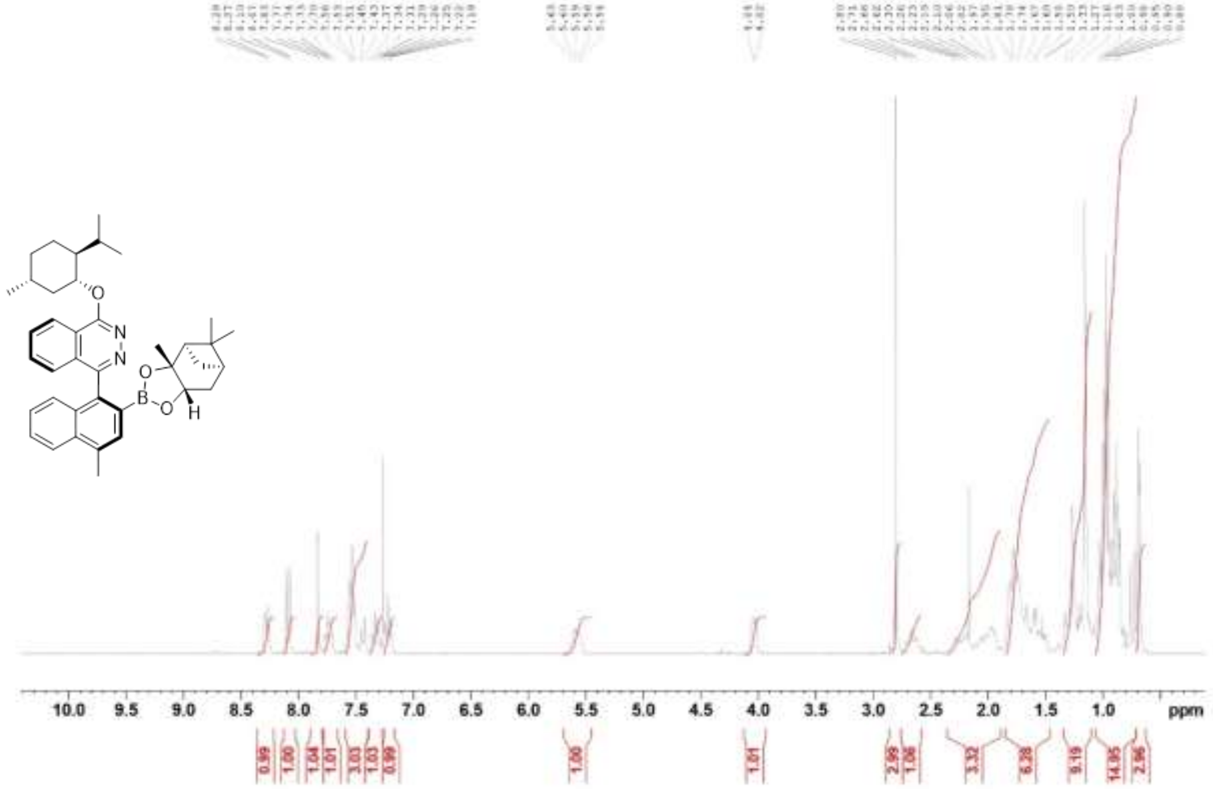

$^{13}C\{^{1}H\}$ NMR ($CDCl_3$, 125 Hz) of **4i-SI**

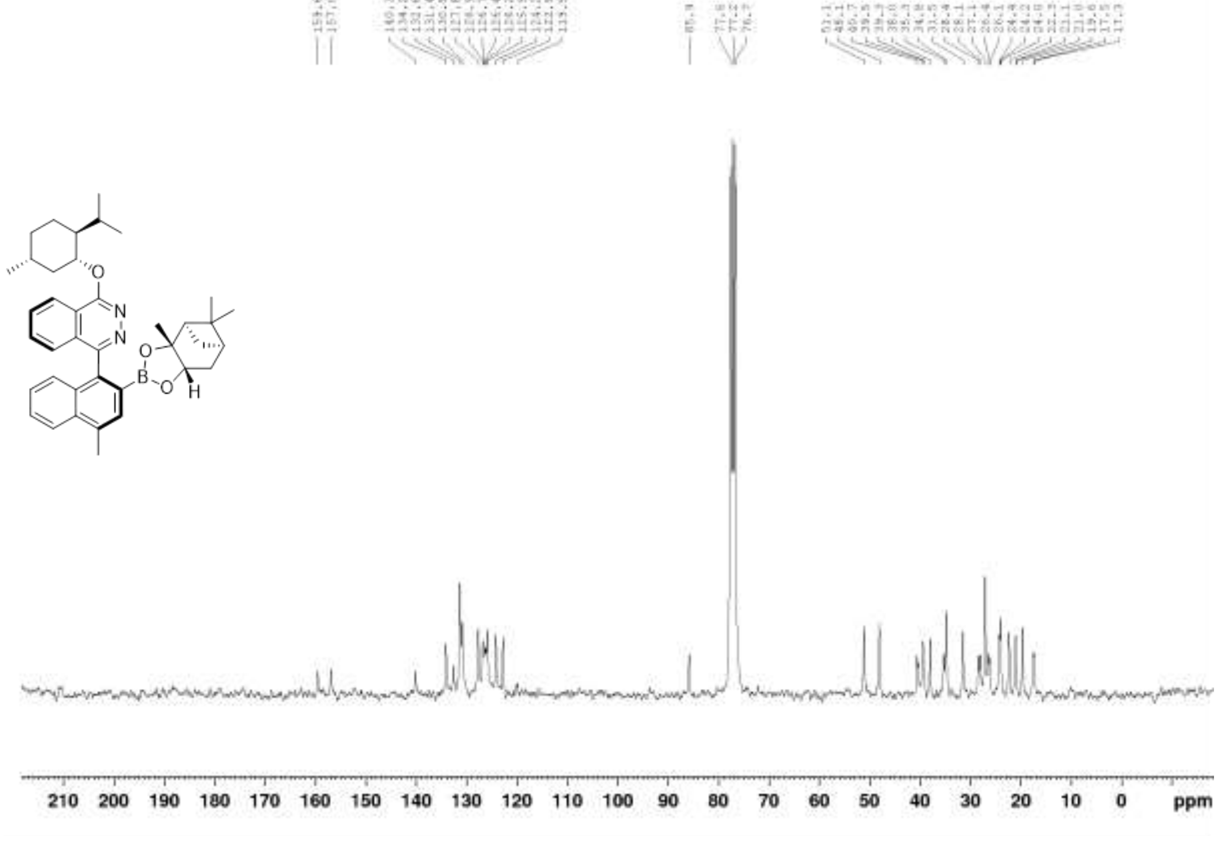

^{1}H NMR ($CDCl_3$, 300 Hz) of **4j-SI**

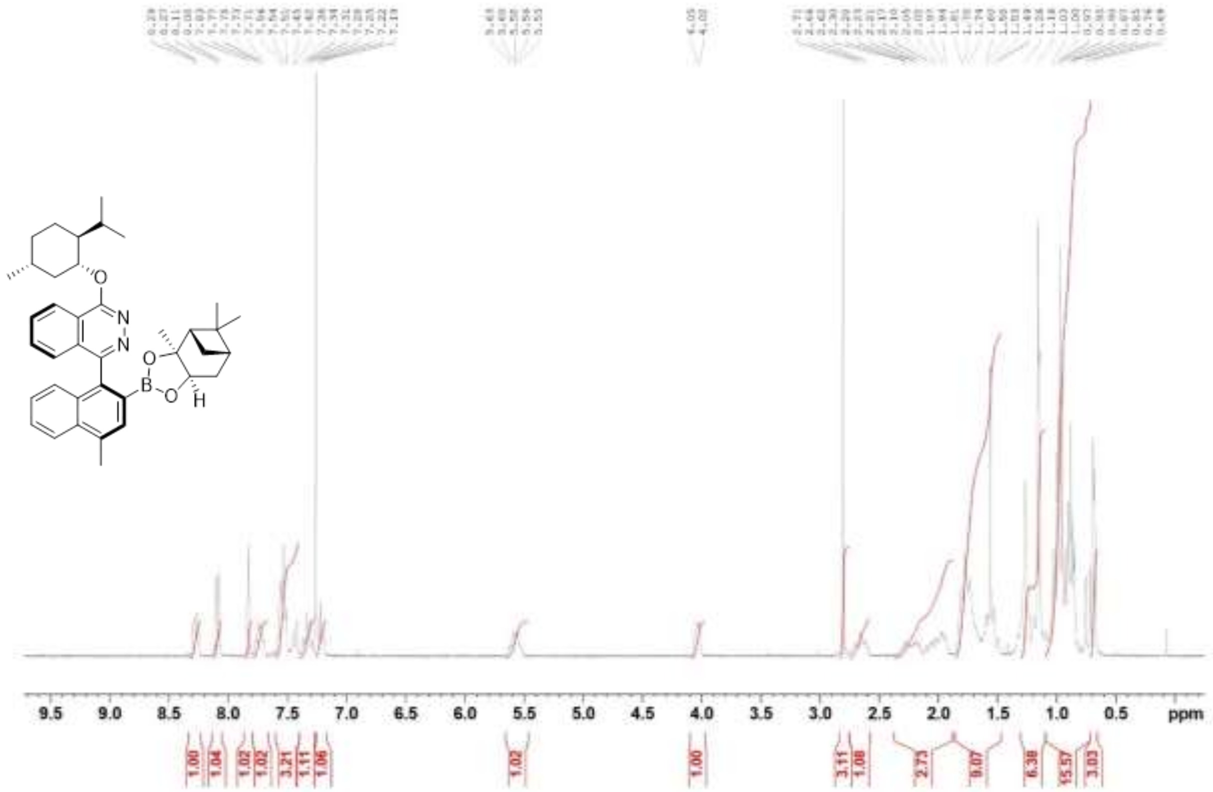

^{13}C{^{1}H} NMR ($CDCl_3$, 125 Hz) of **4j-SI**

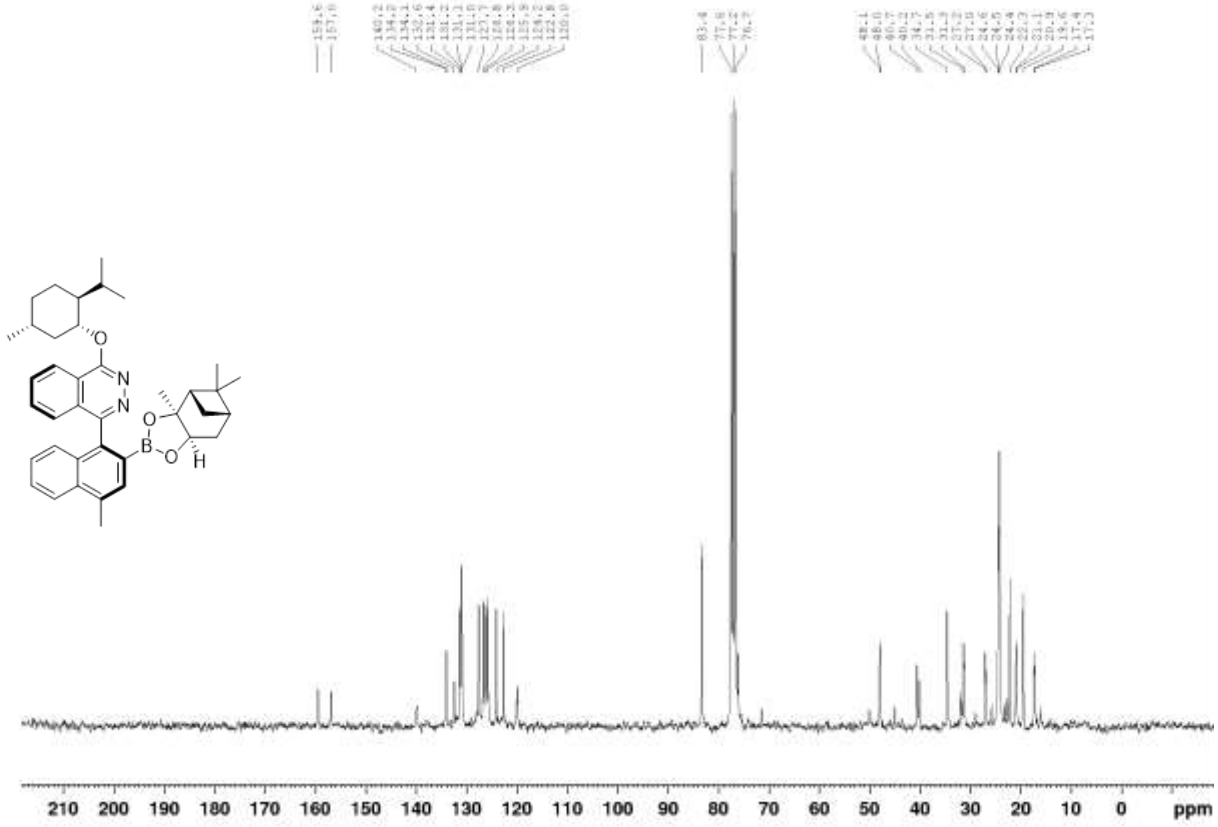

^{1}H NMR ($CDCl_3$, 300 Hz) of **4k-SI**

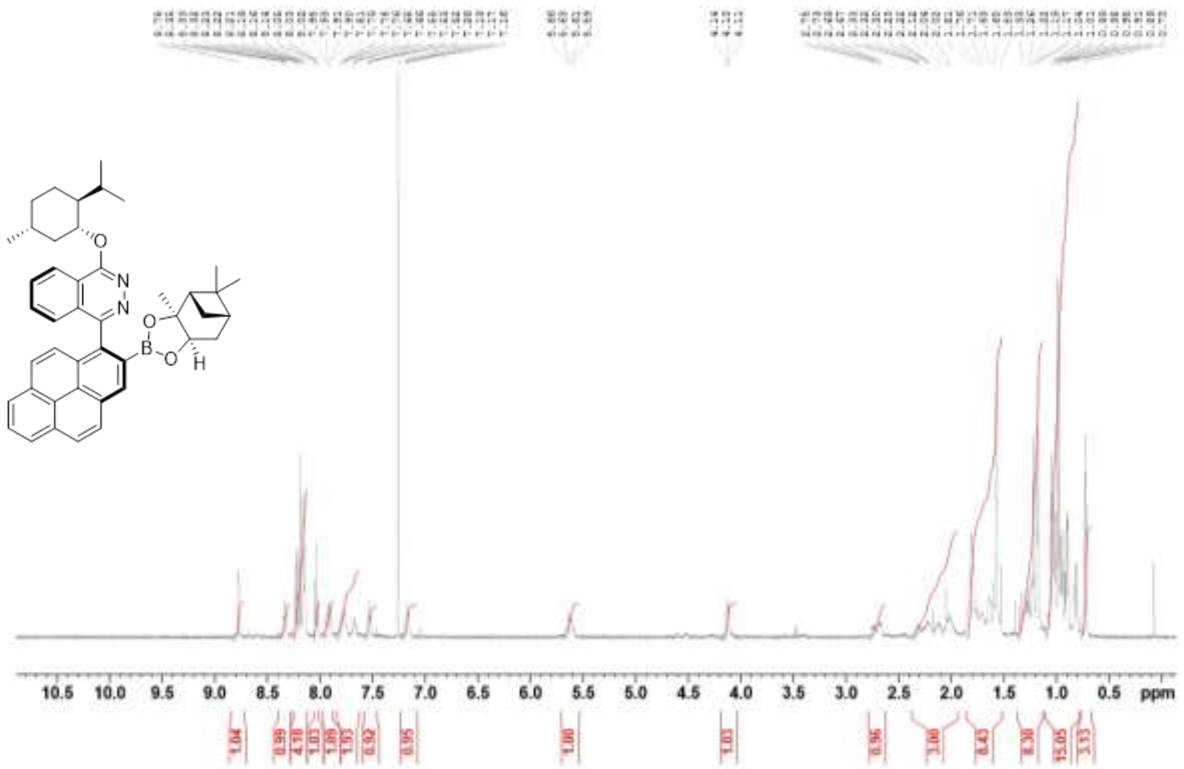

$^{13}C\{^{1}H\}$ NMR ($CDCl_3$, 125 Hz) of **4k-SI**

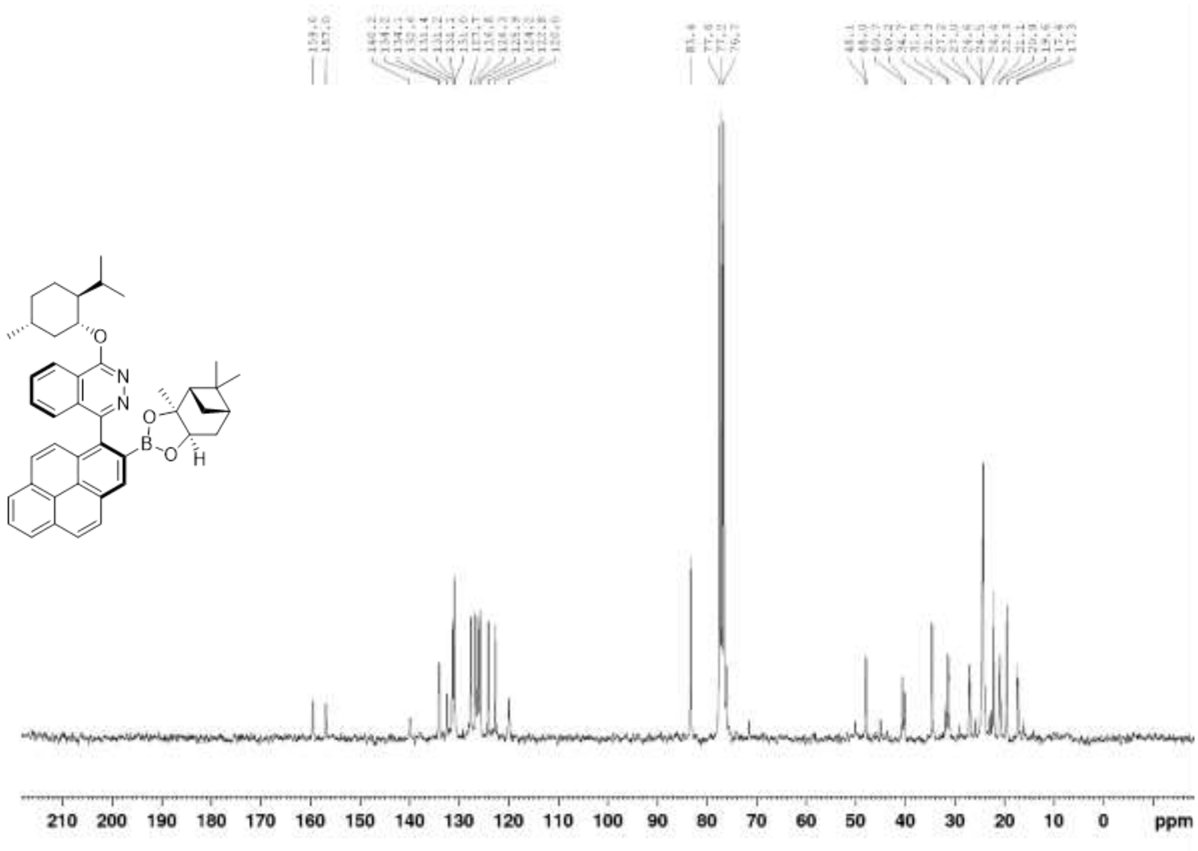

^{1}H NMR ($CDCl_3$, 300 Hz) of **4l-SI**

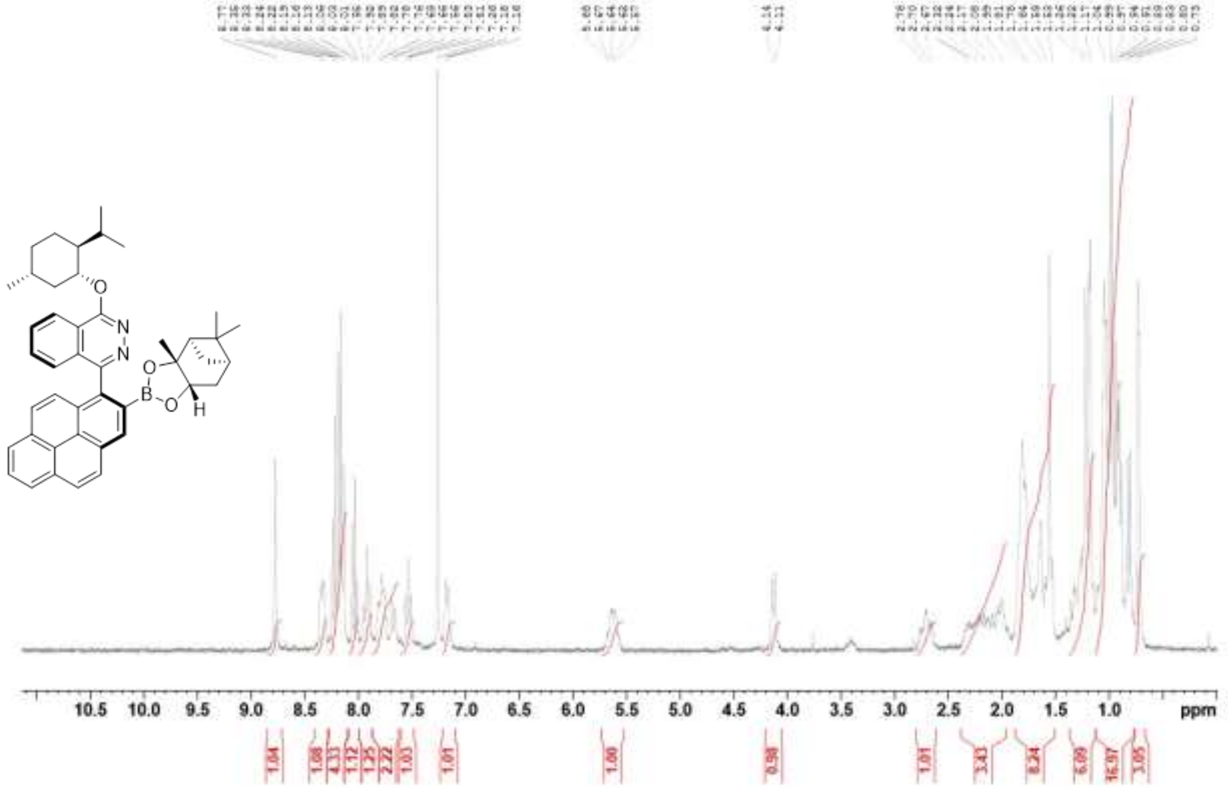

$^{13}C\{^{1}H\}$ NMR ($CDCl_3$, 125 Hz) of **4l-SI**

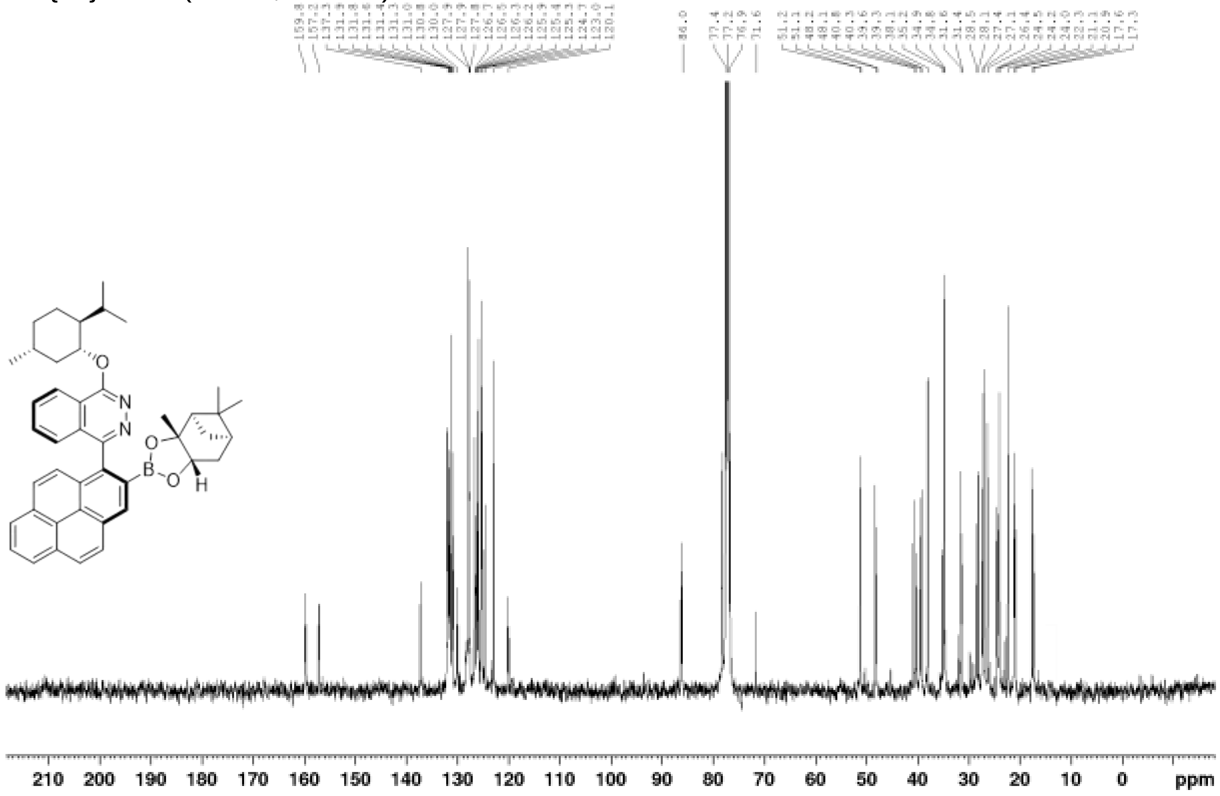

^{1}H NMR ($CDCl_3$, 300 Hz) of **5a**

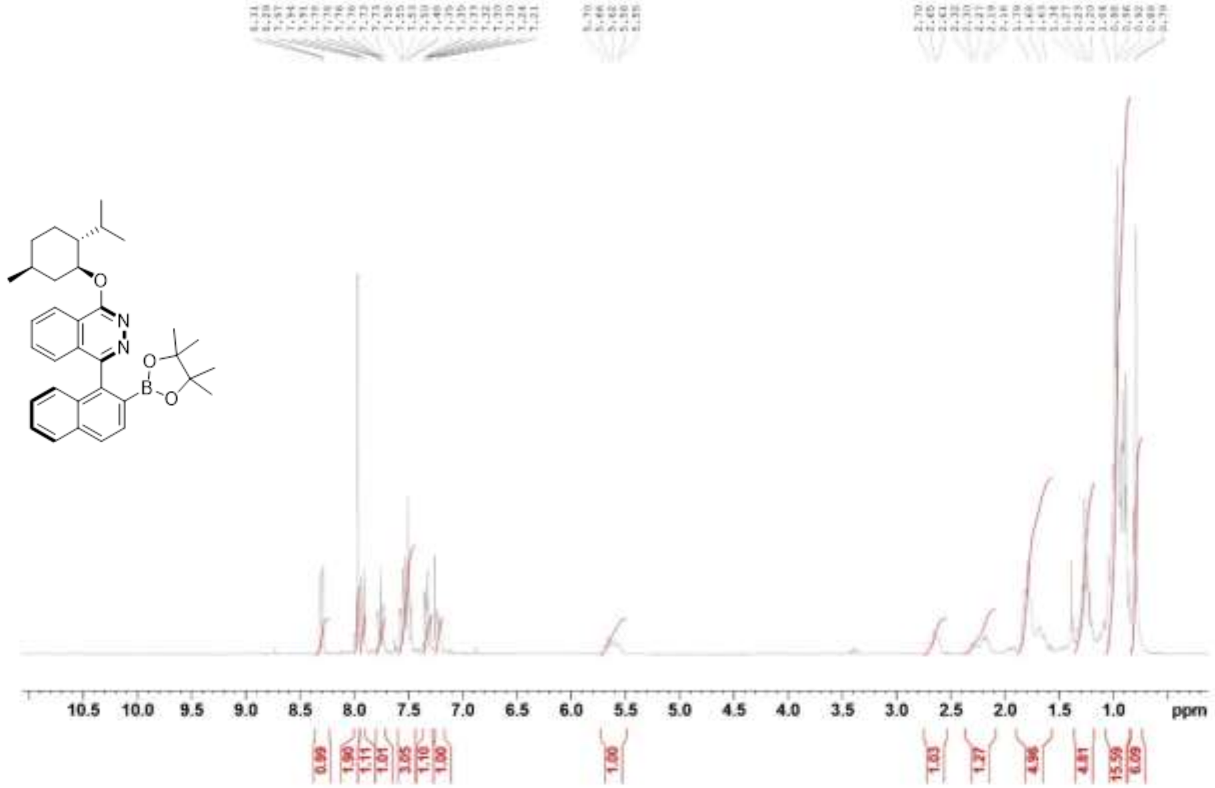

$^{13}C\{^{1}H\}$ NMR ($CDCl_3$, 125 Hz) of **5a**

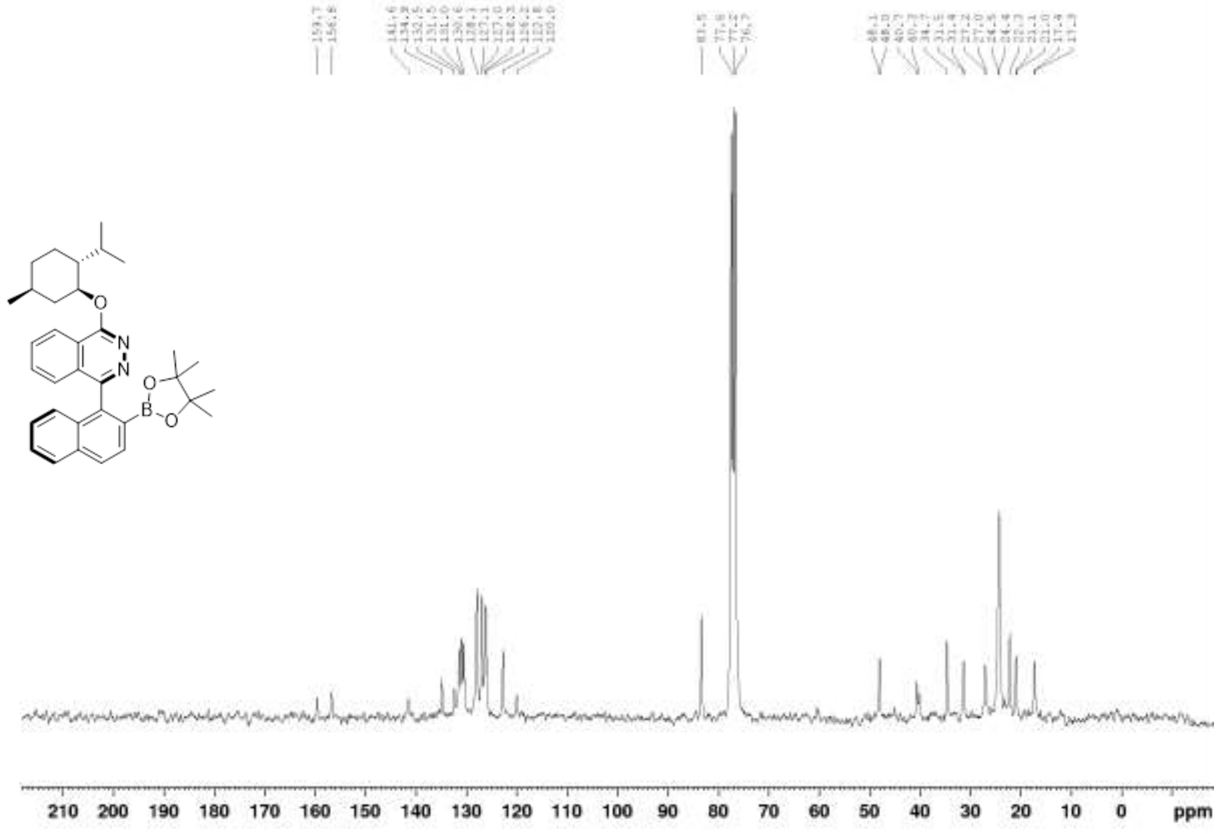

^{1}H NMR ($CDCl_3$, 300 Hz) of **5b**

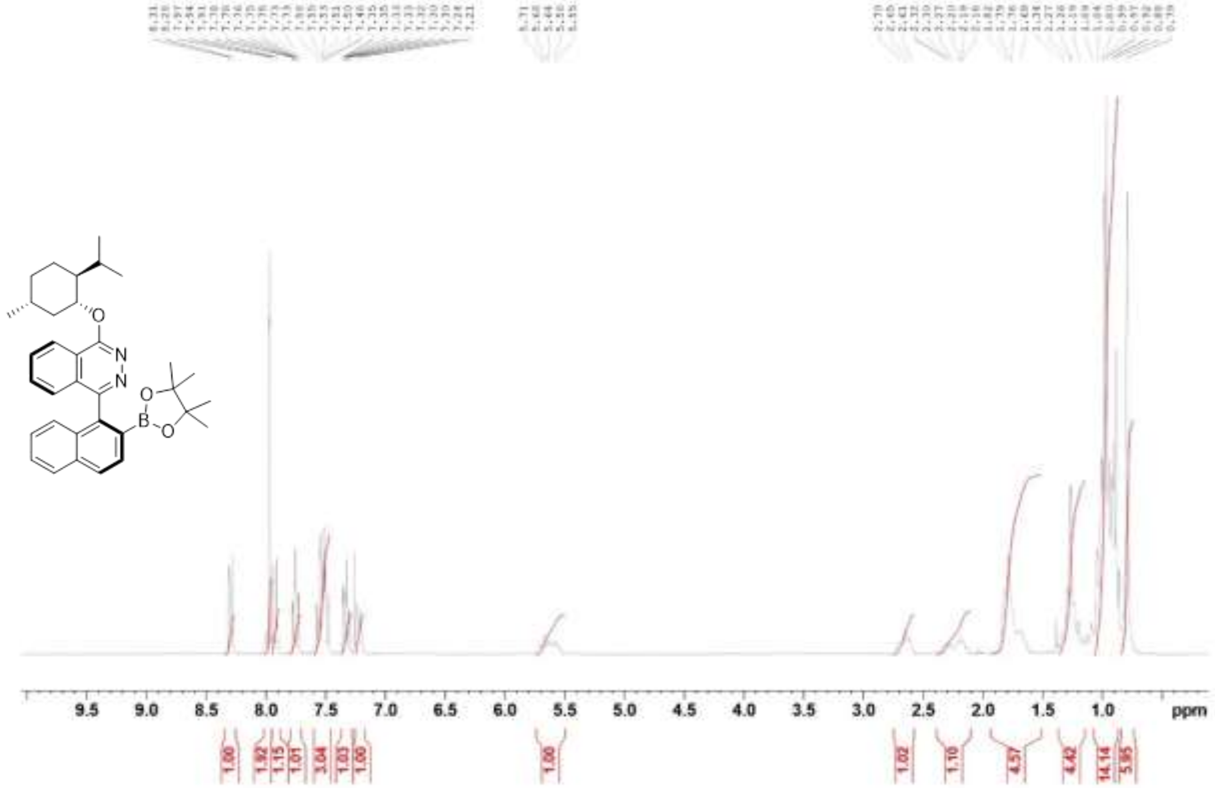

^{13}C{^{1}H} NMR ($CDCl_3$, 125 Hz) of **5b**

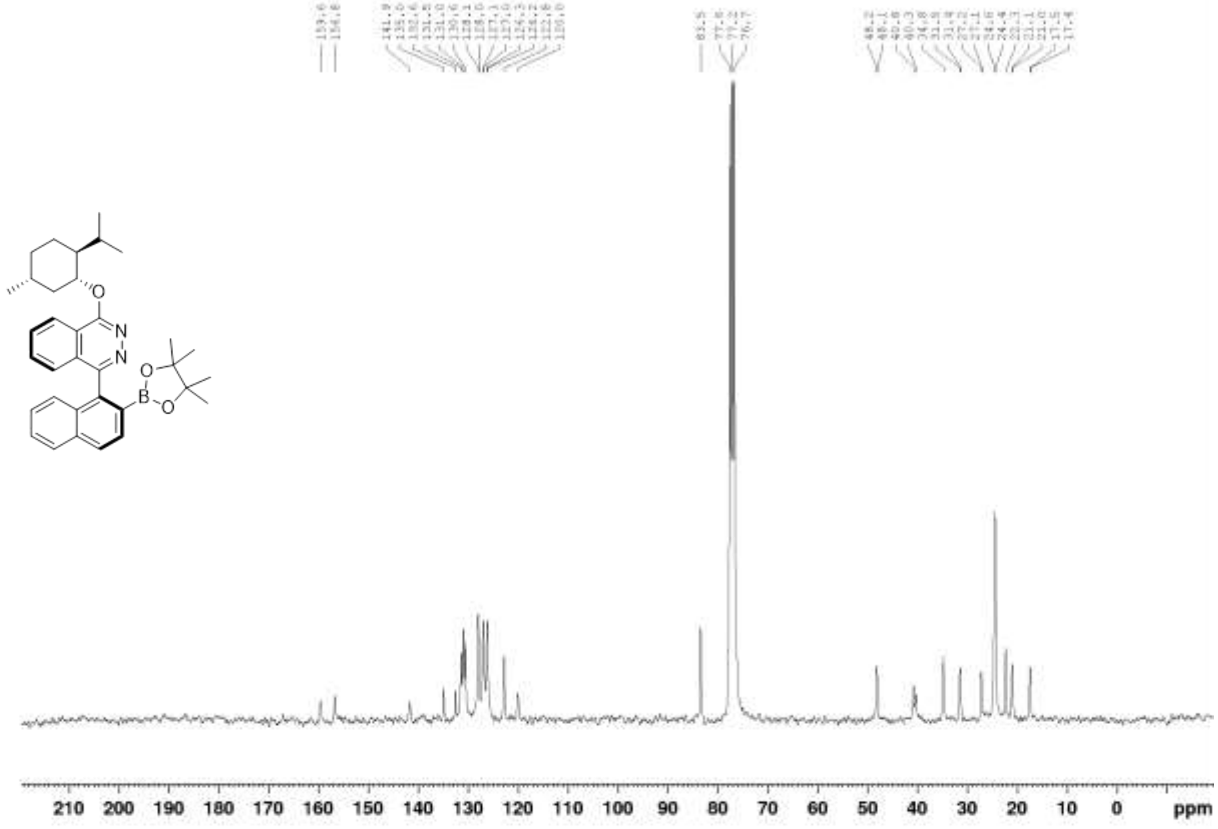

^{1}H NMR ($CDCl_3$, 300 Hz) of **5c**

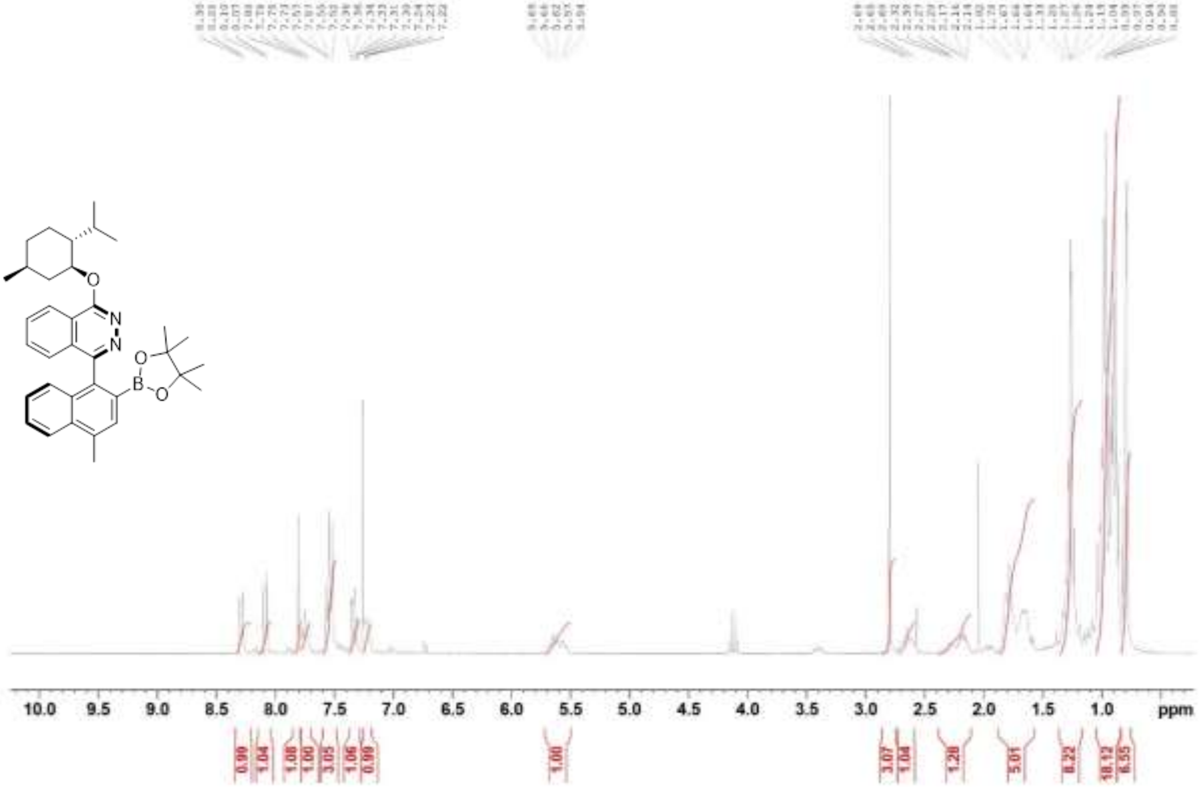

^{13}C{^{1}H} NMR ($CDCl_3$, 125 Hz) of **5c**

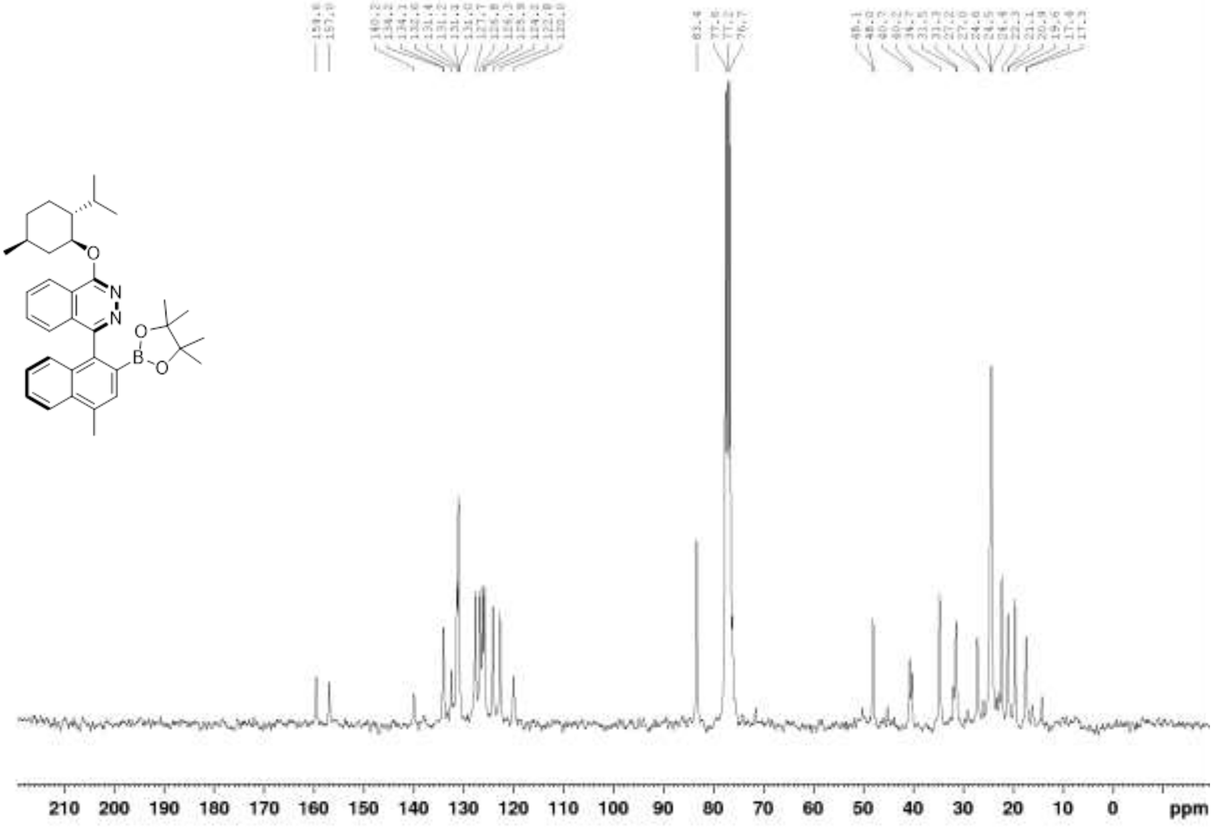

^{1}H NMR ($CDCl_3$, 300 Hz) of **5d**

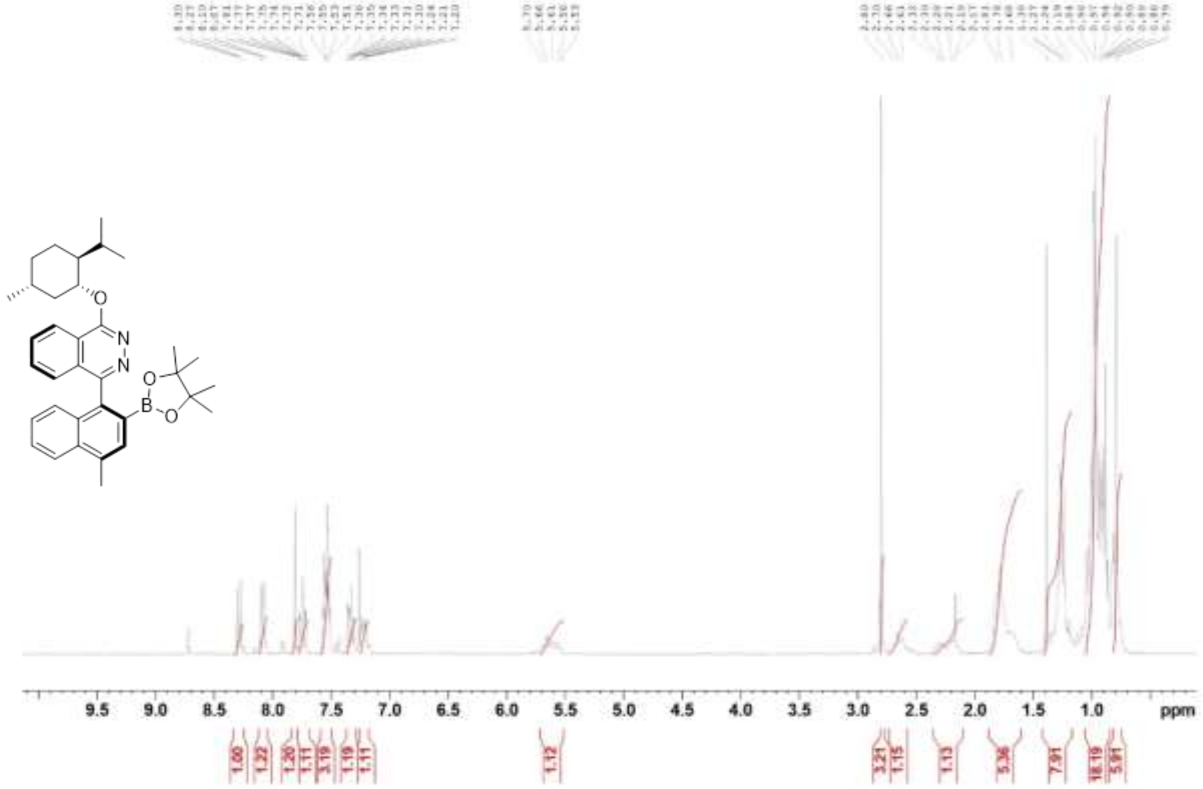

^{13}C{^{1}H} NMR ($CDCl_3$, 125 Hz) of **5d**

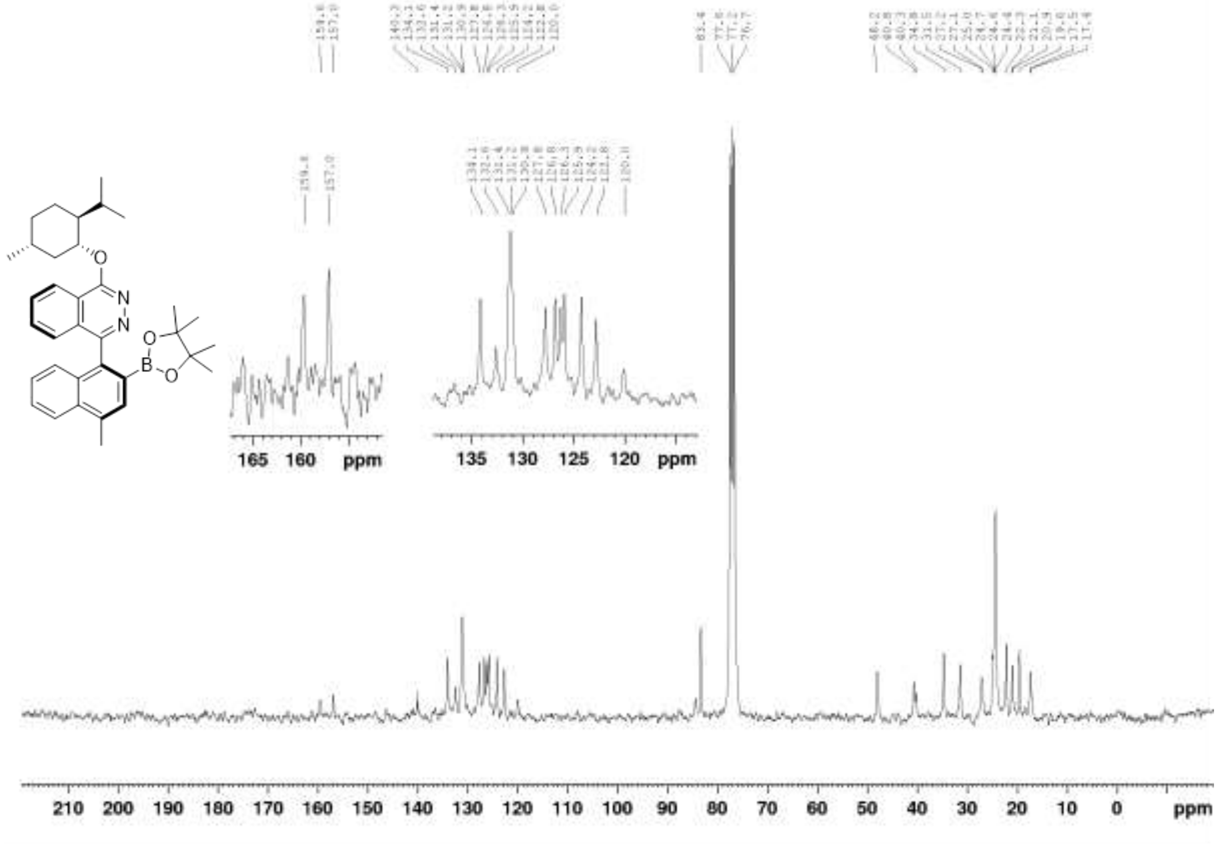

^{1}H NMR ($CDCl_3$, 300 Hz) of **5e**

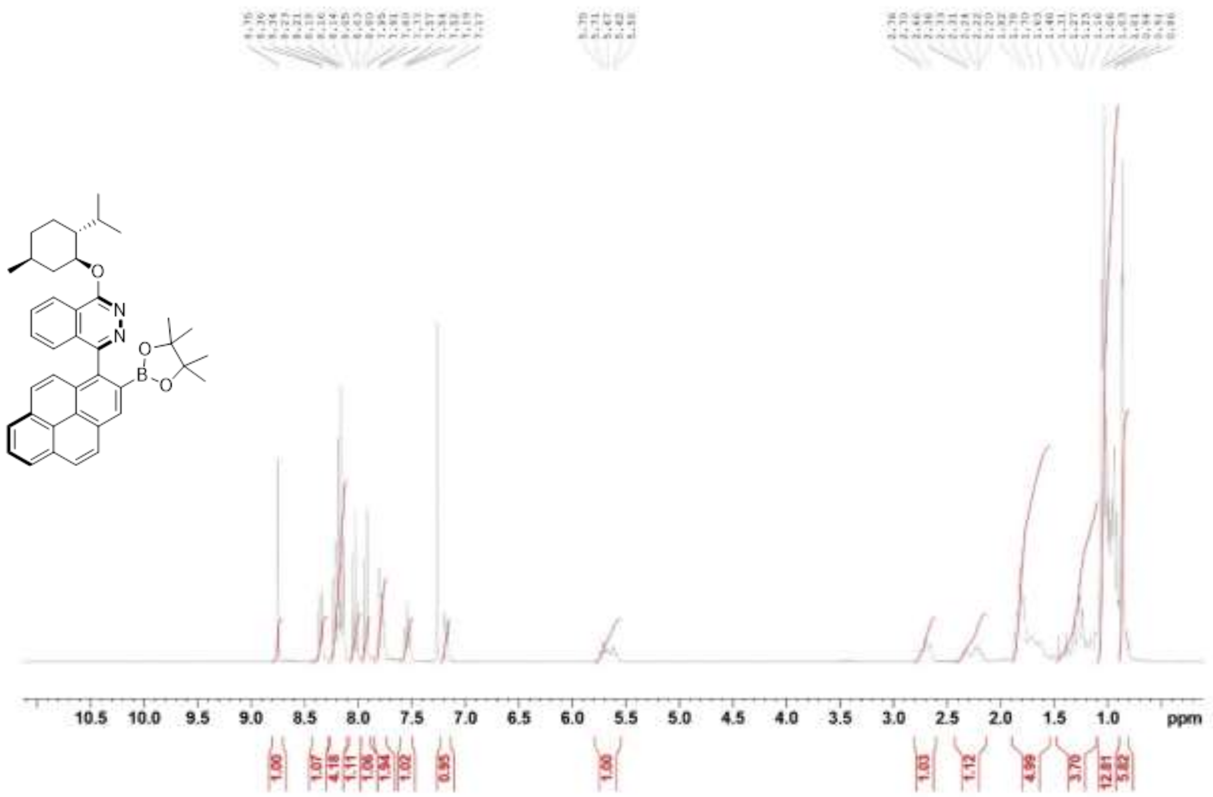

$^{13}C\{^{1}H\}$ NMR ($CDCl_3$, 125 Hz) of **5e**

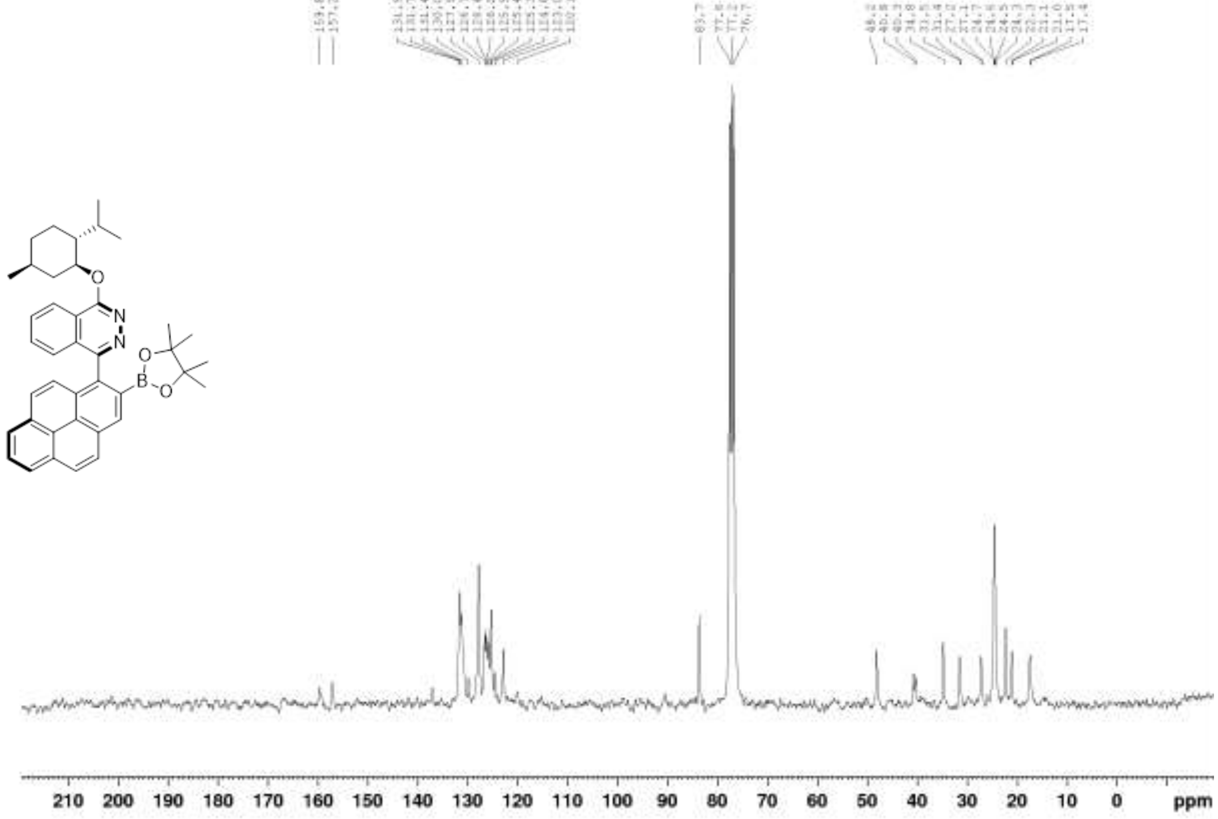

^{1}H NMR ($CDCl_3$, 500 Hz) of **5e** at 20 °C (blue), 0 °C (red) and -10 °C (green) from 9.5 to 5.0 ppm.

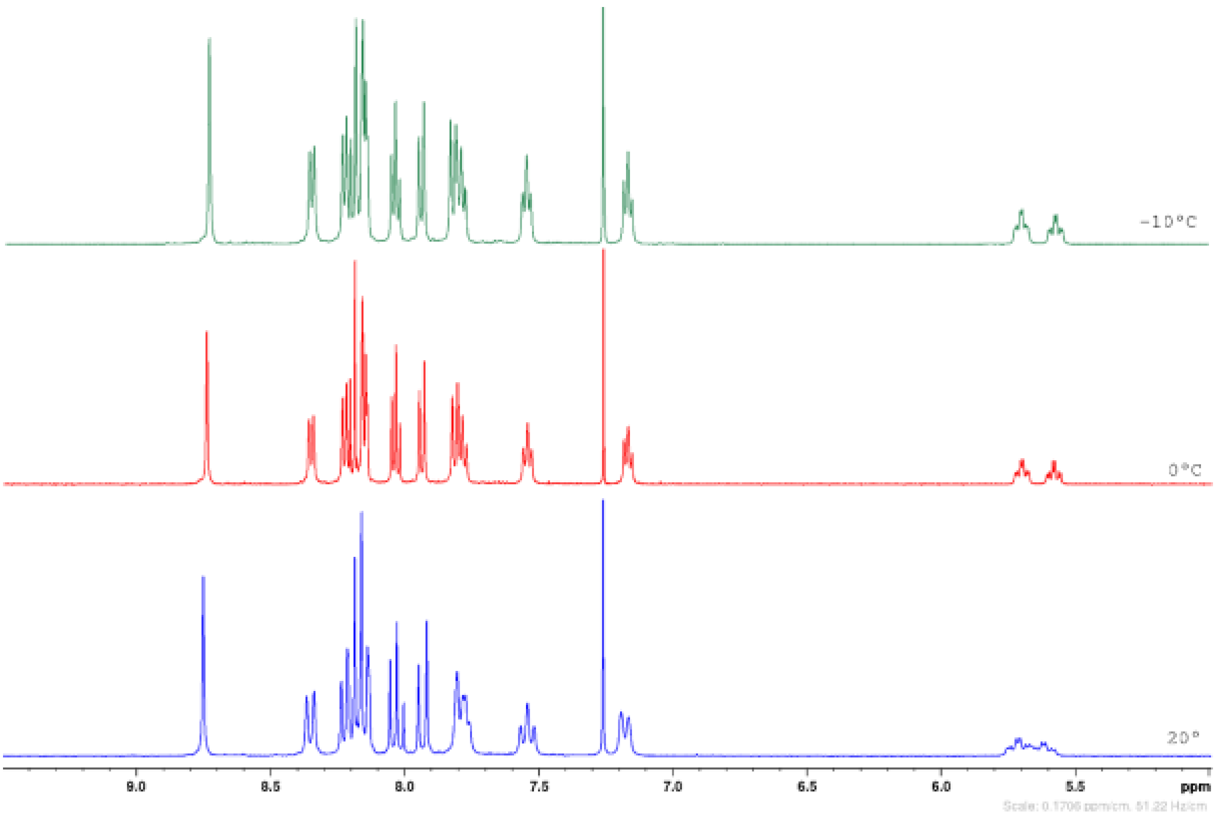

^{1}H NMR ($CDCl_3$, 500 Hz) of **5e** at 20 °C (blue), 0 °C (red) and -10 °C (green) from 6.0 to 0.0 ppm.

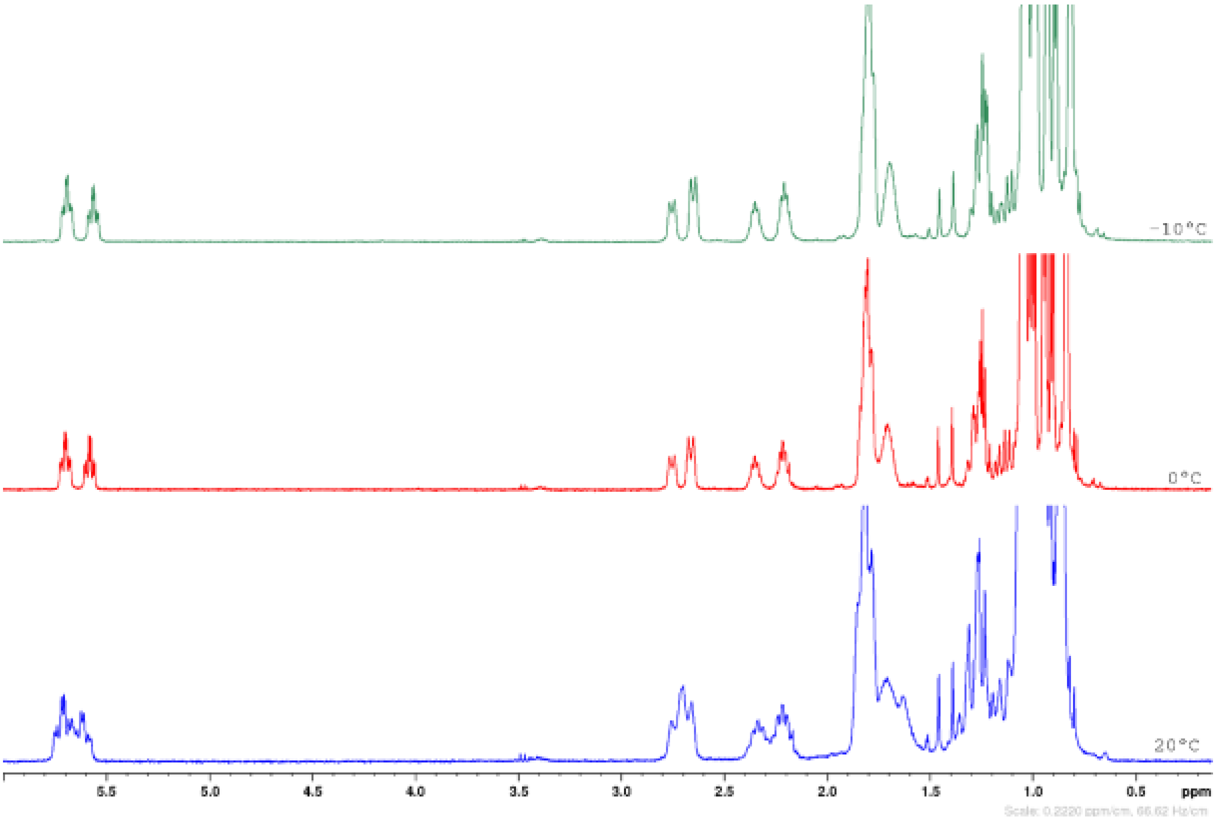

^{1}H NMR (DMSO, 500 Hz) of **5e** at 20 °C (blue) and 60 °C (red).

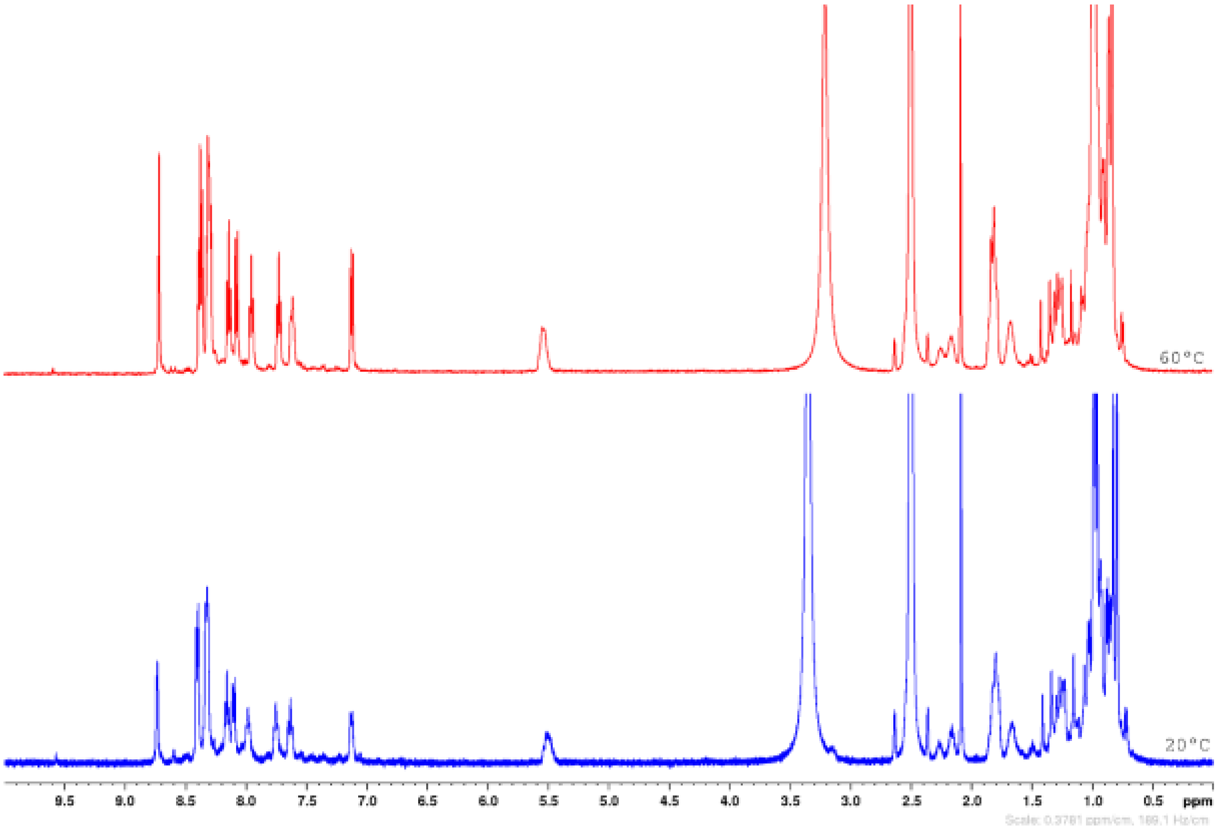

^{13}C NMR, decoupled ($CDCl_3$, 125 Hz) of **5e** at -10 °C.

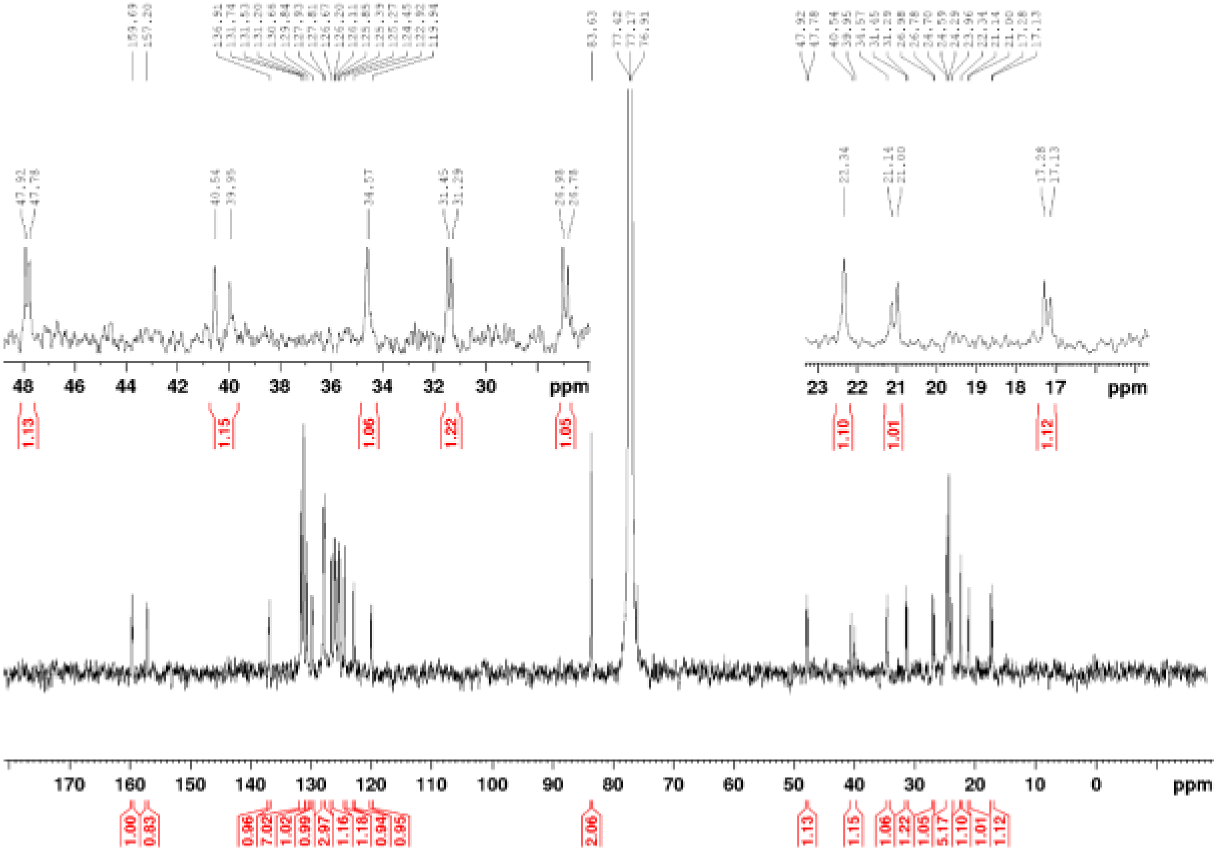

$^{13}C\{^1H\}$ NMR (DMSO, 125 Hz) of **5e** at 60 °C.

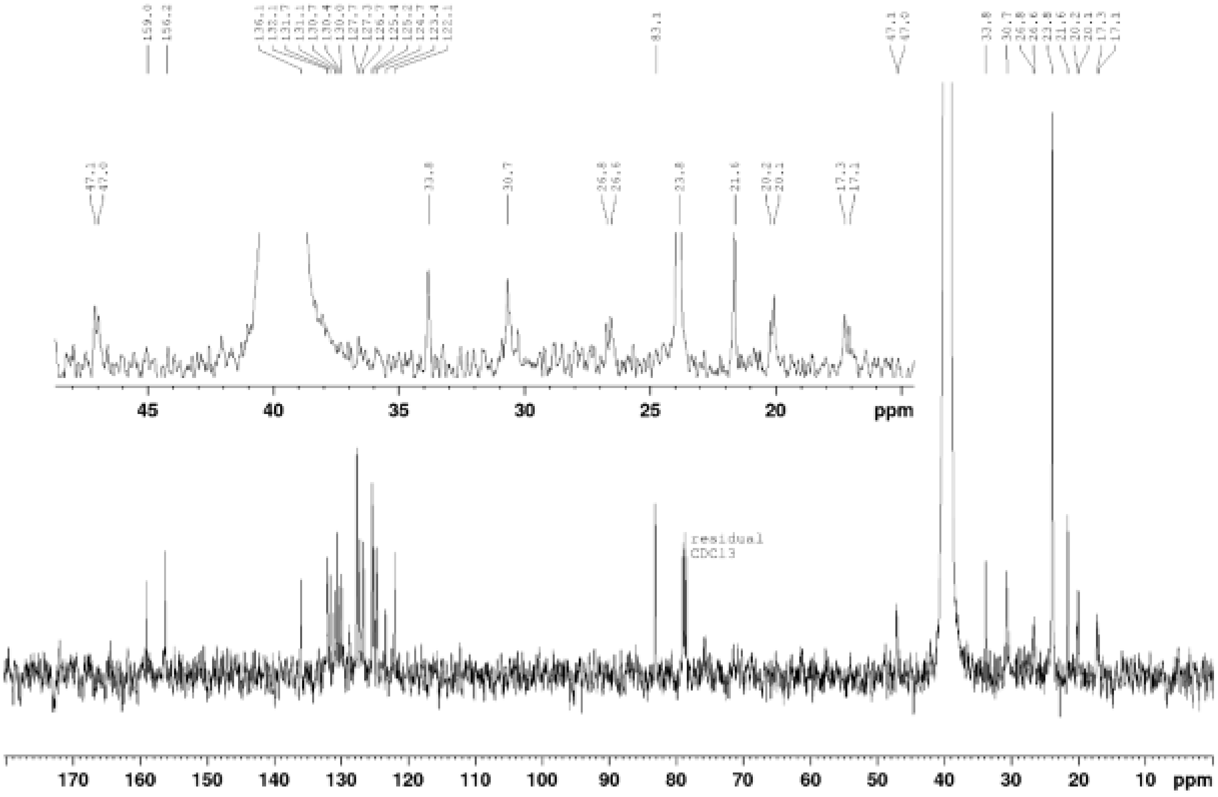

HSQC ($CDCl_3$, 125 Hz) of **5e** at -10 °C of alkylic region

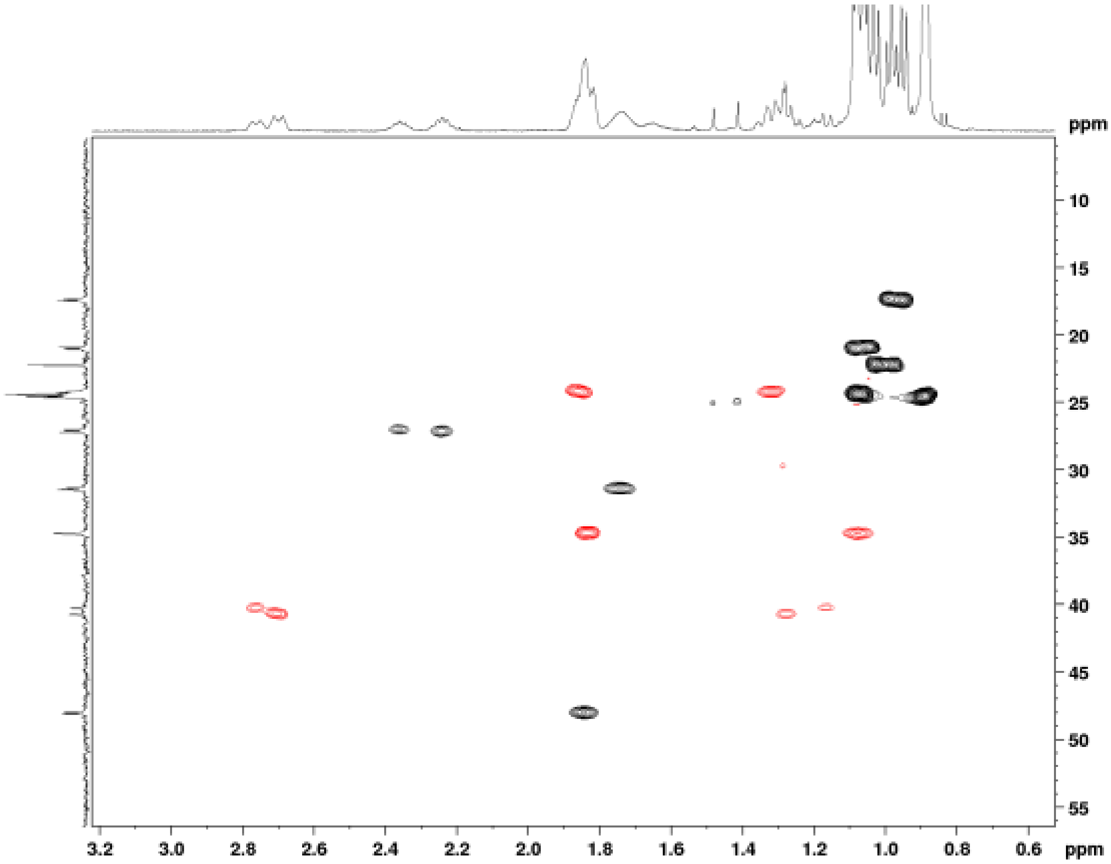

HSQC ($CDCl_3$, 125 Hz) of **5e** at -10 °C

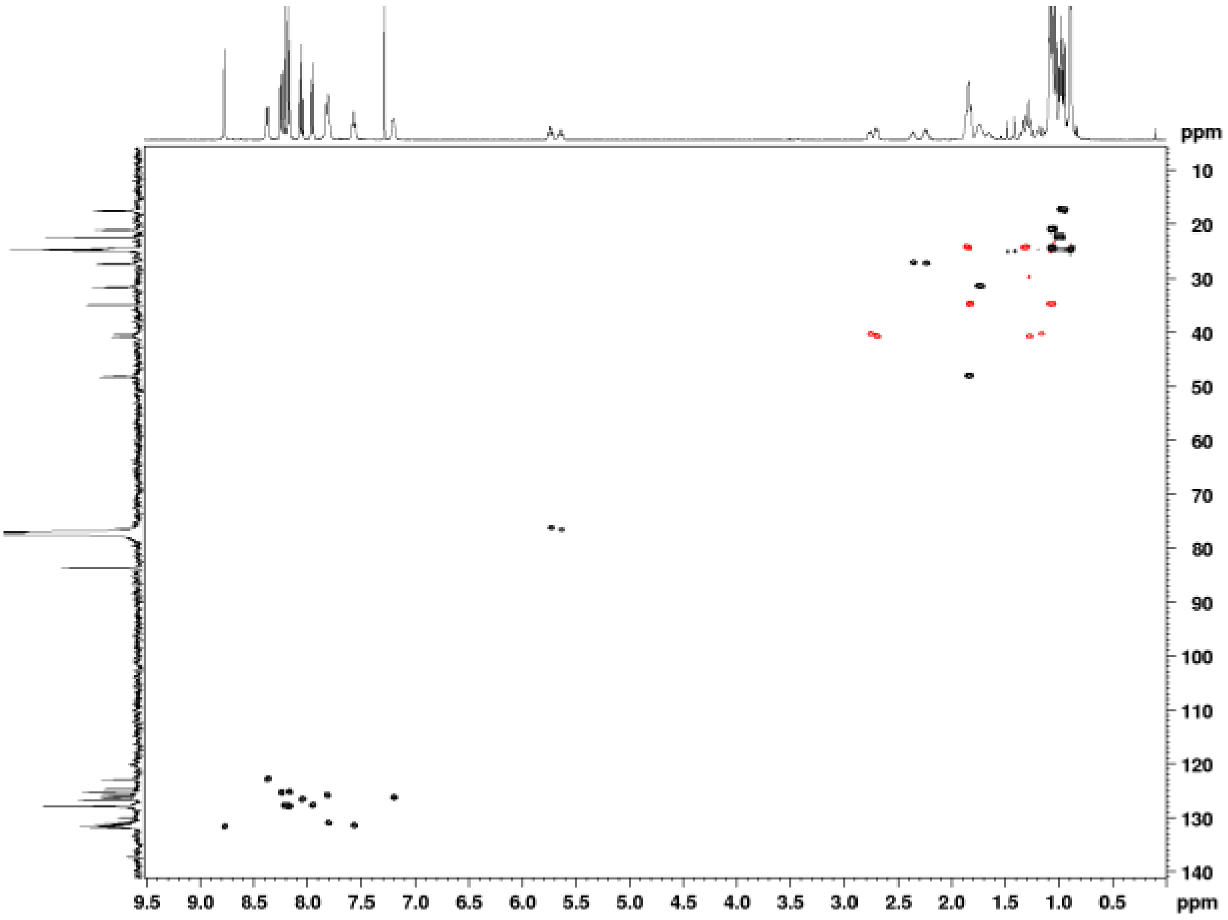

^{1}H NMR ($CDCl_3$, 300 Hz) of **5f**

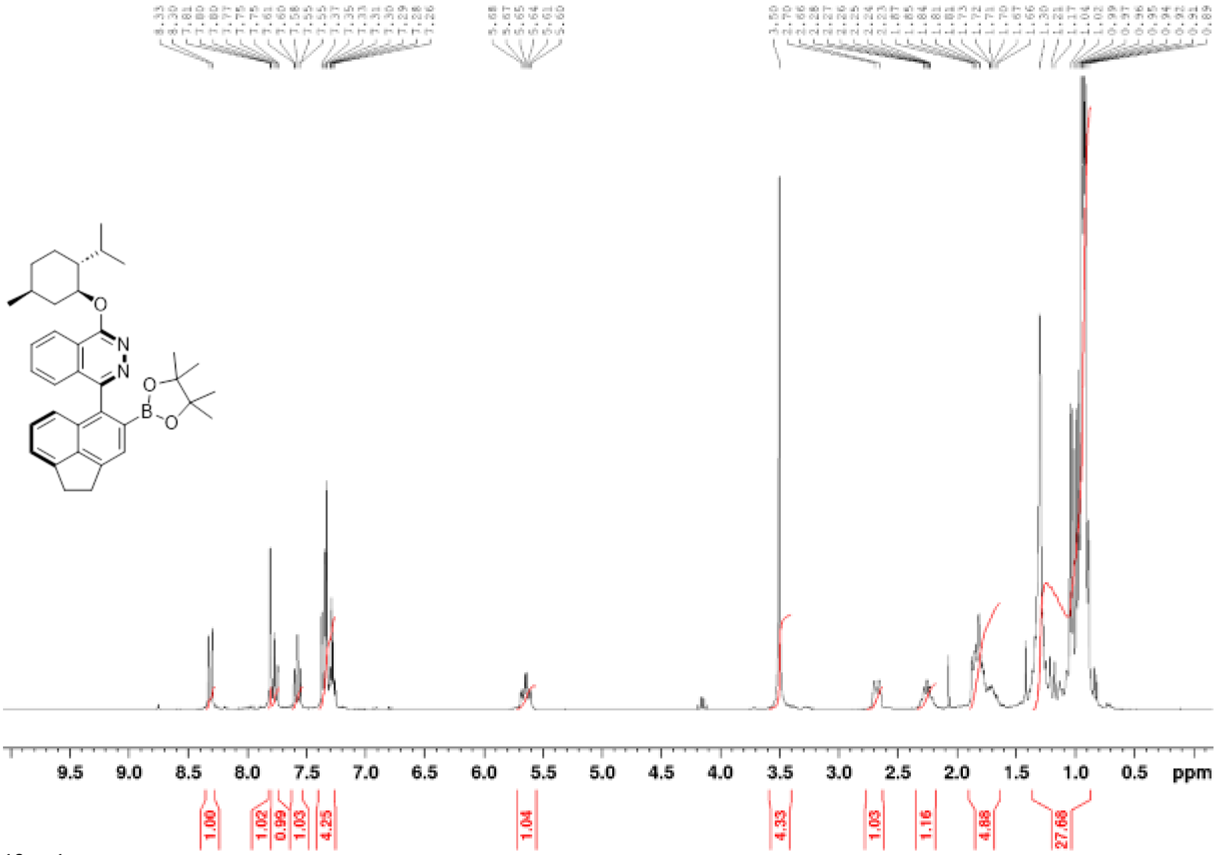

$^{13}C\{^{1}H\}$ NMR ($CDCl_3$, 125 Hz) of **5f**

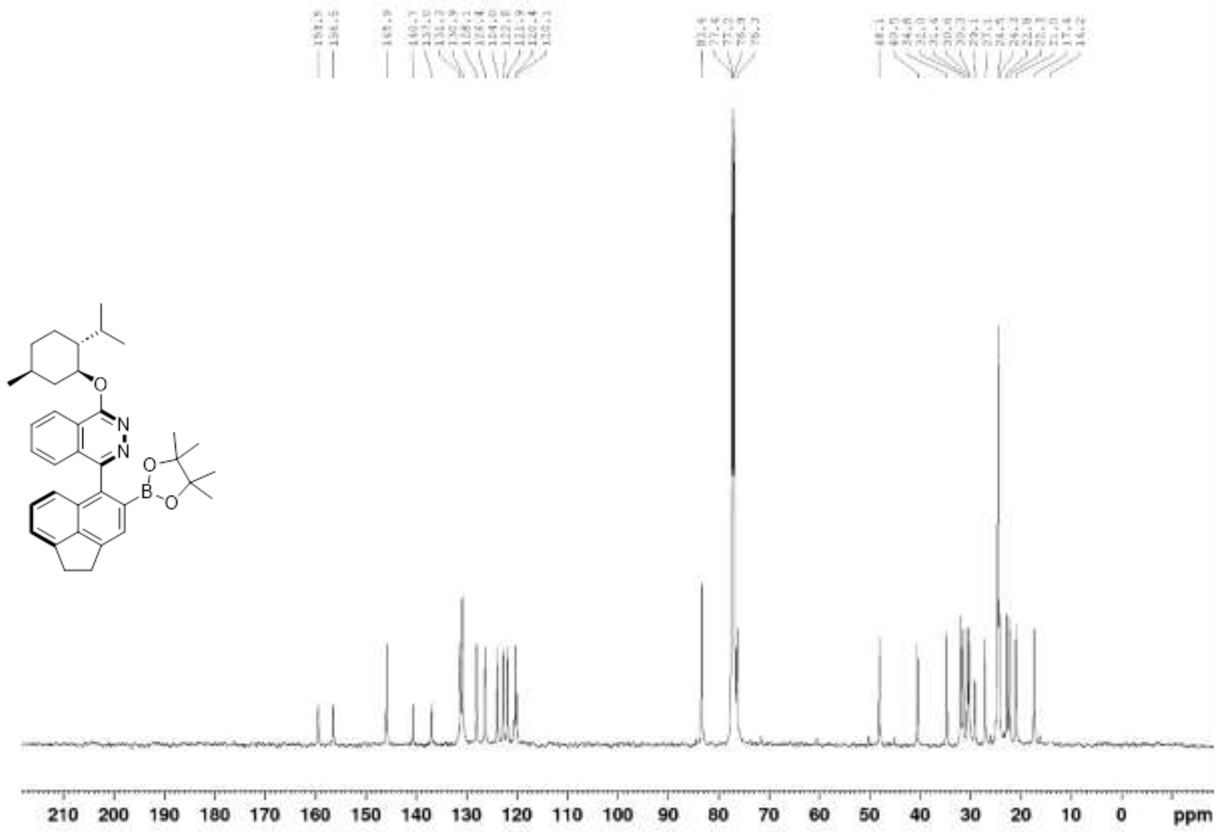

^{1}H NMR ($CDCl_3$, 300 Hz) of **5g**

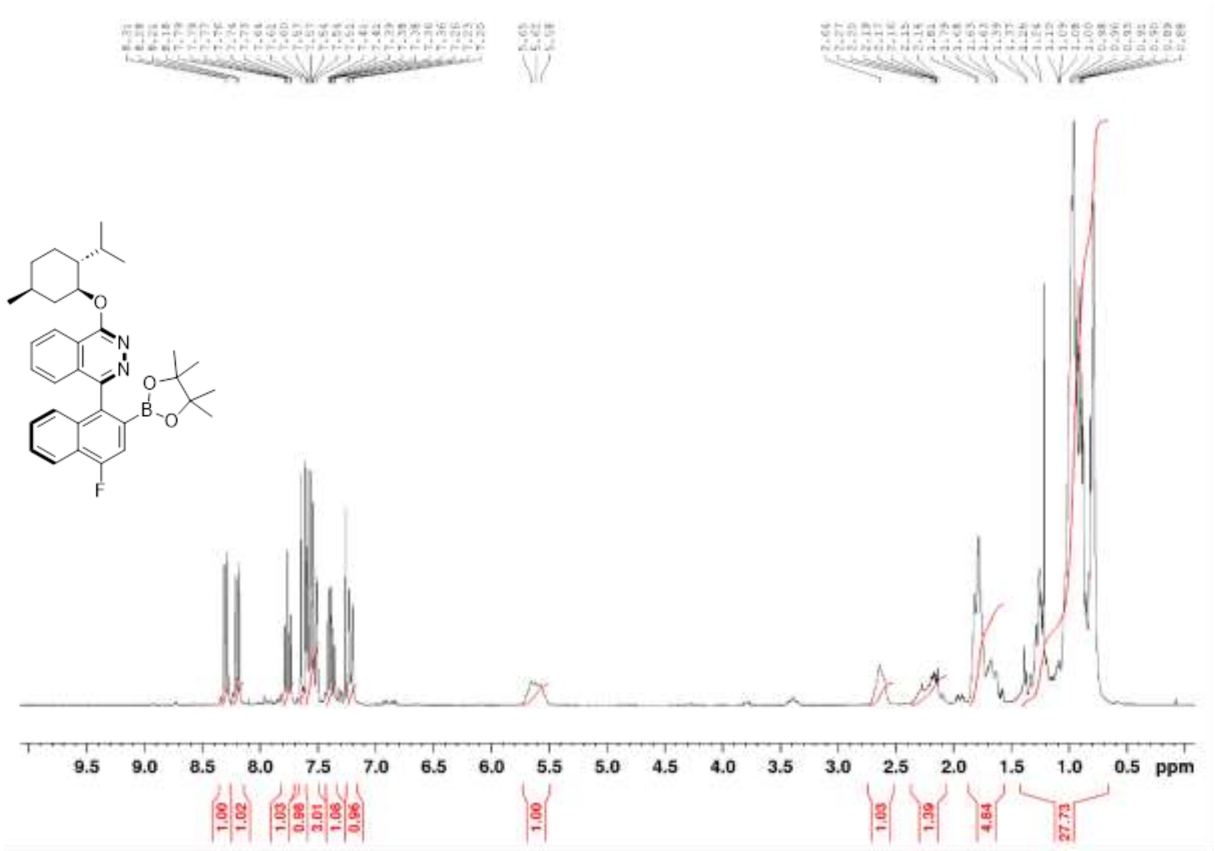

$^{13}C\{^{1}H\}$ NMR ($CDCl_3$, 125 Hz) of **5g**

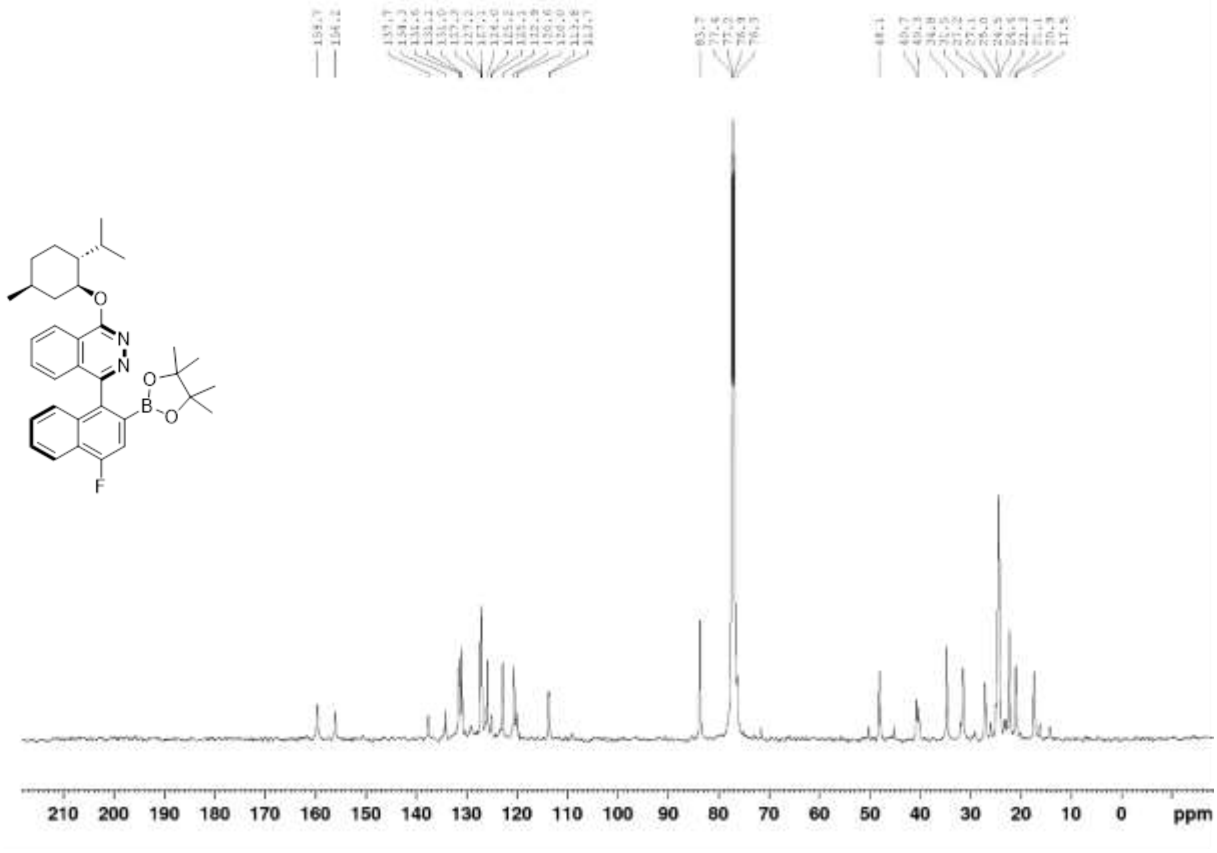

^{19}F NMR ($CDCl_3$, 282 Hz) of **5g**

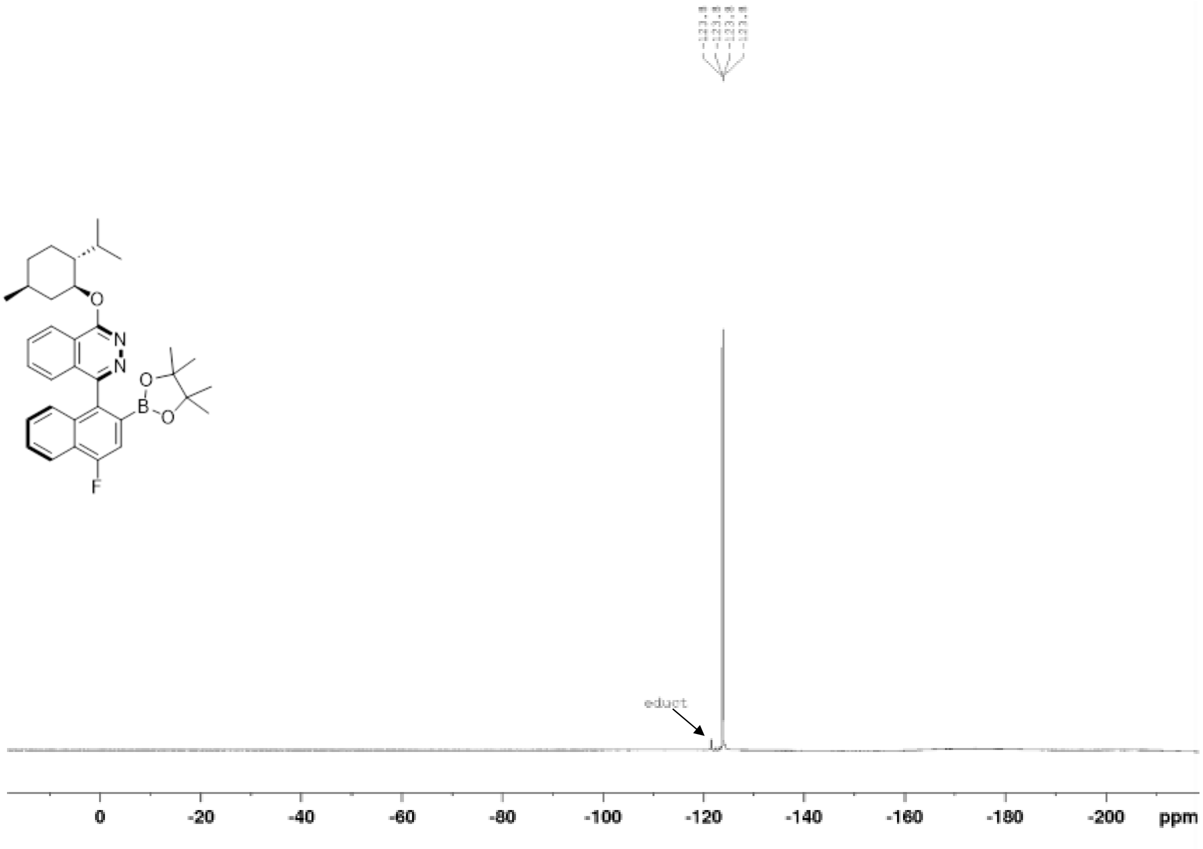

^{1}H NMR ($CDCl_3$, 300 Hz) of **5h**

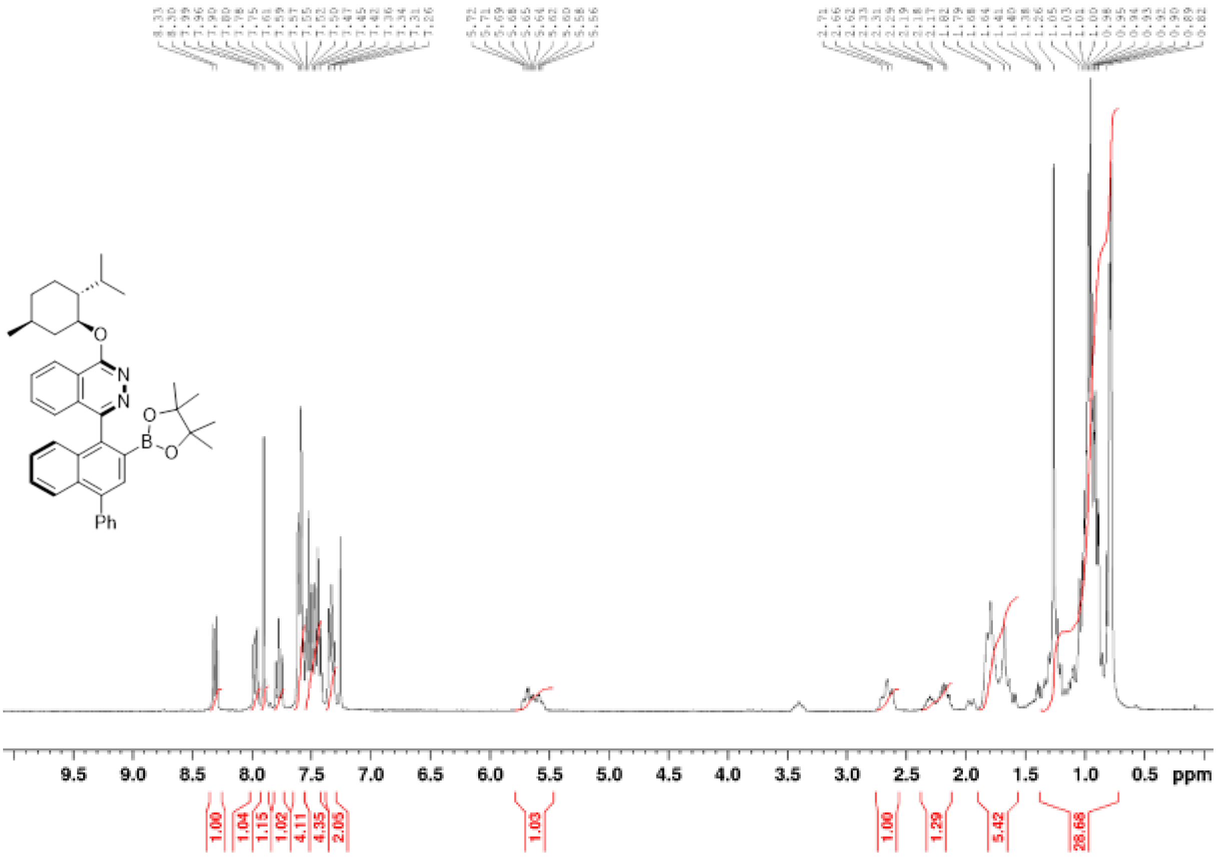

$^{13}C\{^{1}H\}$ NMR ($CDCl_3$, 125 Hz) of **5h**

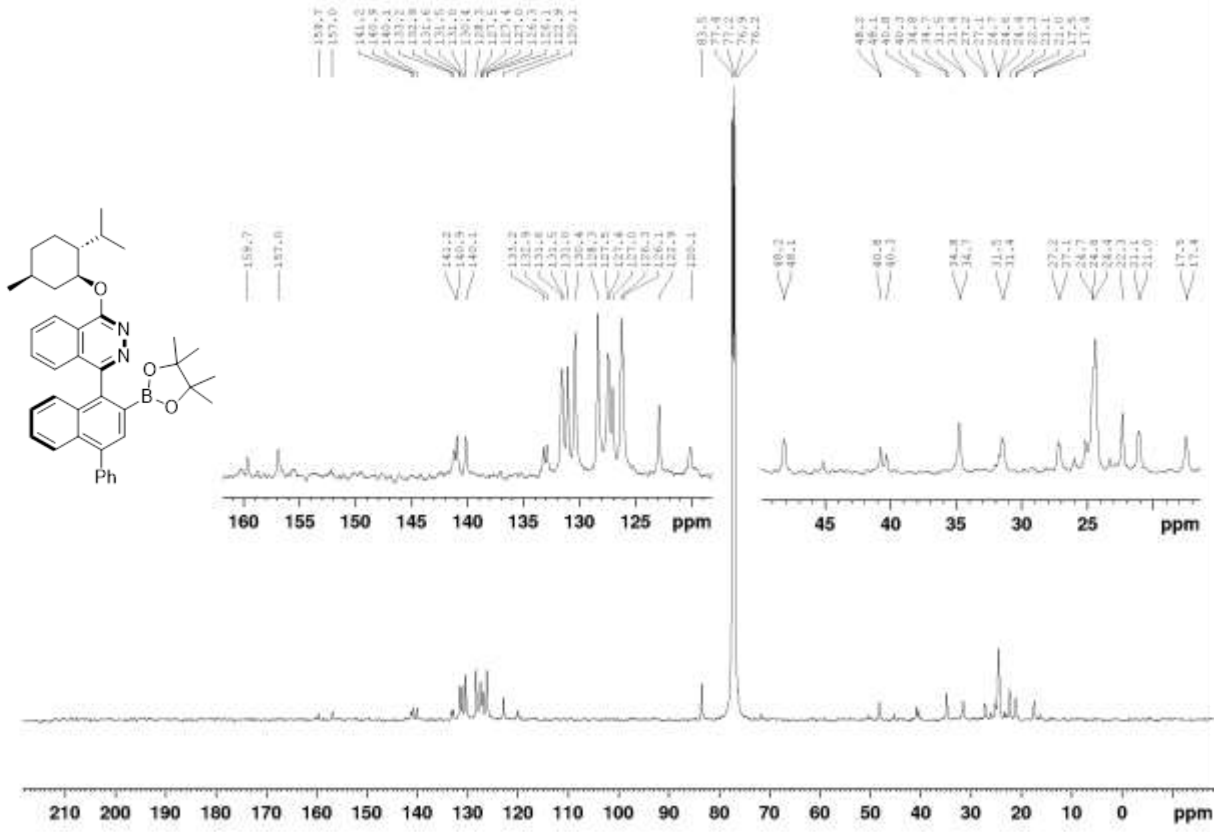

^{1}H NMR ($CDCl_3$, 300 Hz) of **5i**

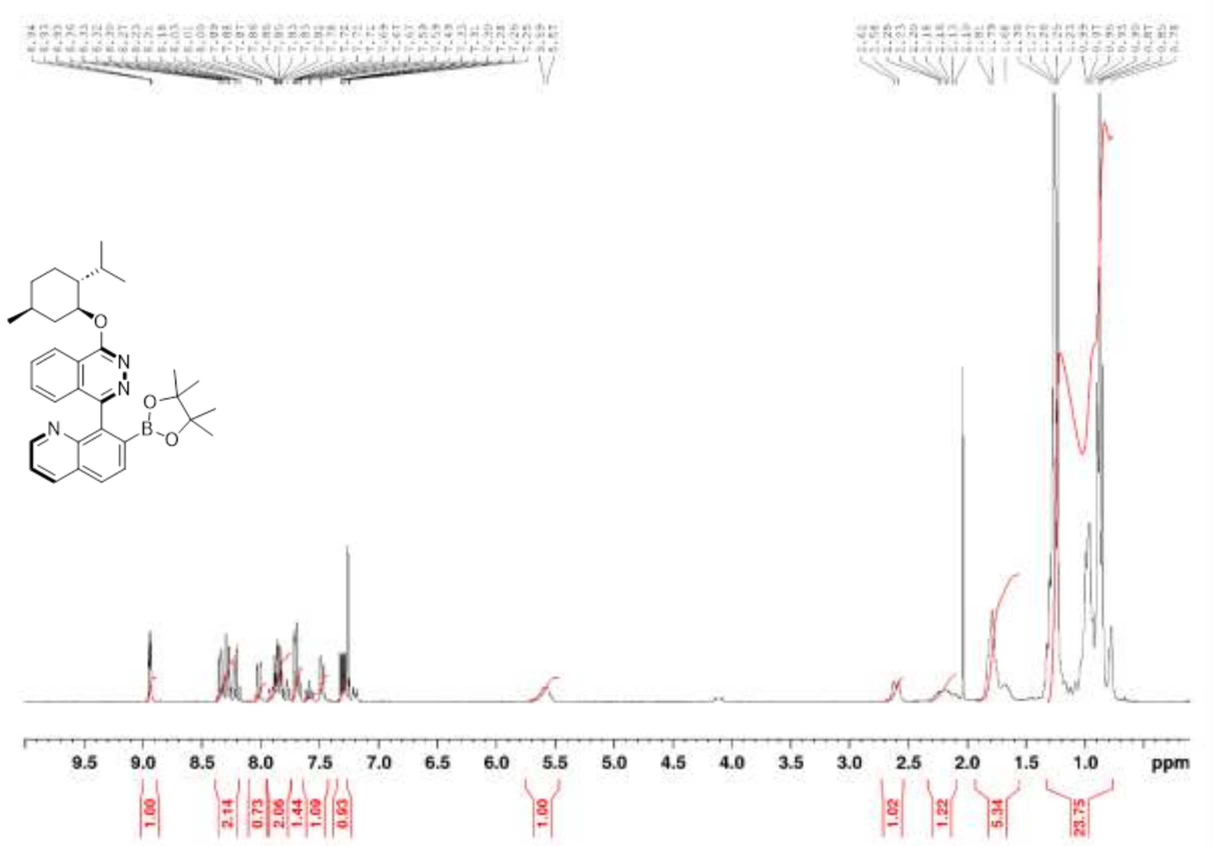

$^{13}C\{^{1}H\}$ NMR ($CDCl_3$, 125 Hz) of **5i**

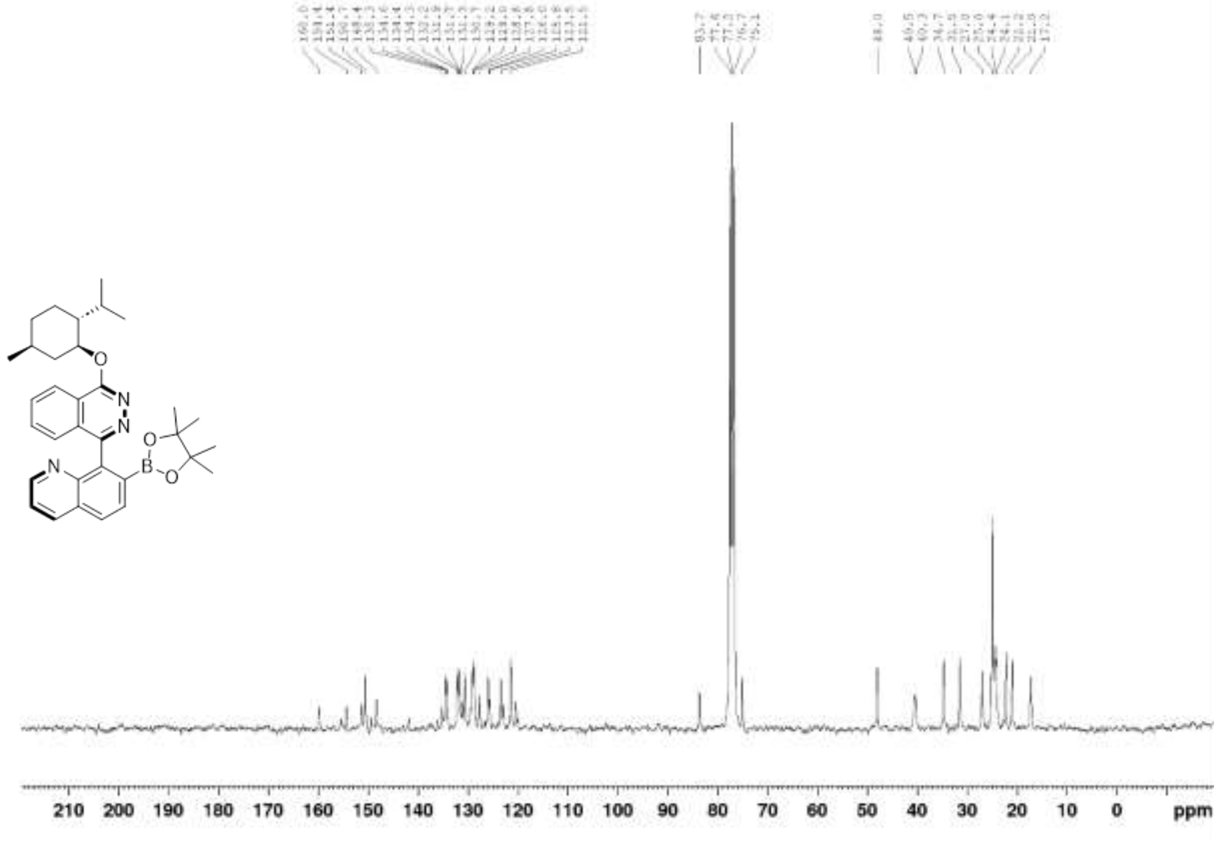

^{1}H NMR ($CDCl_3$, 300 Hz) of **5j**

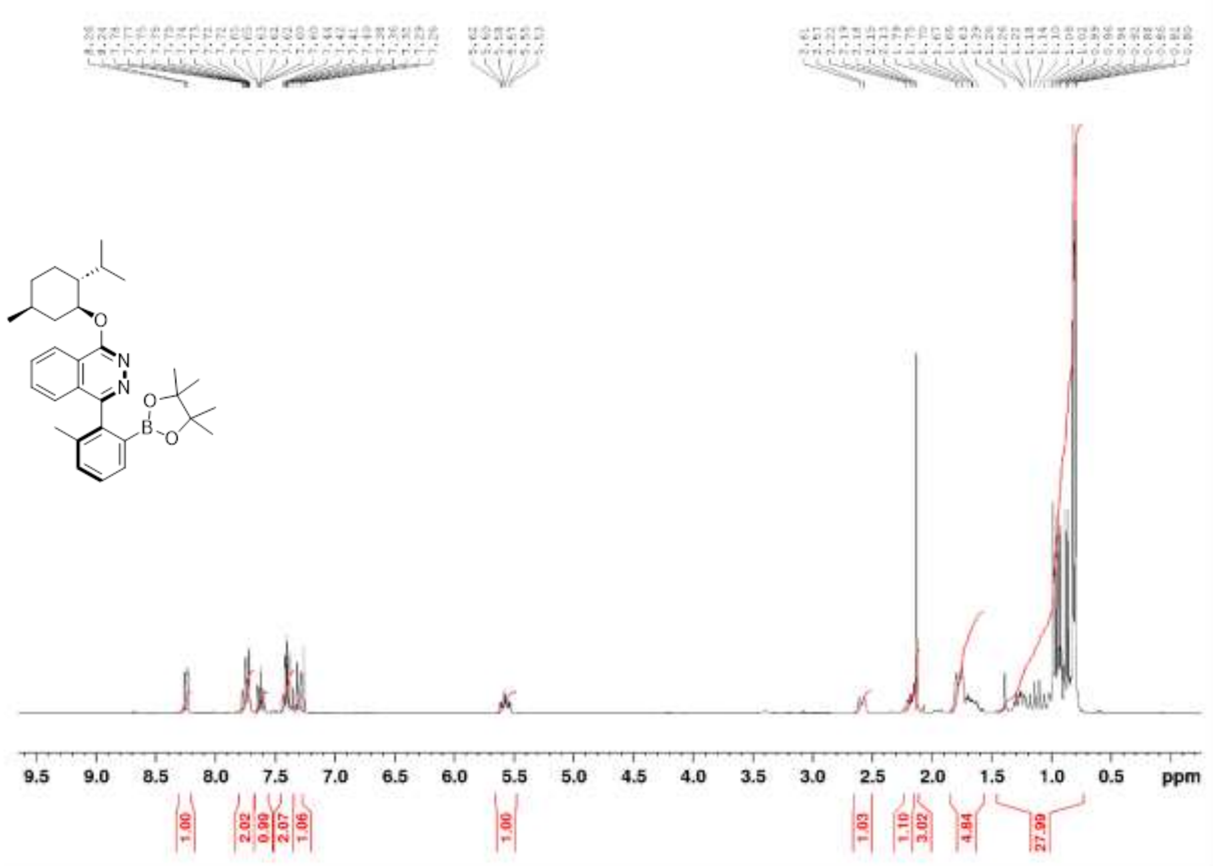

$^{13}C\{^{1}H\}$ NMR ($CDCl_3$, 125 Hz) of **5j**

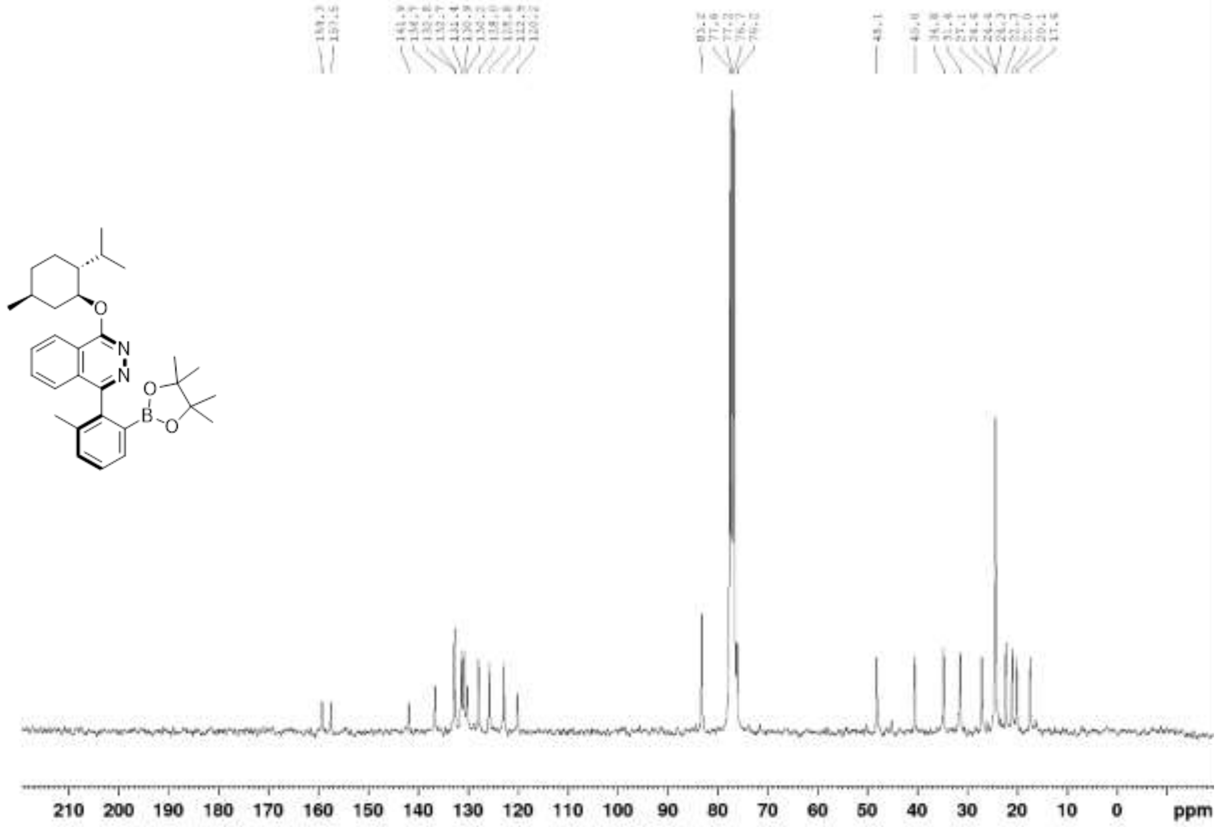

^{1}H NMR ($CDCl_3$, 300 Hz) of **5k**

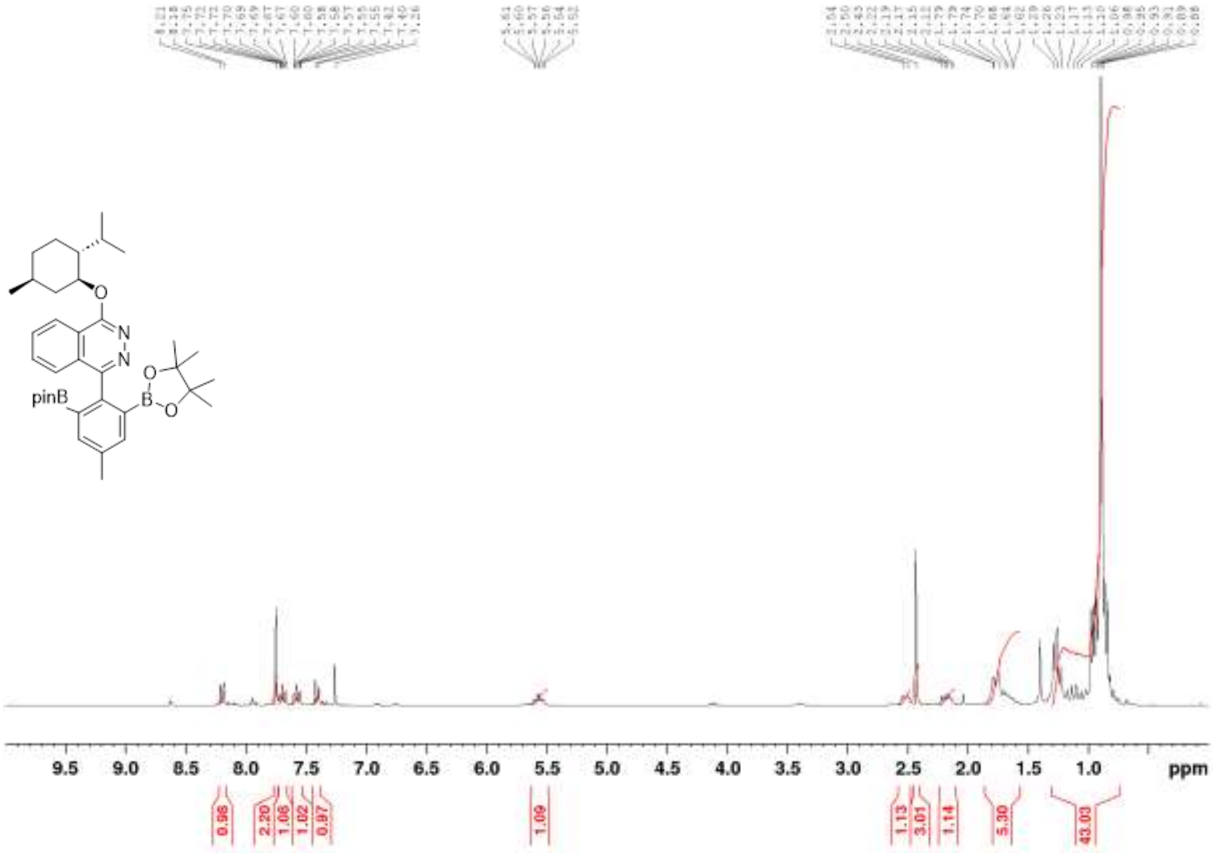

$^{13}C\{^{1}H\}$ NMR ($CDCl_3$, 125 Hz) of **5k**

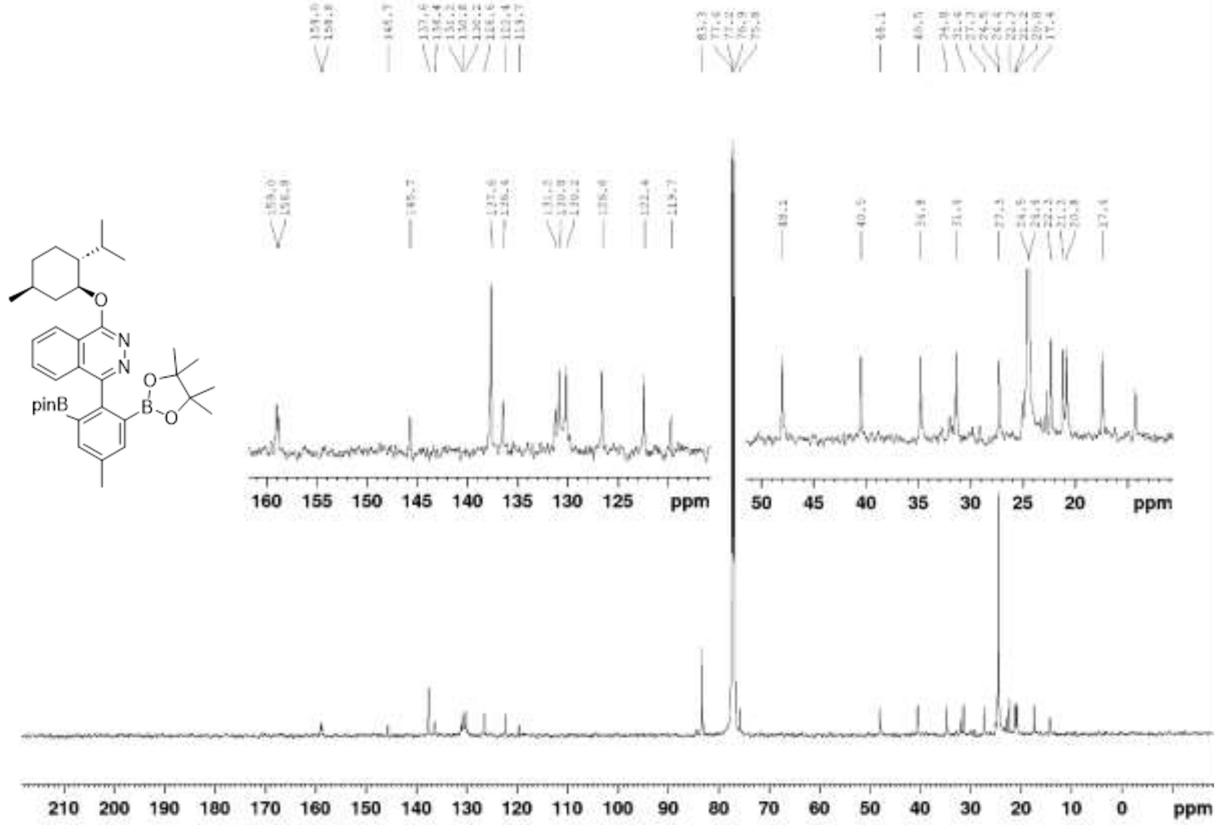

^{1}H NMR ($CDCl_3$, 300 Hz) of **5m-SI**

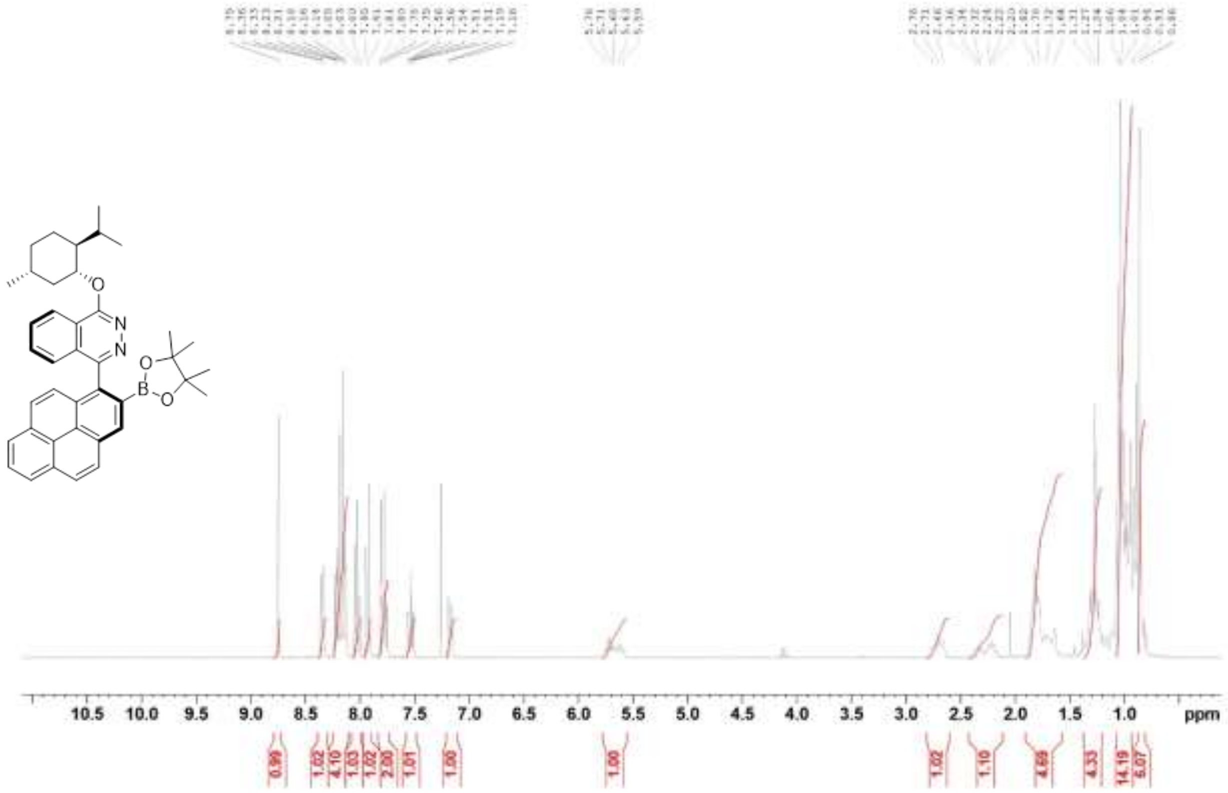

$^{13}C\{^{1}H\}$ NMR ($CDCl_3$, 125 Hz) of **5m-SI**

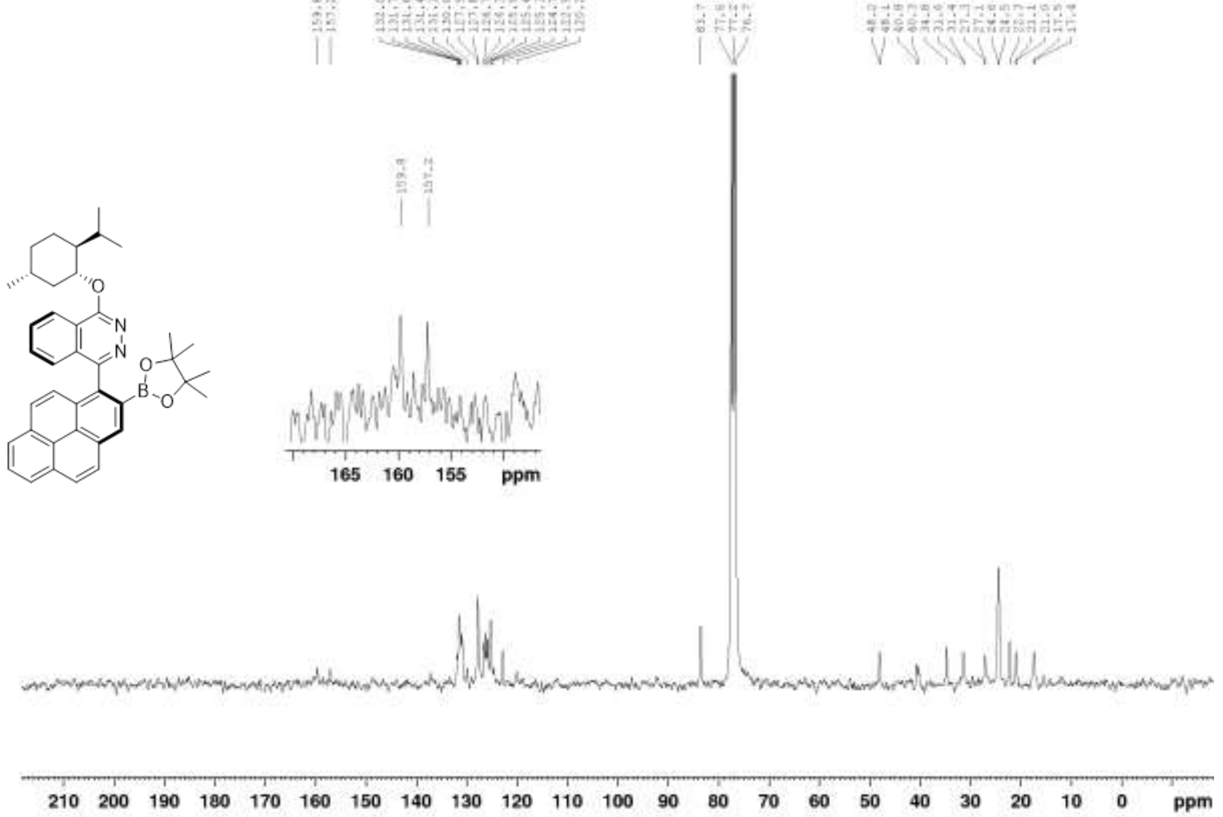

^{1}H NMR ($CDCl_3$, 300 Hz) of **5n-SI**

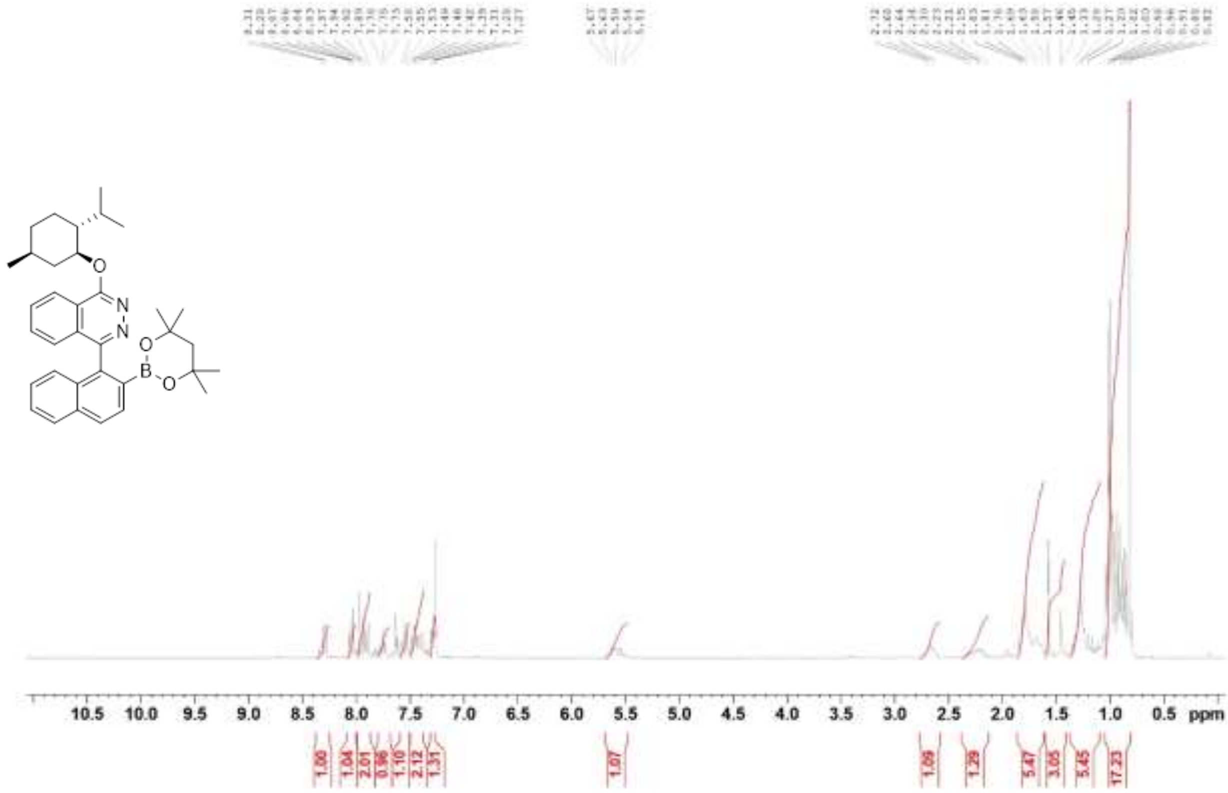

$^{13}C\{^{1}H\}$ NMR ($CDCl_3$, 125 Hz) of **5n-SI**

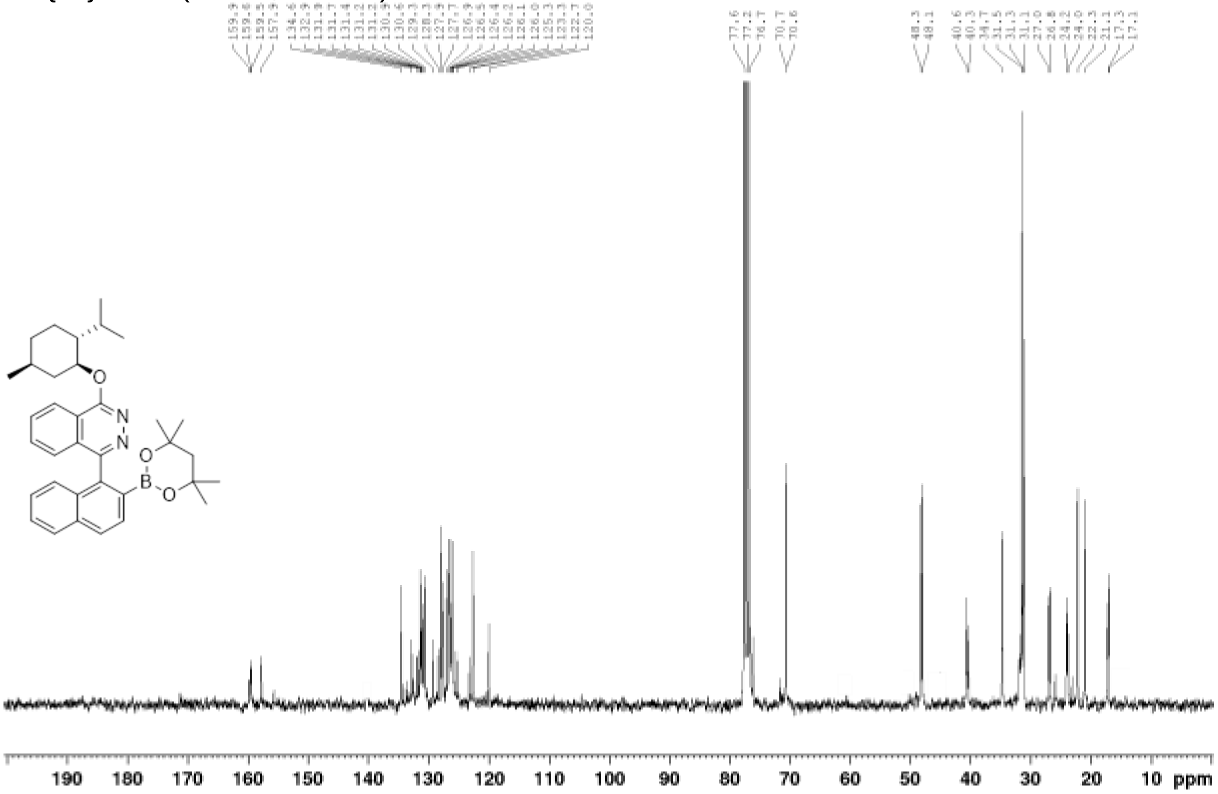

^{1}H NMR ($CDCl_3$, 300 Hz) of **5o-SI**

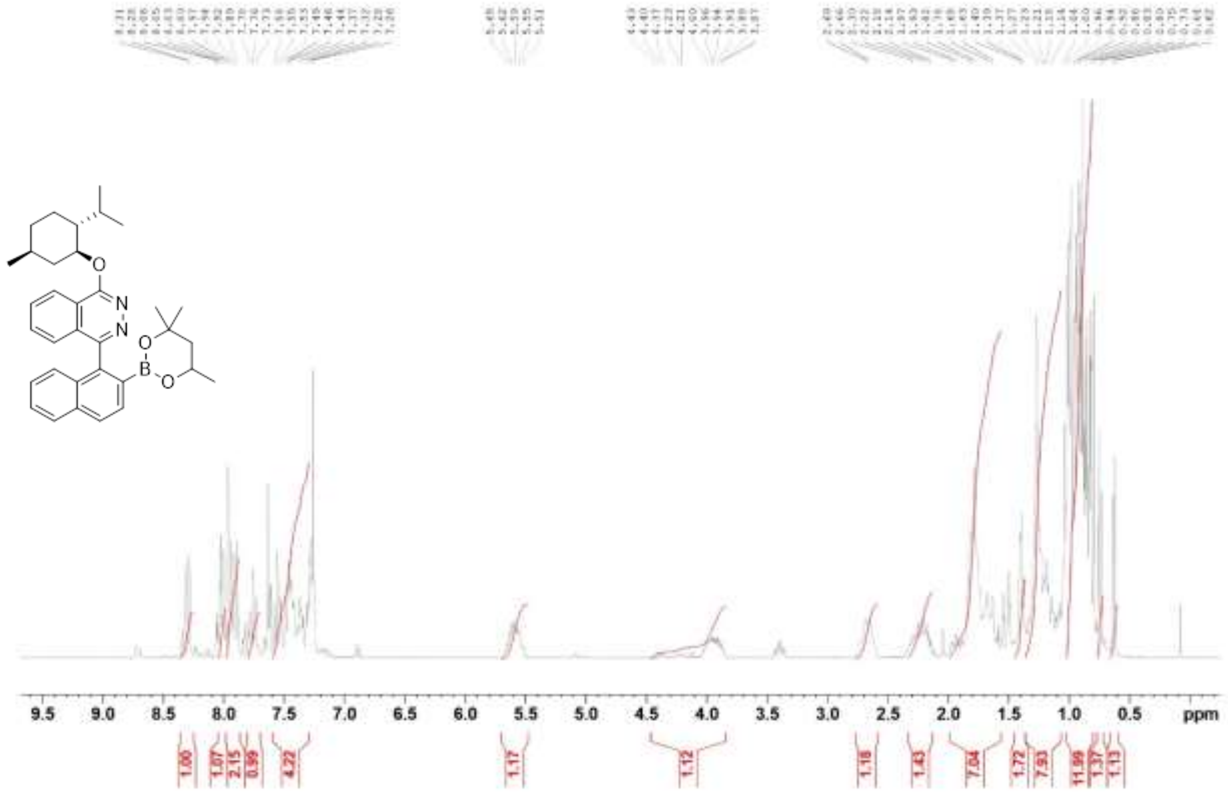

^{13}C{^{1}H} NMR ($CDCl_3$, 125 Hz) of **5o-SI**

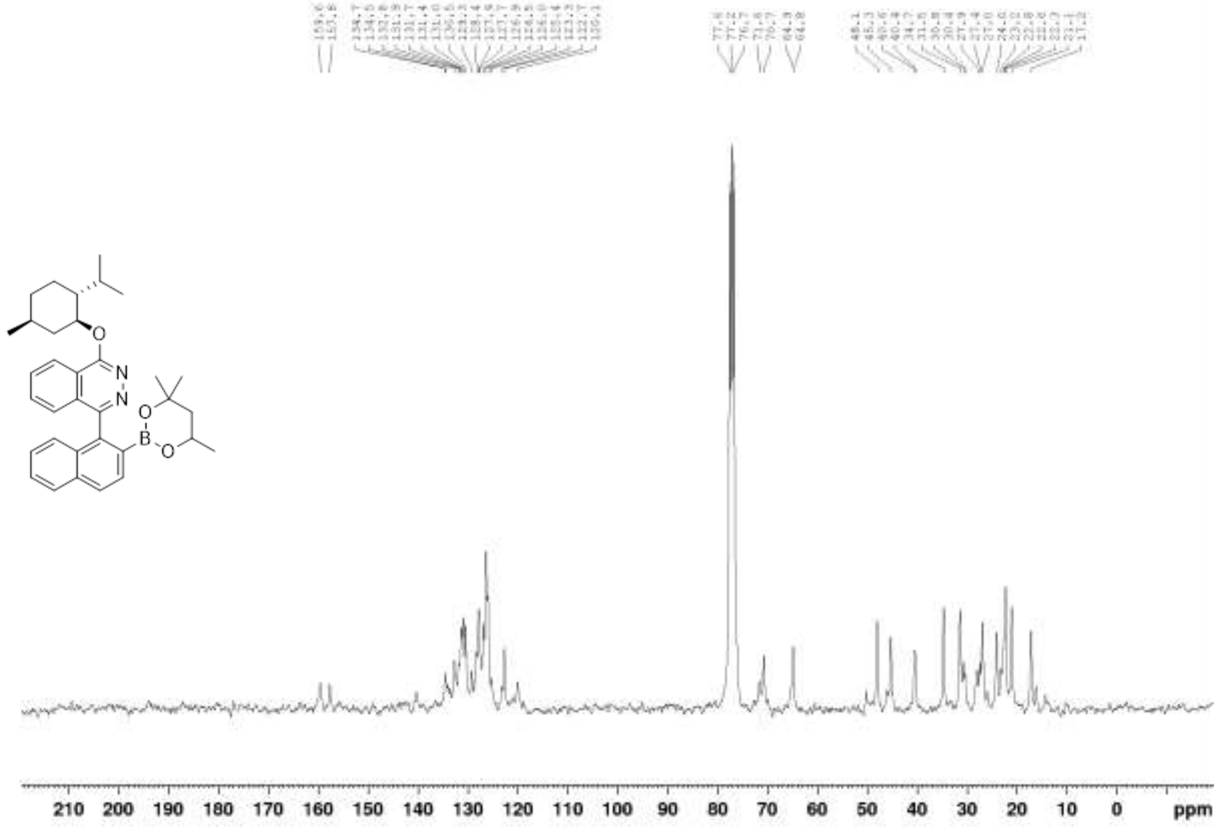

^{1}H NMR ($CDCl_3$, 300 Hz) of **5p-SI**

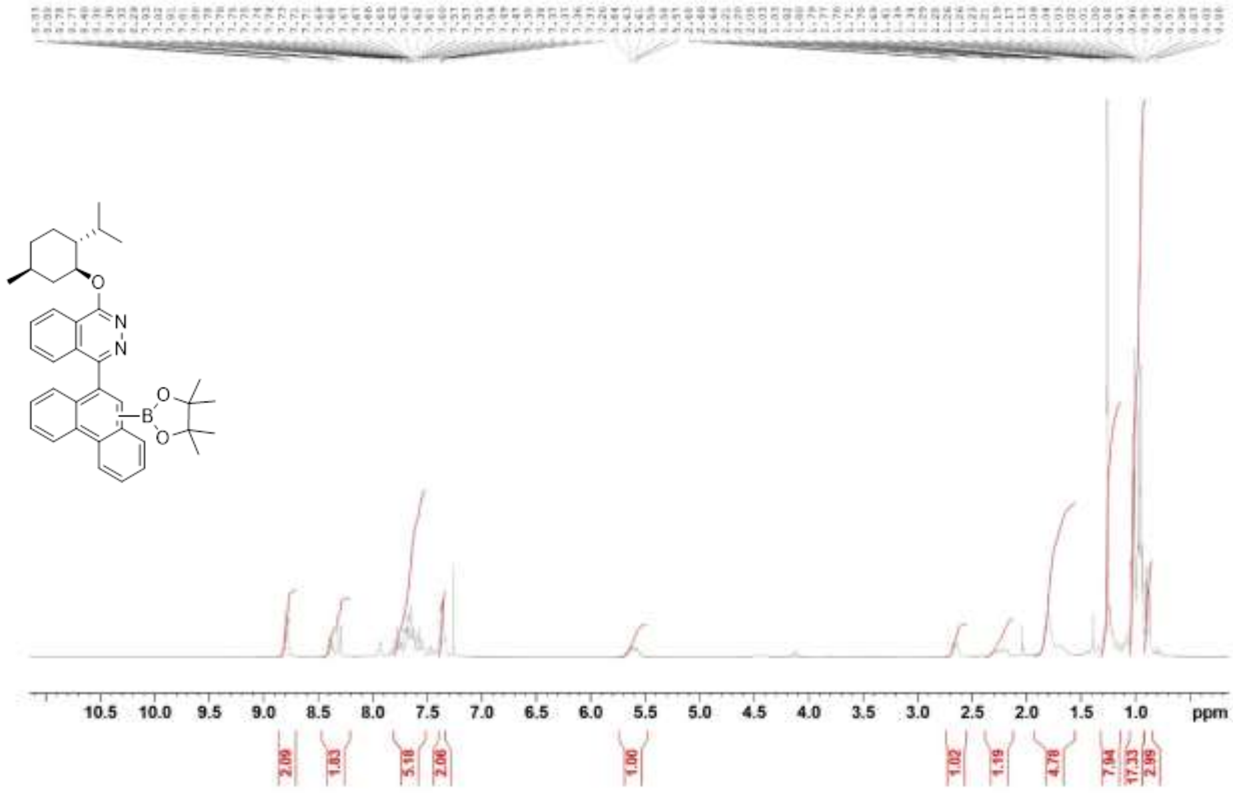

$^{13}C\{^{1}H\}$ NMR ($CDCl_3$, 125 Hz) of **5p-SI**

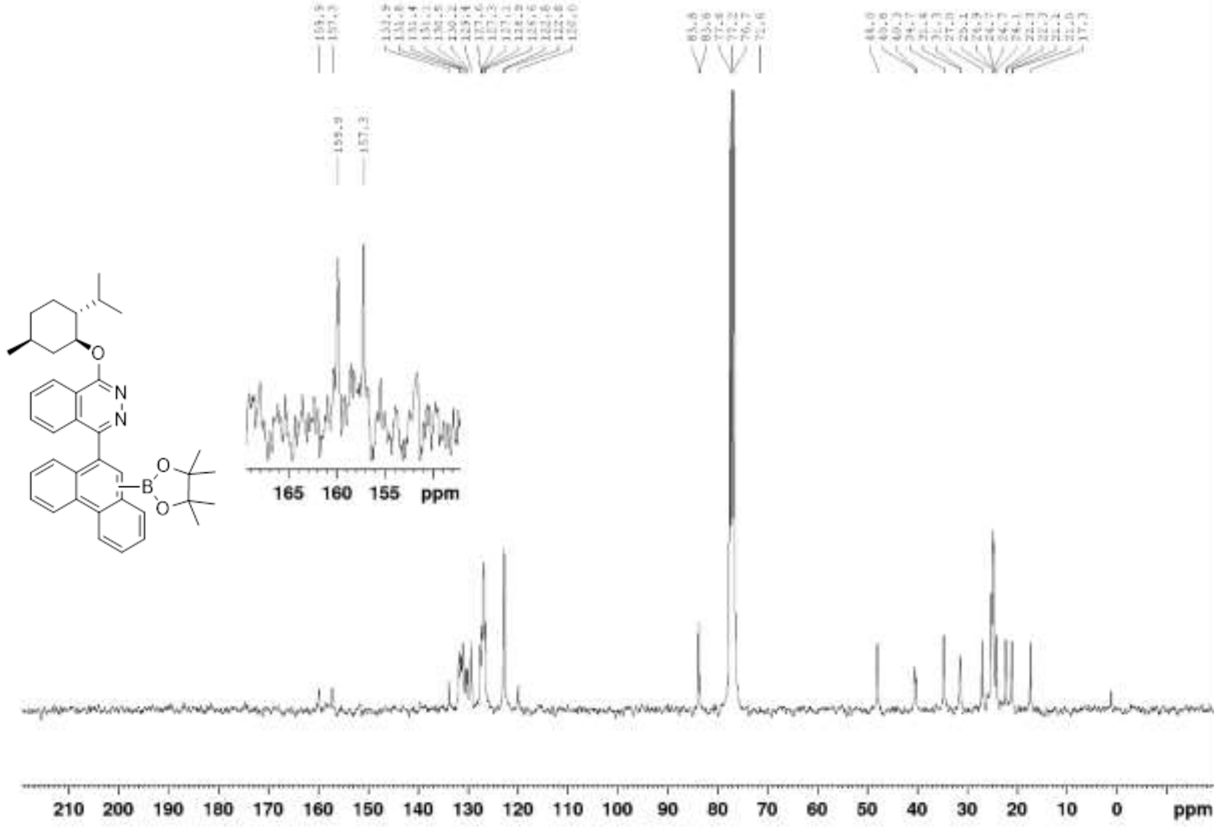

^{1}H NMR ($CDCl_3$, 300 Hz) of **5q-SI**

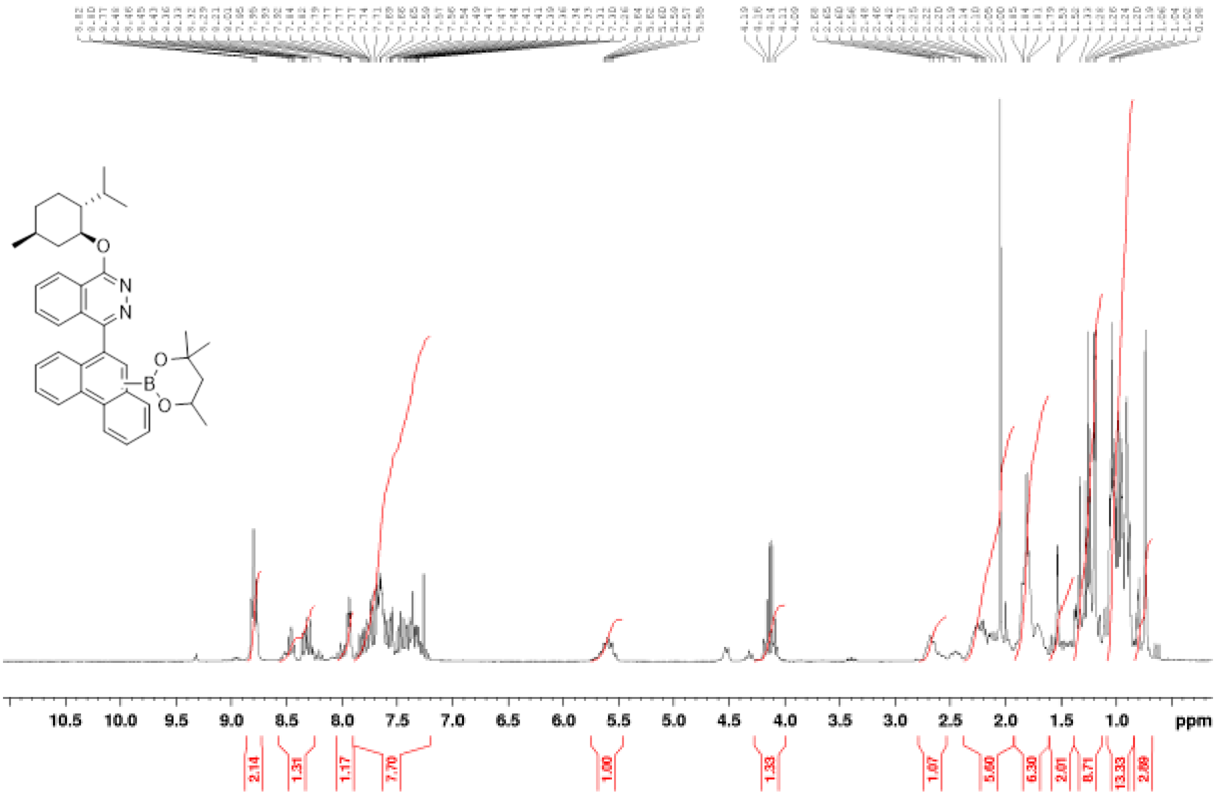

$^{13}C\{^{1}H\}$ NMR ($CDCl_3$, 125 Hz) of **5q-SI**

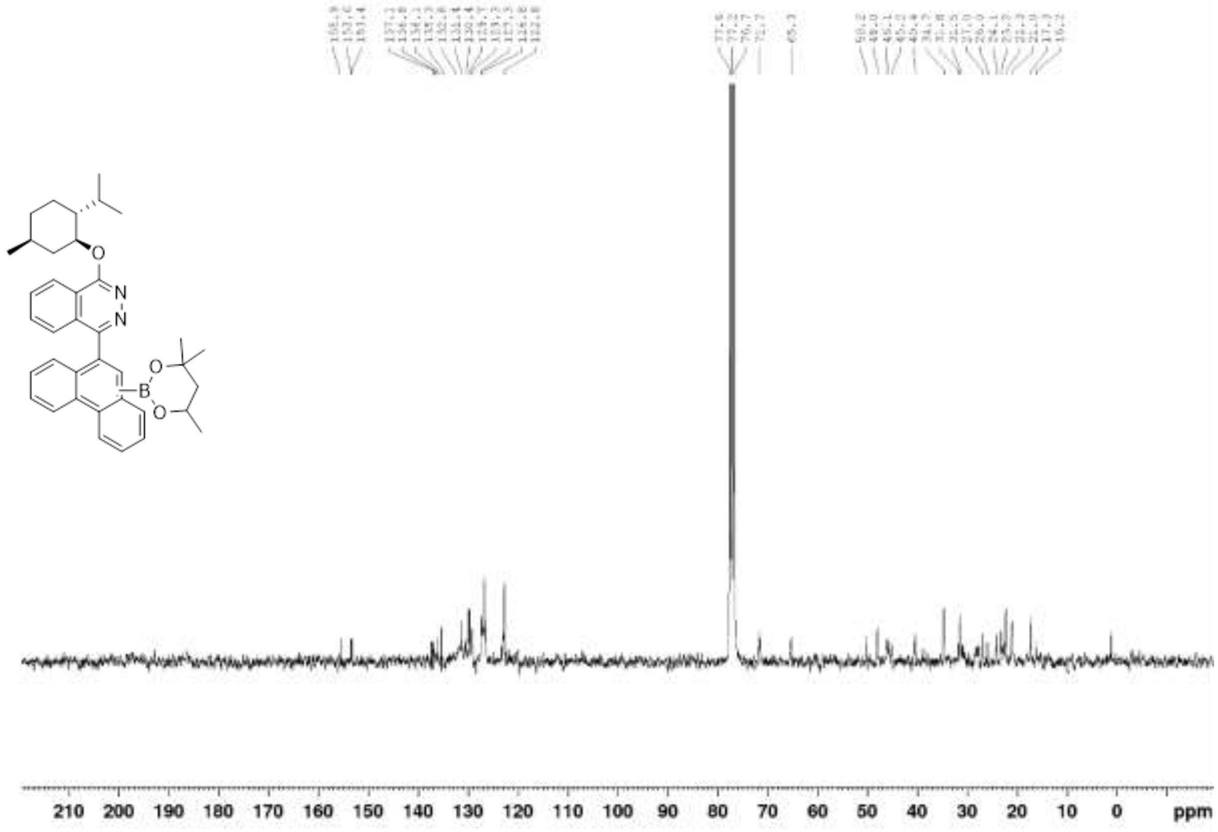

^{1}H NMR ($CDCl_3$, 300 Hz) of (R_a)-**6a** from **4g**

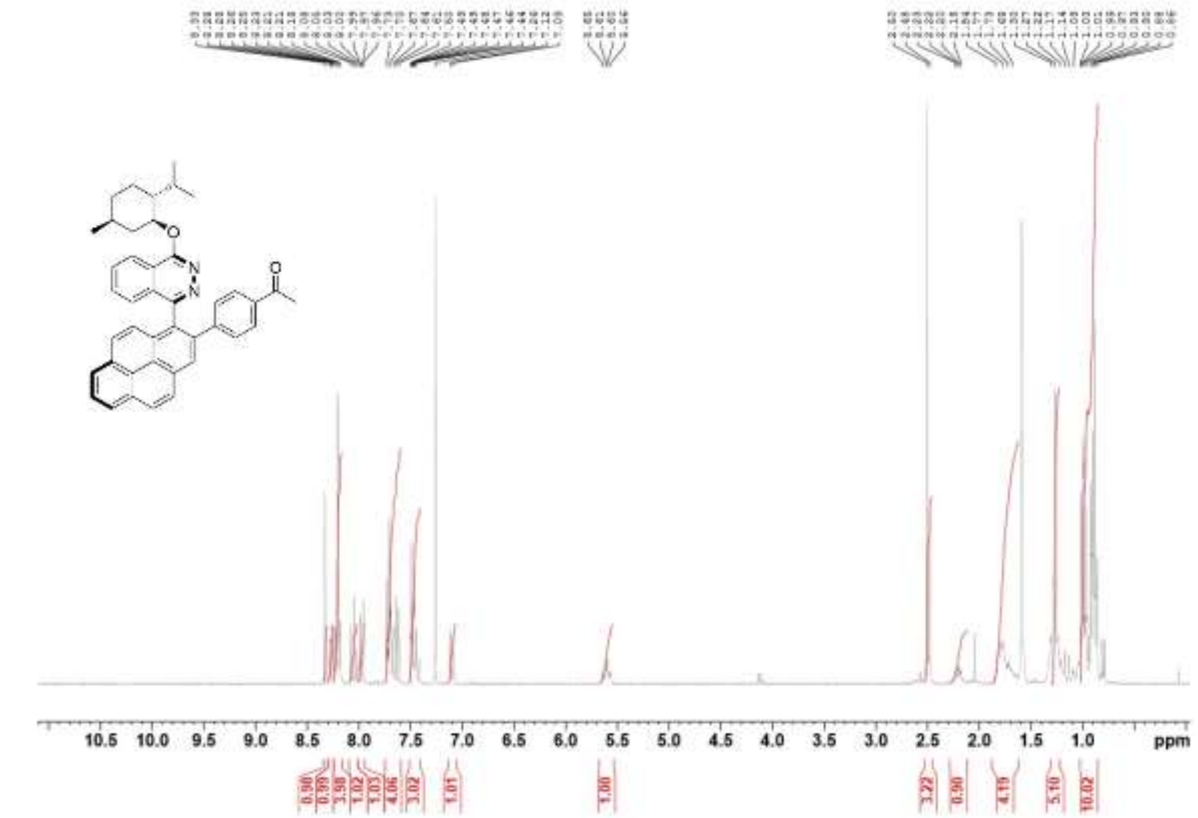

$^{13}C\{^{1}H\}$ NMR ($CDCl_3$, 125 Hz) of (R_a)-**6a** from **4g**

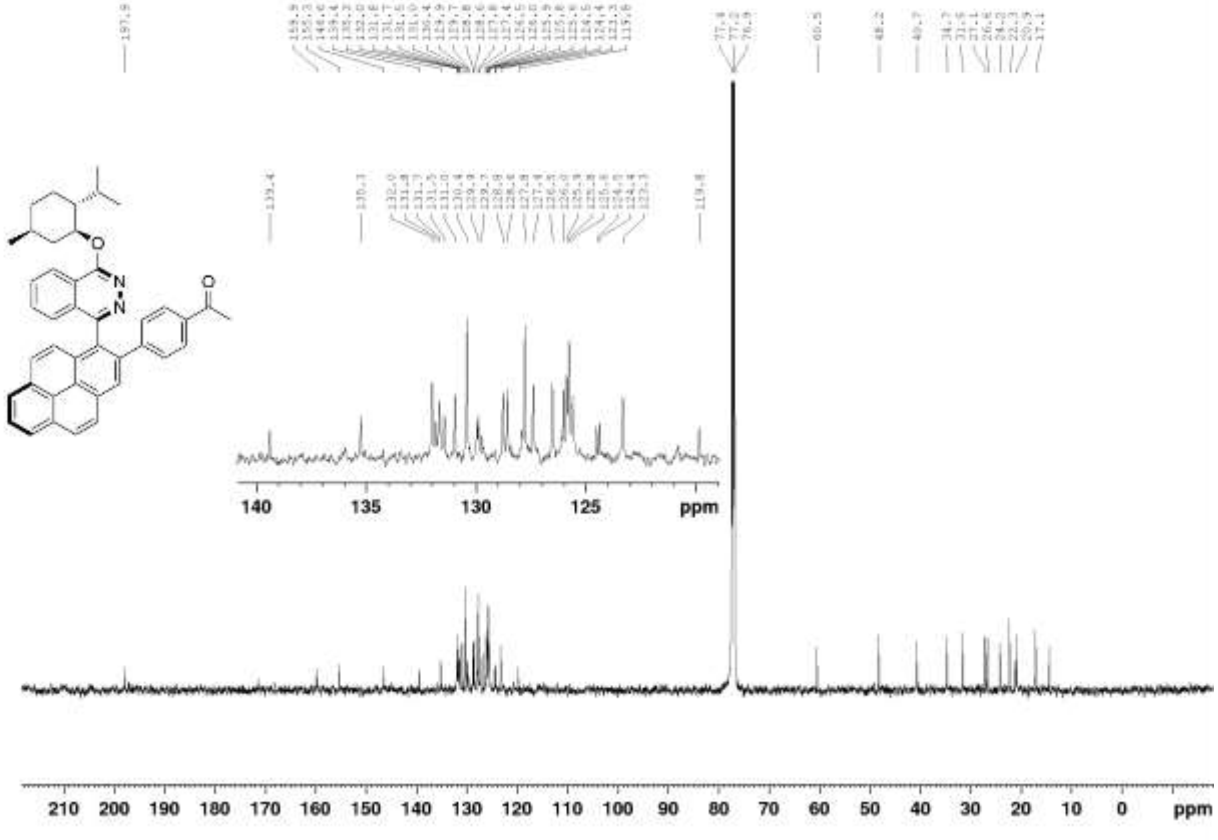

HPLC-chromatogram of (R_a)-**6a** from **4g** (Table 2, entry 3)

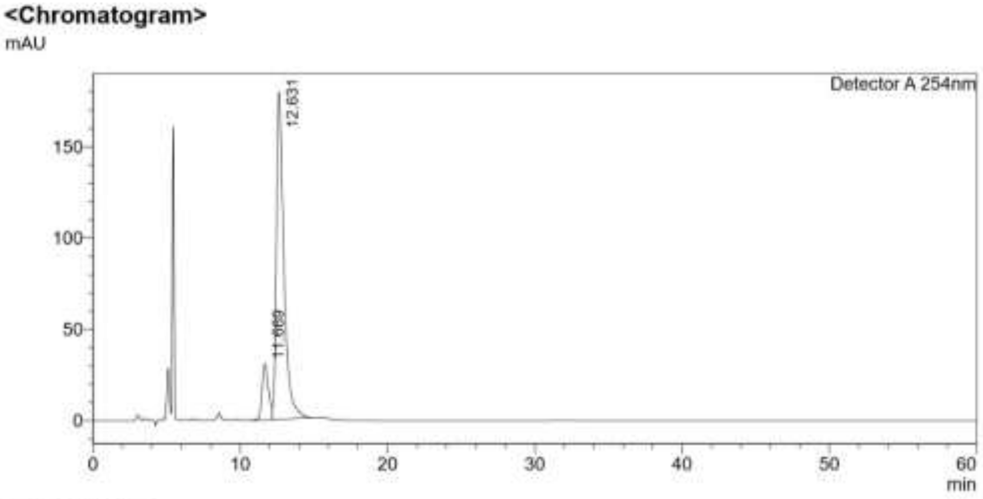

<Peak Table>

Detector A 254nm

Peak#	Ret. Time	Area	Height	Conc.	Unit	Mark	Name
1	11.669	928577	30596	12.643			
2	12.631	6416222	179081	87.357		V	
Total		7344799	209677				

^{1}H NMR ($CDCl_3$, 300 Hz) of (S_a)-**6a** from **4k-SI**

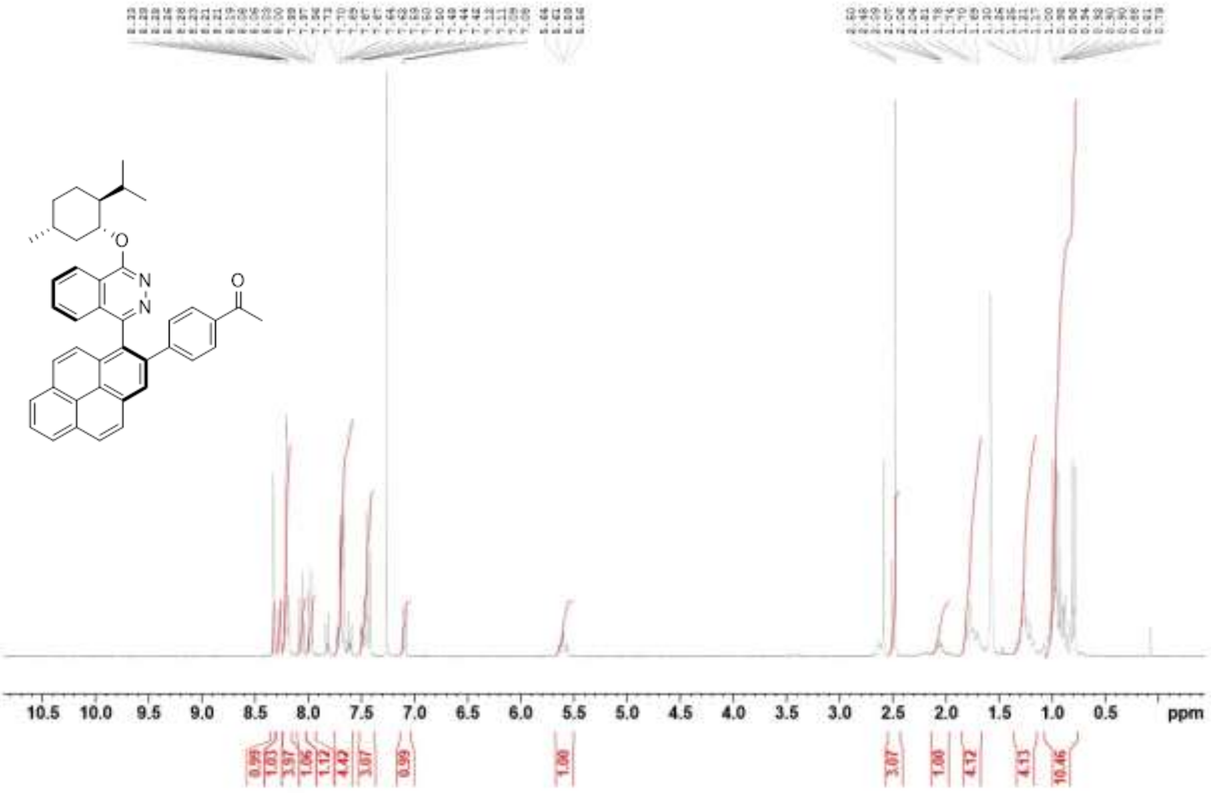

$^{13}C\{^{1}H\}$ NMR ($CDCl_3$, 125 Hz) of (S_a)-**6a** from **4k-SI**

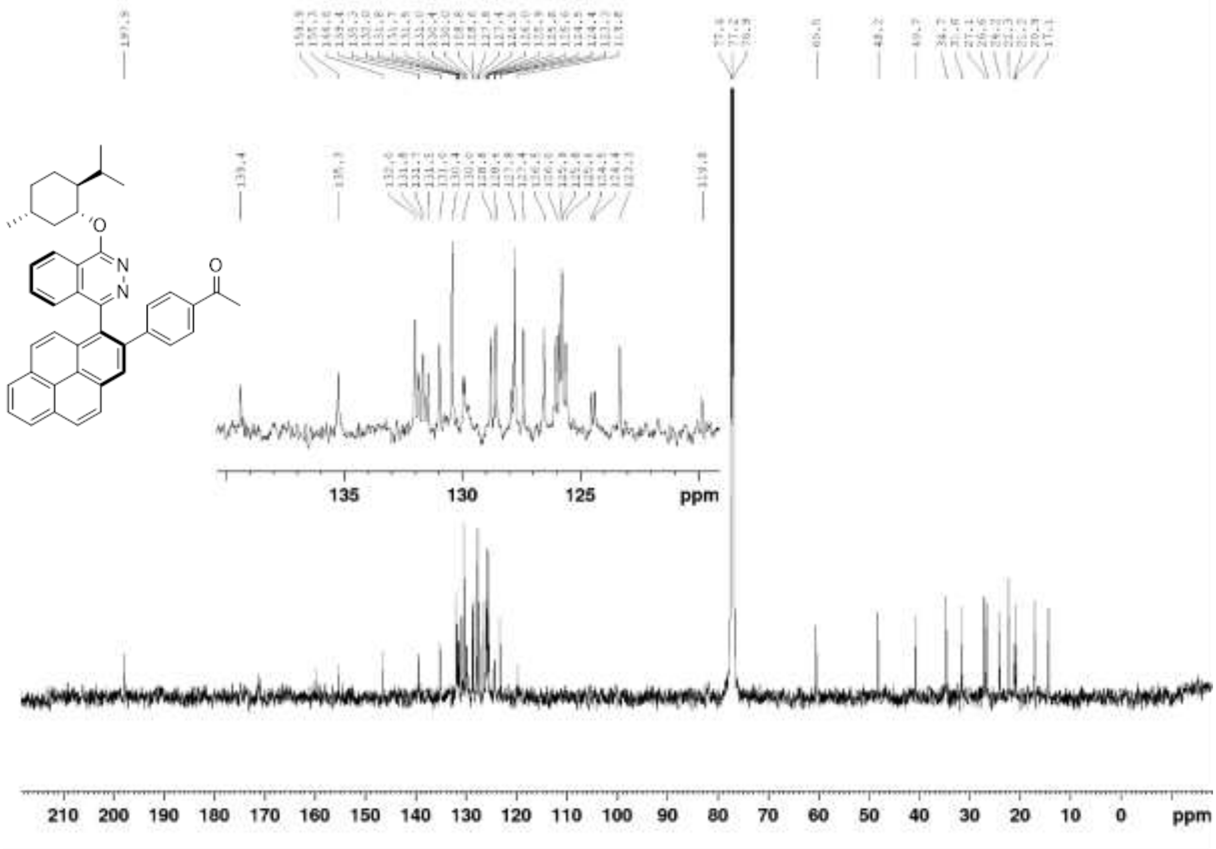

HPLC-chromatogram of (S_a)-**6a** from **4k-SI** (Table 2, entry 4)

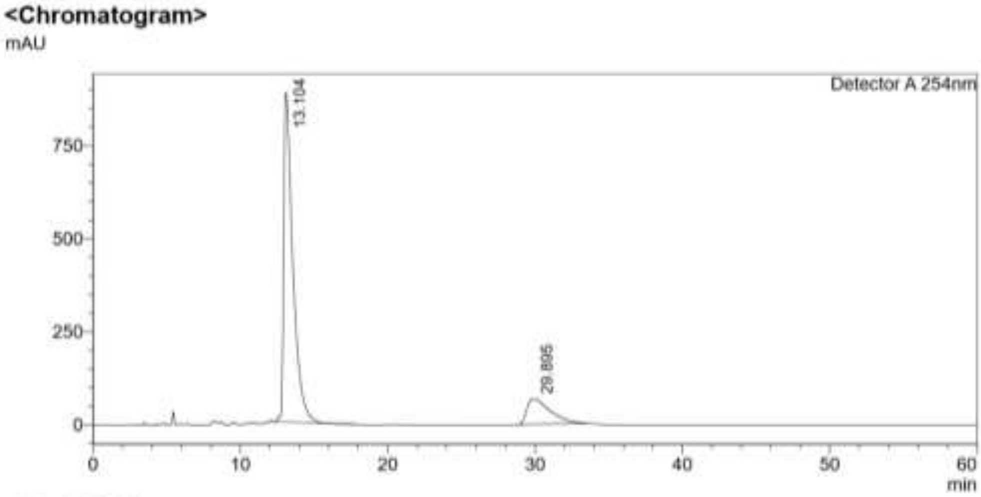

<Peak Table>

Detector A 254nm

Peak#	Ret. Time	Area	Height	Conc.	Unit	Mark	Name
1	13.104	38223425	884712	83.579		M	
2	29.895	7509867	68409	16.421		M	
Total		45733292	953122				

References

(1) (a) Herde, J. L.; Lambert, J. C.; Senoff, C. V.; Cushing, M. A., Cyclooctene and 1,5-Cyclooctadiene Complexes of Iridium(I), Inorganic Syntheses, (Ed.: Parshall, G. W.), John Wiley & Sons, Hoboken, USA **1974**, 18–20. (b) Tajuddin, H.; Harrisson, P.; Bitterlich, B.; Collings, J. C.; Sim, N.; Batasanov, A. S.; Cheung, M. S.; Kawamorita, S.; Maxwall, A. C.; Shukla, L.; Morris. J.; Lin, Z.; Marder, T. B.; Steel, P. G., Iridium–catalyzed C–H borylation of quinolines and unsymmetrical 1,2-disubstituted benzenes: insights into steric and electronic effects on selectivity. *Chem. Sci.* **2012**, *3*, 3505.

(2) Li, J.; Lei, Y.; Yu, Y.; Qin, C.; Fu, Y.; Li, H.; Wang, W., $Co(OAc)_2$-Catalyzed Trifluoromethylation and C(3)-Selective Arylation of 2-(Propargylamino)pyridines via a 6-Endo-Dig Cyclization. *Org. Lett.* **2017**, 19, 6052.

(3) Chen, D.; Xu, G.; Zhou, O.; Chung, L. W.; Tang, W., Practical and Asymmetric Reductive Coupling of Isoquinolines Templated by Chiral Diborons. *J. Am. Chem. Soc.* **2017**, *139*, 9767.

(4) Zhou, M.; Li, K.; Chen, D.; Xu, R.; Xu, G.; Tang, W., Enantioselective Reductive Coupling of Imines Templated by Chiral Diboron. *J. Am. Chem. Soc.* **2020**, *142*, 10337.

(5) Wilking, M.; Daniliuc, C. G.; Hennecke, U. Monomeric Cinchona Alkaloid-Based Catalysts for Highly Enantioselective Bromolactionisation of Alkynes. *Chem. Eur. J.* **2016**, *22*, 18601.

(6) After initial investigations for the synthesis of (+)-**2a**, it was deemed crucial to only use a very minute excess of (+)-menthol, since the alcohol is eluting very similarly to the desired product and would impose a non-trivial impurity for all subsequent steps. This is highlighted by the reaction utilizing 3,6-dichloro-4,5-dimethylpyridazine (**SI-1**) as starting material with (+)-menthol as an inseparable major impurity, which however did neither inhibit nor promote the borylation reaction.

(7) Ros, A.; Estepa, B.; López-Rodriguez, R.; Álvarez, E.; Fernández, R.; Lassaletta, J. M. use of Hemilabile N,N ligands in Nitrogen-directed Iridium-Catalyzed Borylations of Arenes. *Angew. Chem. Int. Ed.* **2011**, *50*, 11724.

(8) Tagata, T.; Nishida, M. Palladium Charcoal-Catalyzed Suzuki−Miyaura Coupling To Obtain Arylpyridines and Arylquinolines. *J. Org. Chem.* **2003**, *68*, 9412.

(9) Song, C.; Ma, Y.; Chai, Q.; Ma, C.; Jiang, W.; Andrus, M. B., Palladium catalyzed Suzuki–Miyaura coupling with aryl chlorides using a bulky phenanthryl N-heterocyclic carbene ligand. *Tetrahedron* **2005**, *61*, 7438.

(10) Guo, X.; Dang, H.; Wisniewski, S. R.; Simmons, E. M., Nickel-Catalyzed Suzuki–Miyaura Cross-Coupling Facilitated by a Weak Amine Base with Water as a Cosolvent. *Organometallics* **2022**, *41*, 1269.

(11) SHELXS-97: Sheldrick, G. M. *Acta Cryst.* **2008**, *A64*, 112.

(12) SHELXL-2018: Sheldrick, G. M. *Acta Cryst.* **2015**, *C71*, 3.
